# Chemical Structure-Related Drug-Like Criteria of Global Approved Drugs

**DOI:** 10.3390/molecules21010075

**Published:** 2016-01-12

**Authors:** Fei Mao, Wei Ni, Xiang Xu, Hui Wang, Jing Wang, Min Ji, Jian Li

**Affiliations:** Shanghai Key Laboratory of New Drug Design, School of Pharmacy, East China University of Science and Technology, 130 Mei Long Road, 200237 Shanghai, China; maofei@ecust.edu.cn (F.M.); niwei@ecust.edu.cn (W.N.); 10121434@mail.ecust.edu.cn (X.X.); 10121433@mail.ecust.edu.cn (H.W.); 10121432@mail.ecust.edu.cn (J.W.); 10121437@mail.ecust.edu.cn (M.J.)

**Keywords:** drug-like property, chemical structure-related criteria, ring system, functional group, the heavy atoms proportion, drug design

## Abstract

The chemical structure of a drug determines its physicochemical properties, further determines its ADME/Tox properties, and ultimately affects its pharmacological activity. Medicinal chemists can regulate the pharmacological activity of drug molecules by modifying their structure. Ring systems and functional groups are important components of a drug. The proportion of non-hydrocarbon atoms among non-hydrogen atoms reflects the heavy atoms proportion of a drug. The three factors have considerable potential for the assessment of the drug-like properties of organic molecules. However, to the best of our knowledge, there have been no studies to systematically analyze the simultaneous effects of the number of aromatic and non-aromatic rings, the number of some special functional groups and the proportion of heavy atoms on the drug-like properties of an organic molecule. To this end, the numbers of aromatic and non-aromatic rings, the numbers of some special functional groups and the heavy atoms proportion of 6891 global approved small drugs have been comprehensively analyzed. We first uncovered three important structure-related criteria closely related to drug-likeness, namely: (1) the best numbers of aromatic and non-aromatic rings are 2 and 1, respectively; (2) the best functional groups of candidate drugs are usually -OH, -COOR and -COOH in turn, but not -CONHOH, -SH, -CHO and -SO_3_H. In addition, the -F functional group is beneficial to CNS drugs, and -NH_2_ functional group is beneficial to anti-infective drugs and anti-cancer drugs; (3) the best R value intervals of candidate drugs are in the range of 0.05–0.50 (preferably 0.10–0.35), and R value of the candidate CNS drugs should be as small as possible in this interval. We envision that the three chemical structure-related criteria may be applicable in a prospective manner for the identification of novel candidate drugs and will provide a theoretical foundation for designing new chemical entities with good drug-like properties.

## 1. Introduction

The discovery of small molecule drug is complex and difficult. The traditional small molecule drug development process usually preferentially considers the efficacy of a molecule, then assesses its drugability, which often leads to high failure rates and development costs. As reported, obtaining one approved drug required about 30.4 preclinical new chemical entities (NCEs) in 2007–2011, while only 12.4 NCEs were needed in 2003–2007 [1]. Thus, the more and more new drug research and development costs may be ascribed to the increased failure rate in preclinical and clinical experiments. Therefore, useful methods to improve the success rate of drug research and development are particularly noteworthy.

The chemical structure of a drug determines its physicochemical properties, and further determinates its absorption, distribution, metabolism, excretion and toxicity (ADME/Tox) properties, and ultimately affect the pharmacological activity of the drug molecule. Medicinal chemists can regulate the pharmacological activity of drug molecules by modifying their structure. Because approved drugs have already passed strict pre-clinical and clinical studies, there is no doubt that these drugs have good drug-like properties. If the common characteristic of the chemical structures of the global small molecular drugs could be summarized as a criterion to guide the selection, design and optimization of lead compounds and drug candidates in the early stages of preclinical research, it would not only increase the success rate of drug development, but also eliminate those poor drug-like compounds in advance and avoid more research and development expenses. Therefore, the study of the key chemical structure characteristics of small molecule drugs has significant theoretical and practical value.

### 1.1. Drug-Like Properties

Drug-like properties are defined by Sugiyama as the physicochemical properties (such as solubility, stability, * etc.*) and biological characteristics (ADME/Tox) characteristics that are consistent with good clinical performance [2]. According the research findings of Lipinski *et al.* [3], the term drug-like compound refers to those compounds with acceptable ADME/Tox properties, and able to survive Phase I clinical trials. Borchardt [4] pointed out that the responsibility of medicinal chemists is not only to optimize the pharmacological activity of drug molecules, but also optimizing their drug-like properties. Therefore, an organic molecule approved for a disease must have enough good pharmacological activity as well as drug-like properties.

Since the Lipinski “rule of five” was introduced in 1997 [5], researchers have focused on the drug-like property rules of lead compounds increased. The physicochemical properties involved in these studies are mainly molecular weight (MW), lipophilicity (the logarithm of the octanol-water distribution coefficient, logP), numbers of hydrogen bond donors and acceptors (HBD and HBA), rotatable bonds (ROT), number of rings, polar surface area (PSA) and acid/base properties [6,7,8,9,10].

Drug-like properties research aims to guide workers to design compounds with potentially good ADME/Tox properties in the early phase of drug discovery and to a certain extent, reduce the failure rate and cost of drug research and development. In order to achieve this goal, how to improve the drug-like properties of organic molecules has also been widely studied by medicinal chemists. For example, the presence of a non-ionogenic group instead of an ionogenic group will improve the permeability of an organic molecule, thus affecting its *in vivo* oral bioavailability [11]. Decreasing the hydrogen bonds and increasing the lipophilicity will enhance the ability of an organic molecule to cross the blood brain barrier [12].

### 1.2. Chemical Structure Properties and Drug-Like Properties

Since 2001, when the “property-based design” concept was presented by van de Waterbeemd *et al.*, [13] there has been an increased focus on its application in drug design and discovery [14,15,16,17,18,19,20,21,22]. The relationship between chemical structure and physicochemical properties has attracted the attention of medicinal chemists as a new drug research and development strategy that complements the structure-activity relationships in the progress of drug design and discovery.

The chemical structure of a drug influences its physicochemical properties, and the physicochemical properties of a drug molecule [23], such as MW, lipophilicity, aqueous solubility (S), permeability, acid-base ionization constant (pKa), HBD and HBA, ROT and PSA, can be changed by modifying its structure. Further, these physicochemical properties of drug molecule influences its ADME/Tox properties (drug-like properties), such as metabolic stability, plasma stability, P-glycoprotein (Pgp) extrusion, serum albumin binding, cytochrome P450 (CyP450) inhibition, human Ether-à-go-go-Related Gene (hERG) inhibition, the ability to across the blood brain barrier (BBB), pharmacokinetics (PK) and toxicity. Ultimately, these physicochemical properties and the ADME/Tox properties of a drug molecule affect its pharmacodynamics activity, for example, the lower pharmacodynamic activity of central nervous system (CNS) drugs *in vivo* may be attributed to the lower ability to cross the blood brain barrier (BBB).

In this context, we planned to analyze the chemical structure properties of globally approved small drugs (including five sub-databases extracted from the whole database) from three points of view, *i.e.*, the number of aromatic and non-aromatic rings, the number of some special functional groups and the proportion of heavy atoms, hoping to obtain some structure-related criteria which could be applicable in the identification of novel candidate drugs and provide a theoretical foundation for designing new chemical entities with good drug-like properties.

## 2. Results and Discussion

### 2.1. Numbers of Aromatic and Non-Aromatic Rings Analysis of Our Approved Drugs Database

Rings are a structural unit that exists widely in organic molecules and they differ from the chain structure, which is not only reflected in the conformation, but also in the physicochemical properties of drugs. According to the definition of aromaticity proposed by the German chemist Hückel in 1931 based on the molecular orbital theory, ring structures can be divided into two major categories, aromatic rings and non-aromatic rings. In addition, according to the number of atoms in the ring structure, they can be divided into three ring, four ring, five ring, six ring, seven ring and so on. They also can be divided into carbocycles and heterocycle according to the category of atoms in the ring structure. Further, heterocycles can be divided into oxygen heterocycles, nitrogen heterocycles, sulfur heterocycles and so on. Among these ring structures, aromatic rings (including carbo-aromatic rings and hetero-aromatic rings) are the most common part in the structures of small organic molecule drug.

In 1996, Bemis and Murcko [24] analyzed the 5120 drugs molecular skeletons in the Comprehensive Medicinal Chemistry (CMC) database. They adopted two ways to analyze the skeletons, without or with regard to atom type, hybridization, and bond order. Using the first analytical method, there were 1179 different frameworks among the 5120 compounds and half of the drugs contained the top 32 frequently occurring frameworks. With the second analytical method, there were 2560 different frameworks and the drugs with the top 42 frequently occurring frameworks accounted for only a quarter. Furthermore, the most common framework was the benzene ring. They further studied the common features present in drug molecules by investigating the drug side chains based on the second analytical method in 1999 [25]. There were 1246 different side chains among the 5090 compounds analyzed. The average number of side chains per molecule was 4, and the average number of heavy atoms per side chain was 2. The number of side chains was less than five in the structure of 80% of the drugs. The most and least commonly found labeled side chain pairs were carbonyl/carbonyl (C=O/C=O) and carbon-amino/sulfoxide (C-NH_2_/S=O).

In 1999, Ghose *et al.* [26] also analyzed the CMC database and seven different subsets belonging to different classes of drug molecules and identified some drug-like properties. They also thought benzene was the most abundant substructure in the CMC database, slightly more abundant than all the heterocyclic rings combined. Non-aromatic heterocycls were twice as abundant as aromatic heterocycles. The top three abundant functional groups in this database were tertiary aliphatic amine, alcohol hydroxyl and carboxamide.

In 2005, Jiang *et al.* [27] developed a new chemistry space filter for distinguishing a drug-like database from a nondrug-like database by analyzing the properties of compounds in MACCS-II Drug Data Report (MDDR), CMC, and Available Chemicals Directory (ACD). Their results demonstrate that the proportion of drugs containing non-aromatic rings in the non-drug like database, ACD, was lower than in the drugs in the drug-like databases, MDDR and CMC.

In 2009, Ritchie *et al.* [28] analyzed the impact of aromatic ring count (the number of aromatic and heteroaromatic rings) in molecules against various properties such as aqueous solubility, lipophilicity, serum albumin binding, CyP450 inhibition and hERG inhibition and concluded that oral drug candidates containing fewer aromatic rings are more developable. In addition, a molecule containing more than three aromatic rings may be a poorer developability compound. Furthermore, the addition of aromatic heterocycles will have a lesser effect on increasing the lipophilicity than carbon-containing aromatics, but will increase PSA and this might begin to reduce oral absorption and/or cell penetration. In 2011, this group further analyzed the impact of ring count (carbo-aromatic ring hetero-aromatic ring, carbo-aliphatic ring and hetero-aliphatic ring on compound developability. The results indicated that increasing ring counts have detrimental effects on developability in the order carboaromatics > heteroaromatics > carboaliphatics > heteroaliphatics [29]. They also analysed and compared the aqueous solubility, protein binding and CYP450 inhibition data of compounds containing a variety of heteroaromatic and heteroaliphatic rings to determine which ring types fared best and worst in these developability screens [30]. The results suggest that certain hetero-rings were generally more developable than others.

In 2014, Ward *et al.* [31] discussed “what does the aromatic ring number mean for drug design?”, pointing out that current evidence implied that drug molecules with more three aromatic rings were undesirable on compound developability and that heteroaromatics performed better than carboaromatics overall.

In the same year, Taylor *et al.* [32] analyzed the rings, ring systems and frameworks in drugs listed in the FDA Orange Book and listed the ring systems of the top 100 most frequent—the first one was benzene ring; both pyridine and piperidine were the second. Piperazine was the fourth one.

To explore the effect of nitrogen heterocycles on pharmaceuticals, Njardarson *et al.* [33] mainly analyzed the structural diversity, substitution patterns, and frequency of nitrogen heterocycles among U.S. FDA approved pharmaceuticals in 2014 by dividing them into seven categories: (1) three- and four-membered nitrogen heterocycles; (2) five-membered nitrogen heterocycles; (3) six-membered nitrogen heterocycles; (4) seven- and eight-membered nitrogen heterocycles; (5) fused nitrogen heterocycles; (6) bridged bicyclic nitrogen heterocycles; and (7) macrocyclic nitrogen heterocycles, and reported on the top 25 most commonly utilized nitrogen heterocycles in U.S. FDA approved drugs.

Although there were a lot of studies focusing on the ring systems analysis of small molecule drugs databases, there have been no studies to systematically analyze the influence of the number of aromatic and non-aromatic rings simultaneously on the drug-like properties in a globally approved drugs database.

**Figure 1:**
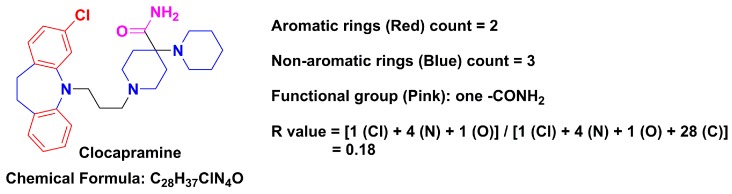
Example of aromatic ring count, non-aromatic ring count, functional group count and R value for clocapramine.

First, we counted the number of aromatic and non-aromatic rings of the 6891 drugs in our approved drugs database. The count rules were as follows: single rings, such as benzene or thiophene were identified as one aromatic ring, fused aromatic ring systems, such as a naphthalene ring were identified as two aromatic rings, a single non-aromatic ring, such as cyclopentane was identified as one non-aromatic ring, and a fused non-aromatic ring system, such as decahydronaphthalene was identified as two non-aromatic rings. Considering the particular case of bridged rings, we dealt with them separately, neither being included in the aromatic ring nor non-aromatic ring groups. A specific example of the aromatic and non-aromatic ring counts of a drug is shown in Figure 1 for clocapramine.

**Figure 2:**
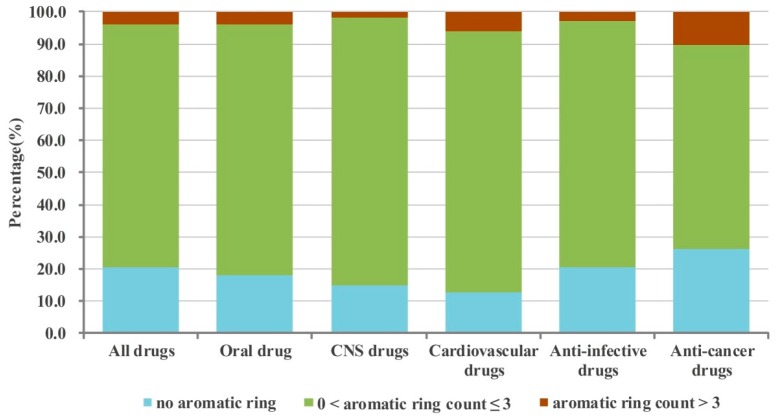
The percentage of different aromatic drugs in the whole database and five sub-databases.

**Figure 3:**
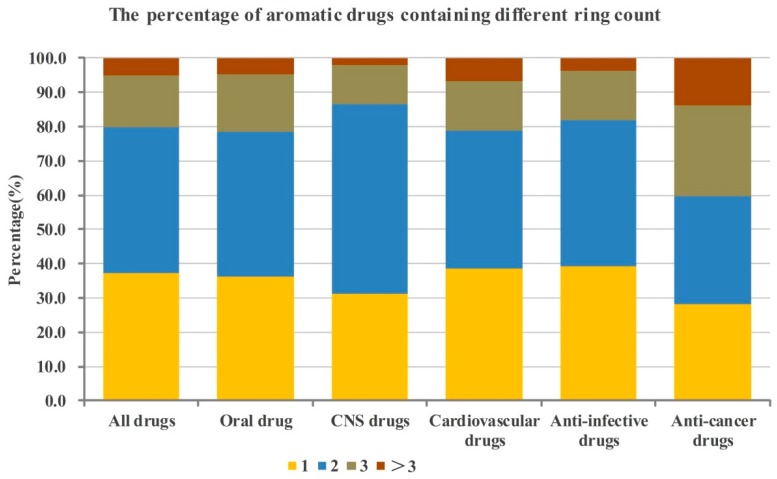
The percentage of aromatic drugs containing different ring count in the whole database and five sub-databases. The bars represent the percentages of drugs with different aromatic ring count in corresponding aromatic drugs databases.

**Figure 4:**
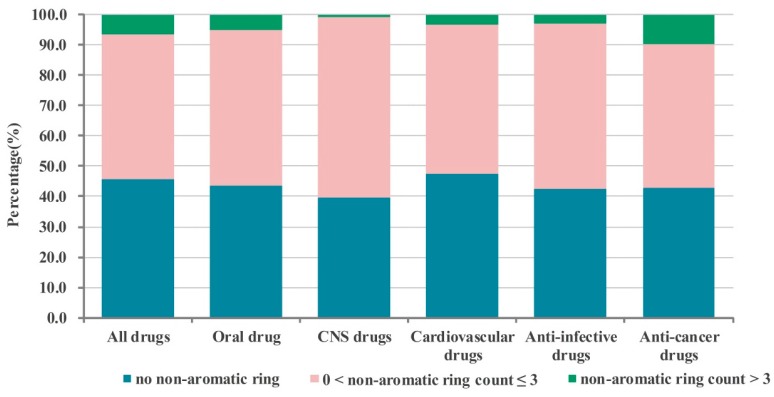
The percentage of different non-aromatic drugs in the whole database and five sub-databases.

**Figure 5:**
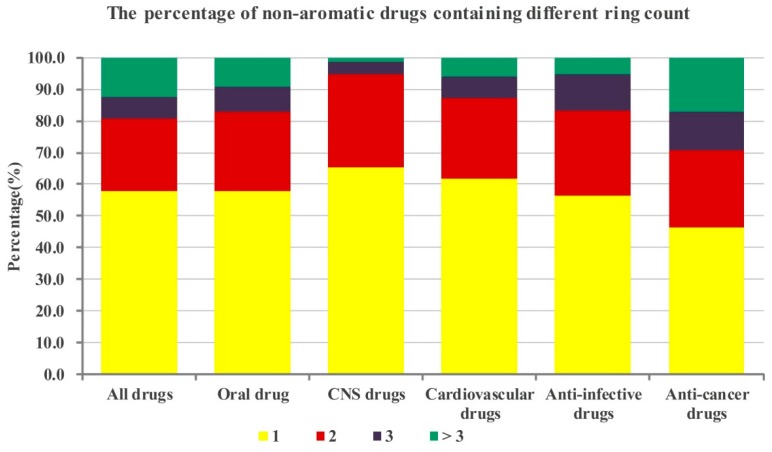
The percentage of non-aromatic drugs containing different ring counts in the whole database and five sub-databases. The bars represent the percentages of drugs with different non-aromatic ring counts in the corresponding non-aromatic drugs databases.

Then according to the number of aromatic and non-aromatic rings, the 6891 drugs were divided into different categories of drugs, such as drugs containing no aromatic rings, drugs containing one aromatic ring, drugs containing two aromatic rings, drugs containing three aromatic rings, drugs containing more than three aromatic rings, drugs containing no non-aromatic rings, drugs containing one non-aromatic rings, drugs containing two non-aromatic rings, drugs containing three non-aromatic rings, drugs containing more than three non-aromatic rings. The numbers of them in the different database categories are shown in Table 1 and Table 2. The percentages of the different categories of drugs are shown in Figure 2, Figure 3, Figure 4 and Figure 5.

**Table 1:** Number of drugs containing aromatic rings in the whole database and five sub-databases.

Number of Aromatic Rings	All Drugs	Oral Drug	CNS Drugs	Cardiovascular Drugs	Anti-Infective Drugs	Anti-Cancer Drugs
0	1245	164	138	112	174	108
1	2041	313	298	343	281	92
2	2341	365	529	360	307	103
3	826	145	110	129	104	86
>3	272	40	20	60	26	45

**Table 2:** Number of drugs containing non-aromatic rings or bridge rings in the whole database and five sub-databases.

Number of Non-Aromatic Rings	All Drugs	Oral Drug	CNS Drugs	Cardiovascular Drugs	Anti-Infective Drugs	Anti-Cancer Drugs
0	2985	435	416	466	372	182
1	2164	343	443	332	293	117
2	854	147	199	138	140	61
3	258	47	29	35	60	31
>3	464	55	8	33	27	43
bridge rings	166	24	27	17	13	8

#### 2.1.1. Numbers of Aromatic Rings Analysis

From the analysis results indicated in Figure 2 we can notice that more than seventy percent of drugs contain at least one aromatic ring. In detail, nearly eighty percent of all drugs and anti-infective drugs contain at least one aromatic ring (79.5% and 79.3%, respectively); more than eighty percent of oral drugs contain at least one aromatic ring (82.1%); more than eighty-five percent of CNS drugs and cardiovascular drugs contain at least one aromatic ring (85.3% and 87.4%, respectively). The proportion of anti-cancer drugs that contain at least one aromatic ring is the least among these categories of drugs analyzed, but it is still high (73.8%). As shown in Table 1 in Figure 2, the numbers of aromatic rings of most of drugs were not more than three, and the proportion of them are as follows (Figure 2): all drugs: 75.6%, oral drugs: 78.3%; CNS drugs: 83.5%; cardiovascular drugs: 81.5%; anti-infective drugs: 76.5%; anti-cancer drugs: 63.6%.

Further, we elaborately analyzed the ring composition of drugs containing aromatic rings, that is the proportion of drugs containing one (or two or three or more) aromatic rings in all drugs possessing aromatic rings. The results shown in Figure 3 indicate that among the drugs possessing aromatic rings, most of drugs having one/two aromatic rings (all drugs: 79.9%, oral drugs: 78.5%; CNS drugs: 86.4%; cardiovascular drugs: 78.9%; anti-infective drugs: 81.9%) except the anti-cancer drugs (59.8%). Different with other categories of drugs, the proportions of anti-cancer drugs having one, two and three aromatic rings are almost the same, 28.2%, 31.6% and 26.4%, respectively. Obviously, among these different categories of drugs, CNS drugs possess the largest proportion that contain two aromatic rings (55.3%).

These statistical results indicate that candidate drugs with less than four aromatic rings (optimally one or two, except anti-cancer drugs, which also tolerate up to three aromatic rings) may possess good drug-like properties and be likely to be developed into approved drugs.

#### 2.1.2. Numbers of Non-aromatic Rings Analysis

According to the analysis result indicated in Figure 4, we can notice that more than half of drugs contain at least one non-aromatic ring and there is no significant difference among the different categories of drugs. The proportions of them are as follows: all drugs: 54.2%, oral drugs: 56.3%; CNS drugs: 60.5%; cardiovascular drugs: 52.7%; anti-infective drugs: 57.5%; anti-cancer drugs: 56.9%.

According to the data in the Table 2 of Figure 4, the numbers of non-aromatic rings of most of drugs were not more than three. The proportion of them not more than three and more than three are as follows: all drugs: 47.5% *vs.* 6.7%, oral drugs: 51.1% *vs.* 5.2%; CNS drugs: 59.8% *vs.* 0.7%; cardiovascular drugs: 49.5% *vs.* 3.2%; anti-infective drugs: 54.5% *vs.* 3.0%; anti-cancer drugs: 47.2% *vs.* 9.7%.

Further, we also analyzed the composition of drugs containing non-aromatic rings, that is the proportion of drugs containing one (or two or three or more) non-aromatic rings in all drugs possessing non-aromatic rings. The results shown in Figure 5 indicate that among the drugs possessing non-aromatic rings, most of drugs having one/two non-aromatic rings (all drugs: 80.7%, oral drugs: 82.7%; CNS drugs: 94.5%; cardiovascular drugs: 87.4%; anti-infective drugs: 83.2%; anti-cancer drugs: 70.6%). All the sub-databases have more than half of drugs containing one non-aromatic ring except the anti-cancer drugs sub-database. Like the case of aromatic rings, among these different categories of drugs, CNS drugs possess the largest proportion containing one or two non-aromatic rings (65.2% and 29.3%). These statistical results indicate that if a candidate drug contains a non-aromatic ring, the number of them should be less than four (and optimally one or two).

#### 2.1.3. Crossed Analysis of the Numbers of Aromatic and Non-aromatic Rings

To study the relationship of numbers of aromatic and non-aromatic rings of drugs, we elaborately analyzed the ring distributions of drugs in the whole database and five sub-databases. The results were shown in Table 3. We also calculated the proportion of drugs containing different rings (only aromatic rings, only non-aromatic rings, both aromatic and non-aromatic rings, no rings, bridge rings) in the whole database and five sub-databases.

**Table 3:** The proportion of drugs with different rings in the whole database and five sub-databases.

Classes	Number of Rings ^a^	0	1	2	3	>3
All drugs	0	5.8	12.1	16.5	6.6	2.3
	1	3.9	11.1	11.8	3.6	1.1
	2	2.4	4.2	4.2	1.3	0.4
	3	0.9	1.4	1.2	0.3	0.1
	>3	5.2	0.9	0.3	0.3	0
Oral drugs	0	4.5	11.1	15.9	7.5	2.4
	1	3.6	11.6	12.4	4.1	1.0
	2	2.1	4.5	5.1	1.9	0.4
	3	1.0	2.1	1.1	0.1	0.1
	>3	4.4	0.5	0.2	0.2	0
CNS drugs	0	5.3	8.8	18.3	3.8	0.8
	1	5.2	12.0	17.4	4.2	0.7
	2	1.4	4.9	9.7	1.5	0.2
	3	0.1	0.6	1.7	0.2	0
	>3	0.3	0.2	0.1	0.1	0.1
Cardiovascular drugs	0	3.6	14.1	17.3	7.1	3.4
	1	2.0	12.8	12.3	3.1	2.3
	2	2.4	5.3	4.2	1.5	0.2
	3	0.5	0.9	1.2	0.9	0
	>3	2.6	0.5	0.2	0	0
Anti-infective drugs	0	3.3	10.5	17.3	7.8	2.1
	1	4.4	13.0	11.9	2.7	0.3
	2	6.3	4.4	3.8	0.9	0.1
	3	3.3	2.2	0.7	0.1	0.3
	>3	1.9	0.9	0.2	0	0
Anti-cancer drugs	0	11.1	9.5	7.7	7.9	5.0
	1	6.6	6.6	6.8	5.0	1.6
	2	3.2	2.3	2.5	2.9	3.0
	3	0.7	1.4	4.1	0.7	0.2
	>3	2.9	1.1	2.2	3.0	0.4

^a^ “1, 2, 3, > 3” in row represent the number of aromatic rings. “1, 2, 3, > 3” in column represent the number of non-aromatic rings.

As indicated in Table 4, the drugs without any rings or containing bridge rings account for only a small proportion. The drugs containing only non-aromatic ring are in range of 7.0%–15.9%. The drugs containing only aromatic rings are in range of 30.1%–42.0%. The drugs containing both aromatic and non-aromatic rings are in range of 41.5%–53.6%. Among drugs containing only aromatic rings, the drugs containing one or two aromatic ring (optimally two) are the majority, except for anti-cancer drugs (one: 9.5%, two: 7.7%, three: 7.9%). Among drugs containing both aromatic and non-aromatic rings, the drugs containing one or two aromatic and one non-aromatic ring are the majority. In addition, it is noticeable that the drugs containing no ring or bridge rings are the minority, and in range of 3.3%–11.1% and 1.4%–2.4%, respectively.

**Table 4:** The proportion of drugs containing only aromatic rings, only non-aromatic rings, both aromatic and non-aromatic rings, no rings and bridge rings in the whole database and five sub-databases.

Classes ^a^	Only Aromatic Rings	Only Non-Aromatic Rings	Both Aromatic and Non-Aromatic Rings	No Rings	Bridge Rings
All drugs	37.5 (12.1, 16.5) ^b^	12.4	42.0 (11.1, 11.8) ^c^	5.8	2.4
Oral drug	36.9 (11.1, 15.9) ^b^	11.1	45.2 (11.6, 12.4) ^c^	4.1	2.3
CNS drugs	31.7 (8.8, 18.3) ^b^	7.0	53.6 (12.0, 17.4) ^c^	5.3	2.4
Cardiovascular drugs	42.0 (14.1, 17.3) ^b^	7.3	45.3 (12.8, 12.3) ^c^	3.6	1.7
Anti-infective drugs	37.8 (10.5, 17.3) ^b^	15.9	41.5 (13.0, 11.9) ^c^	3.3	1.4
Anti-cancer drugs	30.1 (9.5, 7.7, 7.9) ^d^	13.3	43.7 (6.6, 6.8, 5.0) ^e^	11.1	1.8

^a^ The captions in each row represent drugs containing different rings; ^b^ The numbers in brackets represents the proportions of drugs with one aromatic ring and two aromatic rings, sequentially; ^c^ The numbers in brackets represents the proportions of drugs with one aromatic ring and one non-aromatic ring, two aromatic rings and one non-aromatic ring, sequentially; ^d^ The numbers in brackets represents the proportions of drugs with one aromatic ring, two aromatic rings and three aromatic rings, sequentially; ^e^ The numbers in brackets represents the proportions of drugs with two aromatic rings and one non-aromatic ring, two aromatic rings and two non-aromatic rings, two aromatic rings and three non-aromatic rings, sequentially.

In summary, the analysis results demonstated that candidate drugs should have one or two aromatic rings and zero or one non-aromatic ring. Particularly, anti-cancer candidate drugs also tolerate up to three aromatic rings.

### 2.2. Numbers of Special Functional Groups Analysis of Our Approved Drugs Database

In the process of structural modifications of lead compounds, medicinal chemists usually tend to keep one part of the structural unit (optimized for skeleton structure) unchanged, and optimize the structures of lead compounds and explore structure-activity relationship by introducing or changing various types of substituent groups (functional groups), to ultimately obtain candidate drugs with good pharmacological activities. As important components of organic molecule structures, functional groups obviously have a great influence on the drug-like properties, but what kinds of functional groups are suitable for the medicinal molecules with good drug-like properties and great possibility to be developed into approved drugs? What is the proper number of each functional group?

In 1999, Ghose *et al.* [26] analyzed the CMC database and the frequency of some functional groups in drugs, and pointed out that the top three most abundant functional groups in this database were tertiary aliphatic amine, alcoholic hydroxyl and carboxamide. They also discussed the applications of organic carbamates in drug design and medicinal chemistry in 2015. In 2009, Mirza *et al.* [34] studied 1493 (aryl-amine/nitro drugs) and 831 (sulphur/halogen compounds) marketed drugs from the DrugBank database, counted and analyzed the proportion of drugs with special functional groups, such as sulphur atoms, aromatic amines, nitro, halogen atoms and CNOH groups.

Considering the drug statistics reported by previous studies were from a single database, not covering the global drugs, and only parts of functional groups were analyzed. therefore, in order to comprehensively analyze the frequency of functional groups’ occurrence in approved drugs and the their relationship with the drug-like properties, we counted the frequency of occurrence of 16 kinds of functional groups of the 6891 drugs in our approved drugs database, including -F, -CF_3_, -CN, -NO_2_, -NH_2_, -OH, -SH, -CHO, -COOH, -CONHOH, -COOR, -CONH_2_, -SO_3_H, -SO_2_NH_2_, -PO_3_H, -AsO_3_H. There were no drugs containing -PO_3_H and-AsO_3_H. According to the number of functional groups and functional group categories, the 6891 drugs were divided into drugs containing one special functional group (for example drugs containing one F, drugs containing one CF_3_, drugs containing one CN), drugs containing two special functional groups and drugs containing more than two special functional groups. The percentages of drugs containing different categories of functional groups were shown in Table 5. Through the analysis of the data in Table 5, the functional group distribution of approved drugs reveals some noteworthy observations: (1) the top three most abundant functional groups in drugs were as follows: all drugs: -OH (33.3%), -COOR (20.7%), -COOH (14.9%); oral drugs: -OH (31.8%), -COOR (19.4%), -COOH (17.9%); CNS drugs: -OH (18.1%), -F (11.2%), -COOR (10.9%); cardiovascular drugs: -OH (35.1%), -COOR (24.3%), -COOH (14.5%); anti-infective drugs: -OH (39.5%), -COOH (29.5%), -NH_2_ (28.4%); anti-cancer drugs: -OH (41.4%), -COOR (21.7%), -NH_2_ (21.3%). Thus, in all databases, the most common functional group is the hydroxyl, as its occurrence frequency in all databases is the top one. Moreover, -COOR or -COOH are the second most common functional groups. The substituent -F, which is thought to be beneficial to the development of drugs [35,36,37,38] has a higher occurrence frequency in CNS drugs, but relatively lower occurrence frequency in the other drug sub-databases. The occurrence frequency of functional groups -NH_2_ in anti-infective drugs and anti-cancer drugs is higher than in the other sub-databases. (2) The bottom three abundant functional groups in drugs were listed as follows: all drugs: -CONHOH (0.2%), -SH (0.3%), -CHO (0.7%); oral drugs: -CONHOH (0.4%), -SO_3_H (0.5%), -SH (0.6%); CNS drugs: -CONHOH (0.1%), -SH (0.1%), -CHO (0.2%); cardiovascular drugs: -CONHOH (0.1%), -SO_3_H (0.1%), -SH (0.5%), -CHO (0.5%); anti-infective drugs: -SH (0%), -CONHOH (0.1%), -SO_2_NH_2_ (1.0%); anti-cancer drugs: -SH (0.2%), -SO_3_H (0.2%), -CHO (0.7%), -SO_2_NH_2_ (0.7%). Thus, in all databases, the most rare functional group is -SH, whose occurrence frequency is the bottom in all categories of drugs. Moreover, -CONHOH, -CHO and -SO_3_H functional groups are also very rare in approved drugs. (3) Drugs containing -OH functional groups account for more than 30% in all databases (all drugs: 33.3%; oral drugs: 31.8%; cardiovascular drugs: 35.1%; anti-infective drugs: 39.5%; anti-cancer drugs: 41.4%) except the CNS drugs sub-database (18.1%), and most (61.7%–93.1%) of them contain one or two -OH groups.

More than 10% of drugs in all databases contain -COOR functional groups, and most (60.4%–86.1%) of them have one. More than 7% of drugs in all the databases contain -NH_2_ functional groups, and most (81.7%–96.0%) of them have one. More than 4% of drugs in all the databases contain -F functional groups, and most (66.7%–81.7%) of them have one. More than 4% of drugs in all databases contain -COOH functional groups, and most (68.0%–93.7%) of them have one.

**Table 5:** The percentages of drugs containing different categories of functional groups in the whole database and five sub-databases.

Functional Groups	All Drugs			Oral Drug			CNS Drugs			Cardiovascular Drugs			Anti-Infective Drugs			Anti-Cancer Drugs		
	1 ^a^	2 ^a^	>2 ^a^	1 ^a^	2^ a^	>2 ^a^	1^ a^	2^ a^	>2 ^a^	1^ a^	2^ a^	>2 ^a^	1^ a^	2^ a^	>2 ^a^	1^ a^	2^ a^	>2 ^a^
-F	5.4	1.6	0.4	6.8	2.1	0.4	9.2	2.0	0	2.9	1.2	0.2	6.4	2.0	1.2	6.6	1.6	0.2
-CF_3_	2.5	0.2	0.1	2.8	0.4	0	3.9	0.4	0.1	2.3	0.1	0.2	0.9	0.1	0	1.8	0	0
-CN	2.4	0.2	0	2.1	0.5	0	2.0	0	0	3.8	0.3	0	2.0	0.3	0	1.1	0.5	0
-NO_2_	2.0	0.1	0	3.3	0.1	0	1.5	0	0	2.5	0	0	5.1	0.8	0	2.0	0.5	0
-NH_2_	9.3	1.1	0.5	11.1	1.2	0	6.8	0.5	0	7.1	0.3	0	23.2	2.7	2.5	17.6	3.2	0.5
-OH	20.0	7.2	6.1	19.1	6.2	6.5	14.0	2.9	1.2	24.1	6.2	4.8	19.0	7.2	13.3	14.3	11.3	15.8
-SH	0.3	0	0	0.5	0.1	0	0.1	0	0	0.5	0	0	0	0	0	0.2	0	0
-CHO	0.7	0	0	0.7	0	0	0.2	0	0	0.4	0	0.1	1.9	0	0	0.7	0	0
-COOH	12.9	1.7	0.3	16.8	1.0	0.1	3.6	0.6	0.2	12.3	2.2	0	26.9	2.5	0.1	7.7	2.7	0.9
-CONHOH	0.2	0	0	0.4	0	0	0.1	0	0	0.1	0	0	0	0.1	0	1.1	0	0
-COOR	16.1	3.6	1.0	14.1	4.5	0.8	9.4	1.3	0.2	14.7	7.6	2.0	13.6	3.5	1.3	13.1	3.8	4.8
-CONH_2_	3.5	0.1	0.1	4.5	0.1	0	5.5	0.4	0	3.0	0.2	0	5.2	0	0	3.4	0	0
-SO_3_H	0.7	0.2	0	0.3	0.2	0	0.2	0.3	0	0.3	0	0	1.2	0.3	0.1	0.2	0	0
-SO_2_NH_2_	1.3	0.1	0	2.6	0.1	0	0.7	0	0	0.7	0	0	0.9	0.1	0	0.7	0	0

^a^ “1, 2, 3, > 3” in row represent the number of functional groups.

In summary, regarding specific functional groups, most drugs preferably contain one and the -OH functional group, the most abundant functional group in all the drugs databases, is the optimal substituent choice when modifying the structures of lead compounds. The -F functional group, the second most abundant functional group in CNS drugs, is the optimal substituted choice when developing CNS candidate drugs. Moreover, the -NH_2_ functional group, the third most abundant functional group in anti-infective drugs and anti-cancer drugs, is the optimal substituent choice when developing anti-infective and anti-cancer candidate drugs.

### 2.3. Heavy Atoms Proportion Analysis of Our Approved Drugs Database

As is known to all, carbon and hydrogen are the two main basic elements to construct organic compounds, but besides these two elements, a drug molecule often contains many other elements, such as nitrogen, oxygen, sulfur, halogen, * etc.* As a result, it is significant and helpful to explore these issues as follows: what kinds of elements are beneficial to the drug-like properties? What is their proper proportion? A candidate drug with what kinds of elements and element proportion may have a greater possibility to be developed into an approved drug? 

In 2014, Njardarson *et al.* [39] first studied the elemental composition (sulfur, chlorine, fluorine, phosphorous, bromine, iodine, and iron and so on) of U.S. FDA approved drug architectures. Then they further explored the distribution of sulfur and fluorine in 12 disease categories [40]. However their research only analyzed the distribution and frequency of specific elements in drug molecules, and did not conduct a proportion analysis of these elements.

In the process of optimizing the structure of lead compounds, medicinal chemists often tend to introduce non-hydrocarbon structures or groups. In order to study the effects of non-hydrocarbon atom (defined as heavy atoms) number on drug-like properties, we define the R value as the proportion of heavy atoms to non-hydrogen atoms (R = the number of non-hydrocarbon atoms/the number of non-hydrogen atoms). Through the statistics and analysis of R distribution in the whole database and different sub-databases, we can discuss the relationship between heavy atoms proportion among non-hydrogen atoms with drug-like properties. Therefore, we calculated the R value of the 6891 drugs in our approved drug database and analyzed their distribution in the whole database and five sub-databases.

**Figure 6:**
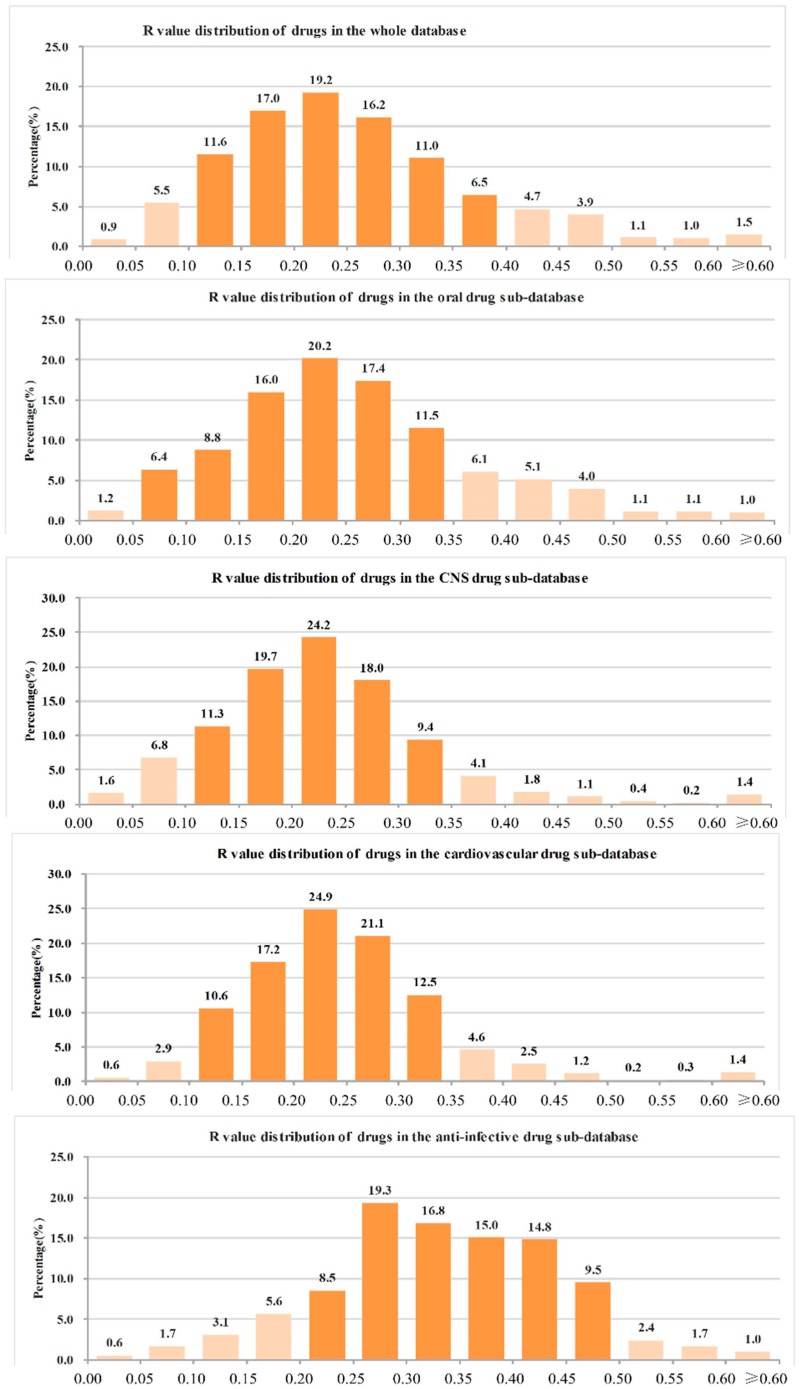

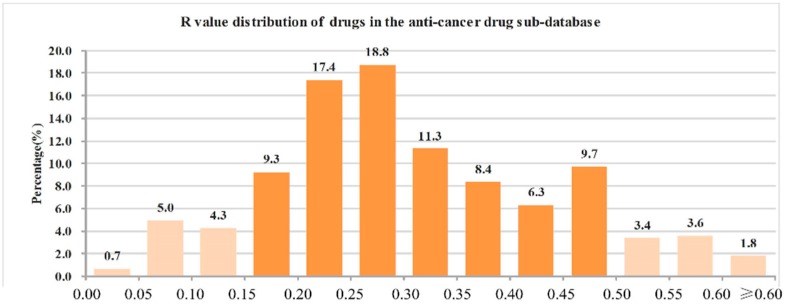
Heavy atoms proportion (R value) distribution of different kinds of approved drugs.

The number of different categories of drugs, mean, maximum and minimum of R value are shown in Table 6, and their R value distribution is shown in Figure 6. The data in Table 6 shows that the order of the R mean value of different categories of drugs are as follows: CNS drugs (0.24) < cardiovascular drugs (0.25) < oral drugs (0.26) < anti-cancer drugs (0.32) < anti-infective drugs (0.34), indicating that candidate CNS drugs, cardiovascular drugs and oral drugs with smaller heavy atoms proportions may possess good drug-like properties and be likely to be developed into approved drugs, while anti-cancer drugs and anti-infective drugs should have larger heavy atoms proportions.

**Table 6:** The number of different categories of drugs, mean, maximum and minimum of R value in our approved drug database.

Classes	Number of Drugs	Mean	Minimum	Maximum
All drugs	6891	0.26	0	1
Oral drugs	1051	0.26	0.04	0.82
CNS drugs	1122	0.24	0.04	0.80
Cardiovascular drugs	1021	0.25	0.04	0.80
Anti-infective drugs	905	0.34	0.02	0.86
Anti-cancer drugs	442	0.32	0	0.80

The analysis of the R value distribution of different kinds of approved drugs in Figure 6 reveals some noteworthy observations:

(1) The ranges of R values covering more than 90% of drugs were as follows: all drugs: 0.05–0.45 (91.6%); oral drugs: 0.05–0.45 (91.4%); CNS drugs: 0.05–0.40 (93.5%); cardiovascular drugs: 0.10–0.40 (90.9%); anti-infective drugs: 0.10–0.50 (92.7%); anti-cancer drugs: 0.05–0.50 (90.9%).
(2) The range of R values covering more than 70% of drugs were as follows: all drugs: 0.10–0.35 (75.0%); oral drugs: 0.10–0.35 (73.8%); CNS drugs: 0.10–0.30 (73.3%); cardiovascular drugs: 0.15–0.35 (75.7%); anti-infective drugs: 0.20–0.45 (74.5%); anti-cancer drugs: 0.15–0.45 (71.5%).
(3) The R values of more than 90% of drugs were not more than 0.50, and the details were as follows: all drugs: 96.4%; oral drugs: 96.7%; CNS drugs: 98.0%; cardiovascular drugs: 98.1%; anti-infective drugs: 94.9%; anti-cancer drugs: 91.2%.
(4) The R value of more than 70% of drugs was not more 0.40, and the details were as follows: all drugs: 87.8%; oral drugs: 87.5%; CNS drugs: 95.1%; cardiovascular drugs: 94.4%; anti-infective drugs: 70.6%; anti-cancer drugs: 75.1%.

In summary, the R value of candidate drugs in the range of 0.05–0.50 (preferred for 0.10–0.35) may indicate good drug-like properties. In this range, the R value of CNS drugs should be as small as possible, while for anti-cancer drugs it should be as larger as possible. If a candidate drug possesses good drug-like properties and has large possibility to be developed into an approved drug, its heavy atoms count should be not more than its carbon atom count (R value of more than 90% of drugs ≤ 0.50 and R value of more than 70% of drugs ≤ 0.40). Especially, for CNS drugs and cardiovascular drugs, the heavy atoms count should be not more than two-thirds of the carbon atom count (R value of 95.1% CNS drugs ≤ 0.40 and R value of 94.4% cardiovascular drugs ≤ 0.40).

## 3. Database Construction

### 3.1. Database Source and Data Collection

The 9990 approved drugs in our database were from the collection published in Nature in 2007 [41]. Firstly, after excluding antiseptics, pharmaceutical aids, therapeutic plants or animal extracts, vaccines, insecticides, surfactants and oligodeoxyribonucleotides, 8649 drug entities were left. In addition, combination drugs were recorded as two or more single components, and salts were recorded as the corresponding free acids or bases. Removing the combination drugs (80), adding their single components (160) and subtracting the duplicate components (227), the number of total drugs was 8502.

The chemical structures and Chemical Abstracts Service (CAS) Registry Numbers of these 8502 drugs were obtained through the SciFinder database [42] as the major source and the Drugbank database [43] as a minor one. The oral availability information about them were inquired through Thomson Reuters Integrity [44] and Cortellis for CI (Thomson Reuters Pharma) [45] database. According to the SciFinder and Drugbank database searches, the number of drug molecules without reported chemical structures was 996 and these were eliminated from the approved drugs database. Thus, the number of total drugs in the database was 7506.

**Figure 7:**
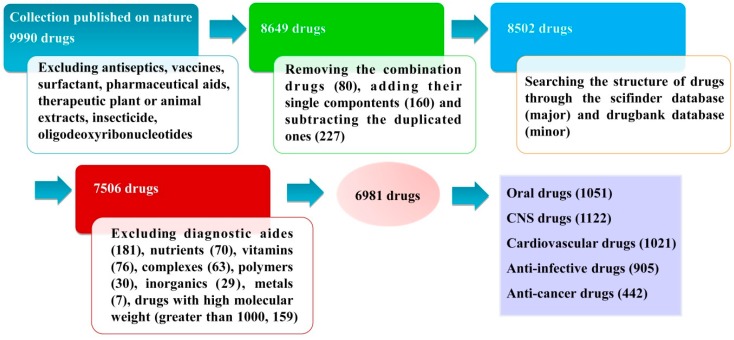
Schematic representation of the approved drugs database source, data collection and classification.

Secondly, in order to analyze the chemical structure property criteria of small organic molecule drugs, provide some new standards to evaluate drug-like properties and guide the design and structure optimization, the following drugs were further excluded: diagnostic aides (181), nutrients (70), vitamins (76), complexes (63), polymers (30), inorganics (29), metals (7), drugs with high molecular weight (greater than 1000, 159). Thus, 6891 small organic molecule drugs were left, forming the basis of database used in this paper. The common names, indications, CAS Registry Numbers and molecular formulas of all 6891 approved drugs are listed in Table S1 (see the Supplementary Materials). The data processing is schematically shown in Figure 7. Finally, the numbers of aromatic and non-aromatic rings, the numbers of some special functional groups (e.g., -F, -CF_3_, -CN, -NO_2_, -NH_2_, -OH, -SH, -CHO, -COOH, -CONHOH, -COOR, -CONH_2_, -SO_3_H, -SO_2_NH_2_) and the proportion of heavy atoms (R value) of the 6891 globally approved small drugs were counted, eventually forming the analysis database (LDD, L drug-like database) of this paper.

### 3.2. Database Classification

Among all the administration routes the oral route is generally the preferred choice for administration because it is the easiest and most convenient administration route and the most likely to be complied with by patients; BBB penetration ability is an essential feature for the CNS drugs [46]; the physicochemical properties of small molecule drugs for different indications may be quite different. Considering these factors and to carefully explore the structure-related drug-like properties of different categories of drugs, the LDD was divided into five sub-databases, including an oral drugs sub-database, a CNS drugs sub-database, an anti-cancer drugs sub-database, an anti-infective drugs sub-database, and a cardiovascular drugs sub-database (Figure 7).

#### 3.2.1. Oral Drugs

Whether the drugs are administered orally was determined from data in the Thomson Reuters Integrity and Cortellis for CI database. There were 1051 oral drugs in our final approved drugs database.

#### 3.2.2. CNS Drugs

CNS drugs include anticonvulsants, antidepressants, antipsychotics, anxiolytics, anti-Parkinson’s drugs, antiseizure drugs, sedatives, nootropics, and antimigraine drugs. There were 1122 CNS drugs in our final approved drugs database.

#### 3.2.3. Cardiovascular Drugs

Antianginals, antiarrhythmics, antihyperlipidemics, antihypertensives, antihypotensives, antithrombotics, cardiotonics and vasodilators belong to the cardiovascular drug class. There were 1021 cardiovascular drugs in our final approved drugs database.

#### 3.2.4. Anti-Infective Drugs

Anti-infective drugs include antibiotics, antibacterials, antifungals and antivirals. There were 905 anti-infective drugs in our final approved drugs database.

#### 3.2.5. Anti-Cancer Drugs

Drugs were indicated as antineoplastics. There were 442 anti-cancer drugs in our final approved drugs database.

## 4. Conclusions

The ring systems, functional groups, and heavy atoms proportion (R value) are three very important chemical structure-related characters, which are closely related to physicochemical properties and drug-like properties. Therefore, in this context, we analyzed three chemical structure-related properties of globally approved small molecule drugs (including five sub-database extracted from the whole database), * i.e.*, the numbers of aromatic and non-aromatic rings, the numbers of some special functional groups and the heavy atoms proportion.

The analysis results of rings count indicated that more than 70% of drugs contain at least one aromatic ring, and most drugs had one/two aromatic rings (optimized for two), except anti-cancer drugs, which could also have three aromatic rings. More than half of drugs contain at least one non-aromatic ring, among which most drugs have one/two non-aromatic rings (optimized for one). Through the crossed analysis of the numbers of aromatic and non-aromatic rings in our approved drugs database, we could conclude that the proportions of drugs containing only aromatic rings or both aromatic and non-aromatic rings were larger than others. Among the drugs containing only aromatic rings, the drugs containing one or two aromatic (optimized for two) are the majority, except for anti-cancer drugs. Among the drugs containing both aromatic and non-aromatic rings, the drugs containing one or two aromatic and one non-aromatic ring are the majority. The analysis results revealed that candidate drugs should have one or two aromatic rings and zero or one non-aromatic ring, and anti-cancer candidate drugs can tolerate three aromatic rings. This revelation can be used as a drug-like criterion to guide medicinal chemists to design active compounds with good drug-like properties.

Through the functional groups analysis of our approved drugs database, we can conclude the following: as for specific functional groups, most drugs prefer to contain one. The -OH functional group, the most abundant functional group in all the drugs databases, is the optimal substituent choice when modifying the structures of lead compounds. Roughly, the superior functional groups in drugs were -OH, -COOR and -COOH, while the inferior functional groups were -CONHOH, -SH, -CHO and -SO_3_H. In addition, the -F functional group is beneficial to CNS drugs, and the -NH_2_ functional group is beneficial to anti-infective and anti-cancer drugs. This is the second structure-related drug-like criterion to guide medicinal chemists when designing active compounds with good drug-like properties.

The third structure-related drug-like criterion to guide the medicinal chemists to design active compounds with good drug-like properties is correlated with the proportion of heavy atoms (R value). It is described as follows: candidate drugs with R values in the range of 0.05–0.50 (preferably 0.10–0.35) may possess good drug-like properties. In this range, the R value of CNS drugs should be as small as possible, while for anti-cancer drugs it should be as large as possible. If a candidate drug possesses good drug-like properties and has a good possibility of being developed into an approved drug, its heavy atoms count should be not more than its carbon atom count. Especially for CNS drugs and cardiovascular drugs, the heavy atoms count should be not more than two-thirds of the carbon atom count. The key points of our studies are summarized in Table 7.

**Table 7:** Highlights of the research.

The best numbers of aromatic and non-aromatic rings of candidate drugs are 2 and 1, respectively.
The best functional groups of candidate drugs are usually -OH, -COOR and –COOH.
The -F functional group is beneficial to CNS drugs.
The -NH_2_ functional group is beneficial to anti-infective drugs and anti-cancer drugs.
The best R value interval of candidate drugs is in the range of 0.05–0.50 (preferably 0.10–0.35).

We envision that these three chemical structure-related criteria may be applicable in a prospective manner for the identification of novel candidate drugs and will provide a theoretical foundation for designing new chemical entities with good drug-like properties.

## Supplementary Materials

The common names, indications, CAS register numbers and molecular formulas of all 6891 approved drugs are listed in the Supplementary materials. Supplementary materials can be accessed at: http://www.mdpi.com/1420-3049/21/1/75/s1.
