# Supplementary material for: Chemical Structure-Related Drug-Like Criteria of Global Approved Drugs
Source: Molecules. 2016 Jan 12;21(1):75. doi: 10.3390/molecules21010075 (PMC6273477; doi:10.3390/molecules21010075)
Supplement: Supplementary file 1 [file molecules-21-00075-s001.pdf]

Supplementary Materials: Chemical Structure-Related Drug-Like Criteria of Global Approved Drugs

Fei Mao [1], Wei Ni [1], Xiang Xu [1], Hui Wang [1], Jing Wang [1], Min Ji [1] and Jian Li *

Table S1. Common names, indications, CAS Registry Numbers and molecular formulas of 6891 approved drugs.

Common Name	Indication	CAS Number	Oral	Molecular Formula
Abacavir	Antiviral	136470-78-5	Y	$C_{14}H_{18}N_6O$
Abafungin	Antifungal	129639-79-8		$C_{21}H_{22}N_4OS$
Abamectin Component B1a	Anthelminithic	65195-55-3		$C_{48}H_{72}O_{14}$
Abamectin Component B1b	Anthelminithic	65195-56-4		$C_{47}H_{70}O_{14}$
Abanoquil	Adrenergic	90402-40-7		$C_{22}H_{25}N_3O_4$
Abaperidone	Antipsychotic	183849-43-6		$C_{25}H_{25}FN_2O_5$
Abecarnil	Anxiolytic	111841-85-1	Y	$C_{24}H_{24}N_2O_4$
Abiraterone	Antineoplastic	154229-19-3	Y	$C_{24}H_{31}NO$
Abitesartan	Antihypertensive	137882-98-5		$C_{26}H_{31}N_5O_3$
Ablukast	Bronchodilator	96566-25-5		$C_{28}H_{34}O_8$
Abunidazole	Antifungal	91017-58-2		$C_{15}H_{19}N_3O_4$
Acadesine	Cardiotonic	2627-69-2	Y	$C_9H_{14}N_4O_5$
Acamprosate	Alcohol Deterrant	77337-76-9	Y	$C_5H_{11}NO_4S$
Acaprazine	Nootropic	55485-20-6	Y	$C_{15}H_{21}Cl_2N_3O$
Acarbose	Antidiabetic	56180-94-0	Y	$C_{25}H_{43}NO_{18}$
Acebrochol	Steroid	514-50-1		$C_{29}H_{48}Br_2O_2$
Acebutolol	Antihypertensive	37517-30-9	Y	$C_{18}H_{28}N_2O_4$
Acecainide	Antiarrhythmic	32795-44-1	Y	$C_{15}H_{23}N_3O_2$
Acecarbromal	Sedative	77-66-7	Y	$C_9H_{15}BrN_2O_3$
Aceclidine	Cholinergic	827-61-2		$C_9H_{15}NO_2$
Aceclofenac	Antiinflammatory	89796-99-6	Y	$C_{16}H_{13}Cl_2NO_4$
Acedapsone	Antibiotic	77-46-3		$C_{16}H_{16}N_2O_4S$
Acediasulfone Sodium	Antibiotic	80-03-5		$C_{14}H_{14}N_2O_4S$
Acedoben	Nootropic	556-08-1		$C_9H_9NO_3$
Acefluranol	Steroid	83282-71-7		$C_{25}H_{26}F_2O_8$
Acefylline Clofibrol	Bronchodilator	70788-27-1		$C_{19}H_{21}ClN_4O_5$
Acefylline Piperazine	Bronchodilator	652-37-9		$C_9H_{10}N_4O_4$
Aceglatone	Antineoplastic	642-83-1		$C_{10}H_{10}O_8$
Aceglutamide	Antiulcerative	2490-97-3		$C_7H_{12}N_2O_4$
Acemetacin	Antiinflammatory	53164-05-9	Y	$C_{21}H_{18}ClNO_6$
Acenocoumarol	Anticoagulant	152-72-7	Y	$C_{19}H_{15}NO_6$
Aceperone	Adrenergic	807-31-8		$C_{24}H_{29}FN_2O_2$
Acepromazine	Sedative	61-00-7	Y	$C_{19}H_{22}N_2OS$
Aceprometazine	Antidepressant	13461-01-3	Y	$C_{19}H_{22}N_2OS$
Acetaminophen	Analgesic	103-90-2	Y	$C_8H_9NO_2$
Acetaminosalol	Analgesic	118-57-0		$C_{15}H_{13}NO_4$
Acetanilide	Analgesic	103-84-4		C_8H_9NO
Acetarsone	Antiprotozoal	97-44-9		$C_8H_{10}AsNO_5$
Acetazolamide	Antiglaucoma	59-66-5	Y	$C_4H_6N_4O_3S_2$
Acetergamine	Nootropic	3031-48-9		$C_{18}H_{23}N_3O$
Acetiromate	Antihyperlipidemic	2260-08-4		$C_{15}H_9I_3O_5$
Acetohexamide	Antidiabetic	968-81-0	Y	$C_{15}H_{20}N_2O_4S$
Acetohydroxamic Acid	Antiurolithic	546-88-3	Y	$C_2H_5NO_2$
Acetophenazine	Antipsychotic	2751-68-0	Y	$C_{23}H_{29}N_3O_2S$
Acetorphine	Sedative	25333-77-1		$C_{27}H_{35}NO_5$
Acetosulfone Sodium	Antibacterial	80-80-8		$C_{14}H_{15}N_3O_5S_2$
21-Acetoxypregnenolone	Antiinflammatory	566-78-9		$C_{23}H_{34}O_4$
Acetryptine	Antihypotensive	3551-18-6		$C_{12}H_{14}N_2O$
Acetyl Strophanthidin	Cardiotonic	60-38-8		$C_{25}H_{34}O_7$
Acetylcholine	Cholinergic	51-84-3		$C_7H_{16}NO_2+$
Acetylcysteine	Expectorant	616-91-1	Y	$C_5H_9NO_3S$
Acetyldigitoxin	Cardiotonic	1111-39-3	Y	$C_{43}H_{66}O_{14}$
Acetyl-L-Leucine	Antiemetic	1188-21-2		$C_8H_{15}NO_3$
Acexamic Acid	Antiinflammatory	57-08-9		$C_8H_{15}NO_3$
Acifran	Antihyperlipidemic	72420-38-3		$C_{12}H_{10}O_4$
Acipimox	Antihyperlipidemic	51037-30-0	Y	$C_6H_6N_2O_3$

Table S1. *Cont.*

Common Name	Indication	CAS Number	Oral	Molecular Formula
Acitazanolast	Bronchodilator	114607-46-4		$C_9H_7N_5O_3$
Acitemate	Antineoplastic	101197-99-3		$C_{14}H_{18}N_2O_5$
Acitretin	Dermatologic	55079-83-9	Y	$C_{21}H_{26}O_3$
Acivicin	Antineoplastic	42228-92-2		$C_5H_7ClN_2O_3$
Aclantate	Antiinflammatory	39633-62-0		$C_{15}H_{14}ClNO_4S$
Aclarubicin	Antineoplastic	57576-44-0		$C_{42}H_{53}NO_{15}$
Aclatonium Napadisilate	Cholinergic	55077-30-0		$C_{10}H_{20}NO_4.1/2C_{10}H_6O_6S_2$
Acodazole	Antineoplastic	79152-85-5		$C_{20}H_{19}N_5O$
Acolbifene	Antineoplastic	182167-02-8		$C_{29}H_{31}NO_4$
Aconiazide	Antibiotic	13410-86-1		$C_{15}H_{13}N_3O_4$
Aconitine	Analgesic	302-27-2		$C_{34}H_{47}NO_{11}$
Acotiamide	Gastroprokinetic	185106-16-5	Y	$C_{21}H_{30}N_4O_5S$
Acoxatrine	Vasodilator	748-44-7		$C_{23}H_{28}N_2O_3$
Acreozast	Bronchodilator	123548-56-1		$C_{15}H_{14}ClN_3O_6$
Acridorex	Gastroprokinetic	47487-22-9		$C_{24}H_{24}N_2$
Acrihellin	Cardiotonic	67696-82-6		$C_{29}H_{38}O_7$
Acrisorcin	Antifungal	90-45-9		$C_{13}H_{10}N_2$
Acrivastine	Antihistaminic	87848-99-5	Y	$C_{22}H_{24}N_2O_2$
Acrocinonide	Steroid	28971-58-6		$C_{24}H_{29}FO_6$
Acronine	Antineoplastic	7008-42-6		$C_{20}H_{19}NO_3$
Actarit	Antirheumatic	18699-02-0	Y	$C_{10}H_{11}NO_3$
Actinoquinol Sodium	Dermatologic	15301-40-3		$C_{11}H_{11}NO_4S$
Actisomide	Antiarrhythmic	96914-39-5		$C_{23}H_{35}N_3O$
Actodigin	Cardiotonic	36983-69-4		$C_{29}H_{44}O_9$
Acyclovir	Antiviral	59277-89-3	Y	$C_8H_{11}N_5O_3$
Adafenoxate	Antidepressant	82168-26-1		$C_{20}H_{26}ClNO_3$
Adamexine	Mucolytic	54785-02-3		$C_{20}H_{26}Br_2N_2O$
Adapalene	Dermatologic	106685-40-9		$C_{28}H_{28}O_3$
Adaprolol Maleate	Antihypertensive	101479-70-3		$C_{26}H_{39}NO_4$
Adatanserin	Antidepressant	127266-56-2		$C_{21}H_{31}N_5O$
Adefovir	Antiviral	106941-25-7		$C_8H_{12}N_5O_4P$
Adefovir Dipivoxil	Antiviral	142340-99-6	Y	$C_{20}H_{32}N_5O_8P$
Adekalant	Cardiotonic	227940-00-3		$C_{22}H_{31}N_3O_4$
Adelmidrol	Oxytocic	1675-66-7		$C_{13}H_{26}N_2O_4$
Ademetionine	Hepatic Protectant	29908-03-0	Y	$C_{15}H_{22}N_6O_5S$
Adenine	Antineoplastic	73-24-5		$C_5H_5N_5$
Adenosine	Antiarrhythmic	58-61-7		$C_{10}H_{13}N_5O_4$
Adibendan	Cardiotonic	100510-33-6		$C_{16}H_{14}N_4O$
Adicillin	Antibiotic	525-94-0		$C_{14}H_{21}N_3O_6S$
Adimolol	Antihypertensive	78459-19-5		$C_{25}H_{29}N_3O_3$
Adinazolam	Antidepressant	37115-32-5		$C_{19}H_{18}ClN_5$
Adiphenine	Antispasmodic	64-95-9		$C_{20}H_{25}NO_2$
Adosopine	Urologic	88124-26-9		$C_{17}H_{14}N_2O_3$
Adozelesin	Antineoplastic	110314-48-2		$C_{30}H_{22}N_4O_4$
Adrafinil	Antidepressant	63547-13-7	Y	$C_{15}H_{15}NO_3S$
Adrenalone	Hemostatic	99-45-6		$C_9H_{11}NO_3$
Adrenochrome Monoaminoguanidine Mesilate	Hemostatic	1214-74-0		$C_{10}H_{13}N_5O_2$
Adrogolide	Antiparkinsonian	171752-56-0		$C_{22}H_{25}NO_4S$
Afeletecan	Antineoplastic	215604-75-4		$C_{45}H_{49}N_7O_{11}S$
Afloqualone	Muscle Relaxant	56287-74-2		$C_{16}H_{14}FN_3O$
Afurolol	Antihypertensive	65776-67-2		$C_{15}H_{21}NO_4$
Aganodine	Cardiotonic	86696-87-9		$C_9H_{10}Cl_2N_4$
Aglepristone	Progestogen	124478-60-0		$C_{29}H_{37}NO_2$
Agomelatine	Antidepressant	138112-76-2	Y	$C_{15}H_{17}NO_2$
Ajmaline	Antihypertensive	4360-12-7		$C_{20}H_{26}N_2O_2$
Aklomide	Antibacterial	3011-89-0		$C_7H_5ClN_2O_3$
Alacepril	Antihypertensive	74258-86-9	Y	$C_{20}H_{26}N_2O_5S$
Alafosfalin	Bone Resorption Inhibitor	60668-24-8		$C_5H_{13}N_2O_4P$
Alagebrium Chloride	Antidiabetic	341028-37-3		$C_{13}H_{14}ClNOS$
Alamifovir	Antiviral	193681-12-8		$C_{19}H_{20}F_6N_5O_5PS$
Alaproclate	Antidepressant	60719-82-6		$C_{13}H_{18}ClNO_2$
Alatrofloxacin	Antibacterial	146961-76-4		$C_{26}H_{25}F_3N_6O_5$

Table S1. *Cont.*

Common Name	Indication	CAS Number	Oral	Molecular Formula
Alazanine Triclofenate	Antiinflammatory	7775-97-5		$C_{21}H_{21}N_2S_2.C_6H_2Cl_3O$
Albaconazole	Antifungal	187949-02-6		$C_{20}H_{16}ClF_2N_5O_2$
Albendazole	Anthelminithic	54965-21-8	Y	$C_{12}H_{15}N_3O_2S$
Albendazole Oxide	Anthelminithic	54029-12-8		$C_{12}H_{15}N_3O_3S$
Albuterol	Bronchodilator	18559-94-9		$C_{13}H_{21}NO_3$
Albutoin	Anticonvulsant	830-89-7		$C_{10}H_{16}N_2OS$
Alclofenac	Analgesic	22131-79-9		$C_{11}H_{11}ClO_3$
Alclometasone Dipropionate	Antiinflammatory	66734-13-2		$C_{28}H_{37}ClO_7$
Alcohol	Antidote	64-17-5		C_2H_6O
Alcuronium Chloride	Neuromuscular Blocking Agent	15180-03-7		$C_{44}H_{50}N_4O_2.2Cl$
Aldioxa	Antiulcerative	5579-81-7		$C_4H_7AlN_4O_5$
Alemcinal	Gastroprokinetic	150785-53-8		$C_{38}H_{67}NO_{10}$
Alendronic Acid	Bone Resorption Inhibitor	66376-36-1	Y	$C_4H_{13}NO_7P_2$
Alentemol Hydrobromide	Antipsychotic	112891-97-1		$C_{19}H_{25}NO$
Alepride	Gastroprokinetic	66564-15-6		$C_{22}H_{30}ClN_3O_2$
Alestramustine	Antineoplastic	139402-18-9		$C_{26}H_{36}Cl_2N_2O_4$
Aletamine	Antidepressant	4255-23-6		$C_{11}H_{15}N$
Alexidine	Antibacterial	22573-93-9		$C_{26}H_{56}N_{10}$
Alfadex	Erectile Dysfunction	10016-20-3	Y	$C_{36}H_{60}O_{30}$
Alfadolone	Anesthetic	14107-37-0		$C_{21}H_{32}O_4$
Alfaprostol	Prostaglandin	74176-31-1		$C_{24}H_{38}O_5$
Alfatradiol	Estrogen	57-91-0		$C_{18}H_{24}O_2$
Alfaxalone	Anesthetic	23930-19-0		$C_{21}H_{32}O_3$
Alfentanil	Analgesic	71195-58-9		$C_{21}H_{32}N_6O_3$
Alfuzosin	Antihypertensive	81403-80-7		$C_{19}H_{27}N_5O_4$
Algestone Acetonide	Antiinflammatory	4968-09-6		$C_{24}H_{34}O_4$
Algestone Acetophenide	Dermatologic	24356-94-3		$C_{29}H_{36}O_4$
Alibendol	Choleretic	26750-81-2		$C_{13}H_{17}NO_4$
Aliconazole	Antifungal	63824-12-4		$C_{18}H_{13}Cl_3N_2$
Alifedrine	Cardiotonic	78756-61-3		$C_{18}H_{27}NO_2$
Aliflurane	Anesthetic	56689-41-9		$C_4H_3ClF_4O$
Alimadol	Nootropic	52742-40-2		$C_{19}H_{23}NO$
Alinastine	Antihistaminic	154541-72-7	Y	$C_{28}H_{39}N_3O$
Alinidine	Antiarrhythmic	33178-86-8		$C_{12}H_{13}Cl_2N_3$
Alipamide	Diuretic	3184-59-6		$C_9H_{12}ClN_3O_3S$
Aliskiren	Antihypertensive	173334-57-1	Y	$C_{30}H_{53}N_3O_6$
Alitretinoin	Antineoplastic	5300-03-8		$C_{20}H_{28}O_2$
Alizapride	Antiemetic	59338-93-1	Y	$C_{16}H_{21}N_5O_2$
Alkofanone	Antidiarrheal	7527-94-8		$C_{21}H_{19}NO_3S$
Allantoin	Dermatologic	97-59-6		$C_4H_6N_4O_3$
Alletorphine	Analgesic	23758-80-7		$C_{27}H_{35}NO_4$
Allobarbital	Sedative	52-43-7		$C_{10}H_{12}N_2O_3$
Alloclamide	Antitussive	5486-77-1		$C_{16}H_{23}ClN_2O_2$
Allocupreide Sodium	Antirheumatic	500-48-1		$C_{11}H_{12}N_2O_2S$
Allomethadione	Anticonvulsant	526-35-2		$C_7H_9NO_3$
Allopurinol	Antiurolithic	315-30-0	Y	$C_5H_4N_4O$
Allyl Isothiocyanate	Dermatologic	57-06-7		C_4H_5NS
Allylestrenol	Progestogen	432-60-0		$C_{21}H_{32}O$
Allylprodine	Analgesic	25384-17-2		$C_{18}H_{25}NO_2$
Almecillin	Antibiotic	87-09-2		$C_{13}H_{18}N_2O_4S_2$
Almestrone	Steroid	10448-96-1		$C_{19}H_{24}O_2$
Alminoprofen	Antiinflammatory	39718-89-3		$C_{13}H_{17}NO_2$
Almitrine	Respiratory Stimulant	27469-53-0		$C_{26}H_{29}F_2N_7$
Almokalant	Antiarrhythmic	123955-10-2		$C_{18}H_{28}N_2O_3S$
Almotriptan	Antimigraine	154323-57-6	Y	$C_{17}H_{25}N_3O_2S$
Almoxatone	Antidepressant	84145-89-1		$C_{18}H_{19}ClN_2O_3$
Almurtide	Immunomodulator	61136-12-7		$C_{18}H_{30}N_4O_{11}$
Alnespirone	Antidepressant	138298-79-0		$C_{26}H_{38}N_2O_4$
Alniditan Di	Antimigraine	152317-89-0		$C_{17}H_{26}N_4O$
Alonacic	Opthalmic	105292-70-4		$C_9H_{16}N_2O_3S$
Alonimid	Sedative	2897-83-8		$C_{14}H_{13}NO_3$
Aloracetam	Nootropic	119610-26-3		$C_{11}H_{16}N_2O_2$

Table S1. *Cont.*

Common Name	Indication	CAS Number	Oral	Molecular Formula
Alosetron	Antidiarrheal	122852-42-0	Y	$C_{17}H_{18}N_4O$
Alovudine	Antiviral	25526-93-6		$C_{10}H_{13}F_2N_2O_4$
Aloxistatin	Immunomodulator	88321-09-9		$C_{17}H_{30}N_2O_5$
Alozafone	Anticonvulsant	65899-72-1		$C_{21}H_{21ClFN3O2}$
Alpertine	Antipsychotic	27076-46-6		$C_{25}H_{31}$N3O4
Alphacetylmethadol	Analgesic	17199-58-5		$C_{23}H_{31}NO_2$
Alpha Cypermethrin	Ectoparasiticide	67375-30-8		$C_{22}H_{19}Cl_2NO_3$
Alphameprodine	Analgesic	468-51-9		$C_{17}H_{25}NO_2$
Alphamethadol	Analgesic	17199-54-1		$C_{21}H_{29}NO$
Alphaprodine	Analgesic	77-20-3		$C_{16}H_{23}NO_2$
Alpidem	Anxiolytic	82626-01-5		$C_{21}H_{23}Cl_2N_3O$
Alpiropride	Antimigraine	81982-32-3		$C_{17}H_{26}N_4O_4S$
Alprafenone	Antiarrhythmic	124316-02-5		$C_{25}H_{35}NO_4$
Alprazolam	Anxiolytic	28981-97-7	Y	$C_{17}H_{13}ClN_4$
Alprenolol	Antihypertensive	13655-52-2	Y	$C_{15}H_{23}NO_2$
Alprenoxime	Antiglaucoma	118552-63-9		$C_{15}H_{22}N_2O_2$
Alprostadil	Vasodilator	745-65-3		$C_{20}H_{34}O_5$
Alrestatin	Antidiabetic	51411-04-2		$C_{14}H_9NO_4$
Altanserin Tartrate	Antidepressant	76330-71-7		$C_{22}H_{22}FN_3O_2S$
Altapizone	Vasodilator	93277-96-4		$C_{24}H_{28}N_4O_2$
Alteconazole	Antifungal	93479-96-0		$C_{17}H_{12}Cl_3N_3O$
Althiazide	Diuretic	5588-16-9		$C_{11}H_{14}ClN_3O_4S_3$
Altinicline	Nootropic	179120-92-4		$C_{12}H_{14}N_2$
Altoqualine	Antibiotic	121029-11-6		$C_{27}H_{36}N_2O_8$
Altrenogest	Progestogen	850-52-2		$C_{21}H_{26}O_2$
Altretamine	Antineoplastic	645-05-6		$C_9H_{18}N_6$
Alvameline Maleate	Antipsychotic	120241-31-8		$C_9H_{15}N_5$
Alverine	Antispasmodic	150-59-4		$C_{20}H_{27}N$
Alvimopan	Antiemetic	156053-89-3	Y	$C_{25}H_{32}N_2O_4$
Alvocidib	Antineoplastic	146426-40-6		$C_{21}H_{20}ClNO_5$
Amadinone Acetate	Progestogen	22304-34-3		$C_{22}H_{27}ClO_4$
Amafolone	Steroid	50588-47-1		$C_{19}H_{31}NO_2$
Amanozine	Diuretic	537-17-7		$C_9H_9N_5$
Amantadine	Antiviral	768-94-5	Y	$C_{10}H_{17}N$
Amantocillin	Antibiotic	10004-67-8		$C_{19}H_{27}N_3O_4S$
Ambamustine	Antineoplastic	85754-59-2		$C_{29}H_{39}Cl_2FN_4O_4S$
Ambasilide	Antiarrhythmic	83991-25-7		$C_{21}H_{25}N_3O$
Ambazone	Antibacterial	539-21-9		$C_8H_{11}N_7S$
Ambenonium	Neuromuscular Blocking Agent	7648-98-8	Y	$C_{28}H_{42}Cl_2N_4O_2$
Ambenoxan	Anxiolytic	2455-84-7		$C_{14}H_{21}NO_4$
Ambrisentan	Anticoagulant	177036-94-1	Y	$C_{22}H_{22}N_2O_4$
Ambroxol	Expectorant	18683-91-5	Y	$C_{13}H_{18}Br_2N_2O$
Ambruticin	Antifungal	58857-02-6		$C_{28}H_{42}O_6$
Ambucaine	Anesthetic	119-29-9		$C_{17}H_{28}N_2O_3$
Ambucetamide	Antispasmodic	519-88-0		$C_{17}H_{28}N_2O_2$
Ambuphylline	Bronchodilator	124-68-5		$C_4H_{11}NO$
Ambuside	Diuretic	3754-19-6		$C_{13}H_{16}ClN_3O_5S_2$
Ambutonium Bromide	Antispasmodic	115-51-5		$C_{20}H_{27}BrN_2O$
Amcinafal	Antiinflammatory	3924-70-7		$C_{26H35FO6}$
Amcinafide	Antiinflammatory	7332-27-6		$C_{29}H_{33}FO_6$
Amcinonide	Glucocorticoid	51022-69-6		$C_{28}H_{35}FO_7$
Amdinocillin	Antibiotic	32887-01-7		$C_{15}H_{23}N_3O_3S$
Amdoxovir	Antiviral	145514-04-1		$C_9H_{12}N_6O_3$
Amebucort	Glucocorticoid	83625-35-8		$C_{28}H_{40}O_7$
Amedalin	Antidepressant	22136-26-1		$C_{19}H_{22}N_2O$
Amelometasone	Steroid	123013-22-9		$C_{26H35FO6}$
Ameltolide	Anticonvulsant	787-93-9		$C_{15}H_{16}N_2O$
Amelubant	Antirheumatic	346735-24-8		$C_{33}H_{34}N_2O_5$
Amesergide	Antidepressant	121588-75-8		$C_{25}H_{35}N_3O$
Ametantrone Acetate	Antineoplastic	64862-96-0		$C_{22}H_{28}N_4O_4$
Amezepine	Analgesic	60575-32-8		$C_{18}H_{20}N_2$
Amezinium Metilsulfate	Antihypotensive	41658-78-0		$C_{11}H_{12}N_3O$

Table S1. *Cont.*

Common Name	Indication	CAS Number	Oral	Molecular Formula
Amfenac	Antiinflammatory	51579-82-9		$C_{15}H_{13}NO_3$
Amfepentorex	Anorexic	15686-27-8		$C_{15}H_{25}N$
Amfetaminil	Nootropic	17590-01-1		$C_{17}H_{18}N_2$
Amflutizole	Antiurolithic	82114-19-0		$C_{11}H_7F_3N_2O_2S$
Amfonelic Acid	Nootropic	15180-02-6		$C_{18}H_{16}N_2O_3$
Amicarbalide	Antiprotozoal	3459-96-9		$C_{15}H_{16}N_6O$
Amicibone	Antitussive	23271-63-8		$C_{22}H_{31}NO_3$
Amicycline	Antibiotic	5874-95-3		$C_{21}H_{23}N_3O_7$
Amidantel	Anthelminithic	49745-00-8		$C_{13}H_{19}N_3O_2$
Amidapsone	Antiviral	3569-77-5		$C_{13}H_{13}N_3O_3S$
Amidephrine Mesylate	Decongestant	37571-84-9		$C_{10}H_{16}N_2O_3S$
Amiflamine	Antidepressant	77518-07-1		$C_{12}H_{20}N_2$
Amifloverine	Antispasmodic	54063-24-0		$C_{16}H_{27}NO_3$
Amifloxacin	Antibiotic	86393-37-5		$C_{16}H_{19}FN_4O_3$
Amifostine	Antineoplastic	20537-88-6		C_5H_{15}N2O3PS
Amiglumide	Gastroprokinetic	119363-62-1		$C_{26}H_{36}$N2O4
Amikacin	Antibiotic	37517-28-5		$C_{22}H_{43}$N5O13
Amikhelline	Antispasmodic	4439-67-2		$C_{18}H_{21}NO_5$
Amiloride	Diuretic	2609-46-3	Y	$C_6H_8ClN_7O$
Amiloxate	Dermatologic	71617-10-2		$C_{15}H_{20}O_3$
Amindocate	Estrogen	31386-24-0		$C_{19}H_{29}N_3O_2$
Amineptine	Nootropic	57574-09-1	Y	$C_{22}H_{27}NO_2$
Aminobenzoic Acid	Dermatologic	1321-11-5		$C_7H_7NO_2$
Aminocaproic Acid	Hemostatic	1319-82-0	Y	$C_6H_{13}NO_2$
Aminoethyl Nitrate	Unclassified	646-02-6		$C_2H_6N_2O_3$
Aminoglutethimide	Antineoplastic	125-84-8	Y	$C_{13}H_{16}N_2O_2$
Aminolevulinic Acid	Antineoplastic	106-60-5	Y	$C_5H_9NO_3$
Aminometradine	Diuretic	642-44-4		$C_9H_{13}N_3O_2$
Aminopentamide Sulfate	Antispasmodic	60-46-8		$C_{19}H_{24}N_2O$
Aminophenazone Cyclamate	Analgesic	100-88-9		$C_6H_{13}NO_3S$
Aminopromazine	Antispasmodic	58-37-7		$C_{19}H_{25}N_3S$
Aminopterin	Antibacterial	54-62-6		$C_{19}H_{20}N_8O_5$
Aminopyrine	Antiinflammatory	58-15-1		$C_{13}H_{17}N_3O$
Aminorex	Anorexic	2207-50-3		$C_9H_{10}N_2O$
Aminosalicylic Acid	Antibacterial	28088-64-4	Y	$C_7H_7NO_3$
Amiodarone	Antiarrhythmic	1951-25-3	Y	$C_{25}H_{29}I_2NO_3$
Amiperone	Sedative	1580-71-8		C24H28ClFN2O2
Amiphenazole	Nootropic	490-55-1		$C_9H_9N_3S$
Amipizone	Bronchodilator	69635-63-8		$C_{14}H_{16}ClN_3O_2$
Amiprilose	Immunosuppressant	56824-20-5		$C_{14}H_{27}NO_6$
Amiquinsin	Antihypertensive	13425-92-8		$C_{11}H_{12}N_2O_2$
Amisometradine	Diuretic	550-28-7		$C_9H_{13}N_3O_2$
Amisulpride	Antipsychotic	71675-85-9	Y	$C_{17}H_{27}N_3O_4S$
Amiterol	Bronchodilator	54063-25-1		$C_{12}H_{20}N_2O$
Amithiozone	Antibacterial	104-06-3		$C_{10}H_{12}N_4OS$
Amitivir	Antiviral	111393-84-1		$C_3H_2N_4S$
Amitraz	Ectoparasiticide	33089-61-1		$C_{19}H_{23}N_3$
Amitriptyline	Antidepressant	50-48-6	Y	$C_{20}H_{23}N$
Amitriptylinoxide	Antidepressant	4317-14-0		$C_{20}H_{23}NO$
Amixetrine	Antiinflammatory	24622-72-8		$C_{17}H_{27}NO$
Amlexanox	Antihistaminic	68302-57-8		$C_{16}H_{14}N_2O_4$
Amlodipine	Antianginal	88150-42-9	Y	$C_{20}H_{25}ClN_2O_5$
Ammonium Valerate	Sedative	109-52-4		$C_5H_{10}O_2$
Amobarbital	Sedative	57-43-2		$C_{11}H_{18}N_2O_3$
Amocarzine	Anthelminithic	36590-19-9		$C_{18}H_{21}N_5O_2S$
Amodiaquine	Antimalarial	86-42-0	Y	$C_{20}H_{22}ClN_3O$
Amogastrin	Antispasmodic	16870-37-4		$C_{35}H_{46}N_6O_8S$
Amolanone	Anesthetic	76-65-3		$C_{20}H_{23}NO_2$
Amonafide	Antineoplastic	69408-81-7		$C_{16}H_{17}N_3O_2$
Amoproxan	Antiarrhythmic	22661-76-3		$C_{22}H_{35}NO_7$
Amopyroquine	Antimalarial	550-81-2		$C_{20}H_{20}ClN_3O$
Amorolfine	Antibacterial	78613-35-1		$C_{21}H_{35}NO$
Amoscanate	Anthelminithic	26328-53-0		$C_{13}H_9N_3O_2S$

Table S1. *Cont.*

Common Name	Indication	CAS Number	Oral	Molecular Formula
Amosulalol	Antihypertensive	85320-68-9	Y	$C_{18}H_{24}N_2O_5S$
Amotosalen	Dermatologic	161262-29-9		$C_{17}H_{19}NO_4$
Amotriphene	Vasodilator	5585-64-8		$C_{26}H_{29}NO_3$
Amoxapine	Antidepressant	14028-44-5	Y	$C_{17}H_{16}ClN_3O$
Amoxecaine	Anesthetic	553-65-1		$C_{17}H_{29}N_3O_2$
Amoxicillin	Antibiotic	26787-78-0	Y	$C_{16}H_{19}N_3O_5S$
Amoxydramine Camsilate	Antihypertensive	3922-74-5		$C_{17}H_{21}NO_2$
Amoxydramine Camsilate	Antihypertensive	3144-16-9		$C_{10}H_{16}O_4S$
Amperozide	Sedative	75558-90-6		$C_{23}H_{29}F_2N_3O$
Amphecloral	Anorexic	5581-35-1		$C_{11}H_{12}Cl_3N$
Amphenidone	Sedative	134-37-2		$C_{11}H_{10}N_2O$
Amphetamine	Nootropic	300-62-9	Y	$C_9H_{13}N$
Amphotalide	Antithrombotic	1673-06-9		$C_{19}H_{20}N_2O_3$
Ampicillin	Antibiotic	69-53-4	Y	$C_{16}H_{19}N_3O_4S$
Ampiroxicam	Antiinflammatory	99464-64-9		$C_{20}H_{21}N_3O_7S$
Amprenavir	Antiviral	161814-49-9	Y	$C_{25}H_{35}N_3O_6S$
Amprolium	Antibacterial	121-25-5		$C_{14}H_{19}N_4.Cl$
Amprotropine Phosphate	Antihypertensive	148-32-3		$C_{18}H_{29}NO_3$
Ampyrimine	Antineoplastic	5587-93-9		$C_{12}H_{11}N_7$
Ampyzine Sulfate	Nootropic	5214-29-9		$C_6H_9N_3$
Amquinate	Antimalarial	17230-85-2		$C_{18}H_{24}N_2O_3$
Amrinone	Cardiotonic	60719-84-8		$C_{10}H_9N_3O$
Amrubicin	Antineoplastic	110267-81-7		$C_{25}H_{25}NO_9$
Amsacrine	Antineoplastic	51264-14-3		$C_{21}H_{19}N_3O_3S$
Amtolmetin Guacil	Analgesic	87344-06-7	Y	$C_{24}H_{24}N_2O5$
Amustaline Dihydrochloride	Antibacterial	878189-87-8		$C_{22}H_{25}Cl_2N_3O_2$
Amylene Hydrate	Sedative	75-85-4		$C_5H_{12}O$
Amylocaine	Anesthetic	644-26-8		$C_{14}H_{21}NO_2$
Anagestone Acetate	Progestogen	3137-73-3		$C_{24}H_{36}O_3$
Anagrelide	Antithrombotic	68475-42-3	Y	$C_{10}H_7Cl_2N_3O$
Anastrozole	Antineoplastic	120511-73-1	Y	$C_{17}H_{19}N_5$
Anatibant	Antirheumatic	209733-45-9		$C_{34}H_{36}Cl_2N_6O_5S$
Anaxirone	Antineoplastic	99212-42-7		$C_{11}H_{15}N_3O_5$
Anazocine	Analgesic	15378-99-1		$C_{16}H_{23}NO$
Ancarolol	Antihypertensive	75748-50-4		$C_{18}H_{24}N_2O_4$
Ancitabine	Antineoplastic	31698-14-3		$C_9H_{11}N_3O_4$
Ancriviroc	Antiviral	370893-06-4		$C_{28}H_{37}BrN_4O_3$
Andolast	Bronchodilator	132640-22-3		$C_{15}H_{11}N_9O$
Androstenediol	Steroid	521-17-5		$C_{19}H_{30}O_2$
Androstenedione	Steroid	63-05-8		$C_{19}H_{26}O_2$
Anecortave Acetate	Opthalmic	7753-60-8		$C_{23}H_{30}O_5$
Anethole	Antitussive	104-46-1		$C_{10}H_{12}O$
Anidoxime	Analgesic	34297-34-2		$C_{21}H_{27}N_3O_3$
Anilamate	Anticonvulsant	5591-49-1		$C_{15}H_{14}N_2O_3$
Anileridine	Analgesic	144-14-9		$C_{22}H_{28}N_2O_2$
Anilopam	Analgesic	53716-46-4		$C_{20}H_{26}N_2O$
Anipamil	Antianginal	83200-10-6		$C_{34}H_{52}N_2O_2$
Aniracetam	Nootropic	72432-10-1	Y	$C_{12}H_{13}NO_3$
Anirolac	Antiinflammatory	66635-85-6		$C_{16}H_{15}NO_4$
Anisacril	Unclassified	5129-14-6		$C_{22}H_{18}O_3$
Anisindione	Anticoagulant	117-37-3	Y	$C_{16}H_{12}O_3$
Anisomycin	Antibiotic	22862-76-6		$C_{14}H_{19}NO_4$
Anisopirol	Unclassified	857-62-5		$C_{21}H_{27}FN_2O_2$
Anisotropine	Antispasmodic	25333-49-7	Y	$C_{16}H_{29}NO_2$
Anisperimus	Immunosuppressant	170368-04-4		$C_{18}H_{39}N_7O_3$
Anitrazafen	Antiinflammatory	63119-27-7		$C_{18}H_{17}N_3O_2$
Anpirtoline	Analgesic	98330-05-3		$C_{10}H_{13}ClN_2S$
Ansoxetine	Antidepressant	79130-64-6		$C_{26}H_{25}NO_3$
Antafenite	Antiprotozoal	6649-73-6		$C_{11}H_{10}N_2S$
Antazoline	Antihistaminic	91-75-8		$C_{17}H_{19}N_3$
Antazonite	Antihistaminic	5028-87-5		$C_{11}H_{12}N_2O_2S_2$
Anthralin	Dermatologic	1143-38-0		$C_{14}H_{10}O_3$
Anthelmycin	Anthelminithic	12706-94-4		$C_{21}H_{37}N_5O_{14}$

Table S1. *Cont.*

Common Name	Indication	CAS Number	Oral	Molecular Formula
Anthiolimine	Diuretic	70-49-5		$C_4H_6O_4S$
Anthracene	Antineoplastic	120-12-7		$C_{14}H_{10}$
Anthramycin	Antineoplastic	4803-27-4		$C_{16}H_{17}N_3O_4$
Antienite	Unclassified	5719-88-0		$C_9H_8N_2S_2$
Antimony Sodium Thioglycollate	Anthelminithic	1843-43-2		$C_4H_5O_4S_2Sb$
Antipyrine	Antiinflammatory	60-80-0		$C_{11}H_{12}N_2O$
Antrafenine	Analgesic	55300-29-3		$C_{30}H_{26}F_6N_4O_2$
Apadoline	Analgesic	135003-30-4		$C_{23}H_{29}N_3OS$
Apafant	Antithrombotic	105219-56-5		$C_{22}H_{22}ClN_5O_2S$
Apalcillin	Antibacterial	63469-19-2		$C_{25}H_{23}N_5O_6S$
Apaxifylline	Bronchodilator	151581-23-6		$C_{16}H_{22}N_4O_3$
Apaziquone	Antineoplastic	114560-48-4		$C_{15}H_{16}N_2O_4$
Apazone	Antiinflammatory	13539-59-8	Y	$C_{16}H_{20}N_4O_2$
Apicycline	Antibiotic	15599-51-6		$C_{30}H38N4O11$
Apixaban	Anticoagulant	503612-47-3	Y	$C_{25}H_{25}N_5O_4$
Aplindore Fumarate	Antipsychotic	189681-70-7		$C_{18}H_{18}N_2O_3$
Apomorphine	Antiparkinsonian	58-00-4		$C_{17}H_{17}NO_2$
Apovincamine	Cardiotonic	4880-92-6		$C_{21}H_{24}N_2O_2$
Apraclonidine	Opthalmic	66711-21-5		$C_9H_{10}Cl_2N_4$
Apramycin	Antibiotic	37321-09-8		$C_{21}H_{41}N_5O_{11}$
Apratastat	Antihyperlipidemic	287405-51-0		$C_{17}H_{22}N_2O_6S_2$
Aprepitant	Antiemetic	170729-80-3	Y	$C_{23}H_{21}F_7N_4O_3$
Aprikalim	Cardiotonic	132562-26-6		$C_{12}H_{16}N_2OS_2$
Aprindine	Antiarrhythmic	37640-71-4	Y	$C_{22}H_{30}N_2$
Aprobarbital	Sedative	77-02-1		$C_{10}H_{14}N_2O_3$
Aprofene	Antihypertensive	3563-01-7		$C_{21}H_{27}NO_2$
Aptazapine Maleate	Antidepressant	71576-40-4		$C_{16}H_{19}N_3$
Aptiganel	Nootropic	137159-92-3		$C_{20}H_{21}N_3$
Aptocaine	Anesthetic	19281-29-9		$C_{14}H_{20}N_2O$
Aranidipine	Antihypertensive	86780-90-7		$C_{19}H_{20}N_2O_7$
Aranotin	Antiviral	19885-51-9		$C_{20}H_{18}N_2O_7S_2$
Araprofen	Antiinflammatory	15250-13-2		$C_{16}H_{15}NO_4$
Arasertaconazole	Antifungal	583057-48-1		$C_{20}H_{15}Cl_3N_2OS$
Arbaprostil	Antiulcerative	55028-70-1		$C_{21}H_{34}O_5$
Arbekacin	Antibiotic	51025-85-5		$C_{22}H_{44}N_6O_{10}$
Arbutamine	Antihypotensive	128470-16-6		$C_{18}H_{23}NO_4$
Arbutin	Dermatologic	497-76-7		$C_{12}H_{16}O7$
Arecoline	Anthelminithic	63-75-2		$C_8H_{13}NO2$
Arfendazam	Anxiolytic	37669-57-1		$C_{18}H_{17}ClN_2O_3$
Arformoterol Tartrate	Bronchodilator	67346-49-0		$C_{19}H_{24}N_2O_4$
Argatroban	Anticoagulant	74863-84-6		$C_{23}H_{36}N_6O_5S$
Argimesna	Antineoplastic	74-79-3		$C_6H_{14}N_4O_2$
Arildone	Antiviral	56219-57-9		$C_{20}H_{29}ClO_4$
Arimoclomol	Capillary Protectant	289893-25-0	Y	$C_{14}H_{20}ClN_3O_3$
Aripiprazole	Antipsychotic	129722-12-9	Y	$C_{23}H_{27}Cl_2N_3O_2$
Armodafinil	Nootropic	112111-43-0	Y	$C_{15}H_{15}NO_2S$
Arnolol	Antihypertensive	87129-71-3		$C_{14}H_{23}NO_3$
Arofylline	Bronchodilator	136145-07-8		$C_{14}H_{13}ClN_4O_2$
Aronixil	Antihypertensive	86627-15-8		$C_{14}H_{15}ClN_4O_2$
Arotinolol	Antianginal	68377-92-4	Y	$C_{15}H_{21}N_3O_2S_3$
Arprinocid	Antibacterial	55779-18-5		$C_{12}H_9ClFN_5$
Arpromidine	Analgesic	106669-71-0		$C_{21}H_{25}FN_6$
Arsanilic Acid	Antibacterial	98-50-0		$C_6H_8AsNO_3$
Arsphenamine	Antibiotic	150-44-7		$C_{12}H_{12}As_2N_2O_2$
Arsthinol	Antiamebic	119-96-0		$C_{11}H_{14}AsNO_3S_2$
Arteflene	Antimalarial	123407-36-3		$C_{19}H_{18}F_6O_3$
Artemether	Antimalarial	71963-77-4	Y	$C_{16}H_{26}O_5$
Artemifone	Antimalarial	255730-18-8		$C_{19}H_{31}NO_6S$
Artemisinin	Antimalarial	63968-64-9		$C_{15}H_{22}O_5$
Artemotil	Antimalarial	75887-54-6		$C_{17}H_{28}O_5$
Artenimol	Antimalarial	71939-50-9	Y	$C_{15}H_{24}O_5$
Artesunate	Antimalarial	88495-63-0	Y	$C_{19}H_{28}O_8$
Articaine	Anesthetic	23964-58-1		$C_{13}H_{20}N_2O_3S$

Table S1. *Cont.*

Common Name	Indication	CAS Number	Oral	Molecular Formula
Artilide Fumarate	Antiarrhythmic	133267-19-3		$C_{19}H_{34}N_2O_3S$
Arundic Acid	Nootropic	185517-21-9		$C_{11}H_{22}O_2$
Arzoxifene	Progestogen	182133-25-1		$C_{28}H_{29}NO_4S$
Asenapine Maleate	Antipsychotic	65576-45-6		$C_{17}H_{16}ClNO$
Aseripide	Antispasmodic	153242-02-5		$C_{26}H_{30}FN_3O_6S$
Asimadoline	Analgesic	153205-46-0	Y	$C_{27}H_{30}N_2O_2$
Asobamast	Antihistaminic	104777-03-9		$C_{13}H_{15}N_3O_5S$
Asocainol	Antiarrhythmic	77400-65-8		$C_{27}H_{31}NO_3$
Asoprisnil	Progestogen	199396-76-4		$C_{28}H_{35}NO_4$
Asoprisnil Ecamate	Progestogen	222732-94-7		$C_{31}H_{40}N_2O_5$
Aspartocin	Antibacterial	4117-65-1		$C_{42}H_{64}N_{12}O_{12}S_2$
Asperlin	Antibacterial	30387-51-0		$C_{10}H_{12}O_5$
Aspirin	Antiinflammatory	50-78-2	Y	$C_9H_8O_4$
Aspoxicillin	Antibiotic	63358-49-6		$C_{21}H_{27}N_5O_7S$
Astemizole	Antihistaminic	68844-77-9		$C_{28}H_{31}FN_4O$
Astromicin Sulfate	Antibacterial	55779-06-1		$C_{17}H_{35}N_5O_6$
Asulacrine	Antineoplastic	80841-47-0		$C_{24}H_{24}N_4O_4S$
Ataciguat	Vasodilator	254877-67-3		$C_{21}H_{19}Cl_2N_3O_6S_3$
Atamestane	Antineoplastic	96301-34-7		$C_{20}H_{26}O_2$
Ataprost	Prostaglandin	83997-19-7		$C_{21}H_{32}O_4$
Ataquimast	Antihistaminic	586348-21-2		$C_{11}H_{13}N_3O$
Atazanavir	Antiviral	198904-31-3	Y	$C_{38}H_{52}N_6O_7$
Atenolol	Antihypertensive	29122-68-7	Y	$C_{14}H_{22}N_2O_3$
Atevirdine Mesylate	Antiviral	136816-75-6		$C_{21}H_{25}N_5O_2$
Atibeprone	Anticonvulsant	153420-96-3		$C_{17}H_{18}N_2O_3S$
Atipamezole	Antihypertensive	104054-27-5		$C_{14}H_{16}N_2$
Atiprimod Dihydrochloride	Antiarrhythmic	123018-47-3		$C_{22}H_{44}N_2$
Atiprosin Maleate	Antihypertensive	89303-63-9		$C_{20}H_{29}N_3$
Atizoram	Dermatologic	135637-46-6		$C_{18}H_{24}N_2O_3$
Atliprofen	Antiinflammatory	108912-14-7		$C_{13}H_{12}O_2S$
Atolide	Anticonvulsant	16231-75-7		$C_{18}H_{23}N_3O$
Atomoxetine	Nootropic	83015-26-3	Y	$C_{17}H_{21}NO$
Atorvastatin	Antihyperlipidemic	134523-00-5	Y	$C_{33}H_{35}FN_2O_5$
Atosiban	Tocolytic	90779-69-4		$C_{43}H_{67}N_{11}O_{12}S_2$
Atovaquone	Antimalarial	95233-18-4	Y	$C_{22}H_{19}ClO_3$
Atracurium	Anticonvulsant	64228-79-1		$C_{53}H_{72}N_2O_{12}2+$
Atrasentan	Anesthetic	173937-91-2	Y	$C_{29}H_{38}N_2O_6$
Atreleuton	Bronchodilator	154355-76-7		$C_{16}H_{15}FN_2O_2S$
Atrimustine	Antineoplastic	75219-46-4		$C_{41}H_{47}Cl_2NO_6$
Atrinositol	Vasodilator	28841-62-5		$C_6H_{15}O_{15}P_3$
Atromepine	Antispasmodic	428-07-9		$C_{18}H_{25}NO_3$
Atropine	Mydriatic	51-55-8		$C_{17}H_{23}NO_3$
Atropine Methylbromide	Antihypotensive	80-49-9	Y	$C_{17}H_{13}BrNO_3$
Atropine Oxide	Antihypotensive	4438-22-6		$C_{17}H_{23}NO_4$
Auranofin	Antirheumatic	34031-32-8	Y	C_{20}H34AuO9PS
Aurothioglucose	Antirheumatic	12192-57-3		$C_6H_{11}AuO_5S$
Aurothioglycanide	Antirheumatic	16925-51-2		C_8H_8AuNOS
Avanafil	Erectile Dysfunction	330784-47-9	Y	$C_{23}H_{26}ClN_7O_3$
Avasimibe	Antihyperlipidemic	166518-60-1		$C_{29}H_{43}NO_4S$
Avitriptan Fumarate	Antimigraine	151140-96-4		$C_{22}H_{30}N_6O_3S$
Avizafone	Anticonvulsant	65617-86-9		$C_{22}H_{27}ClN_4O_3$
Avobenzone	Dermatologic	70356-09-1		$C_{20}H_{22}O_3$
Avosentan	Anesthetic	290815-26-8		$C_{23}H_{21}N_5O_5S$
Avridine	Antiviral	35607-20-6		$C_{43}H_{90}N_2O_2$
Axamozide	Antipsychotic	85076-06-8		$C_{21}H_{22}ClN_3O_3$
Axitinib	Antineoplastic	319460-85-0	Y	$C_{22}H_{18}N_4OS$
Axitirome	Antihyperlipidemic	156740-57-7		$C_{25}H_{24}FNO_6$
Axomadol	Analgesic	187219-99-4		$C_{16}H_{25}NO_3$
Azabon	Nootropic	1150-20-5		$C_{14}H_{20}N_2O_2S$
Azabuperone	Antipsychotic	2856-81-7		$C_{17}H_{23}FN_2O$
Azacitidine	Antineoplastic	320-67-2		$C_8H_{12}N_4O_5$
Azaclorzine	Vasodilator	49864-70-2		$C_{22}H_{24}ClN_3OS$
Azaconazole	Antifungal	60207-31-0		$C_{12}H_{11}Cl_2N_3O_2$

Table S1. *Cont.*

Common Name	Indication	CAS Number	Oral	Molecular Formula
Azacosterol	Antibacterial	313-05-3		$C_{25}H_{44}N_2O$
Azacyclonol	Anxiolytic	115-46-8		$C_{18}H_{21}NO$
Azaftozine	Anxiolytic	54063-26-2		$C_{23}H_{24}F_3N_3OS$
Azalanstat Dihydrochloride	Antihyperlipidemic	143393-27-5		$C_{22}H_{24}ClN_3O_2S$
Azaloxan Fumarate	Antidepressant	72822-56-1		$C_{18}H_{25}N_3O_3$
Azamethiphos	Antineoplastic	35575-96-3		$C_9H_{10}ClN_2O_5PS$
Azamethonium Bromide	Antihypertensive	306-53-6		$C_{13}H_{33}Br_2N_3$
Azamulin	Antibacterial	76530-44-4		$C_{24}H_{38}N_4O_4S$
Azanator Maleate	Bronchodilator	37855-92-8		$C_{18}H_{18}N_2O$
Azanidazole	Antiprotozoal	62973-76-6		$C_{10}H_{10}N_6O_2$
Azaperone	Sedative	1649-18-9		$C_{19}H_{22}FN_3O$
Azapetine Phosphate	Anxiolytic	146-36-1		$C_{17}H_{17}N$
Azaprocin	Antibiotic	448-34-0		$C_{18}H_{24}N_2O$
Azaquinzole	Unclassified	5234-86-6		$C_{12}H_{16}N_2$
Azaribine	Dermatologic	2169-64-4		$C_{14}H_{17}N_3O_9$
Azarole	Immunomodulator	55872-82-7		$C_{14}H_{12}N_4$
Azaserine	Antifungal	115-02-6		$C_5H_7N_3O_4$
Azasetron	Antiemetic	123040-69-7		$C_{17}H_{20}ClN_3O_3$
Azaspirium Chloride	Unclassified	34959-30-3		$C_{22}H_{24}ClNO_5$
Azastene	Immunomodulator	13074-00-5		$C_{23}H_{33}NO_2$
Azatadine	Antihistaminic	3964-81-6	Y	$C_{20}H_{22}N_2$
Azathioprine	Immunosuppressant	446-86-6	Y	$C_9H_7N_7O_2S$
Azelaic Acid	Dermatologic	123-99-9		$C_9H_{16}O_4$
Azelastine	Antihistaminic	58581-89-8		$C_{22}H_{24}ClN_3O$
Azelnidipine	Antihypertensive	123524-52-7	Y	$C_{33}H_{34}N_4O_6$
Azepexole	Antihypertensive	36067-73-9		$C_9H_{15}N_3O$
Azepindole	Antidepressant	26304-61-0		$C_{12}H_{14}N_2$
Azetepa	Antineoplastic	125-45-1		$C_8H_{14}N_5OPS$
Azetirelin	Thyroid	95729-65-0		$C_{15}H_{20}N_6O_4$
Azidamfenicol	Antibiotic	13838-08-9		$C_{11}H_{13}N_5O_5$
Azidocillin	Antibacterial	17243-38-8		$C_{16}H_{17}N_5O_4S$
Azimexon	Immunomodulator	64118-86-1		$C_9H_{14}N_4O$
Azimilide Dihydrochloride	Antiarrhythmic	149908-53-2	Y	$C_{23}H_{28}ClN_5O_3$
Azintamide	Choleretic	1830-32-6		$C_{10}H_{14}ClN_3OS$
Azipramine	Antidepressant	58503-82-5		$C_{26}H_{26}N_2$
Azithromycin	Antibiotic	83905-01-5	Y	$C_{38}H_{72}N_2O_{12}$
Azlocillin	Antibiotic	37091-66-0		$C_{20}H_{23}N_5O_6S$
Azolimine	Diuretic	40828-45-3		$C_{10}H_{11}N_3O$
Azosemide	Diuretic	27589-33-9	Y	$C_{12}H_{11}ClN_6O_2S_2$
Azosulfamide	Diuretic	132-38-7		$C_{18}H_{16}N_4O_{10}S_3$
Azotomycin	Antineoplastic	7644-67-9		$C_{17}H_{23}N_7O_8$
Aztreonam	Antibiotic	78110-38-0		$C_{13}H_{17}N_5O_8S_2$
Azumolene Sodium	Muscle Relaxant	64748-79-4		$C_{13}H_9BrN_4O_3$
Bacampicillin	Antibiotic	50972-17-3	Y	$C_{21}H_{27}N_3O_7S$
Baclofen	Muscle Relaxant	1134-47-0	Y	$C_{10}H_{12}ClNO_2$
Bacmecillinam	Antibacterial	50846-45-2		$C_{20}H_{31}N_3O_6S$
Bakeprofen	Antiinflammatory	74168-02-8		$C_{16}H_{14}O_4$
Balaglitazone	Antidiabetic	199113-98-9		$C_{20}H_{17}N_3O_4S$
Balazipone	Antiinflammatory	137109-71-8		$C_{13}H_{11}NO_2$
Balicatib	Unclassified	354813-19-7		$C_{23}H_{33}N_5O_2$
Balofloxacin	Antibacterial	127294-70-6		$C_{20}H_{24}FN_3O_4$
Balsalazide	Antiinflammatory	80573-04-2	Y	$C_{17}H_{15}N_3O_6$
Bamaluzole	Nootropic	87034-87-5		$C_{14}H_{12}ClN_3O$
Bamaquimast	Antihistaminic	135779-82-7		$C_{16}H21N3O3$
Bambuterol	Bronchodilator	81732-65-2	Y	$C_{18}H_{29}N_3O5$
Bamethan	Vasodilator	3703-79-5		$C_{12}H_{19}NO_2$
Bamifylline	Bronchodilator	2016-63-9		$C_{20}H_{27}N_5O_3$
Bamipine	Antihistaminic	4945-47-5		$C_{19}H_{24}N_2$
Bamirastine	Antitussive	215529-47-8		$C_{31}H_{37}N_5O_3$
Bamnidazole	Antiprotozoal	31478-45-2		$C_7H_{10}N_4O_4$
Baquiloprim	Antibacterial	102280-35-3		$C_{17}H_{20}N_6$
Barbital	Sedative	57-44-3	Y	$C_8H_{12}N_2O_3$
Barixibat	Unclassified	263562-28-3		$C_{42}H_{55}N_5O_8$

Table S1. *Cont.*

Common Name	Indication	CAS Number	Oral	Molecular Formula
Barmastine	Antihistaminic	99156-66-8		$C_{27}H_{29}N_7O_2$
Barnidipine	Antihypertensive	104713-75-9	Y	$C_{27}H_{29}N_3O_6$
Barucainide	Antiarrhythmic	79784-22-8		$C_{22}H_{30}N_2O_2$
Barusiban	Tocolytic	285571-64-4		$C_{40}H_{63}N_9O_8S$
Batabulin Sodium	Antineoplastic	195533-53-0		$C_{13}H_7F_6NO_3S$
Batanopride	Antiemetic	102670-46-2		$C_{17}H_{26}ClN_3O_3$
Batebulast	Anticoagulant	81907-78-0		$C_{19}H_{29}N_3O_2$
Batelapine Maleate	Antipsychotic	95634-82-5		$C_{16}H_{20}N_6$
Batimastat	Antineoplastic	130370-60-4		$C_{23}H_{31}N_3O_4S_2$
Batoprazine	Antipsychotic	105685-11-8		$C_{13}H_{14}N_2O_2$
Baxitozine	Anxiolytic	84386-11-8		$C_{13}H_{14}O_6$
Bazedoxifene Acetate	Bone Resorption Inhibitor	198481-32-2	Y	$C_{30}H_{34}N_2O_3$
Bazinaprine	Antidepressant	94011-82-2		$C_{17}H_{19}N_5O$
Becampanel	Antiseizure	188696-80-2		$C_{10}H_{11}N_4O_7P$
Becanthone	Anthelminithic	15351-04-9		$C_{22}H_{28}N_2O_2S$
Becatecarin	Antineoplastic	119673-08-4		$C_{33}H_{34}Cl_2N_4O_7$
Beciparcil	Antithrombotic	130782-54-6		$C_{12}H_{13}NO_3S_2$
Beclamide	Anticonvulsant	501-68-8		$C_{10}H_{12}ClNO$
Becliconazole	Antifungal	112893-26-2		$C_{18}H_{12}Cl_2N_2O$
Beclobrate	Antihyperlipidemic	55937-99-0		$C_{20}H_{23}ClO_3$
Beclomethasone Dipropionate	Glucocorticoid	5534-09-8		$C_{28}H_{37}ClO_7$
Befetupitant	Antidepressant	290296-68-3		$C_{29}H_{29}F_6N_3O_2$
Befiperide	Antispasmodic	100927-14-8		$C_{25}H_{31}N_3O_2$
Befloxatone	Antidepressant	134564-82-2		$C_{15}H_{18}$F3NO5
Befunolol	Antiglaucoma	39552-01-7		$C_{16}H_{21}NO_4$
Befuraline	Antidepressant	41717-30-0		$C_{20}H_{20}N_2O_2$
Bekanamycin	Antibiotic	4696-76-8		$C_{18}H_{37}N_5O_{10}$
Belaperidone	Antipsychotic	208661-17-0		$C_{22}H_{22}FN_3O_2$
Belarizine	Antihistaminic	52395-99-0		$C_{24}H_{26}N_2O$
Belfosdil	Antihypertensive	103486-79-9		$C_{27}H_{50}O_7P_2$
Belotecan	Antineoplastic	256411-32-2		$C_{25}H_{27}N_3O_4$
Beloxamide	Antihyperlipidemic	15256-58-3		$C_{18}H_{21}NO_2$
Beloxepin	Antidepressant	135928-30-2		$C_{19}H_{21}NO_2$
Bemarinone	Cardiotonic	92210-43-0		$C_{11}H_{12}N_2O_3$
Bemegride	Nootropic	64-65-3		C_8H_{13NO2}
Bemesetron	Antiemetic	40796-97-2		$C_{15}H_{17}Cl_2NO_2$
Bemetizide	Antidiabetic	1824-52-8		$C_{15}H_{16}ClN_3O_4S_2$
Beminafil	Antihypertensive	566906-50-1		$C_{25}H_{24}ClN_3O_3S$
Bemitradine	Antihypertensive	88133-11-3		$C_{15}H_{17}N_5O$
Bemoradan	Cardiotonic	112018-01-6		$C_{13}H_{13}N_3O_3$
Bemotrizinol	Dermatologic	187393-00-6		$C_{38}H_{49}N_3O_5$
Benactyzine	Antispasmodic	302-40-9		$C_{20}H_{25}NO_3$
Benafentrine	Cardiotonic	35135-01-4		$C_{23}H_{27}N_3O_3$
Benapryzine	Antihypertensive	22487-42-9		$C_{21}H_{27}NO_3$
Benaxibine	Antineoplastic	27661-27-4		$C_{12}H_{15}NO_6$
Benazepril	Antihypertensive	86541-75-5	Y	$C_{24}H_{28}N_2O_5$
Benazeprilat	Antihypertensive	86541-78-8		$C_{22}H_{24}N_2O_5$
Bencianol	Antispasmodic	85443-48-7		$C_{28}H_{22}O_6$
Bencisteine	Unclassified	42293-72-1		$C_{15}H_{19}NO_4S$
Benclonidine	Antihypertensive	57647-79-7		$C_{16}H_{13}Cl_2N_3O$
Bencyclane	Vasodilator	2179-37-5		$C_{19}H_{31}NO$
Bendacalol Mesylate	Antihypertensive	81703-42-6		$C_{20}H_{23}NO_6$
Bendamustine	Antineoplastic	16506-27-7		$C_{16}H_{21}Cl_2N_3O_2$
Bendazac	Antiinflammatory	20187-55-7		$C_{16}H_{14}N_2O_3$
Bendazol	Vasodilator	621-72-7	Y	$C_{14}H_{12}N_2$
Benderizine	Vasodilator	59752-23-7		$C_{28}H_{34}N_2O_2$
Bendroflumethiazide	Diuretic	73-48-3	Y	$C_{15}H_{14}F_3N_3O_4S_2$
Benethamine Penicillin	Antibacterial	3647-71-0		$C_{15}H_{17}N$
Benexate	Antiulcerative	78718-52-2		$C_{23}H_{27}N_3O_4$
Benfluorex	Antihyperlipidemic	23602-78-0		$C_{19}H_{20}F_3NO_2$
Benfosformin	Antidiabetic	738523-03-0		$C_9H_{14}N_5O_3P$
Benfurodil Hemisuccinate	Cardiotonic	3447-95-8		$C_{19}H_{18}O_7$
Benhepazone	Unclassified	363-13-3		$C_{15}H_{12}N_2O$

Table S1. *Cont.*

Common Name	Indication	CAS Number	Oral	Molecular Formula
Benidipine	Antihypertensive	105979-17-7	Y	$C_{28}H31N3O6$
Benmoxin	Antidepressant	7654-03-7		$C_{15}H_{16}N_2O$
Benolizime	Antibiotic	61864-30-0		$C_{19}H_{26}N_2O_3$
Benorilate	Analgesic	5003-48-5		$C_{17}H_{15}NO_5$
Benorterone	Steroid	3570-10-3		$C_{19}H_{28}O_2$
Benoxafos	Antineoplastic	16759-59-4		$C_{12}H_{14}Cl_2NO_3PS_2$
Benoxaprofen	Antiinflammatory	51234-28-7		$C_{16}H_{12}ClNO_3$
Benoxinate	Anesthetic	99-43-4		$C_{17}H_{28}N_2O_3$
Benperidol	Antipsychotic	2062-84-2		$C_{22}H_{24}FN_3O_2$
Benproperine	Antitussive	2156-27-6		$C_{21}H_{27}NO$
Benrixate	Unclassified	24671-26-9		$C_{19}H_{30}N_2O_2$
Benserazide	Antiparkinsonian	322-35-0		$C_{10}H_{15N3O5}$
Bensulide	Antiinflammatory	741-58-2		$C_{14}H_{24}NO_4PS_3$
Bentazepam	Sedative	29462-18-8		$C_{17}H_{16}N_2OS$
Bentemazole	Antifungal	63927-95-7		$C_{11}H_{10}N_6$
Bentipimine	Analgesic	17692-23-8		$C_{27}H_{31}ClN_2S$
Benurestat	Antibacterial	38274-54-3		$C_9H_9ClN_2O_3$
Benzaprinoxide	Antidepressant	52758-02-8		$C_{20}H_{20}ClNO$
Benzarone	Capillary Protectant	1477-19-6		$C_{17}H_{14}O_3$
Benzbromarone	Uricosuric	3562-84-3	Y	$C_{17}H_{12}Br_2O_3$
Benzestrol	Estrogen	85-95-0		$C_{20}H_{26}O_2$
Benzethidine	Antihypertensive	3691-78-9		$C_{23}H_{29}NO_3$
Benzetimide	Antihypertensive	14051-33-3		$C_{23}H_{26}N_2O_2$
Benzilonium Bromide	Antihypertensive	1050-48-2		$C_{22}H_{28}NO_3.Br$
Benzindopyrine	Antipsychotic	16571-59-8		$C_{22}H_{20}N_2$
Benziodarone	Vasodilator	68-90-6		$C_{17}H_{12}I_2O_3$
Benzmalecene	Antihyperlipidemic	148-07-2		$C_{20}H_{19}Cl_2NO_3$
Benznidazole	Antiprotozoal	22994-85-0	Y	$C_{12}H_{12}N_4O_3$
Benzobarbital	Sedative	744-80-9		$C_{19}H_{16}N_2O_4$
Benzocaine	Anesthetic	94-09-7		$C_9H_{11}NO_2$
Benzoclidine	Antihypertensive	16852-81-6		$C_{14}H_{17}NO_2$
Benzoctamine	Muscle Relaxant	17243-39-9		$C_{18}H_{19}N$
Benzodepa	Antineoplastic	1980-45-6		$C_{12}H_{16}N_3O_3P$
Benzomethamine Chloride	Antihypotensive	510-08-7		$C_{22}H_{31}ClN_2O_2$
Benzonatate	Antitussive	104-31-4	Y	$C_{30}H_{53}NO_{11}$
Benzotript	Gastroprokinetic	39544-74-6		$C_{18}H_{15}ClN_2O_3$
Benzoyl Peroxide	Dermatologic	94-36-0		$C_{14}H_{10}O_4$
Benzoylpas Calcium	Antibacterial	13898-58-3		$C_{14}H_{11}NO_4$
Benzphetamine	Anorexic	156-08-1	Y	$C_{17}H_{21}N$
Benzpiperylon	Antiinflammatory	53-89-4		$C_{22}H_{25}N_3O$
Benzpyrinium Bromide	Cholinergic	587-46-2		$C_{15}H_{17}BrN_2O_2$
Benzquercin	Capillary Protectant	13157-90-9		$C_{50}H_{40}O_7$
Benzquinamide	Antipsychotic	63-12-7		$C_{22}H_{32}N_2O_5$
Benzthiazide	Diuretic	91-33-8	Y	$C_{15}H_{14}ClN_3O_4S_3$
Benztropine	Antiparkinsonian	86-13-5	Y	$C_{21}H_{25}NO$
Benzydamine	Analgesic	642-72-8		$C_{19}H_{23N3O}$
Benzyl Benzoate	Dermatologic	120-51-4		$C_{14}H_{12}O_2$
Benzyl Nicotinate	Dermatologic	94-44-0		$C_{13}H_{11}NO_2$
Benzyl Salicylate	Antiulcerative	118-58-1		$C_{14}H_{12}O_3$
Benzylhydrochlorothiazide	Diuretic	1824-50-6		$C_{14}H_{14}ClN_{3O4S2}$
Benzylpenicillin	Antibiotic	61-33-6		$C_{16}H18N2O4S$
Bepafant	Antithrombotic	114776-28-2		$C_{23}H_{22}ClN_5O_2S$
Beperidium Iodide	Antibiotic	86434-57-3		$C_{23}H_{34}IN_3O_3$
Bephenium Hydroxynaphthoate	Anthelminithic	3818-50-6		$C_{28}H_{29}NO_4$
Bepiastine	Antihistaminic	10189-94-3		$C_{16}H_{17}N_3OS$
Bepotastine	Antihistaminic	125602-71-3		$C_{21}H_{25}ClN_2O_3$
Bepridil	Antianginal	64706-54-3	Y	$C_{24}H_{34}N_2O$
Beraprost Sodium	Prostaglandin	88430-50-6	Y	$C_{24}H_{30}O_5$
Berberine	Antiprotozoal	2086-83-1		$C_{20}H_{18}NO_{4}+$
Berefrine	Mydriatic	105567-83-7		$C_{14}H_{21}NO_2$
Bergenin	Antiulcerative	477-90-7		$C_{14}H_{16}O_9$
Berlafenone	Antiarrhythmic	18965-97-4		$C_{19}H_{25}NO_2$
Bermoprofen	Antiinflammatory	78499-27-1		$C_{18}H_{16}O_4$

Table S1. *Cont.*

Common Name	Indication	CAS Number	Oral	Molecular Formula
Bertosamil	Antiarrhythmic	126825-36-3		$C_{19}H_{36}N_2$
Berupipam	Antipsychotic	150490-85-0		$C_{19}H_{19}BrClNO_2$
Bervastatin	Antihyperlipidemic	132017-01-7		$C_{28}H_{31}FO_5$
Berythromycin	Antiamebic	527-75-3		$C_{37}H_{67}NO_{12}$
Besipirdine	Nootropic	119257-34-0		$C_{16}H_{17}N_3$
Besonprodil	Antiparkinsonian	253450-09-8		$C_{21}H_{23}FN_2O_3S$
Besulpamide	Diuretic	90992-25-9		$C_{15}H_{16}ClN_3O_3S$
Besunide	Glucocorticoid	36148-38-6		$C_{18}H_{22}N_2O_4S$
Betacetylmethadol	Analgesic	17199-59-6		$C_{23}H_{31}NO_2$
Betahistine	Vasodilator	5638-76-6		$C_8H_{12}N_2$
Betaine	Antidote	107-43-7	Y	$C_5H_{11}NO_2$
Betameprodine	Analgesic	468-50-8		$C_{17}H_{25}NO_2$
Betamethadol	Glucocorticoid	17199-55-2		$C_{21}H_{29}NO$
Betamethasone	Glucocorticoid	378-44-9		$C_{22}H_{29}FO_5$
Betamethasone Acetate	Glucocorticoid	987-24-6		$C_{24}H_{31FO6}$
Betamethasone Acibutate	Glucocorticoid	5534-05-4		$C_{28}H_{37}FO_7$
Betamethasone Benzoate	Glucocorticoid	22298-29-9		$C_{29}H_{33}FO_6$
Betamethasone Butyrate Propionate	Glucocorticoid	5534-02-1		$C_{29}H_{39}FO_7$
Betamethasone Dipropionate	Glucocorticoid	5593-20-4		$C_{28}H_{37}FO_7$
Betamethasone Sodium Phosphate	Glucocorticoid	360-63-4		$C_{22}H_{30}FO_8P$
Betamethasone Valerate	Glucocorticoid	2152-44-5		$C_{27}H_{37}FO_6$
Betamicin Sulfate	Antibacterial	36889-15-3		$C_{19}H_{38}N_4O_{10}$
Betamipron	Antibacterial	3440-28-6		$C_{10}H_{11}NO_3$
Betaprodine	Analgesic	468-59-7		$C_{16}H_{23}NO_2$
Betaxolol	Antianginal	63659-18-7	Y	$C_{18}H_{29}NO_3$
Bethanechol	Cholinergic	674-38-4	Y	$C_7H_{17}N_2O_2$
Bethanidine	Antihypertensive	55-73-2	Y	$C_{10}H_{15}N_3$
Betoxycaine	Anesthetic	3818-62-0		$C_{19}H_{32}N_2O_4$
Bevantolol	Antianginal	59170-23-9		$C_{20}H_{27}NO_4$
Bevonium Metilsulfate	Antihypotensive	5205-82-3		$C_{22}H_{28}NO_3.CH_3O_4S$
Bexarotene	Antineoplastic	153559-49-0	Y	$C_{24}H_{28}O_2$
Bexlosteride	Antineoplastic	148905-78-6	Y	$C_{14}H_{16}ClNO$
Bezafibrate	Antihyperlipidemic	41859-67-0	Y	$C_{19}H_{20}ClNO_4$
Bezitramide	Analgesic	15301-48-1		$C_{31}H_{32}N_4O_2$
Bialamicol	Antiamebic	493-75-4	Y	$C_{28}H_{40}N_2O_2$
Biapenem	Antibacterial	120410-24-4		$C_{15}H_{18}N_4O_4S$
Bibenzonium Bromide	Antitussive	15585-70-3	Y	$C_{19}H_{26}BrNO$
Bibrocathol	Unclassified	6915-57-7		$C_6HBiBr_4O_3$
Bicalutamide	Antineoplastic	90357-06-5	Y	$C_{18}H_{14}F_4N_2O_4S$
Bicifadine	Analgesic	71195-57-8	Y	$C_{12}H_{15}N$
Biclodil	Antihypertensive	85125-49-1		$C_8H_8Cl_2N_4O$
Biclofibrate	Antihyperlipidemic	54063-27-3		$C_{20}H_{21}Cl_2NO_4$
Bicozamycin	Antibiotic	38129-37-2		$C_{12}H_{18}N_2O_7$
Bidimazium Iodide	Unclassified	21817-73-2		$C_{26}H_{25}N_2S.I$
Bidisomide	Antiarrhythmic	116078-65-0		$C_{22}H_{34}ClN_3O_2$
Bietamiverine	Antispasmodic	479-81-2		$C_{19}H_{30}N_2O_2$
Bietaserpine	Antihypertensive	53-18-9		$C_{39}H_{53}N_3O_9$
Bifemelane	Nootropic	90293-01-9		$C_{18}H_{23}NO$
Bifepramide	Analgesic	70976-76-0		$C_{21}H_{28}N_2O$
Bifeprofen	Antiinflammatory	108210-73-7		$C_{22}H_{25}ClN_2O_3$
Bifeprunox	Antipsychotic	350992-10-8		$C_{24}H_{23}N_3O_2$
Bifluranol	Steroid	34633-34-6		$C_{17}H_{18}F_2O_2$
Bifonazole	Antifungal	60628-96-8		$C_{22}H_{18}N_2$
Bilastine	Antihistaminic	202189-78-4	Y	$C_{28}H_{37}N_3O_3$
Bimakalim	Antihypertensive	117545-11-6		C_{17}H14N2O2
Bimatoprost	Antiglaucoma	155206-00-1		$C_{25}H_{37}NO_4$
Bimethoxycaine Lactate	Analgesic	47302-54-5		$C_{20}H_{27}NO_2$
Bimoclomol	Capillary Protectant	130493-03-7		$C_{14}H_{20}ClN_3O_2$
Bimosiamose	Antiinflammatory	187269-40-5		$C_{46}H_{54}O_{16}$
Bindarit	Antirheumatic	130641-38-2		$C_{19}H_{20}N_2O_3$
Binedaline	Antidepressant	60662-16-0		$C_{19}H_{23}N_3$
Binfloxacin	Antibacterial	108437-28-1		$C_{19}H_{22}FN_3O_3$
Binifibrate	Antihyperlipidemic	69047-39-8	Y	$C_{25}H_{23}ClN_2O_7$

Table S1. *Cont.*

Common Name	Indication	CAS Number	Oral	Molecular Formula
Binizolast	Bronchodilator	86662-54-6		$C_{18}H_{23}N_5O$
Binodenoson	Vasodilator	144348-08-3		$C_{17}H_{25}N_7O_4$
Binospirone Mesylate	Anxiolytic	102908-59-8		$C_{20}H_{26}N_2O_4$
Bioresmethrin	Dermatologic	28434-01-7		$C_{22}H_{26}O_3$
Bipenamol	Antidepressant	79467-22-4		$C_{14}H_{15}NOS$
Biperiden	Antiparkinsonian	514-65-8	Y	$C_{21}H_{29}NO$
Biphenamine	Anesthetic	3572-52-9		$C_{19}H_{23}NO_3$
Biricodar	Antineoplastic	159997-94-1		$C_{34}H_{41}N_3O_7$
Biriperone	Antipsychotic	42021-34-1		$C_{24}H_{26}FN_3O$
Bisacodyl	Laxative	603-50-9	Y	$C_{22}H_{19}NO_4$
Bisantrene	Antineoplastic	78186-34-2		$C_{22}H_{22}N_8$
Bisaramil	Antiarrhythmic	89194-77-4		$C_{17}H_{23}ClN_2O_2$
Bisbendazole	Antifungal	32195-33-8		$C_{28}H_{28}N_6S_4$
Bisdequalinium Diacetate	Antibacterial	3785-44-2		$C_{44}H_{64}N_4O_4$
Bisfenazone	Antiinflammatory	55837-24-6		$C_{25}H_{29}N_5O_2$
Bisfentidine	Antihistaminic	96153-56-9		$C_{14}H_{18}N_4$
Bismuth Sodium Triglycollamate	Immunomodulator	738525-05-8		$C_6H_8BiNO_7$
Bismuth Sodium Triglycollamate	Immunomodulator	139-13-9		$C_6H_9NO_6$
Bismuth Subsalicylate	Antidiarrheal	14882-18-9		$C_7H_5BiO_4$
Bisnafide Dimesylate	Antineoplastic	144849-63-8		$C_{32}H_{28}N_6O_8$
Bisobrin Lactate	Anticoagulant	22407-74-5		$C_{26}H_{36}$N2O4
Bisoctrizole	Dermatologic	103597-45-1		$C_{41}H_{50}N_6O_2$
Bisoprolol	Antihypertensive	66722-44-9	Y	$C_{18}H_{31}NO_4$
Bisoxatin Acetate	Laxative	14008-48-1		$C_{24}H_{19}NO_6$
Bithionolate Sodium	Antibacterial	97-18-7		$C_{12}H_6Cl_4O_2S$
Bithionoloxide	Anthelminithic	844-26-8		$C_{12}H_6Cl_4O_3S$
Bitipazone	Antiinflammatory	13456-08-1		$C_{20}H_{38}N_8S_2$
Bitolterol	Bronchodilator	30392-40-6		$C_{28}H_{31}NO_5$
Bitoscanate	Anthelminithic	4044-65-9		$C_8H_4N_2S_2$
Bizelesin	Antineoplastic	129655-21-6		$C_{43}H_{36}Cl_2N_8O_5$
Blastomycin	Antifungal	522-70-3		$C_{26}H_{36}N_2O_9$
Blonanserin	Antipsychotic	132810-10-7	Y	$C_{23}H_{30}FN_3$
Bluensomycin	Antineoplastic	11011-72-6		$C_{21}H_{39}N_5O_{14}$
Bofumustine	Antineoplastic	55102-44-8		$C_{18}H_{21}ClN_4O_9$
Bolandiol Dipropionate	Steroid	1986-53-4		$C_{24}H_{36}O_4$
Bolasterone	Steroid	1605-89-6		$C_{21}H_{32}O_2$
Bolazine	Antiinflammatory	4267-81-6		$C_{40}H_{64}N_2O_2$
Boldenone Undecylenate	Steroid	13103-34-9		$C_{30}H_{44}O_3$
Bolenol	Steroid	16915-78-9		$C_{20}H_{32}O$
Bolmantalate	Steroid	1491-81-2		$C_{29}H_{40}O_3$
Bometolol	Antiarrhythmic	65008-93-7		$C_{25}H_{32}N_2O_7$
Bopindolol	Antihypertensive	62658-63-3	Y	$C_{23}H_{28}N_2O_3$
Bornaprine	Antihypertensive	20448-86-6		$C_{21}H_{31}NO_2$
Bornaprolol	Antihypertensive	66451-06-7		C19H29NO2
Bortezomib	Antineoplastic	179324-69-7		C19H25BN4O4
Bosentan	Anticoagulant	147536-97-8	Y	$C_{27}H_{29}N_5O_6S$
Bosutinib	Antineoplastic	380843-75-4	Y	$C_{26}H_{29}Cl_2N_5O_3$
Botiacrine	Nootropic	4774-53-2		$C_{20}H_{24}N_2OS$
Boxidine	Antihyperlipidemic	10355-14-3		$C_{19}H_{20}F_3NO$
Brallobarbital	Sedative	561-86-4		$C_{10}H_{11}BrN_2O_3$
Brasofensine	Antiparkinsonian	171655-91-7		$C_{16}H_{20}Cl_2N_2O$
Brazergoline	Antiparkinsonian	60019-20-7		$C_{23}H_{30}BrN_3O_2$
Brecanavir	Antiviral	313682-08-5		$C_{33}H_{41}N_3O_{10}S_2$
Brefonalol	Antiarrhythmic	103880-26-8		$C_{22}H_{28}N_2O_2$
Bremazocine	Analgesic	75684-07-0		$C_{20}H_{29}NO_2$
Brequinar Sodium	Antineoplastic	96187-53-0		$C_{23}H_{15}F_2NO_2$
Bretazenil	Anxiolytic	84379-13-5		$C_{19}H_{20}BrN_3O_3$
Bretylium	Antiarrhythmic	59-41-6		$C_{11}H_{17}BrN$
Brimonidine	Antiglaucoma	59803-98-4		$C_{11}H_{10}BrN_5$
Brinazarone	Antiarrhythmic	89622-90-2		$C_{25}H_{32}N_2O_2$
Brindoxime	Analgesic	55837-17-7		$C_{18}H_{19}Br_2N_5O_2$
Brinzolamide	Antiglaucoma	138890-62-7		$C_{12}H_{21}N_3O_5S_3$
Brivaracetam	Nootropic	357336-20-0	Y	$C_{11}H_{20}N_2O_2$

Table S1. *Cont.*

Common Name	Indication	CAS Number	Oral	Molecular Formula
Brivudine	Antiviral	69304-47-8		$C_{11}H_{13}BrN_2O_5$
Brobactam	Antibiotic	26631-90-3		$C_8H_{10}BrNO_3S$
Broclepride	Gastroprokinetic	71195-56-7		$C_{20}H_{23}BrClN_3O_2$
Brocresine	Antiparkinsonian	555-65-7		$C_7H_8BrNO_2$
Brocrinat	Diuretic	72481-99-3		$C_{15}H_9BrFNO_4$
Brodimoprim	Antineoplastic	56518-41-3		$C_{13}H_{15}BrN_4O_2$
Brofaromine	Antidepressant	63638-91-5		$C_{14}H_{16}BrNO_2$
Brofezil	Nootropic	17969-45-8		$C_{12}H_{10}BrNO_2S$
Brofoxine	Antipsychotic	21440-97-1		$C_{10}H_{10}BrNO_2$
Brolaconazole	Antifungal	118528-04-4		$C_{17}H_{15}BrN_2$
Brolamfetamine	Nootropic	64638-07-9		$C_{11}H_{16}BrNO_2$
Bromacrylide	Antineoplastic	4213-51-8		$C_7H_{11}BrN_2O_2$
Bromadoline Maleate	Analgesic	67579-24-2		$C_{15}H_{21}BrN_2O$
Bromamid	Unclassified	332-69-4		$C_{11}H_{15}BrN_2O$
Bromazepam	Anxiolytic	1812-30-2	Y	$C_{14}H_{10}BrN_3O$
Bromebric Acid	Antineoplastic	5711-40-0		$C_{11}H_9BrO_4$
Bromerguride	Antipsychotic	83455-48-5		$C_{20}H_{25}BrN_4O$
Bromfenac	Antiinflammatory	91714-94-2		$C_{15}H_{12}BrNO_3$
Bromhexine	Expectorant	3572-43-8	Y	$C_{14}H_{20}Br_2N_2$
Bromindione	Anticoagulant	1146-98-1		$C_{15}H_9BrO_2$
Bromisovalum	Sedative	496-67-3		$C_6H_{11}BrN_2O_2$
Bromociclen	Unclassified	1715-40-8		$C_8H_5BrCl_6$
Bromocriptine	Pituitary	25614-03-3	Y	$C_{32}H_{40}BrN_5O_5$
Bromodiphenhydramine	Antihistaminic	118-23-0		$C_{17}H_{20}BrNO$
Bromofenofos	Antineoplastic	21466-07-9		$C_{12}H_7Br_4O_5P$
Bromoform	Sedative	75-25-2		$CHBr_3$
Bromofos	Antineoplastic	2104-96-3		$C_8H_8BrCl_2O_3PS$
Bromopride	Antiemetic	4093-35-0		$C_{14}H_{22}BrN_3O_2$
Bromoxanide	Anthelminithic	41113-86-4		$C_{19}H_{18}BrF3N2O4$
Bromperidol	Antipsychotic	10457-90-6		$C_{21}H_{23}BrFNO_2$
Bromperidol Decanoate	Antipsychotic	75067-66-2		$C_{31}H_{41}BrFNO_3$
Brompheniramine	Antihistaminic	86-22-6	Y	$C_{16}H_{19}BrN_2$
Broparestrol	Estrogen	479-68-5		$C_{22}H_{19}Br$
Broperamole	Antiinflammatory	33144-79-5		$C_{15}H_{18BrN5O}$
Bropirimine	Antineoplastic	56741-95-8		$C_{10}H_8BrN_3O$
Brosotamide	Nootropic	40912-73-0		$C_8H_8BrNO_2$
Brostallicin	Antibiotic	203258-60-0		$C_{30}H_{35}BrN_{12}O_5$
Brosuximide	Antineoplastic	22855-57-8		$C_{10}H_8BrNO_2$
Brotianide	Antiarrhythmic	23233-88-7		$C_{15}H_{10}Br2ClNO2S$
Brotizolam	Sedative	57801-81-7	Y	$C_{15}H_{10}BrClN_{4S}$
Brovanexine	Expectorant	54340-61-3		$C_{24}H_{28}Br_2N_2O_4$
Brovincamine	Vasodilator	57475-17-9	Y	$C_{21}H_{25}BrN_2O_3$
Broxaldine	Antibacterial	3684-46-6		$C_{17}H_{11}Br_2NO_2$
Broxaterol	Bronchodilator	76596-57-1		$C_9H_{15}BrN_2O_2$
Broxitalamic Acid	Unclassified	86216-41-3		$C_{12}H_{11}Br_3N_2O_5$
Broxuridine	Antineoplastic	59-14-3		$C_9H_{11}BrN_2O_5$
Brucine	Nootropic	357-57-3		$C_{23}H_{26}N_2O_4$
Bucainide Maleate	Antiarrhythmic	51481-62-0		$C_{21}H_{35}N_3$
Bucetin	Analgesic	1083-57-4		$C_{12}H_{17}NO_3$
Buciclovir	Antiviral	86304-28-1		$C_9H_{13}N_5O_3$
Bucillamine	Antirheumatic	65002-17-7	Y	$C_7H_{13}NO_3S_2$
Bucindolol	Antihypertensive	71119-11-4	Y	$C_{22}H_{25}N_3O_2$
Bucladesine	Cardiotonic	362-74-3		$C_{18}H_{24}N_5O_8P$
Buclizine	Antiemetic	82-95-1		$C_{28}H_{33}ClN_2$
Buclosamide	Antifungal	575-74-6		$C_{11}H_{14}ClNO_2$
Bucloxic Acid	Antiinflammatory	32808-51-8		$C_{16}H_{19}ClO_3$
Bucolome	Antiinflammatory	841-73-6		$C_{14}H_{22}N_2O_3$
Bucricaine	Analgesic	316-15-4		$C_{17}H_{22}N_2$
Bucromarone	Antiarrhythmic	78371-66-1		$C_{29}H_{37}NO_4$
Bucumolol	Antianginal	58409-59-9		$C_{17}H_{23}NO_4$
Budesonide	Glucocorticoid	51333-22-3	Y	$C_{25}H_{34}O_6$
Budipine	Antiparkinsonian	57982-78-2	Y	$C_{21}H_{27}N$
Budralazine	Antihypertensive	36798-79-5		$C_{14}H_{16}N_4$

Table S1. *Cont.*

Common Name	Indication	CAS Number	Oral	Molecular Formula
Bufenadrine	Antihistaminic	604-74-0		$C_{21}H_{29}NO$
Bufeniode	Antihypertensive	22103-14-6		$C_{19}H_{23}I_2NO_2$
Bufetolol	Antiarrhythmic	53684-49-4		$C_{18}H_{29}NO_4$
Bufexamac	Antiinflammatory	2438-72-4		$C_{12}H_{17}NO_3$
Bufezolac	Antiinflammatory	50270-32-1		$C_{21}H_{22}N_2O_2$
Buflomedil	Vasodilator	55837-25-7	Y	$C_{17}H_{25}NO_4$
Bufogenin	Cardiotonic	465-39-4		$C_{24}H_{32}O_4$
Buformin	Antidiabetic	692-13-7		$C_6H_{15}N_5$
Bufrolin	Antihistaminic	54867-56-0		$C_{18}H_{16}N_2O_6$
Bufuralol	Antianginal	54340-62-4		$C_{16}H_{23}NO_2$
Bulaquine	Antimalarial	79781-00-3	Y	$C_{21}H_{27}N_3O_3$
Bumadizone	Analgesic	3583-64-0		$C_{19}H_{22}N_2O_3$
Bumecaine	Anesthetic	30103-44-7		$C_{18}H_{28}N_2O$
Bumepidil	Antihypertensive	62052-97-5		$C_{12}H_{17}N_5$
Bumetanide	Diuretic	28395-03-1	Y	$C_{17}H_{20}N_2O_5S$
Bumetrizole	Dermatologic	3896-11-5		$C_{17}H_{18}ClN_3O$
Bunaftine	Antiarrhythmic	32421-46-8		$C_{21}H_{30}N_2O$
Bunamidine	Anthelminithic	3748-77-4		$C_{25}H_{38}N_2O$
Bunamiodyl Sodium	Unclassified	1233-53-0		$C_{15}H_{16}I_3NO_3$
Bunaprolast	Bronchodilator	99107-52-5		$C_{17}H_{20}O_3$
Bunazosin	Antihypertensive	80755-51-7	Y	$C_{19}H_{27}N_5O_3$
Bunitrolol	Antianginal	34915-68-9		$C_{14}H_{20}N_2O_2$
Bunolol	Antihypertensive	27591-01-1		$C_{17}H_{25}NO_3$
Buparvaquone	Antineoplastic	88426-33-9		$C_{21}H_{26}O_3$
Bupicomide	Antihypertensive	22632-06-0		$C_{10}H_{14}N_2O$
Bupivacaine	Anesthetic	38396-39-3		$C_{18}H_{28}N_2O$
Bupranolol	Antianginal	14556-46-8		$C_{14}H_{22}ClNO_2$
Buprenorphine	Analgesic	52485-79-7		$C_{29}H_{41}NO_4$
Bupropion	Antidepressant	34911-55-2	Y	$C_{13}H_{18}ClNO$
Buquineran	Cardiotonic	59184-78-0		$C_{20}H_{29}N_5O_3$
Buquinolate	Antibacterial	5486/3/3		$C_{20}H_{27}NO_5$
Buquiterine	Antispasmodic	76536-74-8		$C_{18}H_{23}N_3O_3$
Buramate	Anticonvulsant	4663-83-6		$C_{10}H_{13}NO_3$
Burodiline	Vasodilator	36121-13-8		$C_{19}H_{29}NO_5$
Buspirone	Anxiolytic	36505-84-7	Y	$C_{21}H_{31}N_5O_2$
Busulfan	Antineoplastic	55-98-1	Y	$C_6H_{14}O_6S_2$
Butabarbital	Sedative	125-40-6	Y	$C_{10}H_{16}N_2O_3$
Butacaine	Anesthetic	149-16-6		$C_{18}H_{30}N_2O_2$
Butacetin	Analgesic	2109-73-1		$C_6H_{12}O$
Butaclamol	Antipsychotic	51152-91-1		$C_{25}H_{31}NO$
Butadiazamide	Analgesic	7007-88-7		$C_{12}H_{14}ClN_3O_2S_2$
Butafosfan	Antineoplastic	17316-67-5		$C_7H_{18}NO_2P$
Butalamine	Vasodilator	22131-35-7		$C_{18}H_{28}N_4O$
Butalbital	Sedative	77-26-9		$C_{11}H_{16}N_2O_3$
Butallylonal	Sedative	1142-70-7		$C_{11}H_{15}BrN_2O_3$
Butamben	Anesthetic	94-25-7		$C_{11}H_{15}NO_2$
Butamin	Unclassified	891-33-8		$C_{14}H_{22}N_2O_2$
Butamirate	Anesthetic	18109-80-3	Y	$C_{18}H_{29}NO_3$
Butamisole	Anthelminithic	54400-59-8		$C_{15}H_{19}N_3OS$
Butamoxane	Sedative	4442-60-8		$C_{13}H_{19}NO_2$
Butanilicaine	Anesthetic	3785-21-5		$C_{13}H_{19}ClN_2O$
Butanixin	Antiinflammatory	55285-35-3		$C_{16}H_{18}N_2O_2$
Butanserin	Antihypertensive	87051-46-5		$C_{24}H_{26}FN_3O_3$
Butantrone	Dermatologic	75464-11-8		$C_{18}H_{16}O_4$
Butaperazine	Antipsychotic	653-03-2		$C_{24}H_{31}N_3OS$
Butaprost	Bronchodilator	69648-38-0		$C_{24}H_{40}O_5$
Butaverine	Antispasmodic	55837-14-4		$C_{18}H_{27}NO_2$
Butedronic Acid	Bone Resorption Inhibitor	51395-42-7		$C_5H_{10}O_{10}P_2$
Butenafine	Antifungal	101828-21-1		$C_{23}H_{27}N$
Buterizine	Vasodilator	68741-18-4		$C_{31}H_{38}N_4$
Butetamate	Antitussive	14007-64-8		$C_{16}H_{25}NO_2$
Butethal	Sedative	77-28-1		$C_{10}H_{16}N_2O_3$
Butethamine	Anesthetic	2090-89-3		$C_{13}H_{20}N_2O_2$

Table S1. *Cont.*

Common Name	Indication	CAS Number	Oral	Molecular Formula
Buthalital Sodium	Anesthetic	468-65-5		$C_{11}H_{16}N_2O_2S$
Buthiazide	Diuretic	2043-38-1		$C_{11}H_{16}ClN_3O_4S_2$
Butibufen	Antiinflammatory	55837-18-8		$C_{14}H_{20}O_2$
Butidrine	Antiarrhythmic	7433-10-5		$C_{16}H_{25}NO$
Butikacin	Antibacterial	59733-86-7		$C_{22}H_{45}N_5O_{12}$
Butinazocine	Analgesic	93821-75-1		$C_{18}H_{23}NO_2$
Butixirate	Analgesic	5769-10-8		$C_{12}H_{17}N$
Butixocort	Bronchodilator	120815-74-9		$C_{25}H_{36}O_5S$
Butobendine	Antiarrhythmic	55769-65-8		$C_{32}H_{48}N_2O_{10}$
Butoconazole	Antifungal	64872-76-0		$C_{19}H_{17}Cl_3$N2S
Butocrolol	Unclassified	55165-22-5		$C_{19}H_{23NO_6}$
Butoctamide	Sedative	32838-26-9		C12H25NO2
Butofilolol	Antihypertensive	58930-32-8		$C_{17}H_{26}FNO_3$
Butonate	Anthelminithic	126-22-7		$C_8H_{14}Cl_3O_5P$
Butopamine	Cardiotonic	66734-12-1		$C_{18}H_{23}NO_3$
Butopiprine	Unclassified	55837-15-5		$C_{19}H_{29}NO_3$
Butoprozine	Antiarrhythmic	62228-20-0		$C_{28}H_{38}N_2O_2$
Butorphanol	Analgesic	42408-82-2		$C_{21}H_{29}NO_2$
Butoxamine	Antidiabetic	1937-89-9		$C_{15}H_{25}NO_3$
Butoxylate	Antihypertensive	15302-05-3		$C_{32}H_{36}N_2O_2$
Butriptyline	Antidepressant	35941-65-2	Y	$C_{21}H_{27}N$
Butropium Bromide	Antispasmodic	29025-14-7		$C_{28}H_{38}NO_4$.Br
Butyl Chloride	Anthelminithic	109-69-3		C_4H_9Cl
Butylphenamide	Antiglaucoma	131-90-8		$C_{17}H_{19}NO_2$
Butylscopolamine Bromide	Antispasmodic	149-64-4	Y	$C_{21}H_{30}BrNO_4$
Butynamine	Antispasmodic	3735-65-7		$C_{10}H_{19}N$
Buzepide Metiodide	Antispasmodic	15351-05-0		$C_{23}H_{31}N_2O$.I
Cabastine	Antineoplastic	79449-98-2		$C_{26}H_{29}FN_2O_2$
Cabergoline	Antiparkinsonian	81409-90-7	Y	$C_{26}H_{37}N_5O_2$
Cadralazine	Vasodilator	64241-34-5	Y	$C_{12}H_{21}N_5O_3$
Cadrofloxacin	Antibiotic	153808-85-6		$C_{19}H_{20}F_3N_3O_4$
Cafaminol	Decongestant	30924-31-3		$C_{11}H_{17}N_5O_3$
Cafedrine	Nootropic	58166-83-9		$C_{18}H_{23}N_5O_3$
Caffeine	Nootropic	58-08-2	Y	$C_8H_{10}N_4O_2$
Calcifediol	Bone Resorption Inhibitor	19356-17-3	Y	$C_{27}H_{44}O_2$
Calcipotriene	Dermatologic	112965-21-6		$C_{27}H_{40}O_3$
Calcium Carbimide	Alcohol Deterrant	420-04-2		CH_2N_2
Calcium Dobesilate	Capillary Protectant	88-46-0		$C_6H_6O_5S$
Calcobutrol	Unclassified	138168-36-2		$C_{18}H_{34}N_4O_9$
Caldaret	Cardiotonic	133804-44-1		$C_{11}H_{16}N_2O_3S$
Calusterone	Antineoplastic	17021-26-0		$C_{21}H_{32}O_2$
Camazepam	Anxiolytic	36104-80-0		$C_{19}H_{18}ClN_3O_3$
Cambendazole	Anthelminithic	26097-80-3		$C_{14}H_{14}N_4O_2S$
Camiglibose	Antidiabetic	127214-23-7		$C_{13}H_{25}NO_9$
Camiverine	Antispasmodic	54063-28-4		$C_{19}H_{30}N_2O_2$
Camonagrel	Anticoagulant	105920-77-2		$C_{15}H_{16}N_2O_3$
Camostat	Antiviral	59721-28-7	Y	$C_{20}H_{22}N_4O_5$
Camphor	Analgesic	76-22-2		$C_{10}H_{16}O$
Camphoric Acid	Analgesic	5394-83-2		$C_{10}H_{16}O_4$
Camphotamide	Nootropic	4876-45-3		$C_{21}H_{32}N_2O_5S$
Camptothecin	Antineoplastic	7689-03-4		$C_{20}H_{16}N_2O_4$
Camylofin	Antispasmodic	54-30-8		$C_{19}H_{32}N_2O_2$
Canbisol	Anxiolytic	56689-43-1		$C_{24}H_{38}O_3$
Candesartan	Antihypertensive	139481-59-7	Y	$C_{24}H_{20}N_6O_3$
Candesartan Cilexetil	Antihypertensive	145040-37-5	Y	$C_{33}H_{34}N_6O_6$
Candocuronium Iodide	Muscle Relaxant	54278-85-2		$C_{26}H_{46}I_2N_2$
Candoxatril	Cardiotonic	123122-55-4		$C_{29}H_{41}NO_7$
Candoxatrilat	Cardiotonic	123122-54-3		$C_{20}H_{33}NO_7$
Canertinib Dihydrochloride	Antineoplastic	267243-28-7		$C_{24}H_{25}ClFN_5O_3$
Canfosfamide	Antineoplastic	158382-37-7		$C_{26}H_{40}Cl_4N_5O_{10}PS$
Cangrelor	Anticoagulant	163706-06-7		$C_{17}H_{25}Cl_2F_3N_5O_{12}P_3S_2$
Cannabinol	Antiemetic	521-35-7		$C_{21}H_{26}O_2$
Canrenoate	Diuretic	4138-96-9		$C_{22}H_{30}O_4$

Table S1. *Cont.*

Common Name	Indication	CAS Number	Oral	Molecular Formula
Canrenone	Diuretic	976-71-6		$C_{22}H_{28}O_3$
Cantharides	Dermatologic	56-25-7		$C_{10}H_{12}O_4$
Capecitabine	Antineoplastic	154361-50-9	Y	$C_{15}H_{22}FN_3O_6$
Capobenic Acid	Antiarrhythmic	21434-91-3		$C_{16}H_{23}NO_6$
Capravirine	Antiviral	178979-85-6		$C_{20}H_{20}Cl_2N_4O_2S$
Capromorelin Tartrate	Cardiotonic	193273-66-4		$C_{28}H_{35}N_5O_4$
Caproxamine	Antidepressant	53078-44-7		$C_{15}H_{25}N_3O$
Caprylate	Antifungal	74-81-7		$C_8H_{15}O_2$
Capsaicin	Analgesic	404-86-4		$C_{18}H_{27}NO_3$
Captamine	Dermatologic	108-02-1		$C_4H_{11}NS$
Captodiame	Anxiolytic	486-17-9		$C_{21}H_{29}NS_2$
Captopril	Antihypertensive	62571-86-2	Y	$C_9H_{15}NO_3S$
Capuride	Sedative	5579-13-5		$C_9H_{18}N_2O_2$
Carabersat	Antimigraine	184653-84-7		$C_{20}H_{20}FNO_4$
Caracemide	Antineoplastic	81424-67-1		$C_6H_{11}N_3O_4$
Carafiban	Antithrombotic	177563-40-5		$C_{24}H_{27}N_5O_5$
Caramiphen	Antitussive	77-22-5		$C_{18}H_{27}NO_2$
Carazolol	Antihypertensive	57775-29-8		$C_{18}H_{22}N_2O_2$
Carbachol	Cholinergic	51-83-2		$C_6H_{15}N_2O_2.Cl$
Carbadox	Antibacterial	6804-07-5		$C_{11}H_{10}N_4O_4$
Carbamazepine	Anticonvulsant	298-46-4	Y	$C_{15}H_{12}N_2O$
Carbantel Lauryl Sulfate	Anthelminithic	22790-84-7		$C_{12}H_{16}ClN_3O$
Carbantel Lauryl Sulfate	Anthelminithic	151-41-7		$C_{12}H_{26}O_4S$
Carbaril	Ectoparasiticide	63-25-2		$C_{12}H_{11}NO_2$
Carbarsone	Antiamebic	121-59-5		$C_7H_9AsN_2O_4$
Carbazeran	Cardiotonic	70724-25-3		$C_{18}H_{24}N_4O_4$
Carbazochrome	Hemostatic	69-81-8	Y	$C_{10}H_{12}N_4O_3$
Carbazocine	Analgesic	15686-38-1		$C_{22}H_{28}N_2$
Carbenicillin	Antibiotic	4697-36-3		$C_{17}H_{18}N_2O_6S$
Carbenicillin Indanyl Sodium	Antibiotic	35531-88-5		$C_{26}H_{26}N_2O_6S$
Carbenicillin Phenyl Sodium	Antibiotic	27025-49-6		$C_{23}H_{22}N_2O_6S$
Carbenoxolone	Antiulcerative	5697-56-3	Y	C34H50O7
Carbenzide	Antipsychotic	3240-20-8		$C_{11}H_{16}N_2O_2$
Carbetapentane Citrate	Antitussive	77-23-6		$C_{20}H_{31}NO_3$
Carbethyl Salicylate	Unclassified	118-27-4		$C_{19}H_{18}O_7$
Carbetocin	Oxytocic	37025-55-1		$C_{45}H_{69}N_{11}O_{12}S$
Carbidopa	Antiparkinsonian	28860-95-9	Y	$C_{10}H_{14}N_2O_4$
Carbimazole	Thyroid	22232-54-8	Y	$C_7H_{10}N_2O_2S$
Carbinoxamine	Antihistaminic	486-16-8	Y	$C_{16}H_{19}ClN_2O$
Carbiphene	Analgesic	15687-16-8		$C_{28}H_{34}N_2O_2$
Carbocloral	Sedative	541-79-7		$C_5H_8Cl_3NO_3$
Carbocysteine	Mucolytic	638-23-3	Y	$C_5H_9NO_4S$
Carbofenotion	Dermatologic	786-19-6		$C_{11}H_{16}ClO_2PS_3$
Carbol Fuchsin	Antifungal	682730-74-1		$C_{21}H_{21}N_3$
Carbomycin	Antibiotic	4564-87-8		$C_{42}H_{67}NO_{16}$
Carbon Tetrachloride	Anthelminithic	56-23-5		CCl_4
Carboprost	Oxytocic	35700-23-3		$C_{21}H_{36}O_5$
Carboprost Methyl	Oxytocic	35700-21-1		$C_{22}H_{38}O_5$
Carboquone	Antineoplastic	24279-91-2		$C_{15}H_{19}N_3O_5$
Carbromal	Sedative	77-65-6		$C_7H_{13}BrN_2O_2$
Carbubarb	Sedative	960-05-4		$C_{11}H_{17}N_3O_5$
Carburazepam	Anxiolytic	59009-93-7		$C_{17}H_{16}ClN_3O_2$
Carbutamide	Antidiabetic	339-43-5		$C_{11}H_{17}N_3O_3S$
Carbuterol	Bronchodilator	34866-47-2		$C_{13}H_{21}N_3O_3$
Carcainium Chloride	Anesthetic	1042-42-8		$C_{18}H_{22}ClN_3O_2$
Carebastine	Antihistaminic	90729-42-3		$C_{32}H_{37}NO_4$
Carfentanil Citrate	Analgesic	59708-52-0		$C_{24}H_{30}N_2O_3$
Carfimate	Sedative	3567-38-2		$C_{10}H_9NO_2$
Cargutocin	Oxytocic	33605-67-3		$C_{42}H_{65}N_{11}O_{12}$
Caricotamide	Antithrombotic	64881-21-6		$C_8H_{11}N_3O_2$
Cariporide	Cardiotonic	159138-80-4		$C_{12}H_{17}N_3O_3S$
Carisoprodol	Muscle Relaxant	78-44-4	Y	$C_{12}H_{24}N_2O_4$
Carmantadine	Antiparkinsonian	38081-67-3		$C_{14}H_{21}NO_2$

Table S1. *Cont.*

Common Name	Indication	CAS Number	Oral	Molecular Formula
Carmetizide	Antidiabetic	42583-55-1		$C_{10}H_{12}ClN_3O_6S_2$
Carmofur	Antineoplastic	61422-45-5		$C_{11}H_{16}FN_3O_3$
Carmoterol	Bronchodilator	147568-66-9		$C_{21}H_{24}N_2O_4$
Carmoxirole	Antihypertensive	98323-83-2		$C_{24}H_{26}N_2O_2$
Carmustine	Antineoplastic	154-93-8		$C_5H_9Cl_2N_3O_2$
Carnidazole	Antiprotozoal	42116-76-7		C_8H12N4O3S
Carnitine	Antihyperlipidemic	541-15-1		$C_7H_{15}NO_3$
Carocainide	Antiarrhythmic	66203-00-7		C18H25N3O5
Caroverine	Antispasmodic	23465-76-1		$C_{22}H_{27}N_3O_2$
Caroxazone	Antidepressant	18464-39-6		$C_{10}H_{10}N_2O_3$
Carperidine	Analgesic	7008-32-4		$C_{17}H_{24}N_2O_3$
Carperone	Antipsychotic	20977-50-8		$C_{19}H_{27}FN_2O_3$
Carphenazine	Antipsychotic	2622-30-2		$C_{24}H_{31}N_3O_2S$
Carpindolol	Antihypertensive	39731-05-0		$C_{19}H_{28}N_2O_4$
Carpipramine	Antipsychotic	5942-95-0	Y	$C_{28}H_{38}N_4O$
Carprazidil	Antihypertensive	68020-77-9		$C_{12}H_{13}N_5O_4$
Carprofen	Antiinflammatory	53716-49-7	Y	$C_{15}H_{12}ClNO_2$
Carpronium Chloride	Cholinergic	13254-33-6		$C_8H_{18}ClNO_2$
Carsalam	Analgesic	2037-95-8		$C_8H_5NO_3$
Carsatrin Succinate	Cardiotonic	125345-93-9		$C_{25}H_{26}F_2N_6OS$
Cartasteine	Mucolytic	149079-51-6		$C_9H_{14}N_2O_4S_2$
Cartazolate	Antidepressant	34966-41-1		$C_{15}H_{22}N_4O_2$
Carteolol	Antihypertensive	51781-06-7		$C_{16}H_{24}N_2O_3$
Carubicin	Antineoplastic	50935-04-1		$C_{26}H_{27}NO_{10}$
Carumonam Sodium	Antibacterial	87638-04-8		$C_{12}H_{14}N_6O_{10}S_2$
Carvacrol	Anthelminithic	499-75-2		$C_{10}H_{14}O$
Carvedilol	Antihypertensive	72956-09-3	Y	$C_{24}H_{26}N_2O_4$
Carvotroline	Antipsychotic	107266-08-0		$C_{18}H_{18}FN_3$
Carzelesin	Antineoplastic	119813-10-4		$C_{41}H_{37}ClN_6O_5$
Carzenide	Unclassified	138-41-0		$C_7H_7NO_4S$
Casokefamide	Antiulcerative	98815-38-4		$C_{33}H_{40}N_6O_7$
Cathine	Anorexic	492-39-7		$C_9H_{13}NO$
Cathinone	Nootropic	71031-15-7		$C_9H_{11}NO$
Cebaracetam	Nootropic	113957-09-8		$C_{16}H_{18}ClN_3O_3$
Cedefingol	Antineoplastic	35301-24-7		$C_{20}H_{41}NO_3$
Cefaclor	Antibiotic	53994-73-3	Y	$C_{15}H_{14}ClN_3O_4S$
Cefadroxil	Antibiotic	50370-12-2	Y	$C_{16}H_{17}N_3O_5S$
Cefalonium	Antibiotic	5575-21-3		$C_{20}H_{18}N_4O_5S_2$
Cefaloram	Antibiotic	859-07-4		$C_{18}H_{18}N_2O_6S$
Cefamandole	Antibiotic	34444-01-4		$C_{18}H_{18}N_6O_5S_2$
Cefamandole Nafate	Antibiotic	57268-80-1		$C_{19}H_{18}N_6O_6S_2$
Cefaparole	Antibiotic	51627-20-4		$C_{19}H_{19}N_5O_5S_3$
Cefatrizine	Antibiotic	51627-14-6		$C_{18}H_{18}N_6O_5S_2$
Cefazaflur	Antibiotic	58665-96-6		$C_{13}H_{13}F_3N_6O_4S_3$
Cefazedone	Antibiotic	56187-47-4		$C_{18}H_{15}Cl_2N_5O_5S_3$
Cefazolin	Antibiotic	25953-19-9		$C_{14}H_{14}N_8O_4S_3$
Cefbuperazone	Antibiotic	76610-84-9		$C_{22}H_{29}N_9O_9S_2$
Cefcanel	Antibiotic	41952-52-7		$C_{19}H_{18}N_4O_5S_3$
Cefcanel Daloxate	Antibiotic	97275-40-6		$C_{27}H_{27}N_5O_9S_3$
Cefcapene	Antibiotic	135889-00-8	Y	$C_{17}H_{19}N_5O_6S_2$
Cefclidin	Antibiotic	105239-91-6		$C_{21}H_{26}N_8O_6S_2$
Cefdaloxime	Antibiotic	80195-36-4		$C_{14}H_{15}N_5O_6S_2$
Cefdinir	Antibiotic	91832-40-5	Y	$C_{14}H_{13}N_5O_5S_2$
Cefditoren	Antibiotic	104145-95-1	Y	$C_{19}H_{18}N_6O_5S_3$
Cefditoren Pivoxil	Antibiotic	117467-28-4	Y	$C_{25}H_{28}N_6O_7S_3$
Cefedrolor	Antibiotic	57847-69-5		$C_{16}H_{16}ClN_3O_5S$
Cefempidone	Antibiotic	103238-57-9		$C_{22}H_{21}N_7O_6S_2$
Cefepime	Antibiotic	88040-23-7		$C_{19}H_{24}N_6O_5S_2$
Cefetamet	Antibiotic	65052-63-3		$C_{14}H_{15}N_5O_5S_2$
Cefetecol	Antibiotic	117211-03-7		$C_{20}H_{17}N_5O_9S_2$
Cefetrizole	Antibiotic	65307-12-2		$C_{16}H_{15}N_5O_4S_3$
Cefivitril	Antibiotic	66474-36-0		$C_{15}H_{15}N_7O_4S_3$
Cefixime	Antibiotic	79350-37-1		$C_{16}H_{15}N_5O_7S_2$

Table S1. *Cont.*

Common Name	Indication	CAS Number	Oral	Molecular Formula
Cefluprenam	Antibiotic	116853-25-9		$C_{20}H_{25}FN_8O_6S_2$
Cefmatilen	Antibiotic	140128-74-1		$C_{15}H_{14}N_8O_5S_4$
Cefmenoxime	Antibiotic	65085-01-0		$C_{16}H_{17}N_9O_5S_3$
Cefmepidium Chloride	Antibiotic	107452-79-9		$C_{23}H_{25}N_6O_8S_3.Cl$
Cefmetazole	Antibiotic	56796-20-4		$C_{15}H_{17}N_7O_5S_3$
Cefminox	Antibiotic	84305-41-9		$C_{16}H_{21}N_7O_7S_3$
Cefodizime	Antibiotic	69739-16-8		$C_{20}H_{20}N_6O_7S_4$
Cefonicid	Antibiotic	61270-58-4		$C_{18}H_{18}N_6O_8S_3$
Cefoperazone	Antibiotic	62893-19-0		$C_{25}H_{27}N_9O_8S_2$
Ceforanide	Antibiotic	60925-61-3		C_{20}H21N7O6S2
Cefoselis	Antibiotic	122841-10-5		$C_{19}H_{22}N_8O_6S_2$
Cefotaxime	Antibiotic	63527-52-6		$C_{16}H_{17}N_5$O7S2
Cefotetan	Antibiotic	69712-56-7		$C_{17}H_{17}N_7O_8S_4$
Cefotiam	Antibiotic	61622-34-2		$C_{18}H_{23}N_9O_4S_3$
Cefovecin Sodium	Antibiotic	234096-34-5		$C_{17}H_{19}N_5O_6S_2$
Cefoxazole	Antibiotic	36920-48-6		$C_{21}H_{18}ClN_3O_7S$
Cefoxitin	Antibiotic	35607-66-0		$C_{16}H_{17}N_3O_7S_2$
Cefozopran	Antibiotic	113359-04-9		$C_{19}H_{17}N_9O_5S_2$
Cefpimizole	Antibiotic	84880-03-5		$C_{28}H_{26}N_6O_{10}S_2$
Cefpiramide	Antibiotic	70797-11-4		C25H24N8O7S2
Cefpodoxime Proxetil	Antibiotic	87239-81-4	Y	$C_{21}H_{27}N_5O_9S_2$
Cefprozil	Antibiotic	92665-29-7	Y	$C_{18}H_{19}N_3O_5S$
Cefquinome Sulfate	Antibiotic	118443-88-2		$C_{23}H_{25}N_6O_5S_2$
Cefrotil	Antibiotic	52231-20-6		$C_{20}H_{22}N_4O_4S$
Cefroxadine	Antibiotic	51762-05-1		$C_{16}H_{19}N_3O_5S$
Cefsulodin Sodium	Antibiotic	69705-63-1		$C_{22}H_{21}N_4O_8S_2$
Cefsumide	Antibiotic	54818-11-0		$C_{17}H_{20}N_4O_6S_2$
Ceftazidime	Antibiotic	72558-82-8		$C_{22}H_{22}N_6O_7S_2$
Cefteram	Antibiotic	82547-58-8	Y	$C_{16}H_{17}N_9O_5S_2$
Ceftezole	Antibiotic	26973-24-0		$C_{13}H_{12}N_8O_4S_3$
Ceftibuten	Antibiotic	97519-39-6	Y	$C_{15}H_{14}N_4O_6S_2$
Ceftiofur	Antibiotic	80370-57-6		$C_{19}H_{17}N_5O_7S_3$
Ceftiolene	Antibiotic	77360-52-2		$C_{20}H_{18}N_8O_8S_3$
Ceftioxide	Antibiotic	69132-42-9		$C_{16}H_{17}N_5O_8S_2$
Ceftizoxime Alapivoxil	Antibiotic	135821-54-4		$C_{22}H_{28}N_6O_8S_2$
Ceftizoxime	Antibiotic	68401-81-0		$C_{13}H_{13}N_5O_5S_2$
Ceftobiprole	Antibiotic	209467-52-7		$C_{20}H_{22}N_8O_6S_2$
Ceftobiprole Medocaril	Antibiotic	376653-43-9		$C_{26}H_{26}N_8O_{11}S_2$
Ceftriaxone	Antibiotic	73384-59-5		$C_{18}H_{18}N_8O_7S_3$
Cefuracetime	Antibiotic	39685-31-9		$C_{17}H_{17}N_3O_8S$
Cefuroxime	Antibiotic	55268-75-2	Y	$C_{16}H_{16}N_4O_8S$
Cefuroxime Axetil	Antibiotic	64544-07-6	Y	$C_{20}H_{22}N_4O_{10}S$
Cefuroxime Pivoxetil	Antibiotic	100680-33-9		$C_{23}H_{28}N_4O_{11}S$
Cefuzonam	Antibiotic	82219-78-1		$C_{16}H_{15}N_7O_5S_4$
Celecoxib	Antiinflammatory	169590-42-5	Y	$C_{17}H_{14}F_3N_3O_2S$
Celgosivir	Antiviral	121104-96-9	Y	$C_{12}H_{21}NO_5$
Celiprolol	Antihypertensive	56980-93-9	Y	$C_{20}H_{33}N_3O_4$
Cemadotin	Antineoplastic	159776-69-9		$C_{35}H_{56}N_6O_5$
Cephacetrile Sodium	Antibiotic	10206-21-0		$C_{13}H_{13}N_3O_6S$
Cephalexin	Antibiotic	15686-71-2	Y	$C_{16}H_{17}N_3O_4S$
Cephaloglycin	Antibiotic	3577-01-3		$C_{18}H_{19}N_3O_6S$
Cephaloridine	Antibiotic	50-59-9		$C_{19}H_{17}N_3O_4S_2$
Cephalosporin C	Antibiotic	61-24-5		$C_{16}H_{21}N_3O_8S$
Cephalothin	Antibiotic	153-61-7		$C_{16}H_{16}$N2O6S2
Cephapirin	Antibiotic	21593-23-7		$C_{17}H_{17}N_3O_6S_2$
Cepharanthine	Antibiotic	481-49-2		$C_{37}H_{38}N_2O_6$
Cephradine	Antibiotic	38821-53-3	Y	$C_{16}H_{19}N_3O_4S$
Cericlamine	Antidepressant	112922-55-1		$C_{12}H_{17}Cl_2NO$
Cerivastatin	Antihyperlipidemic	145599-86-6		$C_{26}H_{34}FNO_5$
Ceronapril	Antihypertensive	111223-26-8		$C_{21}H_{33}N_2O_6P$
Cetaben Sodium	Antihyperlipidemic	55986-43-1		$C_{23}H_{39}NO_2$
Cetalkonium Chloride	Antibacterial	122-18-9		$C_{25}H_{46}ClN$
Cetamolol	Antihypertensive	34919-98-7		$C_{16}H_{26}N_2O_4$

Table S1. *Cont.*

Common Name	Indication	CAS Number	Oral	Molecular Formula
Cetefloxacin	Antibiotic	141725-88-4		$C_{20}H_{16}F_3N_3O_3$
Cethromycin	Antibacterial	205110-48-1	Y	$C_{42}H_{59}N_3O_{10}$
Cetiedil	Vasodilator	14176-10-4		$C_{20}H_{31}NO2S$
Cetilistat	Anticoagulant	282526-98-1	Y	$C_{25}H_{39}NO_3$
Cetirizine	Antihistaminic	83881-51-0	Y	$C_{21}H_{25}ClN_2O_3$
Cetocycline	Antibacterial	29144-42-1		$C_{22}H_{21}NO_7$
Cetohexazine	Unclassified	7007-92-3		$C_6H_8N_2O$
Cetophenicol	Antibacterial	735-52-4		$C_{13}H_{15}Cl_2NO_4$
Cetoxime	Antihistaminic	25394-78-9		$C_{15}H_{17}N_3O$
Cetraxate	Antiulcerative	34675-84-8	Y	$C_{17}H_{23}NO_4$
Cevimeline	Gastroprokinetic	107233-08-9	Y	$C_{10}H_{17}NOS$
Chaulmoogric Acid	Antibacterial	29106-32-9		$C_{18}H_{32}O_2$
Chaulmosulfone	Antibacterial	473-32-5		$C_{48}H_{76}N_2O_4S$
Chenodiol	Anticholelithogenic	474-25-9	Y	$C_{24H40O4}$
Chiniofon	Antiamebic	547-91-1		$C_9H_6INO_4S$
Chlophedianol	Antitussive	791-35-5		$C_{17}H_{20}ClNO$
Chloracyzine	Vasodilator	800-22-6		$C_{19}H_{21}ClN_2OS$
Chloral Hydrate	Sedative	302-17-0		$C_2H_3Cl_3O_2$
Chloralformamide	Sedative	515-82-2		$C_3H_4Cl_3NO_2$
Chloralose	Sedative	15879-93-3		$C_8H_{11}Cl_3O_6$
Chlorambucil	Antineoplastic	305-03-3	Y	$C_{14}H_{19}Cl_2NO_2$
Chloramine-T	Antibacterial	144-86-5		$C_7H_8ClNO_2S$
Chloramphenicol	Antibiotic	56-75-7		$C_{11}H_{12}Cl_2N_2O_5$
Chloramphenicol Palmitate	Antibiotic	530-43-8		$C_{27}H_{42}Cl_2N_2O_6$
Chloramphenicol Succinate	Antibiotic	3544-94-3		$C_{15}H_{16}Cl_2N_2O_8$
Chloranil	Antifungal	118-75-2		$C_6Cl_4O_2$
Chlorazanil	Antifungal	500-42-5		$C_9H_8ClN_5$
Chlorbenzoxamine	Antispasmodic	522-18-9		$C_{27}H_{31}ClN_2O$
Chlorbetamide	Antiamebic	97-27-8		$C_{11}H_{11}Cl_4NO_2$
Chlorcyclizine	Antihistaminic	82-93-9		$C_{18}H_{21}ClN_2$
Chlordantoin	Antifungal	5588-20-5		$C_{11}H_{17}Cl_3N_2O_2S$
Chlordiazepoxide	Anxiolytic	58-25-3	Y	$C_{16}H_{14}ClN_3O$
Chlordimorine	Unclassified	494-14-4		$C_{19}H_{22}ClNO_2$
Chlorhexadol	Sedative	3563-58-4		$C_8H_{15}Cl_3O_3$
Chlorindanol	Contraceptive	145-94-8		C_9H_9ClO
Chlorisondamine	Antihypertensive	69-27-2		$C_{14}H_{20}Cl_6N_2$
Chlormadinone Acetate	Progestogen	302-22-7	Y	$C_{23}H_{29}ClO_4$
Chlormezanone	Anxiolytic	80-77-3		$C_{11}H_{12}ClNO_3S$
Chlormidazole	Antifungal	3689-76-7		$C_{15}H_{13}ClN_2$
Chlornaphazine	Antineoplastic	494-03-1		$C_{14}H_{15}Cl_2N$
Chlorobutanol	Analgesic	57-15-8		$C_4H_7Cl_3O$
Chloroform	Anesthetic	67-66-3		$CHCl_3$
Chloroguanide	Antimalarial	500-92-5		$C_{11}H_{16}ClN_5$
Chlorophenothane	Ectoparasiticide	50-29-3		$C_{14}H_9Cl_5$
Chloroprednisone Acetate	Glucocorticoid	14066-79-6		$C_{23}H_{27}ClO_6$
Chloroprocaine	Anesthetic	133-16-4		$C_{13}H_{19}ClN_2O_2$
Chloropyramine	Antihistaminic	59-32-5		$C_{16}H_{20}ClN_3$
Chloroquine	Antimalarial	54-05-7	Y	$C_{18}H_{26}ClN_3$
Chloroserpidine	Antihypertensive	7008-24-4		$C_{32}H_{37}ClN_2O_8$
Chlorothen Citrate	Antihistaminic	148-65-2		$C_{14}H_{18}ClN_3S$
Chlorothiazide	Diuretic	58-94-6	Y	$C_7H_6ClN_3O_4S_2$
Chlorotrianisene	Estrogen	569-57-3		$C_{23H21ClO3}$
Chloroxine	Dermatologic	773-76-2		C9H5Cl2NO
Chlorphenesin Carbamate	Muscle Relaxant	886-74-8	Y	C_{10}H12ClNO4
Chlorpheniramine	Antihistaminic	132-22-9	Y	$C_{16}H_{19}ClN_2$
Chlorphenoctium Amsonate	Unclassified	7168-18-5		$C_{31}H_{41}Cl_2N_3O_7S_2$
Chlorphenoxamine	Antihistaminic	77-38-3		$C_{18}H_{22}ClNO$
Chlorphentermine	Anorexic	461-78-9		$C_{10}H_{14}ClN$
Chlorproethazine	Analgesic	84-01-5		$C_{19}H_{23}ClN_2S$
Chlorproguanil	Antimalarial	537-21-3		$C_{11}H_{15}Cl_2N_5$
Chlorpromazine	Antiemetic	50-53-3	Y	$C_{17}H_{19}ClN_2S$
Chlorpropamide	Antidiabetic	94-20-2	Y	$C_{10}H_{13}ClN_2O_3S$
Chlorprothixene	Antipsychotic	113-59-7		$C_{18}H_{18}ClNS$

Table S1. *Cont.*

Common Name	Indication	CAS Number	Oral	Molecular Formula
Chlorpyrifos	Ectoparasiticide	2921-88-2		$C_9H_{11}Cl_3NO_3PS$
Chlorquinaldol	Antibacterial	72-80-0		$C_{10}H_7Cl_2NO$
Chlortetracycline	Antibiotic	57-62-5		$C_{22}H_{23}ClN_2O_8$
Chlorthalidone	Diuretic	77-36-1		$C_{14}H_{11}ClN_2O_4S$
Chlorthenoxazine	Analgesic	132-89-8		$C_{10}H_{10}ClNO_2$
Chlorzoxazone	Muscle Relaxant	95-25-0		$C_7H_4ClNO_2$
Cholic Acid	Choleretic	81-25-4	Y	$C_{24}H_{40}O_5$
Choline Alfoscerate	Nootropic	28319-77-9	Y	$C_8H_{20}NO_6P$
Choline	Cholinergic	62-49-7		$C_5H_{14}NO+$
Choline Iodide Sebacate	Dermatologic	17140-07-7		$C_{20}H_{42}N_2O_4.2I$
Chromocarb	Capillary Protectant	4940-39-0		$C_{10}H_6O_4$
Chromonar	Vasodilator	804-10-4		$C_{20}H_{27}NO_5$
Ciadox	Antibacterial	65884-46-0		$C_{12}H_9N_5O_3$
Ciamexon	Immunosuppressant	75985-31-8		$C_{11}H_{13}N_3O$
Cianergoline	Antihypertensive	74627-35-3		$C_{19}H_{22}N_4O$
Cianidanol	Antidiarrheal	154-23-4		$C_{15}H_{14}O_6$
Cianopramine	Antidepressant	66834-24-0		$C_{20}H_{23}N_3$
Ciapilome	Unclassified	53131-74-1		$C_7H_6N_4O_2$
Cicaprost	Oxytocic	94079-80-8		$C_{22}H_{30}O_5$
Cicarperone	Antipsychotic	54063-29-5		$C_{20}H_{27}FN_2O_3$
Ciclactate	Unclassified	15145-14-9		$C_{12}H_{22}O_3$
Ciclafrine	Antihypotensive	55694-98-9		$C_{15}H_{21}NO_2$
Ciclazindol	Antidepressant	37751-39-6		$C_{17}H_{15}ClN_2O$
Ciclesonide	Glucocorticoid	126544-47-6		$C_{32}H_{44}O_7$
Cicletanine	Antihypertensive	89943-82-8	Y	$C_{14}H_{12}ClNO_2$
Ciclonicate	Vasodilator	53449-58-4		$C_{15}H_{21}NO_2$
Ciclonium Bromide	Antihypertensive	29546-59-6		$C_{22}H_{34}BrNO$
Ciclopirox	Antifungal	29342-05-0		$C_{12}H_{17}NO_2$
Ciclopirox Olamine	Antifungal	41621-49-2		$C_{14}H_{24}N_2O_3$
Ciclopramine	Antidepressant	33545-56-1		$C_{18}H_{20}N_2$
Cicloprofen	Antiinflammatory	36950-96-6		$C_{16}H_{14}O_2$
Cicloprolol	Antihypertensive	94651-09-9		$C_{18}H_{29}NO_4$
Ciclosidomine	Antihypertensive	66564-16-7		$C_{13}H_{20}N_4O_3$
Ciclotizolam	Anxiolytic	58765-21-2		$C_{20}H_{18}BrClN_4S$
Ciclotropium Bromide	Antibacterial	85166-20-7		$C_{24}H_{36}BrNO_2$
Cicloxilic Acid	Gastroprokinetic	57808-63-6		$C_{13}H_{16}O_3$
Cicloxolone	Antiviral	52247-86-6		$C_{38}H_{56}O_7$
Cicortonide	Glucocorticoid	19705-61-4		$C_{29}H_{37}ClFNO_7$
Cicrotoic Acid	Choleretic	25229-42-9		$C_{10}H_{16}O_2$
Cidofovir	Antiviral	113852-37-2		$C_8H_{14}N_3O_6P$
Cidoxepin	Antidepressant	3607-18-9		$C_{19}H_{21}NO$
Cifenline	Antiarrhythmic	53267-01-9	Y	$C_{18}H_{18}N_2$
Cifostodine	Unclassified	633-90-9		$C_9H_{12}N_3O_7P$
Ciglitazone	Antidiabetic	74772-77-3		$C_{18}H_{23}NO_3S$
Ciheptolane	Unclassified	34753-46-3		$C_{20}H_{23}NO_2$
Ciladopa	Antiparkinsonian	80109-27-9		$C_{21}H_{26}N_2O_4$
Cilansetron	Antiemetic	120635-74-7		$C_{20}H_{21}N_3O$
Cilastatin	Antibacterial	82009-34-5		$C_{16}H_{26}N_2O_5S$
Cilazapril	Antihypertensive	88768-40-5	Y	$C_{22}H_{31}N_3O_5$
Cilazaprilat	Antihypertensive	90139-06-3		$C_{20}H_{27}N_3O_5$
Cilengitide	Antineoplastic	188968-51-6		$C_{27}H_{40}N_8O_7$
Cilnidipine	Antihypertensive	132203-70-4	Y	$C_{27}H_{28}N_2O_7$
Cilobamine Mesylate	Antidepressant	69429-84-1		$C_{17}H_{23}Cl_2NO$
Cilobradine	Antianginal	147541-45-5		$C_{28}H_{38}N_2O_5$
Cilomilast	Bronchodilator	153259-65-5		$C_{20}H_{25}NO_4$
Cilostamide	Erectile Dysfunction	68550-75-4		$C_{20}H_{26}N_2O_3$
Cilostazol	Antithrombotic	73963-72-1	Y	$C_{20}H_{27}N_5O_2$
Ciltoprazine	Nootropic	54063-30-8		$C_{23}H_{29}ClN_4O_3$
Ciluprevir	Antiviral	300832-84-2		$C_{40}H_{50}N_6O_8S$
Cilutazoline	Antihypertensive	104902-08-1		$C_{14}H_{18}N_2O$
Cimaterol	Bronchodilator	54239-37-1		$C_{12}H_{17}N_3O$
Cimemoxin	Antipsychotic	3788-16-7		$C_7H_{16}N_2$
Cimepanol	Antianginal	29474-12-2		$C_{10}H_{20}O$

Table S1. *Cont.*

Common Name	Indication	CAS Number	Oral	Molecular Formula
Cimetidine	Antiulcerative	51481-61-9	Y	$C_{10}H_{16}N_6S$
Cimetropium Bromide	Antispasmodic	51598-60-8	Y	$C_{21}H_{28}BrNO_4$
Cimicoxib	Antiinflammatory	265114-23-6		$C_{16}H_{13}ClFN_3O_3S$
Cimoxatone	Antidepressant	73815-11-9		$C_{19}H_{18}N_2O_4$
Cinacalcet	Pituitary	226256-56-0	Y	$C_{22}H_{22}F_3N$
Cinalukast	Bronchodilator	128312-51-6		$C_{23}H_{28}N_2O_3S$
Cinametic Acid	Gastroprokinetic	35703-32-3		$C_{12}H_{14}O_5$
Cinamolol	Antihypotensive	39099-98-4		$C_{16}H_{23}NO_4$
Cinanserin	Antidepressant	1166-34-3		$C_{20}H_{24}N_2OS$
Cinaproxen	Antiinflammatory	89163-44-0		$C_{19}H_{21}NO_5S$
Cinchonidine	Antimalarial	485-71-2		$C_{19}H_{22}N_2O$
Cinchonine	Antimalarial	118-10-5	Y	$C_{19}H_{22}N_2O$
Cinchophen	Analgesic	132-60-5		$C_{16}H_{11}NO_2$
Cinecromen	Antithrombotic	62380-23-8		$C_{34}H_{41}N_3O_{10}$
Cinepaxadil	Unclassified	69118-25-8		$C_{29}H_{36}N_2O_9$
Cinepazet	Antianginal	23887-41-4		$C_{20}H_{28}N_2O_6$
Cinepazic Acid	Unclassified	54063-23-9		$C_{18}H_{24}N_2O_6$
Cinepazide	Vasodilator	23887-46-9		$C_{22}H_{31}N_3O_5$
Cinfenine	Antispasmodic	54141-87-6		$C_{25}H_{27}NO$
Cinfenoac	Antiinflammatory	66984-59-6		$C_{18}H_{14}O_6$
Cinflumide	Muscle Relaxant	64379-93-7		$C_{12}H_{12}FNO$
Cingestol	Progestogen	16915-71-2		$C_{20}H_{28}O$
Cinitapride	Gastroprokinetic	66564-14-5	Y	$C_{21}H_{30}N_4O_4$
Cinmetacin	Antiinflammatory	20168-99-4		$C_{21}H_{19}NO_4$
Cinnamaverine	Analgesic	1679-75-0		$C_{21}H_{25}NO_2$
Cinnamedrine	Antispasmodic	90-86-8		$C_{19}H_{23}NO$
Cinnarizine	Antihistaminic	298-57-7	Y	$C_{26}H_{28}N_2$
Cinnofuradione	Unclassified	477-80-5		$C_{20}H_{18}N_2O_3$
Cinoctramide	Anesthetic	28598-08-5		$C_{19}H_{27}NO_4$
Cinolazepam	Sedative	75696-02-5	Y	$C_{18}H_{13}ClFN_3O_2$
Cinoquidox	Antibacterial	64557-97-7		$C_{13}H_{12}N_4O3$
Cinoxacin	Antibiotic	28657-80-9		$C_{12}H_{10}N_2O_5$
Cinoxate	Dermatologic	104-28-9		$C_{14}H_{18}O_4$
Cinoxolone	Antiulcerative	31581-02-9		$C_{41}H56O5$
Cinoxopazide	Vasodilator	88053-05-8		$C_{20}H_{25}N_3O_4$
Cinperene	Antipsychotic	14796-24-8		$C_{25}H_{28}N_2O_2$
Cinprazole	Antiulcerative	51493-19-7		$C_{30}H_{32}N_4O$
Cinpropazide	Vasodilator	23887-47-0		$C_{21}H_{31}N_3O_5$
Cinromide	Anticonvulsant	58473-74-8		$C_{11}H_{12}BrNO$
Cintazone	Antiinflammatory	2056-56-6		$C_{22}H_{22}N_2O_2$
Cintriamide	Antipsychotic	5588-21-6		$C_{12}H_{15NO4}$
Cinuperone	Antipsychotic	82117-51-9		$C_{23}H_{24}FN_3O$
Cioteronel	Dermatologic	89672-11-7		$C_{16}H_{28}O_2$
Cipamfylline	Antiviral	132210-43-6		$C_{13}H_{17}N_5O_2$
Cipemastat	Antirheumatic	190648-49-8		$C_{22}H_{36}N_4O_5$
Ciprafamide	Antiamebic	35452-73-4		$C_{21}H_{24}N_2O$
Cipralisant	Nootropic	213027-19-1		$C_{14}H_{20}N_2$
Ciprazafone	Anticonvulsant	75616-03-4		$C_{19}H_{18}Cl_2N_2O_2$
Ciprefadol Succinate	Analgesic	59889-36-0		$C_{19}H_{27}NO$
Ciprocinonide	Steroid	58524-83-7		$C_{28}H_{34}F_2O_7$
Ciprofibrate	Antihyperlipidemic	52214-84-3	Y	$C_{13}H_{14}Cl_2O_3$
Ciprofloxacin	Antibiotic	85721-33-1	Y	$C_{17}H_{18}FN_3O_3$
Ciprokiren	Antihypertensive	143631-62-3		$C_{37}H_{55}N_5O_8S$
Ciprpride	Antiemetic	68475-40-1		$C_{17}H_{25}N_3O_4S$
Ciproquazone	Analgesic	33453-23-5		$C_{19}H_{18}N_2O_2$
Ciprostene Calcium	Anticoagulant	81845-44-5		$C_{22}H_{36}O_4$
Ciramadol	Analgesic	63269-31-8		$C_{15}H_{23}NO_2$
Cirazoline	Antidepressant	59939-16-1		$C_{13}H_{16}N_2O$
Cisapride	Gastroprokinetic	81098-60-4	Y	$C_{23}H_{29}ClFN_3O_4$
Cisconazole	Antifungal	104456-95-3		$C_{19}H_{15}F_3N_2OS$
Cismadinone	Progestogen	54063-31-9		$C_{21}H_{27}ClO_3$
Citalopram	Antidepressant	59729-33-8	Y	$C_{20}H_{21}FN_2O$
Citatepine	Antipsychotic	65509-66-2		$C_{20}H_{18}N_2S$

Table S1. *Cont.*

Common Name	Indication	CAS Number	Oral	Molecular Formula
Citenamide	Anticonvulsant	10423-37-7		$C_{16}H_{13}NO$
Citenazone	Antiviral	21512-15-2		$C_7H_6N_4S_2$
Citicoline Sodium	Nootropic	987-78-0	Y	$C_{14}H_{26}N_4O_{11}P_2$
Citiolone	Hepatic Protectant	1195-16-0		$C_6H_9NO_2S$
Citric Acid	Anticoagulant	77-92-9	Y	$C_6H_8O_7$
Cizolirtine	Antipsychotic	142155-43-9		$C_{15}H_{21}N_3O$
Cladribine	Antineoplastic	4291-63-8		$C_{10}H_{12}ClN_5O_3$
Clamidoxic Acid	Antibiotic	6170-69-0		$C_{15}H_{11}Cl_2NO_4$
Clamikalant	Antiarrhythmic	158751-64-5		$C_{19}H_{22}ClN_3O_5S_2$
Clamoxyquin	Antiamebic	2545-39-3		$C_{17}H_{24}ClN_3O$
Clanfenur	Antineoplastic	51213-99-1		$C_{16}H_{15}ClFN_3O_2$
Clanobutin	Choleretic	30544-61-7		$C_{18}H_{18}ClNO_4$
Clantifen	Antihistaminic	16562-98-4		$C_{11}H_7Cl_2NO_2S$
Clarithromycin	Antibiotic	81103-11-9	Y	$C_{38}H_{69}NO_{13}$
Clavulanic Acid	Antibiotic	58001-44-8		$C_8H_9NO_5$
Clazolam	Anxiolytic	7492-29-7		$C_{18}H_{17}ClN_2O$
Clazolimine	Diuretic	40828-44-2		$C_{10}H_{10}ClN_3O$
Clazosentan	Antihistaminic	180384-56-9		$C_{25}H_{23}N_9O6S$
Clazuril	Antibacterial	101831-36-1		$C_{17}H_{10}Cl_2N_4O_2$
Clebopride	Antiemetic	55905-53-8		$C_{20}H_{24}ClN_3O_2$
Clefamide	Antiamebic	3576-64-5		$C_{17}H_{16}Cl_2N_2O_5$
Clemastine	Antihistaminic	15686-51-8	Y	$C_{21}H_{26}ClNO$
Clemeprol	Choleretic	71827-56-0		$C_{17}H_{20}ClNO$
Clemizole	Antihistaminic	442-52-4		$C_{19}H_{20}ClN_3$
Clenbuterol	Bronchodilator	37148-27-9	Y	$C_{12}H_{18}Cl_2N_2O$
Clenpirin	Analgesic	27050-41-5		$C_{14}H_{18}Cl_2N_2$
Clentiazem Maleate	Antihypertensive	96125-53-0		$C_{22}H_{25}ClN_2O_4S$
Cletoquine	Antibiotic	4298-15-1		$C_{16}H_{22}ClN_3O$
Clevidipine	Antihypertensive	167221-71-8		$C_{21}H_{23}Cl_2NO_6$
Clevudine	Antiviral	163252-36-6	Y	$C_{10}H_{13}FN_2O_5$
Clibucaine	Analgesic	15302-10-0		$C_{15}H_{20}Cl_2N_2O$
Clidafidine	Sedative	33588-20-4		$C_9H_8Cl_2N_2O$
Clidanac	Antiinflammatory	34148-01-1		$C_{16}H_{19}ClO_2$
Clidinium	Antispasmodic	7020-55-5		$C_{22}H_{26}NO_3$
Climazolam	Anesthetic	59467-77-5		$C_{18}H_{13}Cl_2N_3$
Climbazole	Antifungal	38083-17-9		$C_{15}H_{17}ClN_2O_2$
Climiqualine	Anxiolytic	55150-67-9		$C_{18}H_{12}ClN_3$
Clinafloxacin	Antibiotic	105956-97-6		$C_{17}H_{17}ClFN_3O_3$
Clindamycin	Antibiotic	18323-44-9	Y	$C_{18}H_{33}ClN_2O_5S$
Clindamycin Palmitate	Antibiotic	36688-78-5		$C_{34}H_{63}ClN_2O_6S$
Clindamycin Phosphate	Antibiotic	24729-96-2		$C_{18H34ClN2O8PS}$
Clinofibrate	Antihyperlipidemic	30299-08-2	Y	$C_{28}H_{36}O_6$
Clinolamide	Diuretic	3207-50-9		$C_{24}H_{43}NO$
Clinprost	Anticoagulant	88931-51-5		$C_{22}H_{36}O_4$
Clioquinol	Antibacterial	130-26-7		C_9H_5ClINO
Clioxanide	Anthelminithic	14437-41-3		$C_{15}H_{10}ClI_2NO_3$
Cliprofen	Antiinflammatory	51022-75-4		$C_{14}H_{11}ClO_3S$
Cliropamine	Cardiotonic	109525-44-2		$C_{19}H_{25}NO_2$
Clobazam	Anxiolytic	22316-47-8	Y	$C_{16}H_{13}ClN_2O_2$
Clobenoside	Capillary Protectant	29899-95-4		$C_{25}H_{32}Cl_2O_6$
Clobenzepam	Antihistaminic	1159-93-9		$C_{17}H_{18}ClN_3O$
Clobenzorex	Anorexic	13364-32-4		$C_{16}H_{18}ClN$
Clobenztropine	Antihistaminic	5627-46-3		$C_{21}H_{24}ClNO$
Clobetasol Propionate	Glucocorticoid	25122-46-7		$C_{25}H_{32}ClFO_5$
Clobetasone Butyrate	Glucocorticoid	25122-57-0		$C_{26}H_{32}ClFO_5$
Clobutinol	Antitussive	14860-49-2		$C_{14}H_{22}ClNO$
Clobuzarit	Antirheumatic	22494-47-9		$C_{17}H_{17}ClO_3$
Clocanfamide	Antineoplastic	18966-32-0		$C_{18}H_{24}ClNO_2$
Clocapramine	Antipsychotic	47739-98-0		$C_{28}H_{37}ClN_4O$
Clociguanil	Antimalarial	3378-93-6		$C_{13}H_{16}Cl_2N_4O$
Clocinizine	Antihistaminic	298-55-5		$C_{26}H_{27}ClN_2$
Clocortolone Acetate	Glucocorticoid	4258-85-9		$C_{24}H_{30}ClFO_5$
Clocortolone Pivalate	Glucocorticoid	34097-16-0		$C_{27}H_{36}ClFO_5$

Table S1. *Cont.*

Common Name	Indication	CAS Number	Oral	Molecular Formula
Clocoumarol	Anticoagulant	35838-63-2		$C_{21}H_{21}ClO_3$
Clodacaine	Anesthetic	5626-25-5		$C_{16}H_{26}ClN_3O$
Clodanolene	Muscle Relaxant	14796-28-2		$C_{14}H_9Cl_2N_3O_3$
Clodazon	Antidepressant	4755-59-3		$C_{18}H_{20}ClN_3O$
Clodoxopone	Antiparkinsonian	71923-34-7		$C_{21}H_{21}ClN_2O_3$
Clofarabine	Antineoplastic	123318-82-1		$C_{10}H_{11}ClFN_5O_3$
Clofazimine	Antibacterial	2030-63-9		$C_{22}H_{27}Cl_2N_4$
Clofenamic Acid	Antiinflammatory	4295-55-0		$C_{13}H_9Cl_2NO_2$
Clofenamide	Diuretic	671-95-4		$C_6H_7ClN_2O_4S_2$
Clofenciclan	Unclassified	5632-52-0		$C_{18}H_{28}ClNO$
Clofenetamine	Decongestant	511-46-6		$C_{20}H_{26}ClNO$
Clofenoxyde	Unclassified	3030-53-3		$C_{16}H_{12}Cl_2O_3$
Clofeverine	Analgesic	54340-63-5		$C_{16}H_{16}ClNO_3$
Clofexamide	Antiinflammatory	1223-36-5		$C_{14}H_{21}ClN_2O_2$
Clofibrate	Antihyperlipidemic	637-07-0	Y	$C_{12}H_{15}ClO_3$
Clofibric Acid	Antihyperlipidemic	882-09-7		$C_{10}H_{11}ClO_3$
Clofibride	Antihyperlipidemic	26717-47-5		$C_{16}H_{22}ClNO_4$
Clofilium Phosphate	Antiarrhythmic	68379-03-3		$C_{21}H_{37}ClN.H_2O_4P$
Clofoctol	Antibiotic	37693-01-9		$C_{21}H_{26}Cl_2O$
Cloforex	Anorexic	14261-75-7		$C_{13}H_{18}ClNO_2$
Clofurac	Analgesic	60986-89-2		$C_{14}H_{15}ClO_2$
Clogestone Acetate	Progestogen	3044-32-4		$C_{25}H_{33}ClO_5$
Cloguanamil	Muscle Relaxant	21702-93-2		$C_9H_8ClN_5O$
Clomacran Phosphate	Antipsychotic	5310-55-4		$C_{18}H_{21}ClN_2$
Clomegestone Acetate	Progestogen	424-89-5		$C_{24}H_{31}ClO_4$
Clometacin	Analgesic	25803-14-9		$C_{19}H_{16}ClNO_4$
Clometherone	Pituitary	5591-27-5		$C_{22}H_{31}ClO_2$
Clomethiazole	Sedative	533-45-9		C_6H_8ClNS
Clometocillin	Antibiotic	1926-49-4		$C_{17}H_{18}Cl_2N_2O_5S$
Clomifenoxide	Antidepressant	97642-74-5		$C_{26}H_{28}ClNO_2$
Clominorex	Anorexic	3876-10-6		$C_9H_9ClN_2O$
Clomiphene	Estrogen	911-45-5	Y	$C_{26}H_{28}ClNO$
Clomipramine	Antidepressant	303-49-1	Y	$C_{19}H_{23}ClN_2$
Clomocycline	Antibiotic	1181-54-0		$C_{23}H_{25}ClN_2O_9$
Clomoxir	Antidiabetic	88431-47-4		$C_{14}H_{17}O_3Cl$
Clonazepam	Anticonvulsant	1622-61-3	Y	$C_{15}H_{10}ClN_3O_3$
Clonazoline	Decongestant	17692-28-3		$C_{14}H_{13}ClN_2$
Clonidine	Antihypertensive	4205-90-7	Y	$C_9H_9Cl_2N_3$
Clonitazene	Analgesic	3861-76-5		$C_{20}H_{23}ClN_4O_2$
Clonitrate	Vasodilator	2612-33-1		$C_3H_5ClN_2O_6$
Clonixeril	Analgesic	21829-22-1		$C_{16}H_{17}ClN_2O_4$
Clonixin	Analgesic	17737-65-4		$C_{13}H_{11}ClN_2O_2$
Clopamide	Antihypertensive	636-54-4	Y	$C_{14}H_{20}ClN_3O_3S$
Clopenthixol	Antipsychotic	982-24-1	Y	$C_{22}H_{25}ClN_2OS$
Cloperastine	Antitussive	3703-76-2		$C_{20}H_{24}ClNO$
Cloperidone	Sedative	4052-13-5		$C_{21}H_{23}ClN_4O_2$
Clopidogrel Sulfate	Anticoagulant	113665-84-2		$C_{16}H_{16}ClNO_2S$
Clopidol	Antibacterial	2971-90-6		$C_7H_7Cl_2NO$
Clopimozide	Antipsychotic	53179-12-7		$C_{28}H_{28}ClF_2N_3O$
Clopipazan Mesylate	Antipsychotic	60085-78-1		$C_{19}H_{18}ClNO$
Clopirac	Antiinflammatory	42779-82-8		$C_{14}H_{14}ClNO_2$
Cloponone	Respiratory Stimulant	85409-44-5		$C_{11}H_9Cl_4NO_2$
Cloprednol	Glucocorticoid	5251-34-3	Y	$C_{21}H_{25}ClO_5$
Cloprostenol	Prostaglandin	40665-92-7		$C_{22}H_{29}ClO_6$
Cloprothiazole	Antibiotic	6469-36-9		$C_7H_{10}ClNS$
Cloquinozine	Diuretic	5220-68-8		$C_{16}H_{22}ClN$
Cloracetadol	Analgesic	15687-05-5		$C_{10}H_{10}Cl_3NO_3$
Cloranolol	Antiarrhythmic	39563-28-5		$C_{13}H_{19}Cl_2NO_2$
Clorazepate	Anxiolytic	23887-31-2	Y	$C_{16}H_{11}ClN_2O_3$
Clorethate	Sedative	5634-37-7		$C_5H_4Cl_6O_3$
Clorexolone	Diuretic	2127-01-7		$C_{14}H_{17}ClN_2O_3S$
Clorgiline	Antidepressant	17780-72-2		$C_{13}H_{15}Cl_2NO$
Cloricromen	Antithrombotic	68206-94-0		$C_{20}H_{26}ClNO_5$

Table S1. *Cont.*

Common Name	Indication	CAS Number	Oral	Molecular Formula
Cloridarol	Vasodilator	3611-72-1		$C_{15}H_{11}ClO_2$
Clorindanic Acid	Antibiotic	153-43-5		$C_{10}H_9ClO_3$
Clorindione	Anticonvulsant	1146-99-2		$C_{15}H_9ClO_2$
Clormecaine	Analgesic	13930-34-2		$C_{11}H_{15}ClN_2O_2$
Cloroperone	Antipsychotic	61764-61-2		$C_{22}H_{23}ClFNO_2$
Cloroqualone	Sedative	25509-07-3		$C_{16}H_{12}Cl_2N_2O$
Clorotepine	Antipsychotic	13448-22-1		$C_{19}H_{21}ClN_2S$
Clorprenaline	Bronchodilator	3811-25-4		$C_{11}H_{16}ClNO$
Clorsulon	Anthelminithic	60200-06-8		$C_8H_8Cl_3N_3O_4S_2$
Clortermine	Anorexic	10389-73-8		$C_{10}H_{14}ClN$
Closantel	Anthelminithic	57808-65-8		$C_{22}H_{14}Cl_2I_2N_2O_2$
Closiramine Aceturate	Antihistaminic	47135-88-6		$C_{18}H_{21}ClN_2$
Clostebol	Steroid	1093-58-9		$C_{19}H_{27}ClO_2$
Clothiapine	Antipsychotic	2058-52-8		$C_{18}H_{18}ClN_3S$
Clothixamide Maleate	Antipsychotic	4177-58-6		$C_{24}H_{28}ClN_3OS$
Clotiazepam	Anxiolytic	33671-46-4	Y	$C_{16}H_{15}ClN_2OS$
Cloticasone Propionate	Antiinflammatory	80486-69-7		$C_{25}H_{31}ClF_2O_5S$
Clotioxone	Analgesic	1856-34-4		$C_9H_5Cl_3N_2O_2S$
Clotrimazole	Antifungal	23593-75-1	Y	$C_{22}H_{17}ClN_2$
Clovoxamine	Antidepressant	54739-19-4		$C_{14}H_{21}ClN_2O_2$
Cloxacepride	Antihistaminic	65569-29-1		$C_{22}H_{27}Cl_2N_3O_4$
Cloxacillin	Antibiotic	61-72-3	Y	$C_{19}H_{18}ClN_3O_5S$
Cloxazolam	Anxiolytic	24166-13-0		$C_{17}H_{14}Cl_2N_2O_2$
Cloxestradiol	Estrogen	54063-33-1		$C_{20}H_{25}Cl_3O_3$
Cloximate	Antiinflammatory	58832-68-1		$C_{14}H_{19}ClN_2O_3$
Cloxotestosterone	Androgen	53608-96-1		$C_{21}H_{29}Cl_3O_3$
Cloxypendyl	Antipsychotic	15311-77-0		$C_{20}H_{25}ClN_4OS$
Cloxyquin	Antibacterial	130-16-5		C_9H_6ClNO
Clozapine	Antipsychotic	5786-21-0	Y	$C_{18}H_{19}ClN_4$
Cocaine	Anesthetic	50-36-2		$C_{17}H_{21}NO_4$
Codeine	Antitussive	76-57-3	Y	$C_{18}H_{21}NO_3$
Codoxime	Antitussive	7125-76-0		$C_{20}H_{24}N_2O_5$
Co Fluampicil	Antibiotic	5250-39-5		$C_{19}H_{17}ClFN_3O_5S$
Cogazocine	Analgesic	57653-29-9		$C_{21}H_{32}NO$
Colchicine	Anticholelithogenic	64-86-8	Y	$C_{22}H_{25}NO_6$
Colestolone	Antihyperlipidemic	50673-97-7		$C_{27}H_{44}O_2$
Colfenamate	Antiinflammatory	30531-86-3		$C_{16}H_{13}F_3N_2O_3$
Colforsin	Bronchodilator	66575-29-9		$C_{22}H_{34}O_7$
Colfosceril Palmitate	Respiratory Stimulant	63-89-8		$C_{40}H_{80}NO_8P$
Colterol Mesylate	Bronchodilator	18866-78-9		$C_{12}H_{19}NO_3$
Coluracetam	Nootropic	135463-81-9		$C_{19}H_{23}N_3O_3$
Conessine Hydrobromide	Analgesic	546-06-5		$C_{24}H_{40}N_2$
Conivaptan	Cardiotonic	210101-16-9		$C_{32}H_{26}N_4O_2$
Conorphone	Analgesic	72060-05-0		$C_{23}H_{29}NO_3$
Cormethasone Acetate	Antiinflammatory	35135-67-2		$C_{24}H_{29}F_3O_6$
Cortisone Acetate	Glucocorticoid	50-04-4		C23H30O6
Cortisuzol	Glucocorticoid	50801-44-0		$C_{37}H_{40}N_2O_8S$
Cortivazol	Glucocorticoid	1110-40-3		$C_{32}H_{38}N_2O_5$
Cortodoxone	Antiinflammatory	152-58-9		$C_{21}H_{30}O_4$
Cotarnine Chloride	Hemostatic	10018-19-6		$C_{12}H_{14}NO_3.Cl$
Cotinine	Antidepressant	486-56-6		$C_{10}H_{12}N_2O$
Cotriptyline	Antidepressant	34662-67-4		$C_{21}H_{20}NO$
Coumaphos	Antineoplastic	56-72-4		$C_{14}H_{16}ClO_5PS$
Coumarin	Anticoagulant	91-64-5		$C_9H_6O_2$
Coumazoline	Anticholelithogenic	37681-00-8		$C_{14}H_{16}N_2O$
Coumetarol	Anticoagulant	4366-18-1		$C_{21}H_{16}O_7$
Creatinolfosfate	Bone Resorption Inhibitor	6903-79-3		$C_4H_{12}N_3O_4P$
Crilvastatin	Antihyperlipidemic	120551-59-9		$C_{14}H_{23}NO_3$
Crisnatol Mesylate	Antineoplastic	96389-68-3		$C_{23}H_{23}NO_2$
Crobenetine	Analgesic	221019-25-6		$C_{25}H_{33}NO_2$
Croconazole	Antifungal	77175-51-0		$C_{18}H_{15}ClN_2O$
Cromakalim	Antihypertensive	94470-67-4		$C_{16}H_{18}N_2O_3$
Cromitrile Sodium	Bronchodilator	53736-51-9		$C_{20}H_{15}N_5O_5$

Table S1. *Cont.*

Common Name	Indication	CAS Number	Oral	Molecular Formula
Cromoglicate Lisetil	Bronchodilator	110816-79-0		$C_{33}H_{36}N_2O_{12}$
Cromolyn	Bronchodilator	16110-51-3	Y	$C_{23}H_{16}O_{11}$
Cronidipine	Antihypertensive	113759-50-5		$C_{30}H_{32}ClN_3O_8$
Cropropamide	Analgesic	633-47-6		$C_{13}H_{24}N_2O_2$
Crotamiton	Dermatologic	483-63-6		$C_{13}H_{17}NO$
Crotetamide	Analgesic	6168-76-9		$C_{12}H_{22}N_2O_2$
Crotoniazide	Antibiotic	7007-96-7		$C_{10}H_{11}N_3O$
Crotoxyfos	Antineoplastic	7700-17-6		$C_{14}H_{19}O_6P$
Crufomate	Anthelminithic	299-86-5		$C_{12}H_{19}ClNO_3P$
Cyacetacide	Antibacterial	140-87-4		$C_3H_5N_3O$
Cyamemazine	Antipsychotic	3546-03-0	Y	$C_{19}H_{21}N_3S$
Cyclacillin	Antibiotic	3485-14-1		$C_{15}H_{23}N_3O_4S$
Cyclandelate	Vasodilator	456-59-7	Y	$C_{17}H_{24}O_3$
Cyclarbamate	Anxiolytic	5779-54-4		$C_{21}H_{24}N_2O_4$
Cyclazocine	Antidote	3572-80-3		$C_{18}H_{25}NO$
Cyclazodone	Antidepressant	14461-91-7		$C_{12}H_{12}N_2O_2$
Cyclexanone	Antitussive	15301-52-7		$C_{16}H_{25}NO_2$
Cyclindole	Antidepressant	32211-97-5		$C_{14}H_{18}N_2$
Cycliramine Maleate	Antihistaminic	47128-12-1		$C_{18}H_{19}ClN_2$
Cyclizine	Antiemetic	82-92-8		$C_{18}H_{22}N_2$
Cyclobarbital	Sedative	52-31-3		$C_{12}H_{16}N_2O_3$
Cyclobendazole	Anthelminithic	31431-43-3		$C_{13}H_{13}N_3O_3$
Cyclobenzaprine	Muscle Relaxant	303-53-7	Y	$C_{20}H_{21}N$
Cyclobutyrol	Choleretic	512-16-3		$C_{10}H_{18}O_3$
Cyclocumarol	Anticoagulant	518-20-7		$C_{20}H_{18}O_4$
Cyclofenil	Androgen	2624-43-3		$C_{23}H_{24}O_4$
Cycloguanil Pamoate	Antimalarial	516-21-2		$C_{11}H_{14}ClN_5$
Cycloheximide	Antineoplastic	66-81-9		$C_{15}H_{23}NO_4$
Cyclomenol	Antiarrhythmic	5591-47-9		$C_{14}H_{20}O$
Cyclomethycaine Sulfate	Analgesic	139-62-8		$C_{22}H_{33}NO_3$
Cyclopentamine	Decongestant	102-45-4		$C_9H_{19}N$
Cyclopenthiazide	Antihypertensive	742-20-1	Y	$C_{13}H_{18}ClN_3O_4S_2$
Cyclopentobarbital sodium	Sedative	76-68-6		$C_{12}H_{14}N_2O_3$
Cyclopentolate	Mydriatic	512-15-2		$C_{17}H_{25}NO_3$
Cyclophenazine	Antipsychotic	17692-26-1		$C_{23}H_{26}F_3N_3S$
Cyclophosphamide	Antineoplastic	50-18-0		$C_7H_{15}Cl_2N_2O_2P$
Cyclopregnol	Progestogen	465-53-2		$C_{21}H_{32}O_2$
Cyclopropane	Anesthetic	75-19-4		C_3H_6
Cycloserine	Antibacterial	68-41-7	Y	$C_3H_6N_2O_2$
Cyclothiazide	Diuretic	2259-96-3		$C_{14}H_{16}ClN3O4S2$
Cyclovalone	Choleretic	579-23-7		$C_{22}H_{22}O_5$
Cycrimine	Antispasmodic	77-39-4		$C_{19}H_{29}NO$
Cyheptamide	Anticonvulsant	7199-29-3		$C_{16}H_{15}NO$
Cyheptropine	Antihypotensive	602-40-4		$C_{24}H_{27}NO_2$
Cynarine	Choleretic	30964-13-7		$C_{25}H_{24}O_{12}$
Cypenamine	Antidepressant	15301-54-9		$C_{11}H_{15}N$
Cyprazepam	Sedative	15687-07-7		$C_{19}H_{18}ClN_3O$
Cyprenorphine	Analgesic	4406-22-8		$C_{26}H_{33}NO_4$
Cyprodenate	Antipsychotic	15585-86-1		$C_{13}H_{25}NO_2$
Cyproheptadine	Antihistaminic	129-03-3	Y	$C_{21}H_{21}N$
Cyprolidol	Antidepressant	4904-00-1		$C_{21}H_{19}NO$
Cyproquinate	Antibacterial	19485-08-6		$C_{20}H_{23}NO_5$
Cyproterone Acetate	Antineoplastic	427-51-0	Y	$C_{24}H_{29}ClO_4$
Cyproximide	Antipsychotic	66537-94-8		$C_{11}H_8ClNO_2$
Cyromazine	Antihistaminic	66215-27-8		$C_6H_{10}N_6$
Cysteamine	Antidote	60-23-1	Y	C_2H_7NS
Cytarabine	Antineoplastic	147-94-4		$C_9H_{13}N_3O_5$
Cyverine	Antispasmodic	4432-75-1		$C_{17}H_{33}N$
Dabelotine	Nootropic	118976-38-8		$C_{15}H_{22}N_2O_2$
Dabigatran	Antithrombotic	211914-51-1	Y	$C_{25}H_{25}N_7O_3$
Dabigatran Etexilate	Antithrombotic	211915-06-9	Y	$C_{34}H_{41}N_7O_5$
Dabuzalgron	Antispasmodic	219311-44-1		$C_{12}H_{16}ClN_3O_3S$
Dacarbazine	Antineoplastic	4342-03-4		$C_6H_{10}N_6O$

Table S1. *Cont.*

Common Name	Indication	CAS Number	Oral	Molecular Formula
Dacemazine	Antihistaminic	518-61-6		$C_{16}H_{16}N_2OS$
Dacinostat	Antineoplastic	404951-53-7		$C_{22}H_{25}N_3O_3$
Dacisteine	Unclassified	18725-37-6		$C_7H_{11}NO_4S$
Dacopafant	Antithrombotic	125372-33-0		$C_{12}H_{11}N_3OS$
Dacuronium Bromide	Muscle Relaxant	27115-86-2		$C_{33}H_{58}N_2O_3Br_2$
Dagapamil	Vasodilator	85247-76-3		$C_{36}H_{56}N_2O_4$
Daglutril	Antihypertensive	182821-27-8		$C_{31}H_{38}N_2O_6$
Dalbraminol	Cardiotonic	81528-80-5		$C_{17}H_{26}N_4O_2$
Dalcotidine	Antiulcerative	120958-90-9		$C_{18}H_{29}N_3O_2$
Daledalin Tosylate	Antidepressant	22136-27-2		$C_{19}H_{24}N_2$
Dalfopristin	Antibacterial	112362-50-2		$C_{34}H_{50}N_4O_9S$
Daltroban	Immunosuppressant	79094-20-5		$C_{16}H_{16}ClNO_4S$
Dalvastatin	Antihyperlipidemic	132100-55-1		$C_{24}H_{31}FO_3$
Dametralast	Vasodilator	71680-63-2		$C_6H_8N_6$
Damotepine	Antipsychotic	1469-07-4		$C_{17}H_{17}NS$
Danazol	Steroid	17230-88-5	Y	$C_{22}H_{27}NO_2$
Daniquidone	Antineoplastic	67199-66-0		$C_{15}H_{11}N_3O$
Danitracen	Antidepressant	31232-26-5		$C_{20}H_{21}NO$
Danofloxacin Mesylate	Antibacterial	112398-08-0		$C_{19}H_{20}FN_3O_3$
Danosteine	Antitussive	4938-00-5		$C_5H_8O_4S$
Danthron	Laxative	117-10-2		$C_{14}H_8O_4$
Dantrolene	Muscle Relaxant	7261-97-4	Y	$C_{14}H_{10}N_4O_5$
Dapabutan	Unclassified	6582-31-6		$C_{19}H_{40}N_2O_2$
Dapiprazole	Antiglaucoma	72822-12-9		$C_{19}H_{27}N_5$
Dapitant	Antiemetic	153438-49-4		$C_{37}H_{39}NO_4$
Dapivirine	Antiviral	244767-67-7		$C_{20}H_{19}N_5$
Dapoxetine	Antidepressant	119356-77-3	Y	$C_{21}H_{23}NO$
Dapsone	Antibiotic	80-08-0	Y	$C_{12}H_{12}N2O2S$
Darapladib	Antihyperlipidemic	356057-34-6	Y	$C_{36}H_{38}F_4N4O2S$
Darbufelone	Antiinflammatory	139226-28-1		$C_{18}H_{24}N_2O_2S$
Darenzepine	Antiulcerative	84629-61-8		$C_{21}H_{21}N_3O_2$
Darglitazone Sodium	Antidiabetic	141200-24-0		$C_{23}H_{20}N_2O_4S$
Darifenacin	Antispasmodic	133099-04-4	Y	$C_{28}H_{30}N_2O_2$
Darodipine	Antihypertensive	72803-02-2		$C_{19}H_{21}N_3O_5$
Darsidomine	Vasodilator	137500-42-6		$C_9H_{16}N_4O$
Darunavir	Antiviral	206361-99-1	Y	$C_{27}H_{37}N_3O_7S$
Darusentan	Anesthetic	171714-84-4		$C_{22}H_{22}N_2O_6$
Dasantafil	Erectile Dysfunction	569351-91-3		$C_{22}H_{28}BrN_5O_5$
Dasatinib	Antineoplastic	302962-49-8	Y	$C_{22}H_{26}ClN_7O_2S$
Datelliptium Chloride	Antineoplastic	105118-14-7		$C_{23}H_{28}N_3O.Cl$
Daunorubicin	Antineoplastic	20830-81-3		$C_{27}H_{29}NO_{10}$
Davasaicin	Analgesic	147497-64-1		$C_{22}H_{30}N_2O_3$
Daxalipram	Antidepressant	189940-24-7		$C_{14}H_{19}NO_4$
Dazadrol Maleate	Antidepressant	47029-84-5		$C_{15}H_{14}ClN_3O$
Dazepinil	Antidepressant	75991-50-3		$C_{17}H_{18}N_2$
Dazidamine	Antineoplastic	75522-73-5		$C_{19}H_{23}N_3S$
Dazmegrel	Anticoagulant	76894-77-4		$C_{16}H_{17}N_3O_2$
Dazolicine	Antiarrhythmic	61477-97-2		$C_{17}H_{24}ClN_3S$
Dazopride Fumarate	Gastroprokinetic	70181-03-2		$C_{15}H_{23}ClN_4O_2$
Dazoquinast	Bronchodilator	76002-75-0		$C_{11}H_7N_3O_2$
Dazoxiben	Antithrombotic	78218-09-4		$C_{12}H_{12}N_2O_3$
Deanol Aceglumate	Nootropic	1188-37-0		$C_7H_{11}NO_5$
Deboxamet	Antiulcerative	34024-41-4		$C_{12}H_{14}N_2O_3$
Debrisoquin Sulfate	Antihypertensive	1131-64-2		$C_{10}H_{13}N_3$
Debropol	Unclassified	24403-04-1		$C_3H_6BrNO_3$
Decamethonium Bromide	Muscle Relaxant	541-22-0		$C_{16}H_{38}Br_2N_2$
Decimemide	Anticonvulsant	14817-09-5		$C_{19}H_{31}NO_4$
Decitabine	Antineoplastic	2353-33-5		$C_8H_{12}N_4O_4$
Decitropine	Antihypotensive	1242-69-9		$C_{23}H_{25}NO$
Declenperone	Sedative	63388-37-4		$C_{22}H_{24}FN_3O_2$
Declopramide	Antiemetic	891-60-1		$C_{13}H_{20}ClN_3O$
Decloxizine	Sedative	3733-63-9		$C_{21}H_{28}N_2O_2$
Decominol	Antihypotensive	60812-35-3		$C_{13}H_{29}NO_2$

Table S1. *Cont.*

Common Name	Indication	CAS Number	Oral	Molecular Formula
Decoquinate	Antibacterial	18507-89-6		$C_{24}H_{35}NO_5$
Dectaflur	Antibacterial	36505-83-6		$C_{18}H_{37}N.FH$
Deditonium Bromide	Antispasmodic	2401-56-1		$C_{38}H_{66}Br_2N_2O_2$
Deferasirox	Antidote	201530-41-8	Y	$C_{21}H_{15}N_3O_4$
Deferiprone	Antidote	30652-11-0	Y	$C_7H_9NO_2$
Deferitrin	Antidote	239101-33-8		$C_{11}H_{11}NO_4S$
Deferoxamine Mesylate	Antidote	70-51-9		$C_{25}H_{48}N_6O_8$
Deflazacort	Antiinflammatory	14484-47-0	Y	$C_{25}H_{31}NO_6$
Defosfamide	Antineoplastic	3733-81-1		$C_9H_{20}Cl_3N_2O_3P$
Dehydrocholic Acid	Choleretic	81-23-2		$C_{24}H_{34}O_5$
Dehydroemetine	Antiamebic	4914-30-1		$C_{29}H_{38}N_2O_4$
Delanterone	Antineoplastic	63014-96-0		$C_{20}H_{28}O$
Delapril	Antihypertensive	83435-66-9	Y	$C_{26}H_{32}N_2O_5$
Delavirdine	Antiviral	136817-59-9	Y	$C_{22}H_{28}N_6O_3S$
Delequamine	Erectile Dysfunction	119905-05-4		$C_{18}H_{26}N_2O_3S$
Delergotrile	Antibiotic	59091-65-5		$C_{17}H_{19}N_3$
Delfantrine	Antimalarial	3436-11-1		$C_{14}H_{22}N_4O_3S$
Delfaprazine	Antipsychotic	117827-81-3		$C_{18}H_{22}N_2$
Delmadinone Acetate	Progestogen	13698-49-2		$C_{23}H_{27}ClO_4$
Delmetacin	Antiinflammatory	16401-80-2		$C_{18}H_{15}NO_3$
Delmopinol	Antibiotic	79874-76-3	Y	$C_{16}H_{33}NO_2$
Delorazepam	Sedative	2894-67-9		$C_{15}H_{10}Cl_2N_2O$
Deloxolone	Dermatologic	68635-50-7		$C_{34}H_{52}O_6$
Delprostenate	Prostaglandin	62524-99-6		$C_{23}H_{29}ClO_6$
Delucemine	Nootropic	186495-49-8		$C_{16}H_{17}F_2N$
Dembrexine	Antidepressant	83200-09-3		$C_{13}H_{17}Br_2NO_2$
Demecarium Bromide	Cholinergic	56-94-0		$C_{32}H_{52}Br_2N_4O_4$
Demeclocycline	Antibiotic	127-33-3	Y	$C_{21}H_{21}ClN_2O_8$
Demecolcine	Antineoplastic	477-30-5		$C_{21}H_{25}NO_5$
Demecycline	Antibacterial	987-02-0		$C_{21}H_{22}N_2O_8$
Demegestone	Progestogen	10116-22-0		$C_{21}H_{28}O_2$
Demelverine	Antispasmodic	13977-33-8		$C_{17}H_{21}N$
Demexiptiline	Antidepressant	24701-51-7		$C_{18}H_{18}N_2O$
Democonazole	Antifungal	70161-09-0		$C_{19}H_{15}Cl_3N_2O_2$
Demoxepam	Anxiolytic	963-39-3		$C_{15}H_{11}ClN_2O_2$
Demoxytocin	Oxytocic	113-78-0		$C_{43}H_{65}N_{11}O_{12}S_2$
Denaverine	Analgesic	3579-62-2		$C_{24}H_{33}NO_3$
Denbufylline	Bronchodilator	57076-71-8		$C_{16}H_{24}N_4O_3$
Denipride	Gastroprokinetic	106972-33-2		$C_{18}H_{26}N_4O_5$
Denopamine	Cardiotonic	71771-90-9		$C_{18}H_{23}NO_4$
Denotivir	Antiviral	51287-57-1		$C_{18}H_{14}ClN_3O_2S$
Denpidazone	Unclassified	42438-73-3		$C_{20}H_{20}N_2O_3$
Denufosol Tetrasodium	Decongestant	211448-85-0		$C_{18}H_{27}N_5O_{21}P_4$
Denzimol	Anticonvulsant	73931-96-1		$C_{19}H_{20}N_2O$
Deoxycholic Acid	Choleretic	83-44-3		$C_{24}H_{40}O_4$
Depramine	Antidepressant	303-54-8		$C_{19}H_{22}N_2$
Deprodone	Antidepressant	20423-99-8		$C_{21}H_{28}O_4$
Deprostil	Antiulcerative	33813-84-2		$C_{21}H_{28}O_4$
Deptropine Citrate	Antihistaminic	604-51-3		$C_{23}H_{27}NO$
Deracoxib	Antiinflammatory	169590-41-4		$C_{17}H_{14}F_3N_3O_3S$
Deramciclane	Vasodilator	120444-71-5		$C_{20}H_{31}NO$
Deriglidole	Antidiabetic	122830-14-2		$C_{16}H_{21}N_3$
Derpanicate	Estrogen	99518-29-3		$C_{46}H_{54}N_8O_{12}S_2$
Dersalazine	Antiinflammatory	188913-58-8	Y	$C_{35}H_{32}N_6O_4$
Desciclovir	Antiviral	84408-37-7		$C_8H_{11}N_5O_2$
Descinolone Acetonide	Glucocorticoid	2135-14-0		$C_{24}H_{31}FO_5$
Deserpidine	Antihypertensive	131-01-1		$C_{32}H_{38}N_2O_8$
Desflurane	Anesthetic	57041-67-5		$C_3H_2F_6O$
Desglugastrin	Antispasmodic	51987-65-6		$C_{49}H_{61}N_9O_{13}$
Desipramine	Antidepressant	50-47-5	Y	$C_{18}H_{22}N_2$
Deslanoside	Cardiotonic	17598-65-1		$C_{47}H_{74}O_{19}$
Desloratadine	Antihistaminic	100643-71-8	Y	$C_{19}H_{19}ClN_2$
Desmeninol	Unclassified	583-91-5		$C_5H_{10}O_3S$

Table S1. *Cont.*

Common Name	Indication	CAS Number	Oral	Molecular Formula
Desmethylmoramide	Analgesic	1767-88-0		$C_{24}H_{30}N_2O_2$
Desocriptine	Pituitary	66759-48-6		$C_{32}H_{45}N_5O_4$
Desogestrel	Progestogen	54024-22-5	Y	$C_{22}H_{30}O$
Desomorphine	Analgesic	427-00-9		$C_{17}H_{21}NO_2$
Desonide	Antiinflammatory	638-94-8		$C_{24}H_{32}O_6$
Desoximetasone	Antiinflammatory	382-67-2		$C_{22}H_{29}FO_4$
Desoxycorticosterone Acetate	Steroid	56-47-3		$C_{23}H_{32}O_4$
Desoxycorticosterone Pivalate	Steroid	808-48-0		$C_{26}H_{38}O_4$
Desvenlafaxine Succinate	Antidepressant	93413-62-8	Y	$C_{16}H_{25}NO_2$
Detanosal	Unclassified	23573-66-2		$C_{13}H_{19}NO_3$
Deterenol	Adrenergic	7376-66-1		$C_{11}H_{17}NO_2$
Detiviciclovir	Antiviral	220984-26-9		$C_9H_{13}N_5O_2$
Detomidine	Analgesic	76631-46-4		$C_{12}H_{14}N_2$
Detorubicin	Antineoplastic	66211-92-5		$C_{33}H_{39}NO_{14}$
Detrothyronine	Pituitary	5714-08-9		$C_{15}H_{12}I_3NO_4$
Deutolperisone	Muscle Relaxant	474641-19-5		$C_{17}H_{18}D_7NO$
Devapamil	Antianginal	92302-55-1		$C_{26}H_{36}N_2O_3$
Devazepide	Antispasmodic	103420-77-5		$C_{25}H_{20}N_4O_2$
Dexamethasone	Glucocorticoid	50-02-2	Y	$C_{22}H_{29}FO_5$
Dexamethasone Acefurate	Steroid	83880-70-0		$C_{29}H_{33}FO_8$
Dexamethasone Acetate	Glucocorticoid	1177-87-3		$C_{24}H_{31}FO_6$
Dexamethasone Beloxil	Antiinflammatory	150587-07-8		$C_{29}H_{35}FO_5$
Dexamethasone Dipropionate	Antiinflammatory	55541-30-5		$C_{28}H_{37}FO_7$
Dexamethasone Sodium Phosphate	Glucocorticoid	2392-39-4		$C_{22}H_{28}FNa_2O_8P$
Dexamisole	Antidepressant	14769-74-5		$C_{11}H_{12}N_2S$
Dexbrompheniramine Maleate	Antihistaminic	132-21-8	Y	$C_{16}H_{19}BrN_2$
Dexbudesonide	Glucocorticoid	51372-29-3		$C_{25}H_{34}O_6$
Dexchlorpheniramine Maleate	Antihistaminic	25523-97-1	Y	$C_{16}H_{19}ClN_2$
Dexclamol	Sedative	52340-25-7		$C_{24}H_{29}NO$
Dexecadotril	Antihypotensive	112573-72-5		$C_{21}H_{23}NO_4S$
Dexefaroxan	Antihypertensive	143249-88-1		$C_{13}H_{16}N_2O$
Dexetimide	Antiparkinsonian	21888-98-2		$C_{23}H_{26}N_2O_2$
Dexetozoline	Decongestant	77519-25-6		$C_{13}H_{20}N_2O_3S$
Dexfenfluramine	Anorexic	3239-44-9		$C_{12}H_{16}F_3N$
Dexfosfoserine	Antifungal	407-41-0		$C_3H_8NO_6P$
Dexibuprofen	Analgesic	51146-56-6		$C_{13}H_{18}O_2$
Deximafen	Antidepressant	42116-77-8		$C_{11}H_{13}N_3$
Dexindoprofen	Analgesic	53086-13-8		$C_{17}H_{15}NO_3$
Dexivacaine	Anesthetic	24358-84-7		$C_{15}H_{22}N_2O$
Dexketoprofen	Analgesic	22161-81-5	Y	$C_{16}H_{14}O_3$
Dexlansoprazole	Antiulcerative	138530-94-6	Y	$C_{16}H_{14}F_3N_3O_2S$
Dexlofexidine	Antihypertensive	81447-79-2		$C_{11}H_{12}Cl_2N_2O$
Dexloxiglumide	Antispasmodic	119817-90-2	Y	$C_{21}H_{30}Cl_2N_2O_5$
Dexmedetomidine	Anxiolytic	113775-47-6		$C_{13}H_{16}N_2$
Dexmethylphenidate	Nootropic	40431-64-9	Y	$C_{14}H_{19}NO_2$
Dexnafenodone	Sedative	92629-87-3		$C_{20}H_{23}NO$
Dexniguldipine	Antihypertensive	120054-86-6		$C_{36}H_{39}N_3O_6$
Dexoxadrol	Analgesic	4741-41-7		$C_{20}H_{23}NO_2$
Dexpanthenol	Cholinergic	81-13-0		$C_9H_{19}NO_4$
Dexpemedolac	Analgesic	114030-44-3		$C_{22}H_{23}NO_3$
Dexpropranolol	Antiarrhythmic	5051-22-9		$C_{16}H_{21}NO_2$
Dexproxibutene	Analgesic	47419-52-3		$C_{22}H_{27}NO_2$
Dexrazoxane	Antianginal	24584-09-6		$C_{11}H_{16}N_4O_4$
Dexsecoverine	Antispasmodic	90237-04-0		$C_{22}H_{35}NO_2$
Dexsotalol	Antiarrhythmic	30236-32-9		$C_{12}H_{20}N_2O_3S$
Dextilidine	Antihistaminic	32447-90-8		$C_{17}H_{23}NO_2$
Dextiopronin	Antidote	29335-92-0		$C_5H_9NO_3S$
Dextofisopam	Antispasmodic	82059-50-5		$C_{22}H_{26}N_2O_4$
Dextroamphetamine	Nootropic	51-64-9	Y	$C_9H_{13}N$
Dextrofemine	Antispasmodic	15687-08-8		$C_{18}H_{23}NO$
Dextromethorphan Hydrobromide	Antitussive	125-71-3	Y	$C_{18}H_{25}NO$
Dextromoramide	Analgesic	357-56-2	Y	$C_{25}H_{32}N_2O_2$
Dextrorphan	Analgesic	125-73-5		$C_{17}H_{23}NO$

Table S1. *Cont.*

Common Name	Indication	CAS Number	Oral	Molecular Formula
Dextrothyroxine	Antihyperlipidemic	51-49-0		$C_{15}H_{11}I_4NO_4$
Dexverapamil	Antianginal	38321-02-7		$C_{27}H_{38}N_2O_4$
Dezaguanine	Antineoplastic	41729-52-6		$C_6H_6N_4O$
Dezinamide	Anticonvulsant	91077-32-6		$C_{11}H_{11}F_3N_2O_2$
Dezocine	Analgesic	53648-55-8		$C_{16}H_{23}NO$
Diacerein	Antirheumatic	13739-02-1	Y	$C_{19}H_{12}O_8$
Diacetamate	Analgesic	2623-33-8		$C_{10}H_{11}NO_3$
Diacetolol	Antihypertensive	22568-64-5		$C_{16}H_{24}N_2O_4$
Diacetylmorphine	Analgesic	561-27-3		$C_{21}H_{23}NO_5$
Diacetylnalorphine	Analgesic	2748-74-5		$C_{23}H_{25}NO_5$
Diamfenetide	Anthelminthic	36141-82-9		$C_{20}H_{24}N_2O_5$
Diamocaine Cyclamate	Anesthetic	27112-37-4		$C_{25}H_{37}N_3O$
Diampromide	Analgesic	552-25-0		$C_{21}H_{28}N_2O$
Diamthazole	Antifungal	95-27-2		$C_{15}H_{23}N_3OS$
Dianicline	Antidepressant	292634-27-6		$C_{13}H_{16}N_2O$
Diapamide	Diuretic	3688-85-5		$C_9H_{11}ClN_2O_3S$
Diaplasinin	Anticoagulant	481631-45-2		$C_{32}H_{31}N_5O$
Diarbarone	Unclassified	1233-70-1		$C_{16}H_{20}N_2O_4$
Diathymosulfone	Antibacterial	5964-62-5		$C_{32}H_{34}N_4O_4S$
Diaveridine	Antibacterial	5355-16-8		$C_{13}H_{16}N_4O_2$
Diazepam	Anxiolytic	439-14-5	Y	$C_{16}H_{13}ClN_2O$
Diaziquone	Antineoplastic	57998-68-2		$C_{16}H_{20}N_4O_6$
Diazoxide	Antihypertensive	364-98-7	Y	$C_8H_7ClN_2O_2S$
Dibekacin	Antibiotic	34493-98-6		$C_{18}H_{37}N_5O_8$
Dibemethine	Antineoplastic	102-05-6		$C_{15}H_{17}N$
Dibenzepin	Antidepressant	4498-32-2		$C_{18}H_{21}N_3O$
Dibenzothiophene	Dermatologic	132-65-0		$C_{12}H_8S$
Dibrospidium Chloride	Antibiotic	86641-76-1		$C_{18}H_{32}Br_2Cl_2N_4O_2$
Dibucaine	Anesthetic	85-79-0		$C_{20}H_{29}N_3O_2$
Dibuprol	Choleretic	2216-77-5		$C_{11}H_{24}O_3$
Dibupyrone	Analgesic	772990-64-4		$C_{16}H_{23}N_3O_4S$
Dibusadol	Unclassified	24353-45-5		$C_{17}H_{26}N_2O_3$
Dibutoline Sulfate	Antispasmodic	532-49-0		$C_{15}H_{33}N_2O_2.1/2O_4S$
Dicarbine	Antidepressant	17411-19-7		$C_{13}H_{18}N_2$
Dicarfen	Antiinflammatory	15585-88-3		$C_{19}H_{24}N_2O_2$
Dichlorisone Acetate	Dermatologic	79-61-8		$C_{23}H_{28}Cl_2O_5$
Dichlormezanone	Anxiolytic	114676-16-3		$C_{11}H_{11}Cl_2NO_3S$
Dichlorophene	Anthelminithic	97-23-4		$C_{13}H_{10}Cl_2O_2$
Dichlorophenarsine	Antibacterial	455-83-4		$C_6H_6AsCl_2NO$
Dichloroxylenol	Anesthetic	133-53-9		$C_8H_8Cl_2O$
Dichlorphenamide	Antiglaucoma	120-97-8	Y	$C_6H_6Cl_2N_2O_4S_2$
Dichlorvos	Anthelminithic	62-73-7		$C_4H_7Cl_2O_4P$
Dicirenone	Antihypotensive	41020-79-5		$C_{26}H_{36}O_5$
Diclazuril	Antibacterial	101831-37-2	Y	$C_{17}H_9Cl_3N_4O_2$
Diclofenac	Antiinflammatory	15307-86-5	Y	$C_{14}H_{11}Cl_2NO_2$
Diclofensine	Antidepressant	67165-56-4		$C_{17}H_{17}Cl_2NO$
Diclofurime	Antihypertensive	64743-08-4		$C_{18}H_{22}Cl_2N_2O_3$
Diclometide	Antipsychotic	17243-49-1		$C_{14}H_{20}Cl_2N_2O2$
Diclonixin	Analgesic	17737-68-7		$C_{12}H_8Cl_2N_2O_2$
Dicloralurea	Antibacterial	116-52-9		$C_5H_6Cl_6N_2O_3$
Dicloxacillin	Antibiotic	3116-76-5	Y	$C_{19}H_{17}Cl_2N_3O_5S$
Dicolinium Iodide	Muscle Relaxant	382-82-1		$C_{16}H_{34}I_2N_2O_2$
Dicresulene	Unclassified	78480-14-5		$C_{15}H_{16}O_8S_2$
Dicumarol	Anticoagulant	66-76-2		$C_{19}H_{12}O_6$
Dicyclomine	Antispasmodic	77-19-0	Y	$C_{19}H_{35}NO_2$
Didanosine	Antiviral	69655-05-6	Y	$C_{10}H_{12}N_4O_3$
Didrovaltrate	Sedative	18296-45-2		$C_{22}H_{32}O_8$
Dieldrin	Dermatologic	60-57-1		$C_{12}H_8Cl_6O$
Dienestrol	Estrogen	84-17-3		$C_{18}H_{18}O_2$
Dienogest	Contraceptive	65928-58-7	Y	$C_{20}H_{25}NO_2$
Diethadione	Anticonvulsant	702-54-5		$C_8H_{13}NO_3$
Diethazine	Antiparkinsonian	60-91-3		$C_{18}H_{22}N_2S$
Diethyl Oxalate	Anorexic	95-92-1		$C_6H_{10}O_4$

Table S1. *Cont.*

Common Name	Indication	CAS Number	Oral	Molecular Formula
Diethylcarbamazine	Anthelminithic	90-89-1	Y	$C_{10}H_{21}N_3O$
Diethylpropion	Anorexic	90-84-6	Y	$C_{13}H_{19}NO$
Diethylstilbestrol	Antineoplastic	56-53-1	Y	$C_{18}H_{20}O_2$
Diethylstilbestrol Dipalmitate	Antineoplastic	6533-53-5		$C_{50}H_{80}O_4$
Diethylstilbestrol Dipropionate	Antineoplastic	130-80-3		$C_{24}H_{28}O_4$
Diethylthiambutene	Analgesic	86-14-6		$C_{16}H_{21}NS_2$
Dietifen	Antihistaminic	3686-78-0		$C_{21}H_{27}NO_2$
Difebarbamate	Sedative	15687-09-9		$C_{28}H_{42}N_4O_9$
Difemerine	Antispasmodic	80387-96-8		$C_{20}H_{25}NO_3$
Difemetorex	Anorexic	13862-07-2		$C_{20}H_{25}NO$
Difenamizole	Analgesic	20170-20-1		$C_{20}H_{22}N_4O$
Difencloxazine	Antidepressant	5617-26-5		$C_{19}H_{22}ClNO_2$
Difenoximide	Antispasmodic	47806-92-8		$C_{32}H_{31}N_3O_4$
Difenoxin	Antidiarrheal	28782-42-5	Y	$C_{28}H_{28}N_2O_2$
Difetarsone	Antiamebic	3639-19-8		$C_{14}H_{18}As_2N_2O_6$
Difeterol	Adrenergic	14587-50-9		$C_{25}H_{29}NO_2$
Diflomotecan	Antineoplastic	220997-97-7		$C_{21}H_{16}F_2N_2O_4$
Diflorasone Diacetate	Antiinflammatory	33564-31-7		$C_{26}H_{32F2O7}$
Difloxacin	Antibiotic	98106-17-3		$C_{21}H_{19F2N3O3}$
Difluanine	Nootropic	5522-39-4		$C_{28}H_{33}F_2N_3$
Diflucortolone	Glucocorticoid	2607-06-9		$C_{22}H_{28}F_2O_4$
Diflucortolone Pivalate	Antiinflammatory	15845-96-2		$C_{27}H_{36}F_2O_5$
Diflumidone Sodium	Antiinflammatory	22736-85-2		$C_{14}H_{11}F_2NO_3S$
Diflunisal	Antiinflammatory	22494-42-4	Y	$C_{13}H_8F_2O_3$
Difluprednate	Antiinflammatory	23674-86-4		$C_{27}H_{34}F_2O_7$
Diftalone	Antiinflammatory	21626-89-1		$C_{16}H_{12}N_2O_2$
Digitoxin	Cardiotonic	71-63-6		$C_{41}H_{64}O_{13}$
Digoxin	Cardiotonic	20830-75-5	Y	$C_{41}H_{64}O_{14}$
Dihexyverine	Anticholelithogenic	561-77-3		$C_{20}H_{35}NO_2$
Dihydralazine	Vasodilator	484-23-1		$C_8H_{10}N_6$
Dihydrocodeine Bitartrate	Analgesic	125-28-0	Y	$C_{18}H_{23}NO_3$
Dihydroergotamine Mesylate	Antimigraine	511-12-6		$C_{33}H_{37}N_5O_5$
Dihydrostreptomycin Sulfate	Antibacterial	128-46-1		$C_{21}H_{41}N_7O_{12}$
Dihydrotachysterol	Steroid	67-96-9		$C_{28}H_{46}O$
Diisopromine	Antidepressant	5966-41-6		$C_{21}H_{29}N$
Diisopropanolamine	Decongestant	110-97-4		$C_6H_{15}NO_2$
Diisopropylamine Dichloroacetate	Vasodilator	108-18-9		$C_6H_{15}N$
Dilazep	Vasodilator	35898-87-4		$C_{31}H_{44}N_2O_{10}$
Dilevalol	Antihypertensive	75659-07-3		$C_{19}H_{24N2O3}$
Dilmefone	Anticonvulsant	37398-31-5		$C_{16}H_{15}NO_3$
Diloxanide	Antiamebic	579-38-4		$C_9H_9Cl_2NO_2$
Diloxanide Furoate	Antiamebic	3736-81-0		$C_{14}H_{11}Cl_2NO_4$
Diltiazem	Antianginal	42399-41-7	Y	$C_{22}H_{26}N_2O_4S$
Dimabefylline	Bronchodilator	1703-48-6		$C_{16}H_{19}N_5O_2$
Dimecamine	Muscle Relaxant	3570-07-8		$C_{12}H_{23}N$
Dimecolonium Iodide	Antispasmodic	3425-97-6		$C_{14}H_{30}I_2$N2O2
Dimecrotic Acid	Choleretic	7706-67-4		$C_{12}H_{14}O_4$
Dimefadane	Analgesic	5581-40-8		$C_{17}H_{19}N$
Dimefline	Nootropic	1165-48-6		C_{20}H21NO3
Dimelazine	Antihypotensive	15302-12-2		$C_{19}H_{22}N_2S$
Dimemorfan	Antitussive	36309-01-0		$C_{18}H_{25}N$
Dimenhydrinate	Antiemetic	85-18-7	Y	$C_7H_7ClN_4O_2$
Dimenoxadol	Analgesic	509-78-4		$C_{20}H_{25}NO_3$
Dimepheptanol	Analgesic	545-90-4		$C_{21}H_{29}NO$
Dimepregnen	Progestogen	21208-26-4		$C_{23}H_{36}O_2$
Dimeprozan	Antipsychotic	6538-22-3		$C_{19}H_{21}NO_2$
Dimercaprol	Antidote	59-52-9		$C_3H_8OS_2$
Dimesna	Mucolytic	45127-11-5		$C_4H_{10}O_6S_4$
Dimesone	Unclassified	25092-07-3		$C_{23}H_{31}FO_4$
Dimestrol	Estrogen	130-79-0		$C_{20}H_{24}O_2$
Dimetacrine	Antidepressant	4757-55-5		$C_{20}H_{26}N_2$
Dimetamfetamine	Anorexic	17279-39-9		$C_{11}H_{17}N$
Dimethadione	Anticonvulsant	695-53-4		$C_5H_7NO_3$

Table S1. *Cont.*

Common Name	Indication	CAS Number	Oral	Molecular Formula
Dimethazan	Antidepressant	519-30-2		$C_{11}H_{17}N_5O_2$
Dimethindene Maleate	Antihistaminic	5636-83-9		$C_{20}H_{24}N_2$
Dimethisoquin	Anesthetic	86-80-6		$C_{17}H_{24}N_2O$
Dimethisterone	Progestogen	79-64-1		$C_{23}H_{32}O_2$
Dimetholizine	Unclassified	7008-00-6		$C_{15}H_{24}N_2O_2$
Dimethoxanate	Antitussive	477-93-0		$C_{19}H_{22}N_2O_3S$
1,3-Dimethylbutyl Salicylate	Antiinflammatory	22490-72-8	Y	$C_{15}H_{33}N_3$
Dimethyl Fumarate	Immunomodulator	624-49-7	Y	$C_6H_8O_4$
Dimethyl Sulfoxide	Urologic	67-68-5		C_2H_6OS
Dimethylaminoethyl Reserpilinate Di	Antihypertensive	5585-67-1		$C_{26}H_{35}N_3O_5$
Dimethylthiambutene	Analgesic	524-84-5		$C_{14}H_{17}NS_2$
Dimethyltubocurarinium Chloride	Neuromuscular Blocking Agent	33335-58-9Y		$C_{40}H_{48}Cl_2N_2O_6$
Dimetipirium Bromide	Antihypertensive	51047-24-6		$C_{23}H_{30}BrNO_3$
Dimetofrine	Antihypotensive	22950-29-4		$C_{11}H_{17}NO_4$
Dimetridazole	Antiprotozoal	551-92-8		$C_5H_7N_3O_2$
Diminazene	Analgesic	536-71-0		$C_{14}H_{15}N_7$
Dimiracetam	Anticonvulsant	126100-97-8		$C_6H_8N_2O_2$
Dimorpholamine	Nootropic	119-48-2		$C_{20}H_{38}N_4O_4$
Dimoxamine	Nootropic	52842-59-8		$C_{13}H_{21}NO_2$
Dimoxaprost	Prostaglandin	90243-98-4		$C_{21}H_{34}O_6$
Dimpylate	Ectoparasiticide	333-41-5		$C_{12}H_{21}N_2O_3PS$
Dinaline	Bronchodilator	58338-59-3		$C_{13}H_{13}N_3O$
Dinazafone	Anticonvulsant	71119-12-5		$C_{20}H_{21}ClN_2O_2$
Diniprofylline	Bronchodilator	17692-30-7		$C_{22}H_{20}N_6O_6$
Dinitolmide	Antibacterial	148-01-6		$C_8H_7N_3O_5$
Dinoprost	Oxytocic	551-11-1	Y	$C_{20}H_{34}O_5$
Dinoprostone	Oxytocic	363-24-6		$C_{20}H_{32}O_5$
Dinsed	Antibacterial	96-62-8		$C_{14}H_{14}N_4O_8S2$
Diosmin	Capillary Protectant	520-27-4	Y	$C_{28}H_{32}O_{15}$
Dioxadilol	Antihypertensive	80743-08-4		$C_{16}H_{25}NO_4$
Dioxadrol	Antidepressant	6495-46-1		$C_{20}H23NO2$
Dioxamate	Antihistaminic	3567-40-6		$C_{15}H_{29}NO_4$
Dioxaphetyl Butyrate	Antispasmodic	467-86-7		$C_{22}H_{27}NO_3$
Dioxethedrin	Bronchodilator	497-75-6		$C_{11}H_{17}NO_3$
Dioxifedrine	Adrenergic	10329-60-9		$C_{10}H_{15}NO_3$
Dioxybenzone	Dermatologic	131-53-3		$C_{14}H_{12}O_4$
Dioxyline Phosphate	Cardiotonic	147-27-3		$C_{22}H_{25}NO_4$
Dipenine Bromide	Antispasmodic	2001-81-2		$C_{20}H_{38}BrNO_2$
Diperodon	Anesthetic	101-08-6		$C_{22}H_{27}N_3O_4$
Diphenhydramine	Antihistaminic	58-73-1	Y	$C_{17}H_{21}NO$
Diphenidol	Antiemetic	972-02-1	Y	$C_{21}H_{27}NO$
Diphenoxylate /Atropine Sulfate	Antispasmodic	915-30-0		$C_{30}H_{32}N_2O_2$
Diphenylpyraline	Antihistaminic	147-20-6		$C_{19}H_{23}NO$
Diphoxazide	Antibacterial	511-41-1		$C_{17}H_{18}N_2O_3$
Dipipanone	Analgesic	467-83-4	Y	$C_{24}H_{31}NO$
Dipiproverine	Antispasmodic	117-30-6		$C_{20}H_{30}N_2O_2$
Dipivefrin	Mydriatic	52365-63-6	Y	$C_{19}H_{29}NO_5$
Diprafenone	Antiarrhythmic	81447-80-5		$C_{23}H_{31}NO_3$
Diprenorphine	Antidote	14357-78-9		$C_{26}H_{35}NO_4$
Diprobutine	Antiparkinsonian	61822-36-4		$C_{10}H_{23}N$
Diprofene	Antihypertensive	5835-72-3		$C_{22}H_{29}NOS$
Diproleandomycin	Antibiotic	14289-25-9		$C_{41}H_{69}NO_{14}$
Diproqualone	Sedative	36518-02-2		$C_{12}H_{14}N_2O_3$
Diproteverine	Antihypertensive	69373-95-1		$C_{26}H_{35}NO_4$
Diproxadol	Analgesic	52042-24-7		$C_{12}H_{14}ClNO_4$
Dipyridamole	Antithrombotic	58-32-2	Y	$C_{24}H_{40}N_8O_4$
Dipyrithione	Antibacterial	3696-28-4		$C_{10}H_8N_2O_2S_2$
Dipyrocetyl	Analgesic	486-79-3		$C_{11}H_{10}O_6$
Dipyrone	Analgesic	50567-35-6		$C_{13}H_{17}N_3O_4S$
Diquafosol Tetrasodium	Mucolytic	59985-21-6		$C_{18}H_{26}N_4O_{23}P_4$
Dirithromycin	Antibiotic	62013-04-1	Y	$C_{42}H78N2O14$

Table S1. *Cont.*

Common Name	Indication	CAS Number	Oral	Molecular Formula
Dirlotapide	Antihyperlipidemic	481658-94-0		$C_{40}H_{33}F_3N_4O_3$
Disermolide	Antiparkinsonian	127943-53-7		$C_{33}H_{55}NO8$
Disobutamide	Antiarrhythmic	68284-69-5		$C_{23}H_{38}ClN_3O$
Disogluside	Unclassified	14144-06-0		$C_{33}H_{52}O_8$
Disopyramide	Antiarrhythmic	3737-09-5	Y	$C_{21}H_{29}N_3O$
Disoxaril	Antiviral	87495-31-6		$C_{20}H_{26}N_2O_3$
Distigmine Bromide	Muscle Relaxant	15876-67-2	Y	$C_{22}H_{32}Br_2N_4O_4$
Disufenton Sodium	Nootropic	168021-77-0		$C_{11}H_{15}NO_7S_2$
Disulergine	Urologic	59032-40-5		$C_{17}H_{24}N_4O_2S$
Disulfiram	Alcohol Deterrant	97-77-8	Y	$C_{10}H_{20}N_2S_4$
Disuprazole	Antiulcerative	99499-40-8		$C_{16}H_{17}N_3OS_2$
Ditazole	Antiinflammatory	18471-20-0		$C_{19}H_{20}N_2O_3$
Ditekiren	Antihypertensive	103336-05-6		$C_{50}H_{75}N_9O_8$
Ditercalinium Chloride	Antihypertensive	74517-42-3		$C_{46}H_{50}Cl_2N_6O_2$
Dithiazanine Iodide	Anthelminithic	514-73-8		$C_{23}H_{23}IN_2S_2$
Ditiocarb	Immunomodulator	147-84-2		$C_5H_{11}NS_2$
Ditiomustine	Antineoplastic	82599-22-2		$C_{10}H_{18}Cl_2N_6O_4S_2$
Ditolamide	Antibiotic	723-42-2		$C_{13}H_{21}NO_2S$
Ditophal	Antibiotic	584-69-0		$C_{12}H_{14}O_2S_2$
Divabuterol	Bronchodilator	54592-27-7		$C_{22}H_{35}NO_5$
Divaplon	Anxiolytic	90808-12-1		$C_{17}H_{17}N_3O_2$
Dixanthogen	Ectoparasiticide	502-55-6		$C_6H_{10}O_2S_4$
Dizatrifone	Nootropic	92257-40-4		$C_{21}H_{21}N_3O_3$
Dizocilpine Maleate	Nootropic	77086-21-6		$C_{16}H_{15}N$
Dobupride	Gastroprokinetic	106707-51-1		$C_{20}H_{30}ClN_3O_4$
Dobutamine	Cardiotonic	34368-04-2		$C_{18}H_{23}NO_3$
Docarpamine	Cardiotonic	74639-40-0		$C_{21}H_{30}N_2O_8S$
Docebenone	Antiinflammatory	80809-81-0		$C_{21}H_{26}O_3$
Docetaxel	Antineoplastic	114977-28-5		$C_{43}H_{53}NO_{14}$
Doconazole	Antifungal	59831-63-9		$C_{26}H_{22}Cl_2N_2O_3$
Doconexent	Unclassified	6217-54-5		$C_{22}H_{32}O_2$
Docusate Sodium	Laxative	10041-19-7		$C_{20}H_{38}O_7S$
Dodeclonium Bromide	Antibiotic	15687-13-5		$C_{22}H_{39}BrClNO$
Dodicin	Antibacterial	6843-97-6		$C_{18}H_{39}N_3O_2$
Dofamium Chloride	Antihistaminic	54063-35-3		$C_{25}H_{44}ClN_3O_2$
Dofequidar	Antineoplastic	129716-58-1		$C_{30}H_{31}N_3O_3$
Dofetilide	Antiarrhythmic	115256-11-6	Y	$C_{19}H_{27}N_3O_5S_2$
Dolasetron	Antiemetic	115956-12-2	Y	$C_{19}H_{20}N_2O_3$
Doliracetam	Anticonvulsant	84901-45-1		$C_{16}H_{14}N_2O_2$
Domazoline Fumarate	Anticholelithogenic	6043-01-2		$C_{14}H_{20}N_2O_2$
Domiodol	Mucolytic	61869-07-6		$C_5H_9IO_3$
Domiphen Bromide	Antibacterial	538-71-6		$C_{22}H_{40}BrNO$
Domipizone	Bronchodilator	95355-10-5		$C_{13}H_{16}N_2O_4$
Domitroban	Antithrombotic	112966-96-8		$C_{20}H_{27}NO_4S$
Domoprednate	Dermatologic	66877-67-6		$C_{26}H_{36}O_5$
Domoxin	Antidepressant	61-74-5		$C_{16}H_{18}N_2O_2$
Domperidone	Antiemetic	57808-66-9	Y	$C_{22}H_{24}ClN_5O_2$
Donepezil	Nootropic	120014-06-4	Y	$C_{24}H_{29}NO_3$
Donetidine	Antihistaminic	99248-32-5		$C_{20}H_{25}N_5O_3S$
Donitriptan	Antimigraine	170912-52-4		$C_{23}H_{25}N_5O_2$
Dopamantine	Antiparkinsonian	39907-68-1		$C_{19}H_{25}NO_3$
Dopamine	Cardiotonic	51-61-6	Y	$C_8H_{11}NO_2$
Dopexamine	Cardiotonic	86197-47-9		$C_{22}H_{32}N_2O_2$
Dopropidil	Antianginal	79700-61-1		$C_{20}H_{35}NO_2$
Doqualast	Antihistaminic	64019-03-0		$C_{13}H_8N_2O_3$
Doramapimod	Antirheumatic	285983-48-4		$C_{31}H_{37}N_5O_3$
Doramectin	Antibacterial	117704-25-3		$C_{50}H_{74}O_{14}$
Doranidazole	Antiprotozoal	149838-23-3		$C_8H_{13}N_3O_6$
Dorastine	Antihistaminic	21228-13-7		$C_{20}H_{22}ClN_3$
Doreptide	Antiparkinsonian	90104-48-6		$C_{17}H_{24}N_4O_3$
Doretinel	Dermatologic	104561-36-6		$C_{24}H_{30}O_2$
Doripenem	Antibiotic	148016-81-3		$C_{15}H_{24}N_4O_6S_2$
Dorzolamide	Antiglaucoma	120279-96-1		$C_{10}H_{16}N_2O_4S_3$

Table S1. *Cont.*

Common Name	Indication	CAS Number	Oral	Molecular Formula
Dosergoside	Antiinflammatory	87178-42-5		$C_{34}H_{53}N_3O_3$
Dotarizine	Vasodilator	84625-59-2		$C_{29}H_{34}N_2O_2$
Dotefonium Bromide	Antispasmodic	26058-50-4		$C_{20}H_{27}BrN_2O_2S$
Dothiepin	Antidepressant	113-53-1	Y	$C_{19}H_{21}NS$
Doxaminol	Cardiotonic	55286-56-1		$C_{26}H_{29}NO_3$
Doxapram	Respiratory Stimulant	309-29-5		$C_{24}H_{30}N_2O_2$
Doxaprost	Bronchodilator	51953-95-8		$C_{21}H_{36}O_4$
Doxazosin	Antihypertensive	74191-85-8	Y	$C_{23}H_{25}N_5O_5$
Doxefazepam	Sedative	40762-15-0		$C_{17}H_{14}ClFN_2O_3$
Doxenitoin	Anticonvulsant	3254-93-1		$C_{15}H_{14}N_2O$
Doxepin	Antidepressant	1668-19-5	Y	$C_{19}H_{21}NO$
Doxercalciferol	Thyroid	54573-75-0	Y	$C_{28}H_{44}O_2$
Doxibetasol	Glucocorticoid	1879-77-2		$C_{22}H_{29}FO_4$
Doxifluridine	Antineoplastic	3094-09-5	Y	$C_9H_{11FN2O5}$
Doxofylline	Bronchodilator	69975-86-6	Y	$C_{11}H_{14N4O4}$
Doxorubicin	Antineoplastic	23214-92-8	Y	$C_{27}H_{29NO11}$
Doxpicomine	Analgesic	62904-71-6		$C_{12}H18N2O2$
Doxycycline	Antibiotic	564-25-0	Y	$C_{22}H_{24}N_2O_8$
Doxylamine	Antihistaminic	469-21-6	Y	$C_{17}H_{22}N_2O$
Draflazine	Cardiotonic	120770-34-5		$C_{30}H_{33}Cl_2F_2N_5O_2$
Dramedilol	Antihypertensive	76953-65-6		$C_{20}H_{29}N_5O_4$
Draquinolol	Antihyperlipidemic	67793-71-9		$C_{24}H_{30}N_2O_4$
Drazidox	Antibacterial	27314-77-8		$C_{10}H_{10}N_4O_3$
Dribendazole	Anthelminithic	63667-16-3		$C_{15}H_{19}N_3O2S$
Drinidene	Analgesic	53394-92-6		$C_{10}H_9NO$
Drobuline	Antiarrhythmic	58473-73-7		$C_{19}H_{25}NO$
Drocarbil	Anthelminthic	63-75-2		C_8H_{13NO2}
Drocinonide	Antiinflammatory	2355-59-1		$C_{24}H_{35}FO_6$
Droclidinium Bromide	Antihypertensive	29125-56-2		$C_{22}H_{32}BrNO_3$
Drofenine	Antispasmodic	1679-76-1		$C_{20}H_{31}NO_2$
Droloxifene	Antineoplastic	82413-20-5		$C_{26}H_{29}NO_2$
Drometrizole	Dermatologic	2440-22-4		$C_{13}H_{11}N_3O$
Dromostanolone Propionate	Antineoplastic	521-12-0		$C_{23}H_{36}O_3$
Dronabinol	Antiemetic	1972-08-3	Y	$C_{21}H_{30}O_2$
Dronedarone	Antiarrhythmic	141626-36-0	Y	$C_{31}H_{44}N_2O_5S$
Dropempine	Unclassified	34703-49-6		$C_{10}H_{19}N$
Droperidol	Antipsychotic	548-73-2		$C_{22}H_{22}FN_3O_2$
Droprenilamine	Vasodilator	57653-27-7		$C_{24}H_{33}N$
Dropropizine	Antitussive	17692-31-8		$C_{13}H_{20}N_2O_2$
Drospirenone	Contraceptive	67392-87-4	Y	$C_{24}H_{30}O_3$
Drotaverine	Antispasmodic	14009-24-6	Y	$C_{24}H_{31}NO_4$
Drotebanol	Antitussive	3176-03-2	Y	$C_{19}H_{27}NO_4$
Droxacin Sodium	Antibacterial	35067-47-1		$C_{14}H_{13}NO_4$
Droxicainide	Antiarrhythmic	78289-26-6		$C_{16}H_{24}N_2O_2$
Droxicam	Antiinflammatory	90101-16-9		$C_{16}H_{11}N_3O_5S$
Droxidopa	Antiparkinsonian	23651-95-8	Y	$C_9H_{11}NO_5$
Droxinavir	Antiviral	159910-86-8		$C_{29}H_{51}N_5O_4$
Droxypropine	Mydriatic	15599-26-5		$C_{18}H_{27}NO_3$
Duazomycin	Antineoplastic	2508-89-6		$C_8H_{15}N_3O_4$
Dulofibrate	Antihyperlipidemic	61887-16-9		$C_{16}H_{14}Cl_2O_3$
Duloxetine	Antidepressant	116539-59-4	Y	$C_{18}H_{19}NOS$
Dulozafone	Anticonvulsant	75616-02-3		$C_{20}H_{22}Cl_2N_2O_4$
Duometacin	Antiinflammatory	25771-23-7		$C_{20}H_{19}NO_5$
Duoperone Fumarate	Nootropic	62030-88-0		$C_{28}H_{26}F_4N_2OS$
Dupracetam	Nootropic	59776-90-8		$C_{12}H_{18}N_4O_4$
Dyclonine	Anesthetic	586-60-7	Y	$C_{18}H_{27}NO_2$
Dutasteride	Steroid	164656-23-9	Y	$C_{27}H_{30}F_6N_2O_2$
Dydrogesterone	Progestogen	152-62-5	Y	$C_{21}H_{28}O_2$
Dymanthine	Anthelminithic	124-28-7	Y	$C_{20}H_{43}N$
Dyphylline	Bronchodilator	479-18-5		$C_{10}H_{14}N_4O_4$
Ebalzotan	Antidepressant	149494-37-1	Y	$C_{19}H_{30}N_2O_2$
Ebastine	Antihistaminic	90729-43-4	Y	$C_{32}H_{39}NO_2$
Eberconazole	Antifungal	128326-82-9		$C_{18}H_{14}Cl_2N_2$

Table S1. *Cont.*

Common Name	Indication	CAS Number	Oral	Molecular Formula
Ebiratide	Steroid	105250-86-0		$C_{48}H_{73}N_{11}O_{10}S$
Ebrotidine	Antiulcerative	100981-43-9	Y	$C_{14}H_{17}BrN_6O_2S_3$
Ebselen	Nootropic	60940-34-3	Y	$C_{13}H_9NOSe$
Ecabapide	Antiulcerative	104775-36-2		$C_{20}H_{25}N_3O_4$
Ecabet	Antiulcerative	33159-27-2	Y	$C_{20}H_{28}O_5S$
Ecadotril	Antihypotensive	112573-73-6		$C_{21}H_{23}NO_4S$
Ecalcidene	Dermatologic	150337-94-3		$C_{29}H_{45}NO_3$
Ecamsule	Dermatologic	92761-26-7		$C_{28}H_{34}O_8S_2$
Ecastolol	Antianginal	77695-52-4		$C_{26}H_{33}N_3O_6$
Ecenofloxacin	Antibiotic	162301-05-5		$C_{19}H_{21}FN_4O_3$
Echothiophate Iodide	Cholinergic	513-10-0		$C_9H_{23}INO_3PS$
Ecipramidil	Antihypotensive	64552-16-5		$C_{29}H_{33}NO_5$
Eclanamine Maleate	Antidepressant	67450-44-6		$C_{16}H_{22}Cl_2N_2O$
Eclazolast	Antihistaminic	80263-73-6		$C_{12}H_{12}ClNO_4$
Ecomustine	Antineoplastic	98383-18-7		$C_{10}H_{18}ClN_3O_6$
Econazole	Antifungal	27220-47-9	Y	$C_{18}H_{15}Cl_3N_2O$
Ecopipam	Sedative	112108-01-7	Y	$C_{19}H_{20}ClNO$
Ecopladib	Analgesic	381683-92-7		$C_{39}H_{33}Cl_3N_2O_5S$
Ecraprost	Anticoagulant	136892-64-3		$C_{28}H_{48}O_6$
Ectylurea	Sedative	95-04-5		$C_7H_{12}N_2O_2$
Edaglitazone	Antidiabetic	213411-83-7		$C_{24}H_{20}N_2O_4S_2$
Edaravone	Nootropic	89-25-8		$C_{10}H_{10}N_2O$
Edatrexate	Antineoplastic	80576-83-6		$C_{22}H_{25}N_7O_5$
Edelfosine	Antineoplastic	70641-51-9	Y	$C_{27}H_{58}NO_6P$
Edetate	Antidote	60-00-4		$C_{10}H_{16}N_2O_8$
Edifolone Acetate	Antiarrhythmic	90733-40-7		$C_{24}H_{37}NO_4$
Edogestrone	Progestogen	809-01-8		$C_{26}H_{38}O_5$
Edonentan	Cardiotonic	210891-04-6		$C_{28}H_{32}N_4O_5S$
Edotecarin	Antineoplastic	174402-32-5		$C_{29}H_{28}N_4O_{11}$
Edoxudine	Antiviral	15176-29-1		$C_{11}H_{16}N_2O_5$
Edronocaine	Analgesic	190258-12-9		$C_{15}H_{25}NO_2$
Edrophonium	Cholinergic	312-48-1		$C_{10}H_{16}NO+$
Efaproxiral	Antihypotensive	131179-95-8		$C_{20}H_{23}NO_4$
Efaroxan	Antihypertensive	89197-32-0	Y	$C_{13}H_{16}N_2O$
Efavirenz	Antiviral	154598-52-4	Y	$C_{14}H_9ClF_3NO_2$
Efegatran Sulfate	Antithrombotic	105806-65-3		$C_{21}H_{32}N_6O_3$
Efepristin	Antibiotic	57206-54-9		$C_{44}H_{52}N_8O_{10}$
Efetozole	Antidepressant	90408-21-2		$C_{12}H_{14}N_2$
Efipladib	Antihypotensive	381683-94-9		$C_{40}H_{35}$Cl3N2O4S
Efletirizine	Antihistaminic	150756-35-7		$C_{21}H_{24}F_2N_2O_3$
Eflornithine	Antineoplastic	70052-12-9	Y	$C_6H_{12}F_2N_2O_2$
Efloxate	Vasodilator	119-41-5		$C_{19}H_{16}O_5$
Eflucimibe	Antihyperlipidemic	202340-45-2		$C_{29}H_{43}NO_2S$
Eflumast	Antihistaminic	70977-46-7		$C_{10}H_8FN_5O_3$
Efonidipine	Antihypertensive	111011-63-3	Y	$C_{34}H_{38}N_3O_7P$
Eglumetad	Anxiolytic	176199-48-7		$C_8H_{11}NO_4$
Egualen	Antiulcerative	99287-30-6	Y	$C_{15}H_{18}O_3S$
Elacridar	Antineoplastic	143664-11-3		$C_{34}H_{33}N_3O_5$
Elantrine	Anticholelithogenic	1232-85-5		$C_{20}H_{24}N_2$
Elanzepine	Anticonvulsant	6196-08-3		$C_{19}H_{21}ClN_2$
Elarofiban	Anticoagulant	198958-88-2		$C_{22}H_{32}N_4O_4$
Elbanizine	Antihistaminic	110629-41-9		$C_{26}H_{31}N_5O_2$
Eldacimibe	Antihyperlipidemic	141993-70-6		$C_{39}H_{58}N_2O_5$
Eletriptan	Antimigraine	143322-58-1	Y	$C_{22}H_{26}N_2O_2S$
Elfazepam	Anxiolytic	52042-01-0		$C_{19}H_{18}ClFN_2O_3S$
Elgodipine	Antianginal	119413-55-7		$C_{29}H_{33}FN_2O_6$
Elinafide	Antineoplastic	162706-37-8		$C_{31}H_{28}N_4O_4$
Eliprodil	Antiparkinsonian	119431-25-3		$C_{20}H_{23}ClFNO$
Elisartan	Antihypertensive	149968-26-3		$C_{27}H_{29}ClN_6O_5$
Ellagic Acid	Hemostatic	476-66-4		$C_{14}H_6O_8$
Elliptinium Acetate	Antineoplastic	58337-35-2		$C_{20}H_{20}N_2O_3$
Elmustine	Antineoplastic	60784-46-5		$C_5H_{10}ClN_3O_3$
Elnadipine	Antiparkinsonian	103946-15-2		$C_{19}H_{19}Cl_2N_3O_3$

Table S1. *Cont.*

Common Name	Indication	CAS Number	Oral	Molecular Formula
Elomotecan	Antineoplastic	220998-10-7		$C_{29}H_{32}ClN_3O_4$
Elopiprazole	Antipsychotic	115464-77-2		$C_{23}H_{22}FN_3O$
Elsamitrucin	Antineoplastic	97068-30-9		$C_{33}H_{35}NO_{13}$
Eltanolone	Anesthetic	128-20-1		$C_{21}H_{34}O_2$
Eltenac	Antiinflammatory	72895-88-6		$C_{12}H_9Cl_2NO_2S$
Eltoprazine	Antipsychotic	98224-03-4	Y	$C_{12}H_{16}N_2O_2$
Eltrombopag Olamine	Hematinic	496775-61-2	Y	$C_{25}H_{22}N_4O_4$
Elucaine	Anticholelithogenic	25314-87-8		$C_{19}H_{23}NO_2$
Elvucitabine	Antiviral	181785-84-2		$C_9H_{10}FN_3O_3$
Elzasonan Citrate	Antidepressant	361343-19-3		$C_{22}H_{23}Cl_2N_3OS$
Elziverine	Antispasmodic	95520-81-3		$C_{32}H_{37}N_3O_5$
Emakalim	Antihypertensive	129729-66-4		$C_{17}H_{16}N_2O_3$
Emapunil	Unclassified	226954-04-7		$C_{23}H_{23}N_5O_2$
Embeconazole	Antifungal	329744-44-7		$C_{27}H_{25}F_3N_4O_3S$
Embramine	Antihistaminic	3565-72-8		$C_{18}H_{22}BrNO$
Embusartan	Antihypertensive	156001-18-2		$C_{25}H_{24}FN_5O_3$
Embutramide	Anesthetic	15687-14-6		$C_{17}H_{27}NO_3$
Emedastine	Antihistaminic	87233-61-2	Y	$C_{17}H_{26}N_4O$
Emetine	Antiamebic	483-18-1		$C_{29}H_{40}N_2O_4$
Emideltide	Unclassified	62568-57-4		$C_{35}H_{48}N_{10}O_{15}$
Emiglitate	Antidiabetic	80879-63-6		$C_{17}H_{25}NO_7$
Emilium Tosylate	Antiarrhythmic	30716-01-9		$C_{19}H_{27}NO_4S$
Emitefur	Antineoplastic	110690-43-2		$C_{28}H_{19}FN_4O_8$
Emivirine	Antiviral	149950-60-7		$C_{17}H_{22}N_2O_3$
Emopamil	Vasodilator	78370-13-5		$C_{23}H_{30}N_2$
Emorfazone	Antiinflammatory	38957-41-4		$C_{11}H_{17}N_3O_3$
Emylcamate	Anxiolytic	78-28-4		$C_7H_{15}NO_2$
Enadoline	Analgesic	124378-77-4		$C_{24}H_{32}N_2O_3$
Enalapril	Antihypertensive	75847-73-3	Y	$C_{20}H_{28}N_2O_5$
Enalaprilat	Antihypertensive	76420-72-9		$C_{18}H_{24}N_2O_5$
Enalkiren	Antihypertensive	113082-98-7		$C_{35}H_{56}N_6O_6$
Enazadrem Phosphate	Dermatologic	107361-33-1		$C_{18}H_{25}N_3O$
Enbucrilate	Dermatologic	6606-65-1		$C_8H_{11}NO_2$
Encainide	Antiarrhythmic	66778-36-7		$C_{22}H_{28}N_2O_2$
Enciprazine	Anxiolytic	68576-86-3		$C_{23}H_{32}N_2O_6$
Enclomiphene	Steroid	15690-57-0	Y	$C_{26}H_{28}ClNO$
Encyprate	Antidepressant	2521-01-9		$C_{13}H_{17}NO_2$
Endixaprine	Antineoplastic	93181-85-2		$C_{15}H_{15}Cl_2N_3O$
Endobenzyline Bromide	Mydriatic	52080-56-5		$C_{20}H_{28}NO_3.Br$
Endomide	Immunomodulator	4582-18-7		$C_{17}H_{28}N_2O_2$
Endralazine Mesylate	Antihypertensive	39715-02-1		$C_{14}H_{15}N_5O$
Endrysone	Antiinflammatory	35100-44-8		$C_{22}H_{30}O_3$
Enecadin	Nootropic	259525-01-4		$C_{21}H_{28}FN_3O$
Enefexine	Antidepressant	67765-04-2		$C_{13}H_{19}N$
Enestebol	Androgen	2320-86-7		$C_{20}H_{28}O_3$
Enfenamic Acid	Antiinflammatory	23049-93-6		$C_{15}H_{15}NO_2$
Enflurane	Anesthetic	13838-16-9		C3H2ClF5O
Englitazone Sodium	Antidiabetic	109229-58-5		$C_{20}H_{19}NO_3S$
Eniclobrate	Antihyperlipidemic	81126-88-7		$C_{24}H_{24}ClNO_3$
Enilconazole	Antifungal	35554-44-0		C_{14}H14Cl2N2O
Enilospirone	Anxiolytic	59798-73-1		$C_{15}H_{18}ClNO_3$
Eniluracil	Antineoplastic	59989-18-3	Y	$C_6H_4N_2O_2$
Eniporide	Cardiotonic	176644-21-6		$C_{14}H_{16}N_4O_3S$
Enisoprost	Antiulcerative	81026-63-3		$C_{22}H_{36}O_5$
Enocitabine	Antineoplastic	55726-47-1		$C_{31}H_{55}N_3O_6$
Enofelast	Bronchodilator	125722-16-9		$C_{16}H_{15}FO$
Enolicam Sodium	Antiinflammatory	59755-82-7		$C_{17}H_{12}Cl_3NO_4S$
Enoxacin	Antibiotic	74011-58-8	Y	$C_{15}H_{17}FN_4O_3$
Enoxamast	Antibacterial	74604-76-5		$C_{13}H_{10}N_2O_5S$
Enoximone	Cardiotonic	77671-31-9		$C_{12}H_{12}$N2O2S
Enoxolone	Antiinflammatory	471-53-4		$C_{30}H_{46}O_4$
Enpiprazole	Anxiolytic	31729-24-5		$C_{16}H_{21}ClN_4$
Enpiroline Phosphate	Antimalarial	66364-73-6		$C_{19}H_{18}F_6N_2O$

Table S1. *Cont.*

Common Name	Indication	CAS Number	Oral	Molecular Formula
Enprazepine	Anticonvulsant	47206-15-5		$C_{20}H_{24}N_2$
Enprofylline	Bronchodilator	41078-02-8		$C_8H_{10}N_4O_2$
Enpromate	Antineoplastic	10087-89-5		$C_{22}H_{23}NO_2$
Enprostil	Antiulcerative	73121-56-9		$C_{23}H_{28}O_6$
Enrasentan	Anesthetic	167256-08-8		$C_{29}H_{30}O_8$
Enrofloxacin	Antibiotic	93106-60-6		$C_{19}H_{22}FN_3O_3$
Ensaculin	Nootropic	155773-59-4		$C_{26}H_{32}N_2O_5$
Ensulizole	Dermatologic	27503-81-7		$C_{13}H_{10}N_2O_3S$
Entacapone	Antiparkinsonian	130929-57-6	Y	$C_{14}H_{15}N_3O_5$
Entecavir	Antiviral	142217-69-4	Y	$C_{12}H_{15}N_5O_3$
Enviomycin	Antibiotic	33103-22-9		$C_{25}H_{43}N_{13}O_{10}$
Enviradene	Antiviral	80883-55-2		$C_{19}H_{21}N_3O_{2S}$
Enviroxime	Antiviral	72301-79-2		$C_{17}H_{18}N_4O_3S$
Enzacamene	Dermatologic	36861-47-9		$C_{18}H_{22}O$
Enzastaurin	Antineoplastic	170364-57-5	Y	$C_{32}H_{29}N_5O_2$
Epalrestat	Antidiabetic	82159-09-9	Y	$C_{15}H_{13}NO_3S_2$
Epanolol	Antianginal	86880-51-5		$C_{20}H_{23}N_3O_4$
Eperezolid	Antibacterial	165800-04-4		$C_{18}H_{23}FN_4O_5$
Eperisone	Muscle Relaxant	64840-90-0		$C_{17}H_{25}NO$
Epervudine	Antiviral	60136-25-6		$C_{12}H_{18}N_2O_5$
Ephedrine	Bronchodilator	299-42-3	Y	$C_{10}H_{15}NO$
Ephedrine Camphorate	Antihypotensive	124-83-4		$C_{10}H_{16}O_4$
Epicainide	Antiarrhythmic	66304-03-8		$C_{21}H_{26}N_2O_2$
Epicillin	Antibiotic	26774-90-3		$C_{16}H_{21}N_3O_4S$
Epicriptine	Pituitary	88660-47-3		$C_{32}H_{43}N_5O_5$
Epiestriol	Steroid	547-81-9		$C_{18}H_{24}O_3$
Epimestrol	Pituitary	7004-98-0		$C_{19}H_{26}O_3$
Epinastine	Antihistaminic	80012-43-7	Y	$C_{16}H_{15}N_3$
Epinephrine	Bronchodilator	51-43-4		$C_9H_{13}NO_3$
Epinephryl Borate	Adrenergic	5579-16-8		$C_9H_{12}BNO_4$
Epipropidine	Antineoplastic	5696-17-3		$C_{16}H_{28}N_2O_2$
Epirizole	Analgesic	18694-40-1		$C_{11}H_{14}N_4O_2$
Epiroprim	Antibiotic	73090-70-7		$C_{19}H_{23}N_5O_2$
Epirubicin	Antineoplastic	56420-45-2		$C_{27}H_{29}NO_{11}$
Epitetracycline	Antibacterial	79-85-6		$C_{22}H_{24}N_2O_8$
Epithiazide	Antihypertensive	1764-85-8		$C_{10}H_{11}ClF_3N_3O_4S_3$
Epitiostanol	Antineoplastic	2363-58-8		$C_{19}H_{30}OS$
Eplerenone	Antihypertensive	107724-20-9	Y	$C_{24}H_{30}O_6$
Eplivanserin	Anxiolytic	130579-75-8		$C_{19}H_{21}FN_2O_2$
Epoprostenol	Antithrombotic	35121-78-9		$C_{20}H_{32}O_5$
Epostane	Oxytocic	80471-63-2		$C_{22}H_{31}NO_3$
Eprazinone	Antitussive	10402-90-1		$C_{24}H_{32}N_2O_2$
Epristeride	Steroid	119169-78-7		$C_{25}H_{37}NO_3$
Eprobemide	Antiparkinsonian	87940-60-1		$C_{14}H_{19}ClN_2O_2$
Eprodisate Disodium	Nootropic	21668-77-9	Y	$C_3H_8O_6S_2$
Eprosartan	Antihypertensive	133040-01-4	Y	$C_{23}H_{24}N_2O_4S$
Eprovafen	Antidepressant	101335-99-3		$C_{18}H_{22}O_2S$
Eproxindine	Antiarrhythmic	85793-29-9		$C_{23}H_{29}N_3O_3$
Eprozinol	Bronchodilator	32665-36-4		$C_{22}H_{30}N_2O_2$
Epsiprantel	Anthelminithic	98123-83-2		$C_{20}H_{26}N_2O_2$
Eptaloprost	Prostaglandin	90693-76-8		$C_{24}H_{34}O_5$
Eptapirone	Anxiolytic	179756-58-2		$C_{16}H_{23}N_7O_2$
Eptastigmine	Cholinergic	101246-68-8		$C_{21}H_{33}N_3O_2$
Eptazocine	Analgesic	72522-13-5		$C_{15}H_{21}NO$
Eptifibatide	Antithrombotic	188627-80-7		$C_{35}H_{49}N_{11}O_9S_2$
Equilin	Estrogen	474-86-2		$C_{18}H_{20}O_2$
Erbulozole	Antineoplastic	124784-31-2		$C_{24}H_{27}N_3O_5S$
Erdosteine	Mucolytic	84611-23-4	Y	$C_8H_{11}NO_4S_2$
Ergonovine	Antihypertensive	60-79-7	Y	$C_{19}H_{23}N_3O_2$
Ergotamine	Analgesic	113-15-5		$C_{33}H_{35}N_5O_5$
Ericolol	Antihypertensive	85320-67-8		$C_{18}H_{24}ClNO_3$
Erizepine	Anticonvulsant	96645-87-3		$C_{20}H_{22}N_2$
Erlotinib	Antineoplastic	183321-74-6	Y	$C_{22}H_{23}N_3O_4$

Table S1. *Cont.*

Common Name	Indication	CAS Number	Oral	Molecular Formula
Erocainide	Antiarrhythmic	85750-38-5		$C_{22}H_{33}ClN_2O$
Ersentilide	Antiarrhythmic	125279-79-0		$C_{21}H_{26}N_4O_5S$
Ertapenem	Antibacterial	153832-46-3		$C_{22}H_{25}N_3O_7S$
Ertiprotafib	Antidiabetic	251303-04-5		$C_{31}H_{27}BrO_3S$
Erythrityl Tetranitrate	Vasodilator	7297-25-8		$C_4H_6N_4O_{12}$
Erythromycin	Antibiotic	114-07-8	Y	$C_{37}H_{67}NO_{13}$
Erythromycin Acistrate	Antibiotic	992-69-8		$C_{39}H_{69}NO_{14}$
Erythromycin Ethylsuccinate	Antibiotic	1264-62-6	Y	$C_{43}H_{75}NO_{16}$
Erythromycin Propionate	Antibiotic	134-36-1		$C_{40}H_{71}NO_{14}$
Erythromycin Salnacedin	Antibiotic	87573-04-4		$C_{12}H_{13}NO_5S$
Esafloxacin	Antibiotic	129672-09-9		$C_{15}H_{17}FN_4O_3$
Esaprazole	Antiulcerative	64204-55-3		$C_{12}H_{23}N_3O$
Esatenolol	Adrenergic	93379-54-5		$C_{14}H_{22}N_2O_3$
Escitalopram Oxalate	Antidepressant	128196-01-0	Y	$C_{20}H_{21}FN_2O$
Esculamine	Antiinflammatory	2908-75-0		$C_{15}H_{19}NO_6$
Esculin	Dermatologic	531-75-9		$C_{15}H_{16}O_9$
Eseridine	Cholinergic	25573-43-7		$C_{15}H_{21}N_3O_3$
Esflurbiprofen	Antiinflammatory	51543-39-6		$C_{15}H_{13}FO_2$
Esketamine	Sedative	33643-46-8		$C_{13}H_{16}ClNO$
Eslicarbazepine	Antipsychotic	104746-04-5	Y	$C_{15}H_{14}N_2O_2$
Esmirtazapine	Antidepressant	61337-87-9		$C_{17}H_{19}N_3$
Esmolol	Antiarrhythmic	81147-92-4		$C_{16}H_{25}NO_4$
Esomeprazole	Antiulcerative	119141-88-7	Y	$C_{17}H_{19}N_3O_3S$
Esonarimod	Antiarrhythmic	101973-77-7		$C_{14}H_{16}O_4S$
Esorubicin	Antineoplastic	63521-85-7		$C_{27}H_{29}NO_{10}$
Esoxybutynin Chloride	Antispasmodic	119618-22-3		$C_{22}H_{31}NO_3$
Espatropate	Bronchodilator	132829-83-5		$C_{19}H_{23}N_3O_3$
Esproquin	Adrenergic	37517-33-2		$C_{14}H_{21}NOS$
Estazolam	Sedative	29975-16-4	Y	$C_{16}H_{11}ClN_4$
Estradiol	Estrogen	50-28-2	Y	$C_{18}H_{24}O_2$
Estradiol 17 Propionate	Estrogen	3758-34-7	Y	$C_{21}H_{28}O_3$
Estradiol Acetate	Estrogen	4245-41-4		$C_{20}H_{26}O_3$
Estradiol Benzoate	Estrogen	50-50-0		$C_{25}H_{28}O_3$
Estradiol Cypionate	Estrogen	313-06-4		$C_{26}H_{36}O_3$
Estradiol Dipropionate	Estrogen	113-38-2		$C_{24}H_{32}O_4$
Estradiol Enanthate	Estrogen	4956-37-0		$C_{25}H_{36}O_3$
Estradiol Undecylate	Estrogen	3571-53-7		$C_{29}H_{44}O_3$
Estradiol Valerate	Estrogen	979-32-8	Y	$C_{23}H_{32}O_3$
Estramustine	Antineoplastic	2998-57-4	Y	$C_{23}H_{31}Cl_2NO_3$
Estramustine Phosphate	Antineoplastic	4891-15-0		$C_{23}H_{32}Cl_2NO_6P$
Estrapronicate	Estrogen	4140-20-9		$C_{27}H_{31}NO_4$
Estrazinol Hydrobromide	Estrogen	5941-36-6		$C_{20}H_{25}NO_2$
Estriol	Estrogen	50-27-1	Y	$C_{18}H_{24}O_3$
Estrofurate	Estrogen	10322-73-3		$C_{24}H_{26}O_4$
Estrone	Estrogen	53-16-7		$C_{18}H_{22}O_2$
Estrone Sodium Sulfate	Estrogen	481-97-0	Y	$C_{18}H_{22}O_5S$
Esuprone	Anticonvulsant	91406-11-0		$C_{13}H_{14}O_5S$
Eszopiclone	Anxiolytic	138729-47-2	Y	$C_{17}H_{17}ClN_6O_3$
Etabenzarone	Capillary Protectant	15686-63-2		$C_{23}H_{27}NO_3$
Etacepride	Antiprotozoal	68788-56-7		$C_{17}H_{24}N_2O_3$
Etafedrine	Adrenergic	7681-79-0		$C_{12}H_{19}NO$
Etafenone	Vasodilator	90-54-0		$C_{21}H_{27}NO_2$
Etalocib	Antineoplastic	161172-51-6		$C_{33}H_{33}FO_6$
Etaminile	Antitussive	15599-27-6		$C_{15}H_{22}N_2$
Etamiphyllin	Bronchodilator	314-35-2		$C_{13}H_{21}N_5O_2$
Etanidazole	Antineoplastic	22668-01-5		$C_7H_{10}N_4O_4$
Etanterol	Bronchodilator	93047-39-3		$C_{18}H_{24}N_2O_3$
Etaqualone	Sedative	7432-25-9		$C_{17}H_{16}N_2O$
Etarotene	Dermatologic	87719-32-2		$C_{25}H_{32}O_2S$
Etasuline	Antiarrhythmic	16781-39-8		$C_{16}H_{15}ClN_2S$
Etazepine	Anticonvulsant	88124-27-0		$C_{17}H_{17}NO_2$
Etazolate	Antipsychotic	51022-77-6	Y	$C_{14}H_{19}N_5O_2$
Etebenecid	Uricosuric	1213-06-5		$C_{11}H_{15}NO_4S$

Table S1. *Cont.*

Common Name	Indication	CAS Number	Oral	Molecular Formula
Eterobarb	Anticonvulsant	27511-99-5		$C_{16}H_{20}N_2O_5$
Etersalate	Analgesic	62992-61-4		$C_{19}H_{19}NO_6$
Ethacrynic Acid	Diuretic	58-54-8	Y	$C_{13}H_{12}Cl_2O_4$
Ethambutol	Antibacterial	74-55-5	Y	$C_{10}H_{24}N_2O_2$
Ethamivan	Nootropic	304-84-7		$C_{12}H_{17}NO_3$
1,2-Ethanedisulfonic Acid	Sedative	110-04-3	Y	$C_2H_6O_6S_2$
Ethanolamine Hydrofluoride	Dermatologic	111-42-2		$C_4H_{11}NO_2$
Ethaverine	Antispasmodic	486-47-5		$C_{24}H_{29}NO_4$
Ethchlorvynol	Sedative	113-18-8	Y	C_7H_9ClO
Ethenzamide	Analgesic	938-73-8		$C_9H_{11}NO_2$
Ether	Anesthetic	60-29-7		C4H10O
Ethiazide	Diuretic	1824-58-4	Y	$C_9H_{12}ClN_3O_4S_2$
Ethinamate	Sedative	126-52-3	Y	$C_9H_{13}NO_2$
Ethinyl Estradiol	Estrogen	57-63-6	Y	$C_{20}H_{24}O_2$
Ethionamide	Antibacterial	536-33-4	Y	C_8H10N2S
Ethisterone	Progestogen	434-03-7		$C_{21}H_{28}O_2$
Ethoheptazine Citrate	Analgesic	77-15-6		$C_{16}H_{23}NO_2$
Ethomoxane	Sedative	3570-46-5		$C_{15}H_{23}NO_3$
Ethonam Nitrate	Antifungal	15037-44-2		$C_{16}H_{18}N_2O_2$
Ethopabate	Antibacterial	59-06-3		$C_{12}H_{15}NO_4$
Ethopropazine	Antiparkinsonian	522-00-9		$C_{19}H_{25}ClN_2S$
Ethosuximide	Anticonvulsant	77-67-8	Y	$C_7H_{11}NO_2$
Ethotoin	Anticonvulsant	86-35-1	Y	$C_{11}H_{12}N_2O_2$
Ethoxazene	Analgesic	94-10-0		$C_{14}H_{16}N_4O$
Ethoxazorutoside	Cardiotonic	30851-76-4		$C_{33}H_{41}NO_{17}$
Ethoxzolamide	Diuretic	452-35-7	Y	$C_9H_{10}N_2O_3S_2$
Ethybenztropine	Antibacterial	524-83-4		$C_{22}H_{27}NO$
Ethyl Biscoumacetate	Anticoagulant	548-00-5		$C_{22}H_{16}O_8$
Ethyl Carfluzepate	Anxiolytic	65400-85-3		$C_{20}H_{17}ClFN_3O_4$
Ethyl Chloride	Anesthetic	75-00-3		C_2H_5Cl
Ethyl Dibunate	Antitussive	5560-69-0		$C_{20}H_{28}O_3S$
Ethyl Dirazepate	Anxiolytic	23980-14-5		$C_{18}H_{14}Cl_2N_2O_3$
Ethyl Linoleate	Prostaglandin	544-35-4		$C_{20}H_{36}O_2$
Ethyl Loflazepate	Anxiolytic	29177-84-2	Y	$C_{18}H_{14}ClFN_2O_3$
Ethyl Nitrite [Spirit]	Antihypertensive	109-95-5		$C_2H_5NO_2$
Ethyl Pyrophosphate	Unclassified	20680-55-1		$C_6H_{16}O_7P_2$
Ethyl Vinyl Ether	Anesthetic	109-92-2		C_4H_8O
Ethylamine Oleate	Dermatologic	112-80-1		$C_{18}H_{34}O_2$
Ethylamine	Dermatologic	75-04-7		C_2H_7N
Ethylenediamine	Urologic	107-15-3	Y	$C_2H_8N_2$
Ethylestrenol	Steroid	965-90-2		$C_{20}H_{32}O$
Ethylmethylthiambutene	Analgesic	441-61-2		$C_{15}H_{19}NS_2$
Ethylmorphine Methiodide	Analgesic	6696-59-9		$C_{20}H_{26}INO_3$
Ethylnorepinephrine	Bronchodilator	536-24-3		$C_{10}H_{15}NO_3$
Ethylstibamine	Antiprotozoal	554-76-7		$C_6H_8NO_3Sb$
Ethynerone	Progestogen	3124-93-4		$C_{20}H_{23}ClO_2$
Ethynodiol Diacetate	Anesthetic	297-76-7		$C_{24}H_{32}O_4$
Ethypicone	Unclassified	467-90-3		$C_{10}H_{15}NO_2$
Etibendazole	Anthelminithic	64420-40-2		$C_{18}H_{16}FN_3O_4$
Eticlopride	Gastroprokinetic	84226-12-0		$C_{17}H_{25}ClN_2O_3$
Eticyclidine	Antiparkinsonian	2201-15-2		$C_{14}H_{21}N$
Etidocaine	Anesthetic	36637-18-0		$C_{17}H_{28}N_2O$
Etidronate	Bone Resorption Inhibitor	2809-21-4	Y	$C_2H_8O_7P_2$
Etifelmine	Antihypotensive	341-00-4	Y	$C_{17}H_{19}N$
Etifoxine	Anxiolytic	21715-46-8		$C_{17}H_{17}ClN_2O$
Etilamfetamine	Anorexic	457-87-4		$C_{11}H_{17}N$
Etilefrine	Antihypotensive	709-55-7		$C_{10}H_{15}NO_2$
Etilefrine Pivalate	Mydriatic	100696-30-8		$C_{15}H_{23}NO_3$
Etilevodopa	Antiparkinsonian	37178-37-3		$C_{11}H_{15}NO_4$
Etintidine	Antihistaminic	69539-53-3		$C_{12}H_{16}N_6S$
Etipirium Iodide	Unclassified	3478-15-7		$C_{21}H_{26}INO_3$
Etiprednol Dicloacetate	Antiinflammatory	199331-40-3		$C_{24}H_{30}Cl_2O_6$
Etiproston	Prostaglandin	59619-81-7		$C_{24}H_{32}O_7$

Table S1. *Cont.*

Common Name	Indication	CAS Number	Oral	Molecular Formula
Etiracetam	Anticonvulsant	33996-58-6		$C_8H_{14}N_2O_2$
Etiroxate	Nootropic	17365-01-4		$C_{18}H_{17}I_4NO_4$
Etisazole	Antifungal	7716-60-1		$C_9H_{10}N_2S$
Etisomicin	Antibiotic	70639-48-4		$C_{20}H_{39}N_5O_7$
Etisulergine	Antimigraine	64795-23-9		$C_{19}H_{28}N_4O_2S$
Etizolam	Anxiolytic	40054-69-1	Y	$C_{17}H_{15}ClN_4S$
Etocarlide	Antibiotic	1234-30-6		$C_{17}H_{20}N_2O_2S$
Etocrylene	Dermatologic	5232-99-5		$C_{18}H_{15}NO_2$
Etodolac	Antiinflammatory	41340-25-4	Y	$C_{17}H_{21}NO_3$
Etodroxizine	Sedative	17692-34-1		$C_{23}H_{31}ClN_2O_3$
Etofamide	Antineoplastic	25287-60-9		$C_{19}H_{20}Cl_2N_2O_5$
Etofenamate	Analgesic	30544-47-9		$C_{18}H_{18}F_3NO_4$
Etofenprox	Antiinflammatory	80844-07-1		$C_{25}H_{28}O_3$
Etofibrate	Antihyperlipidemic	31637-97-5	Y	$C_{18}H_{18}ClNO_5$
Etoformin	Antidiabetic	45086-03-1		$C_8H_{19}N_5$
Etofuradine	Antianginal	17692-35-2		$C_{18}H_{21}N_3O$
Etofylline	Bronchodilator	519-37-9		$C_9H_{12}N_4O_3$
Etoglucid	Antineoplastic	1954-28-5		$C_{12}H_{22}O_6$
Etolorex	Anorexic	54063-36-4		$C_{12}H_{18}ClNO$
Etolotifen	Antihistaminic	82140-22-5		$C_{24}H_{29}NO_4S$
Etoloxamine	Antihistaminic	1157-87-5		$C_{19}H_{25}NO$
Etomidate	Sedative	33125-97-2		$C_{14}H_{16}N_2O_2$
Etomidoline	Antispasmodic	21590-92-1		$C_{23}H_{29}N_3O_2$
Etomoxir	Cardiotonic	124083-20-1		$C_{17}H_{23}ClO_4$
Etonitazene	Analgesic	911-65-9		$C_{22}H_{28}N_4O_3$
Etonogestrel	Prostaglandin	54048-10-1		$C_{22}H_{28}O_2$
Etoperidone	Antidepressant	52942-31-1		$C_{19}H_{28}ClN_5O$
Etoposide	Antineoplastic	33419-42-0		$C_{29}H_{32}O_{13}$
Etoposide Phosphate	Antineoplastic	117091-64-2		$C_{29}H_{33}O_{16}P$
Etoprindole	Nootropic	54063-37-5		$C_{15}H_{21}N_3O$
Etoprine	Antineoplastic	18588-57-3		$C_{12}H_{12}Cl_2N_4$
Etoricoxib	Antiinflammatory	202409-33-4	Y	$C_{18}H_{15}ClN_2O_2S$
Etorphine	Analgesic	14521-96-1		$C_{25}H_{33}NO_4$
Etosalamide	Antifungal	15302-15-5		$C_{11}H_{15}NO_3$
Etoxadrol	Anesthetic	28189-85-7		$C_{16}H_{23}NO_2$
Etoxeridine	Analgesic	469-82-9		C_{18}H27NO4
Etozolin	Diuretic	73-09-6	Y	$C_{13}H_{20}N_2O_3S$
Etrabamine	Antidepressant	70590-58-8		$C_8H_{12}N_2S$
Etravirine	Antiviral	269055-15-4	Y	$C_{20}H_{15}BrN_{6O}$
Etretinate	Dermatologic	54350-48-0		$C_{23}H_{30}O_3$
Etriciguat	Vasodilator	402595-29-3		$C_{22}H_{16}FN_7$
Etryptamine Acetate	Nootropic	2235-90-7		$C_{12}H_{16}N_2$
Etymemazine	Antihistaminic	523-54-6		$C_{20}H_{26}N_2S$
Eucaine	Analgesic	500-34-5		$C_{15}H_{21}NO_2$
Eucatropine	Mydriatic	100-91-4		$C_{17}H_{25}NO_3$
Eugenol	Analgesic	97-53-0		$C_{10}H_{12}O_2$
Euprocin	Anesthetic	1301-42-4		$C_{24}H_{34}N_2O_2$
Evandamine	Antineoplastic	100035-75-4		$C_{11}H_{16}N_4S$
Everolimus	Immunosuppressant	159351-69-6	Y	$C_{53}H_{83}NO_{14}$
Exalamide	Antifungal	53370-90-4		$C_{13}H_{19}NO_2$
Examorelin	Cardiotonic	140703-51-1		C47H58N12O6
Exaprolol	Adrenergic	55837-19-9		$C_{18}H_{29NO2}$
Exatecan	Antineoplastic	171335-80-1		$C_{24}H_{22}FN_3O_4$
Exemestane	Antineoplastic	107868-30-4	Y	$C_{20}H_{24}O_2$
Exepanol	Antianginal	77416-65-0		$C_{11}H_{15}NO_2$
Exifone	Nootropic	52479-85-3		$C_{13}H_{10}O_7$
Exiproben	Choleretic	26281-69-6		$C_{16}H_{24}O_5$
Exisulind	Antineoplastic	59973-80-7		$C_{20}H_{17}FO_4S$
Ezetimibe	Antihyperlipidemic	163222-33-1	Y	$C_{24}H_{21}F_2NO_3$
Ezlopitant	Antiemetic	147116-64-1	Y	$C_{31}H_{38}N_2O$
Fabesetron	Antiemetic	129300-27-2		$C_{18}H_{19}N_3O$
Fadolmidine	Antidiabetic	189353-31-9		$C_{13}H_{14}N_2O$
Fadrozole	Antineoplastic	102676-47-1	Y	$C_{14}H_{13}N_3$

Table S1. *Cont.*

Common Name	Indication	CAS Number	Oral	Molecular Formula
Falintolol	Antihypertensive	90581-63-8		$C_{12}H_{24}N_2O_2$
Falipamil	Antianginal	77862-92-1		$C_{24}H_{32}N_2O_5$
Falnidamol	Antineoplastic	196612-93-8		$C_{18}H_{19}ClFN_7$
Famciclovir	Antiviral	104227-87-4	Y	$C_{14}H_{19}N_5O_4$
Famiraprinium Chloride	Unclassified	105538-42-9		$C_{15}H_{17}N_3O_2$
Famotidine	Antiulcerative	76824-35-6	Y	$C_8H_{15}N_7O_2S_3$
Fampridine	Nootropic	504-24-5	Y	$C_5H_6N_2$
Famprofazone	Analgesic	22881-35-2		$C_{24}H_{31}N_3O$
Fampronil	Unclassified	134183-95-2		$C_{16}H_6Cl_3F_3N_6$
Fananserin	Antipsychotic	127625-29-0		$C_{23}H_{24}FN_3O_2S$
Fanapanel	Nootropic	161605-73-8		$C_{14}H_{15}F_3N_3O_6P$
Fandofloxacin	Antibiotic	164150-99-6		$C_{20}H_{18}F_2N_4O_3$
Fandosentan Potassium	Anticoagulant	221241-63-0		$C_{25}H_{18}F_3NO_6S$
Fanetizole Mesylate	Immunomodulator	79069-94-6		$C_{17}H_{16}N_2S$
Fantofarone	Antihypertensive	114432-13-2		$C_{31}H_{38}N_2O_5S$
Fantridone	Antidepressant	17692-37-4		$C_{18}H_{20}N_2O$
Farampator	Antipsychotic	211735-76-1		$C_{12}H_{13}N_3O_2$
Farglitazar	Antidiabetic	196808-45-4		$C_{34}H_{30}N_2O_5$
Fasidotril	Antihypertensive	135038-57-2		$C_{23}H_{25}NO_6S$
Fasiplon	Anxiolytic	106100-65-6		$C_{13}H_{15}N_5O_2$
Fasoracetam	Nootropic	110958-19-5		$C_{10}H_{16}N_2O_2$
Fasudil	Vasodilator	103745-39-7	y	$C_{14}H_{17}N_3O_2S$
Fazadinium Bromide	Muscle Relaxant	49564-56-9		$C_{28}H_{24}Br_2N_6$
Fazarabine	Antineoplastic	65886-71-7		$C_8H_{12}N_4O_5$
Febantel	Anthelminithic	58306-30-2		$C_{20}H_{22}N_4O_6S$
Febarbamate	Antidepressant	13246-02-1		$C_{20}H_{27}N_3O_6$
Febuprol	Choleretic	3102-00-9		$C_{13}H_{20}O_3$
Febuverine	Anesthetic	7077-33-0		$C_{28}H_{38}N_2O_4$
Febuxostat	Antiurolithic	144060-53-7	y	$C_{16}H_{16}N_2O_3S$
Feclemine	Unclassified	3590-16-7		$C_{24}H_{42}N_2$
Feclobuzone	Antirheumatic	23111-34-4		$C_{27}H_{25}ClN_2O_4$
Fedotozine	Gastroprokinetic	123618-00-8		$C_{22}H_{31}NO_4$
Fedrilate	Antiviral	23271-74-1		$C_{20}H_{29}NO_4$
Felbamate	Anticonvulsant	25451-15-4	Y	$C_{11}H_{14}N_2O_4$
Felbinac	Antiinflammatory	5728-52-9		$C_{14}H_{12}O_2$
Felipyrine	Antiinflammatory	1980-49-0		$C_{15}H_{20}N_2O$
Felodipine	Antihypertensive	72509-76-3	Y	$C_{18}H_{19}Cl_2NO_4$
Feloprentan	Antimigraine	204267-33-4		$C_{31}H_{32}N_2O_6$
Femoxetine	Antidepressant	59859-58-4		$C_{20}H_{25}NO_2$
Fenabutene	Analgesic	5984-83-8		$C_{12}H_{14}O_2$
Fenacetinol	Unclassified	22521-79-5		$C_{10}H_{13}NO_3$
Fenaclon	Antipsychotic	306-20-7		$C_{11}H_{14}ClNO$
Fenadiazole	Sedative	1008-65-7		$C_8H_6N_2O_2$
Fenaftic Acid	Antifungal	27736-80-7		$C_{24}H_{31}NO_4$
Fenalamide	Muscle Relaxant	4551-59-1		C19H30N2O3
Fenalcomine	Cardiotonic	34616-39-2		$C_{20}H_{27}NO_2$
Fenamifuril	Unclassified	735-64-8		$C_{14}H_{17}NO_5$
Fenamole	Antiinflammatory	5467-78-7		$C_7H_7N_5$
Fenaperone	Antipsychotic	54063-38-6		$C_{21}H_{29}FN_2O_3$
Fenbendazole	Anthelminithic	43210-67-9		$C_{15}H_{13}N_3O_2S$
Fenbenicillin	Antibiotic	1926-48-3		$C_{22}H_{22}N_2O_5S$
Fenbufen	Antiinflammatory	36330-85-5	Y	$C_{16}H_{14}O_3$
Fenbutrazate	Anorexic	4378-36-3		$C_{23}H_{29}NO_3$
Fencamfamin	Nootropic	1209-98-9		$C_{15}H_{21}N$
Fencibutirol	Choleretic	5977-10-6		$C_{16}H_{22}O_3$
Fenclofenac	Antiinflammatory	34645-84-6		$C_{14}H_{10}Cl_2O_3$
Fenclonine	Antidepressant	7424-00-2		$C_9H_{10}ClNO_2$
Fenclorac	Antiinflammatory	36616-52-1		$C_{14}H_{16}Cl_2O_2$
Fenclozic Acid	Antiinflammatory	17969-20-9		$C_{11}H_8ClNO_2S$
Fendiline	Vasodilator	13042-18-7		$C_{23}H_{25}N$
Fendizoate	Antipsychotic	84627-04-3		$C_{20}H_{14}O_4$
Fendosal	Antiinflammatory	53597-27-6		$C_{25}H_{19}NO_3$
Feneritrol	Antihypertensive	15301-67-4		$C_{45}H_{52}O_8$

Table S1. *Cont.*

Common Name	Indication	CAS Number	Oral	Molecular Formula
Fenestrel	Estrogen	7698-97-7		$C_{16}H_{20}O_2$
Fenethazine	Antihistaminic	522-24-7		$C_{16}H_{18}N_2S$
Fenethylline	Nootropic	3736-08-1		$C_{18}H_{23}N_5O_2$
Fenetradil	Antihypertensive	54063-39-7		$C_{22}H_{36}N_2O_3$
Fenflumizole	Antiinflammatory	73445-46-2		$C_{23}H_{18}F_2N_2O_2$
Fenfluramine	Anorexic	458-24-2		$C_{12}H_{16}F_3N$
Fengabine	Antidepressant	80018-06-0		$C_{17}H_{17}Cl_2NO$
Fenharmane	Unclassified	15301-68-5		$C_{18}H_{18}N_2$
Fenimide	Antipsychotic	60-45-7		$C_{13}H_{15}NO_2$
Feniodium Chloride	Antiprotozoal	71585-34-7		$C_{12}H_6Cl_5I$
Fenipentol	Choleretic	583-03-9		$C_{11}H_{16}O$
Fenirofibrate	Antihyperlipidemic	54419-31-7		$C_{17}H_{17}ClO_4$
Fenisorex	Anorexic	34887-52-0		$C_{16}H_{16}FNO$
Fenitrothion	Dermatologic	122-14-5		$C_9H_{12}NO_5PS$
Fenleuton	Antiinflammatory	141579-54-6		$C_{17}H_{15}FN_2O_3$
Fenmetozole	Antidepressant	41473-09-0		$C_{10}H_{10}Cl_2N_2O$
Fenmetramide	Antidepressant	5588-29-4		$C_{11}H_{13}NO_2$
Fenobam	Sedative	57653-26-6		$C_{11}H_{11}ClN_4O_2$
Fenocinol	Unclassified	3671-05-4		$C_{16}H_{18}O_3$
Fenoctimine Sulfate	Antiulcerative	69365-65-7		$C_{27}H_{38}N_2$
Fenofibrate	Antihyperlipidemic	49562-28-9	Y	$C_{20}H_{21}ClO_4$
Fenoldopam	Antihypertensive	67227-56-9		$C_{16}H_{16}ClNO_3$
Fenoprofen	Antiinflammatory	29679-58-1	Y	$C_{15}H_{14}O_3$
Fenoterol	Bronchodilator	13392-18-2		$C_{17}H_{21}NO_4$
Fenoverine	Antispasmodic	37561-27-6		$C_{26}H_{25}$N3O3S
Fenoxazoline	Nootropic	4846-91-7		$C_{13}H_{18}N_2O$
Fenoxedil	Vasodilator	54063-40-0		$C_{28}H_{42}N_2O_5$
Fenozolone	Nootropic	15302-16-6		$C_{11}H_{12}N_2O_2$
Fenpentadiol	Antidepressant	15687-18-0		$C_{12}H_{17}ClO_2$
Fenperate	Unclassified	55837-26-8		$C_{25}H_{31}NO_4$
Fenpipalone	Antiinflammatory	21820-82-6		$C_{17}H_{22}N_2O_2$
Fenpipramide	Antispasmodic	77-01-0		$C_{21}H_{26}N_2O$
Fenpiprane	Antispasmodic	3540-95-2		$C_{20}H_{25}N$
Fenpiverinium Bromide	Antispasmodic	258329-46-3		$C_{22}H_{29}BrN_2O$
Fenprinast	Bronchodilator	75184-94-0		$C_{16}H_{16}ClN_5O$
Fenproporex	Anorexic	16397-28-7		$C_{12}H_{16}N_2$
Fenprostalene	Progestogen	69381-94-8		C23H30O6
Fenquizone	Diuretic	20287-37-0		$C_{14}H_{12}ClN_3O_3S$
Fenretinide	Antineoplastic	65646-68-6		$C_{26}H_{33}NO_2$
Fenspiride	Bronchodilator	5053-06-5		C15H20N2O2
Fentanyl	Analgesic	437-38-7		$C_{22}H_{28}N_2O$
Fenthion	Ectoparasiticide	55-38-9		$C_{10}H_{15}O_3PS_2$
Fentiazac	Antiinflammatory	18046-21-4		$C_{17}H_{12}ClNO_2S$
Fenticlor	Antibacterial	97-24-5		$C_{12}H_8Cl_2O_2S$
Fenticonazole	Antifungal	72479-26-6		$C_{24}H_{20}Cl_2N_2OS$
Fentonium Bromide	Antibiotic	34786-74-8		$C_{31}H_{34}BrNO_4$
Fenyripol	Muscle Relaxant	3607-24-7		$C_{12}H_{13}N_3O$
Fepentolic Acid	Unclassified	17243-33-3		$C_{12}H_{16}O_4$
Fepitrizol	Unclassified	53415-46-6		$C_{15}H_{14}N_4O$
Fepradinol	Antiinflammatory	36981-91-6		$C_{12}H_{19}NO_2$
Feprazone	Antiinflammatory	30748-29-9		$C_{20}H_{20}N_2O_2$
Fepromide	Analgesic	54063-41-1		$C_{23}H_{30}N_2O_5$
Feprosidnine	Unclassified	22293-47-6		$C_{11}H_{13}N_3O$
Ferric Cacodylate	Anthelminthic	75-60-5		$C_2H_7AsO_2$
Ferric Glycerophosphate	Hemantic	56-81-5		$C_3H_8O_3$
Fesoterodine	Urologic	286930-02-7	Y	$C_{26}H_{37}NO_3$
Fetoxylate	Muscle Relaxant	54063-45-5		$C_{36}H_{36}N_2O_3$
Fexicaine	Anesthetic	54063-46-6		$C_{25}H_{34}N_2O_4$
Fexinidazole	Antiprotozoal	59729-37-2	Y	$C_{12}H_{13}N_3O_3S$
Fexofenadine	Antihistaminic	83799-24-0	Y	$C_{32}H_{39}NO_4$
Fezatione	Antifungal	15387-18-5		$C_{17}H_{14}N_2S_2$
Fezolamine Fumarate	Antidepressant	80410-36-2		$C_{20}H_{23}N_3$
Fiacitabine	Antiviral	69123-90-6		$C_9H_{11}FIN_3O_4$

Table S1. *Cont.*

Common Name	Indication	CAS Number	Oral	Molecular Formula
Fialuridine	Antiviral	69123-98-4		$C_9H_{10}FIN_2O_5$
Fibracillin	Antibiotic	51154-48-4		$C_{26}H_{28}ClN_3O_6S$
Fidarestat	Antidiabetic	136087-85-9		$C_{12}H_{10}FN_3O_4$
Fidexaban	Anticoagulant	183305-24-0		$C_{25}H_{24}F_2N_6O_5$
Fiduxosin	Androgen	208993-54-8		$C_{30}H_{29}N_5O_4S$
Figopitant	Antiemetic	502422-74-4		$C_{27}H_{31}F_6N_3O$
Filaminast	Bronchodilator	141184-34-1		$C_{15}H_{20}N_2O_4$
Filenadol	Analgesic	78168-92-0		$C_{14}H_{19}NO_4$
Filipin	Antifungal	480-49-9		$C_{35}H_{58}O_{11}$
Finafloxacin	Antibiotic	209342-40-5		$C_{20}H_{19}FN_4O_4$
Finasteride	Urologic	98319-26-7	Y	$C_{23}H_{36}N_2O_2$
Fingolimod	Immunomodulator	162359-55-9	Y	$C_{19}H_{33}NO_2$
Finrozole	Antiprotozoal	160146-17-8		$C_{18}H_{15}FN_4O$
Fipamezole	Antihypertensive	150586-58-6		$C_{14}H_{15}FN_2$
Fipexide	Nootropic	34161-24-5		$C_{20}H_{21}ClN_2O_4$
Firocoxib	Antiinflammatory	189954-96-9		$C_{17}H_{20}O_5S$
Fispemifene	Antineoplastic	341524-89-8		$C_{26}H_{27}ClO_3$
Flamenol	Antiarrhythmic	2174-64-3		$C_7H_8O_3$
Flavamine	Unclassified	15686-60-9		$C_{21}H_{23}NO_2$
Flavodic Acid	Unclassified	37470-13-6		$C_{19}H_{14}O_8$
Flavodilol Maleate	Antihypertensive	79619-31-1		$C_{21}H_{23}NO_4$
Flavoxate	Antispasmodic	15301-69-6	Y	$C_{24}H_{25}NO4$
Flazalone	Antiinflammatory	21221-18-1		$C_{19}H_{19}F_2NO_2$
Flecainide Acetate	Antiarrhythmic	54143-55-4	Y	$C_{17}H20F6N2O3$
Flerobuterol	Antidepressant	82101-10-8		$C_{12}H_{18}FNO$
Fleroxacin	Antibiotic	79660-72-3		$C_{17}H_{18}F3N3O3$
Flesinoxan	Anxiolytic	98206-10-1		$C_{22}H_{26}FN_3O_4$
Flestolol Sulfate	Antihypertensive	87721-62-8		$C_{15}H_{22}FN_3O_4$
Fletazepam	Muscle Relaxant	34482-99-0		$C_{17}H_{13}ClF_4N_2$
Flezelastine	Bronchodilator	135381-77-0		$C_{29}H_{30}FN_3O$
Flibanserin	Antidepressant	167933-07-5		$C_{20}H_{21}F_3N_4O$
Flindokalner	Nootropic	187523-35-9		$C_{16}H_{10}ClF_4NO_2$
Floctafenine	Analgesic	23779-99-9		$C_{20}H_{17}F_3N_2O_4$
Flomoxef	Antibiotic	99665-00-6		$C_{15}H_{18}F_2N_6O_7S_2$
Flopropione	Antispasmodic	2295-58-1		C_{9H10O4}
Florantyrone	Choleretic	519-95-9		$C_{20}H_{14}O3$
Flordipine	Antihypertensive	77590-96-6		$C_{26}H33F3N2O5$
Floredil	Vasodilator	53731-36-5		$C_{16}H_{25}NO_4$
Florfenicol	Antibiotic	73231-34-2		$C_{12}H_{14}Cl_2FNO_4S$
Florifenine	Antiinflammatory	83863-79-0		$C_{23}H22F3N3O2$
Flosatidil	Vasodilator	113593-34-3		$C_{26}H_{34}F_3N_3O_3S$
Flosequinan	Antihypertensive	76568-02-0		$C_{11}H_{10}FNO_2S$
Flosulide	Antiinflammatory	80937-31-1		$C_{16}H_{13}F_2NO_4S$
Flotrenizine	Antihistaminic	89224-08-8		$C_{31}H_{38}F_2N_2O$
Floverine	Analgesic	27318-86-1		$C_{10}H_{14}O_4$
Floxacillin	Antibiotic	5250-39-5	Y	$C_{19}H_{17}ClFN_3O_5S$
Floxacrine	Nootropic	53966-34-0		$C_{20}H_{13}ClF_3NO_3$
Floxuridine	Antiviral	50-91-9	Y	$C_9H_{11}FN_2O_5$
Fluacizine	Antidepressant	30223-48-4		$C_{20}H_{21}F_3N_2OS$
Flualamide	Diuretic	5107-49-3		$C_{17}H_{23}F_3N_2O_2$
Fluanisone	Antipsychotic	1480-19-9		$C_{21}H_{25}FN_2O_2$
Fluazacort	Antiinflammatory	19888-56-3		$C_{25}H_{30}FNO_6$
Flubanilate	Nootropic	847-20-1		$C_{14}H_{19}F_3N_2O_2$
Flubendazole	Anthelminithic	31430-15-6	Y	$C_{16}H_{12}FN_3O_3$
Flubepride	Antiprotozoal	56488-61-0		$C_{20}H_{24}FN_3O_4S$
Flucarbril	Analgesic	2261-94-1		$C_{11}H_8F_3NO$
Flucetorex	Anorexic	40256-99-3		$C_{20}H_{21}F_3N_2O_3$
Flucindole	Antipsychotic	40594-09-0		$C_{14}H_{16}F_2N_2$
Fluciprazine	Antipsychotic	54340-64-6		$C_{21}H_{29}FN_2O_2$
Flucloronide	Glucocorticoid	3693-39-8		$C_{24}H_{29}Cl_2FO_5$
Fluconazole	Antifungal	86386-73-4	Y	$C_{13}H_{12}F_2N_6O$
Flucrylate	Dermatologic	23023-91-8		$C_7H_6F_3NO_2$
Flucytosine	Antifungal	2022-85-7	Y	$C_4H_4FN_3O$

Table S1. *Cont.*

Common Name	Indication	CAS Number	Oral	Molecular Formula
Fludalanine	Antibacterial	35523-45-6		$C_3H_5DFNO_2$
Fludarabine	Antineoplastic	21679-14-1	Y	$C_{10}H_{12}FN_5O_4$
Fludiazepam	Anxiolytic	3900-31-0		$C_{16}H_{12}ClFN_2O$
Fludorex	Anorexic	15221-81-5		$C_{11}H_{14}F_3NO$
Fludoxopone	Antiprotozoal	71923-29-0		$C_{21}H_{21}FN_2O_3$
Fludrocortisone	Steroid	127-31-1	Y	$C_{21}H_{29}FO_5$
Fludrocortisone Acetate	Steroid	514-36-3	Y	$C_{23}H_{31}FO_6$
Flufenamic Acid	Antiinflammatory	530-78-9		C_{14}H10F3NO2
Flufenisal	Analgesic	22494-27-5		$C_{15}H_{11}FO_4$
Flufosal	Analgesic	65708-37-4		$C_8H_6F_3O_6P$
Flufylline	Bronchodilator	82190-91-8		$C_{21}H_{24}FN_5O_3$
Fluindarol	Vasodilator	6723-40-6		$C_{16}H_9F_3O_2$
Fluindione	Anticonvulsant	957-56-2		$C_{15}H_9FO_2$
Flumazenil	Antidote	78755-81-4		$C_{15}H_{14}FN_3O_3$
Flumecinol	Hepatoprotectant	56430-99-0		$C_{16}H_{15}F_3O$
Flumedroxone	Analgesic	15687-21-5		$C_{22}H_{29}F_3O_3$
Flumequine	Antibacterial	42835-25-6		$C_{14}H_{12}FNO_3$
Flumeridone	Antiemetic	75444-64-3		$C_{22}H_{23}ClFN_5O_2$
Flumethasone	Glucocorticoid	2135-17-3		$C_{22}H_{28}F_2O_5$
Flumethasone Pivalate	Glucocorticoid	2002-29-1		$C_{27}H_{36}F_2O_6$
Flumethiazide	Opthalmic	148-56-1		$C_8H_6F_3N_3O_4S_2$
Flumethrin	Ectoparasiticide	69770-45-2		$C_{28}H_{22}Cl_2FNO_3$
Flumetramide	Muscle Relaxant	7125-73-7		$C_{11}H_{10}F_3NO_2$
Flumexadol	Sedative	30914-89-7		$C_{11}H_{12}F_3NO$
Flumezapine	Antipsychotic	61325-80-2		$C_{17}H_{19}FN_4S$
Fluminorex	Anorexic	720-76-3		$C_{10}H_9F_3N_2O$
Flumizole	Antiinflammatory	36740-73-5		$C_{18}H_{15}F_3N_2O_2$
Flumoxonide	Steroid	60135-22-0		$C_{26}H_{34}F_2O_7$
Flunamine	Antidepressant	50366-32-0		$C_{15}H_{15}F_2NO$
Flunarizine	Vasodilator	52468-60-7	y	$C_{26}H_{26}F_2N_2$
Flunidazole	Antiprotozoal	4548-15-6		$C_{11}H_{10}FN_3O_3$
Flunisolide	Glucocorticoid	3385-03-3		$C_{24}H_{31}FO_6$
Flunisolide Acetate	Antiinflammatory	4533-89-5		$C_{26}H_{33}FO_7$
Flunitrazepam	Sedative	1622-62-4	Y	$C_{16}H_{12}FN_3O_3$
Flunixin	Antiinflammatory	38677-85-9		$C_{14}H_{11}F_3N_2O_2$
Flunoprost	Antiulcerative	86348-98-3		$C_{22}H_{29}FO_5$
Flunoxaprofen	Antiinflammatory	66934-18-7		$C_{16}H_{12}FNO_3$
Fluocinolone Acetonide	Antiinflammatory	67-73-2		$C_{24}H_{30}$F2O6
Fluocinonide	Antiinflammatory	356-12-7		$C_{26}H_{32}F_2O_7$
Fluocortin Butyl	Antiinflammatory	41767-29-7		$C_{26}H_{35}FO_5$
Fluocortolone	Glucocorticoid	152-97-6		$C_{22}H_{29}FO_4$
Fluocortolone Caproate	Glucocorticoid	303-40-2		C28H39FO5
Fluoresone	Anticonvulsant	2924-67-6		$C_8H_9FO_2S$
Fluorometholone	Glucocorticoid	426-13-1		$C_{22}H_{29}FO_4$
Fluorouracil	Antineoplastic	51-21-8		$C_4H_3FN_2O_2$
Fluotracen	Antipsychotic	35764-73-9	Y	$C_{21}H_{24}F_3N$
Fluoxetine	Antidepressant	54910-89-3	Y	$C_{17}H_{18}F_3NO$
Fluoxymesterone	Androgen	76-43-7		$C_{20}H_{29}FO_3$
Fluparoxan	Antidepressant	105182-45-4	Y	$C_{10}H_{10}FNO_2$
Flupentixol	Antipsychotic	2709-56-0	Y	$C_{23}H_{25}F_3N_2OS$
Fluperamide	Antispasmodic	53179-10-5		$C_{30}H_{32}ClF_3N_2O_2$
Fluperlapine	Antipsychotic	67121-76-0		$C_{19}H_{20}FN_3$
Fluperolone Acetate	Glucocorticoid	2119-75-7		$C_{24}H_{31}FO_6$
Fluphenazine Decanoate	Antipsychotic	5002-47-1		$C_{32}H_{44}F_3N_3O_2S$
Fluphenazine Enanthate	Antipsychotic	2746-81-8	Y	$C_{29}H_{38}F_3N_3O_2S$
Fluphenazine	Antipsychotic	69-23-8		$C_{22}H_{26}F_3N_3OS$
Flupimazine	Antipsychotic	47682-41-7		$C_{23}H_{27}F_3N_2O_2S$
Flupirtine	Analgesic	56995-20-1	Y	$C_{15}H_{17}FN_4O_2$
Flupranone	Analgesic	21686-10-2		$C_{20}H_{24}FN_3O_2$
Fluprazine	Antihypertensive	76716-60-4		$C_{14}H_{19}F_3N_4O$
Fluprednidene	Analgesic	2193-87-5		C_{22}H27FO5
Fluprednisolone	Glucocorticoid	53-34-9	Y	$C_{21}H_{27}FO_5$
Fluprednisolone Valerate	Glucocorticoid	23257-44-5		$C_{26}H_{35}FO_6$

Table S1. *Cont.*

Common Name	Indication	CAS Number	Oral	Molecular Formula
Fluprofen	Antiinflammatory	17692-38-5		$C_{15}H_{13}FO_2$
Fluprofylline	Bronchodilator	85118-43-0		$C_{22}H_{26}FN_5O_3$
Fluproquazone	Analgesic	40507-23-1		$C_{18}H_{17}FN_2O$
Fluprostenol	Prostaglandin	40666-16-8		$C_{23}H_{29}F_3O_6$
Fluquazone	Antiinflammatory	37554-40-8		$C_{16}H_{10}ClF_3N_2O$
Fluradoline	Analgesic	71316-84-2		$C_{17}H_{16}FNOS$
Flurandrenolide	Glucocorticoid	1524-88-5		$C_{24}H_{33}FO_6$
Flurantel	Anthelminithic	30533-89-2		$C_{19}H_{12}F_6N_2O_7$
Flurazepam	Anticonvulsant	17617-23-1	Y	$C_{21}H_{23}ClFN_3O$
Flurbiprofen	Antiinflammatory	5104-49-4	Y	$C_{15}H_{13}FO_2$
Fluretofen	Antiinflammatory	56917-29-4		$C_{14}H_9F$
Flurithromycin	Antibiotic	82664-20-8		$C_{37}H_{66}FNO_{13}$
Flurocitabine	Antineoplastic	37717-21-8		$C_9H_{10}FN_3O_4$
Flurofamide	Antibiotic	70788-28-2		$C_7H_9FN_3O_2P$
Flurogestone Acetate	Progestogen	2529-45-5		$C_{23}H_{31}FO_5$
Flurothyl	Nootropic	333-36-8		$C_4H_4F_6O$
Fluroxene	Anesthetic	406-90-6		$C_4H_5F_3O$
Flusoxolol	Antihypertensive	84057-96-5		$C_{22}H_{30}FNO_4$
Fluspiperone	Antipsychotic	54965-22-9		$C_{23}H_{25}F_2N_3O_2$
Fluspirilene	Antipsychotic	1841-19-6	Y	$C_{29}H_{31}F_2N_3O$
Flutamide	Antineoplastic	13311-84-7	Y	$C_{11}H_{11}F_3N_2O_3$
Flutazolam	Anxiolytic	27060-91-9		$C_{19}H_{18}ClFN_2O_3$
Flutemazepam	Anxiolytic	52391-89-6		$C_{16}H_{12}ClFN_2O_2$
Flutiazin	Antiinflammatory	7220-56-6		$C_{14}H_8F_3NO_2S$
Fluticasone Propionate	Glucocorticoid	80474-14-2		$C_{25}H_{31}F_3O_5S$
Flutizenol	Antiarrhythmic	10202-40-1		$C_{20}H_{24}F_3N_3OS_2$
Flutomidate	Sedative	84962-75-4		$C_{14}H_{15}FN_2O_2$
Flutonidine	Antihypertensive	28125-87-3		$C_{10}H_{12}FN_3$
Flutoprazepam	Anxiolytic	25967-29-7		$C_{19}H_{16}ClFN_2O$
Flutrimazole	Antifungal	119006-77-8		$C_{22}H_{16}F_2N_2$
Flutroline	Antipsychotic	70801-02-4		$C_{27}H_{25}F_3N_2O$
Flutropium Bromide	Bronchodilator	63516-07-4		$C_{24}H_{29}BrFNO_3$
Fluvastatin	Antihyperlipidemic	93957-54-1	Y	$C_{24}H_{26}FNO_4$
Fluvoxamine	Antidepressant	54739-18-3	Y	$C_{15}H_{21}F_3N_2O_2$
Fluzinamide	Anticonvulsant	76263-13-3		$C_{12}H_{13}F_3N_2O_2$
Fluzoperine	Antitussive	52867-77-3		$C_{15}H_{19}FN_2O_2$
Fodipir	Antitussive	118248-91-2		$C_{22}H_{32}N_4O_{14}P_2$
Folescutol	Capillary Protectant	15687-22-6		$C_{14}H_{15}NO_5$
Fomepizole	Antidote	7554-65-6	Y	$C_4H_6N_2$
Fomidacillin	Antibiotic	98048-07-8		$C_{24}H_{28}N_6O_{10}S$
Fominoben	Antitussive	18053-31-1		$C_{21}H_{24}ClN_3O_3$
Fomocaine	Anesthetic	17692-39-6		$C_{20}H_{25}NO_2$
Fonazine Mesylate	Antimigraine	7456-24-8		$C_{19}H_{25}N_{3O2S2}$
Fopirtoline	Analgesic	22514-23-4		$C_{11}H_{15}ClN_2OS$
Forasartan	Antihypertensive	145216-43-9		$C_{23}H_{28}N_8$
Forfenimex	Immunomodulator	71522-58-2		$C_9H_{11}NO4$
Formebolone	Steroid	2454-11-7		$C_{21}H_{28}O_4$
Formestane	Antineoplastic	566-48-3		$C_{19}H_{26}O_3$
Formetorex	Anorexic	22148-75-0		$C_{10}H_{13}NO$
Forminitrazole	Unclassified	500-08-3		$C_4H_3N_3O_3S$
Formocortal	Glucocorticoid	2825-60-7		$C_{29}H_{38}ClFO_8$
Formoterol	Bronchodilator	73573-87-2	Y	$C_{19}H_{24}N_2O_4$
Forodesine	Antineoplastic	209799-67-7		$C_{11}H_{14}N_4O_4$
Foropafant	Antithrombotic	136468-36-5		$C_{28}H_{40}N_4S$
Fosamprenavir	Antiviral	226700-79-4	Y	$C_{25}H_{36}N_3O_9PS$
Fosarilate	Antiviral	73514-87-1		$C_{17}H_{28}ClO_5P$
Fosazepam	Sedative	35322-07-7		$C_{18}H_{18}ClN_2O_2P$
Foscarnet	Antiviral	4428-95-9		CH_3O_5P
Foscolic Acid	Unclassified	2398-95-0		$C_6H_{11}O_8P$
Fosenazide	Unclassified	16543-10-5		$C_{14}H_{15}N_2O_2P$
Fosfluconazole	Antifungal	194798-83-9		$C_{13}H_{13}F_2N_6O_4P$
Fosfluridine Tidoxil	Antiviral	174638-15-4		$C_{34}H_{62}FN_2O_{10}PS$
Fosfomycin	Antibiotic	23155-02-4	Y	$C_3H_7O_4P$

Table S1. *Cont.*

Common Name	Indication	CAS Number	Oral	Molecular Formula
Fosfonet Sodium	Antiviral	4408-78-0		$C_2H_5O_5P$
Fosfosal	Analgesic	6064-83-1		$C_7H_7O_6P$
Fosfructose Trisodium	Cardiotonic	488-69-7		$C_6H_{14}O_{12}P_2$
Fosinopril	Antihypertensive	98048-97-6		$C_{30}H_{46}NO_7P$
Fosinoprilat	Antihypertensive	95399-71-6		$C_{23}H_{34}NO_5P$
Fosmenic Acid	Unclassified	13237-70-2		$C_7H_{13}O_3P$
Fosmidomycin	Antibiotic	66508-53-0		$C_4H_{10}NO_5P$
Fosopamine	Cardiotonic	103878-96-2		$C_9H_{14}NO_5P$
Fosphenytoin	Anticonvulsant	93390-81-9		$C_{16}H_{15}N_2O_6P$
Fospirate	Anthelminithic	5598-52-7		$C_7H_7Cl_3NO_4P$
Fospropofol Disodium	Sedative	258516-89-1		$C_{13}H_{21}O_5P$
Fosquidone	Antineoplastic	114517-02-1		$C_{28}H_{22}NO_6P$
Fostedil	Vasodilator	75889-62-2		$C_{18}H_{20}NO_3PS$
Fostriecin Sodium	Antineoplastic	87810-56-8		$C_{19}H_{27}O_9P$
Fosveset	Antitussive	193901-91-6		$C_{33}H_{44}N_3O_{14}P$
Fotemustine	Antineoplastic	92118-27-9		$C_9H_{19}ClN_3O_5P$
Fozivudine Tidoxil	Antiviral	141790-23-0		$C_{35}H_{64}N_5O_8PS$
Frabuprofen	Antiinflammatory	98207-14-8		$C_{26}H_{33}F_3N_2O_2$
Fradafiban	Antithrombotic	148396-36-5		$C_{20}H_{21}N_3O_4$
Frakefamide	Antiulcerative	188196-22-7		$C_{30}H_{34}FN_5O_5$
Framycetin	Antibiotic	119-04-0		$C_{23}H_{46}N_6O_{13}$
Frentizole	Immunomodulator	26130-02-9		$C_{15}H_{13}N_3O_2S$
Freselestat	Antidiabetic	208848-19-5		$C_{23}H_{28}N_6O_4$
Fronepidil	Antihypertensive	79700-63-3		$C_{21}H_{31}NO_2$
fropenem	Antibiotic	106560-14-9		$C_{12}H_{15}NO_5S$
Frovatriptan	Antimigraine	158747-02-5	Y	$C_{14}H_{17}N_3O$
Froxiprost	Bronchodilator	62559-74-4		$C_{24}H_{29}F_3O_6$
Ftaxilide	Antidiabetic	19368-18-4		$C_{16}H_{15}NO_3$
Ftivazide	Antibacterial	149-17-7		$C_{14}H_{13}N_3O_3$
Ftormetazine	Antipsychotic	33414-30-1		$C_{21}H_{22}F_3N_3OS$
Ftorpropazine	Antidepressant	33414-36-7		$C_{22}H_{24}F_3N_3O_2S$
Fubrogonium Iodide	Unclassified	3690-58-2		$C_{14}H_{23}BrNO_3I$
Fudosteine	Mucolytic	13189-98-5	Y	$C_6H_{13}NO_3S$
Fulvestrant	Antineoplastic	129453-61-8		$C_{32}H_{47}F_5O_3S$
Fumagillin	Antineoplastic	23110-15-8	Y	$C_{26}H_{34}O_7$
Fumoxicillin	Antibacterial	78186-33-1		$C_{21}H_{21}N_3O_6S$
Fuprazole	Antiulcerative	60248-23-9		$C_{28}H_{30}N_4O_2$
Furacrinic Acid	Unclassified	23580-33-8		$C_{15}H_{14}O_4$
Furafylline	Bronchodilator	80288-49-9		$C_{12}H_{12}N_4O_3$
Furalazine	Antibiotic	556-12-7		$C_9H_7N_5O_3$
Furaltadone	Antibiotic	139-91-3		$C_{13}H_{16}N_4O_6$
Furaprofen	Antiinflammatory	67700-30-5		$C_{17}H_{14}O_3$
Furazabol	Steroid	1239-29-8		$C_{20}H_{30}N_2O_2$
Furazolium Chloride	Antibacterial	3878-26-0		$C_9H_8ClN_3O_3S$
Furbucillin	Antibiotic	54340-65-7		$C_{19}H_{24}N_2O_7S$
Furcloprofen	Antiinflammatory	58012-63-8		$C_{15}H_{11}ClO_3$
Furegrelate Sodium	Antihypertensive	85666-24-6		$C_{15}H_{11}NO_3$
Furethidine	Antihypertensive	2385-81-1		$C_{21}H_{31}NO_4$
Furfenorex	Anorexic	3776-93-0		$C_{15}H_{19}NO$
Furfuryltrimethyl Ammonium Iodide	Unclassified	7618-86-2		$C_8H_{14}INO$
Furidarone	Cardiotonic	4662-17-3		$C_{13}H_{10}I_2O_3$
Furmethoxadone	Unclassified	6281-26-1		$C_9H_9N_3O_5$
Furnidipine	Antihypertensive	138661-03-7		$C_{21}H_{24}N_2O_7$
Furobufen	Antiinflammatory	38873-55-1		$C_{16}H_{12}O_4$
Furodazole	Anthelminithic	56119-96-1		$C_{15}H_{11}N_3O_2$
Furofenac	Antiinflammatory	56983-13-2		$C_{12}H_{14}O_3$
Furomazine	Analgesic	28532-90-3		$C_{24}H_{27}ClN_2O_3S$
Furomine	Antianginal	142996-66-5		$C_{20}H_{32}N_2O_4$
Furosemide	Diuretic	54-31-9	Y	$C_{12}H_{11}ClN_2O_5S$
Furostilbestrol	Antineoplastic	549-40-6		$C_{28}H_{24}O_6$
Furterene	Diuretic	7761-75-3		$C_{10}H_9N_7O$
Fusidic Acid	Antibacterial	6990-06-3	Y	$C_{31}H_{48}O_6$

Table S1. *Cont.*

Common Name	Indication	CAS Number	Oral	Molecular Formula
Fuzlocillin	Antibiotic	66327-51-3		$C_{25}H_{26}N_6O_8S$
Gabapentin	Anticonvulsant	60142-96-3	Y	$C_9H_{17}NO_2$
Gabexate	Anticoagulant	39492-01-8		$C_{16}H_{23}N_3O_4$
Gaboxadol	Anticonvulsant	64603-91-4		$C_6H_8N_2O_2$
Gacyclidine	Anticonvulsant	68134-81-6		$C_{16}H_{25}NS$
Galamustine	Antineoplastic	105618-02-8		$C_{10}H_{19}Cl_2NO_5$
Galantamine	Nootropic	357-70-0	Y	$C_{17}H_{21}NO_3$
Galarubicin	Antineoplastic	195612-80-7		$C_{30}H_{32}FNO_{13}$
Galdansetron	Antiemetic	116684-92-5		$C_{18}H_{19}N_3O$
Gallamine Triethiodide	Neuromuscular Blocking Agent	65-29-2		$C_{30}H_{60}I_3N_3O_3$
Gallopamil	Antianginal	16662-47-8	Y	$C_{28}H_{40}N_2O_5$
Galocitabine	Antineoplastic	124012-42-6		$C_{19}H_{22}FN_3O_8$
Galosemide	Diuretic	52157-91-2		$C_{15}H_{14}F_3N_3O_3S$
Gamfexine	Antidepressant	7273-99-6		$C_{17}H_{27}N$
Gamma-Aminobutyric Acid	Antihypertensive	56-12-2		$C_4H_9NO_2$
Gamolenic Acid	Dermatologic	506-26-3	Y	$C_{18}H_{30}O_2$
Ganaxolone	Anticonvulsant	38398-32-2	Y	$C_{22}H_{36}O_2$
Ganciclovir	Antiviral	82410-32-0	Y	$C_9H_{13}N_5O_4$
Ganglefene	Vasodilator	299-61-6		$C_{20}H_{33}NO_3$
Ganstigmine	Neuromuscular Blocking Agent	457075-21-7		$C_{22}H_{27}N_3O_3$
Gantofiban	Antithrombotic	183547-57-1		$C_{21}H_{29}N_5O_6$
Gapicomine	Analgesic	1539-39-5		$C_{12}H_{13}N_3$
Gapromidine	Analgesic	106686-40-2		$C_{14}H_{21}N_7$
Garenoxacin Mesylate	Antibacterial	194804-75-6		$C_{23}H_{20}F_2N_2O_4$
Gatifloxacin	Antibiotic	112811-59-3		$C_{19}H_{22}FN_3O4$
Gavestinel	Nootropic	153436-22-7		$C_{18}H_{12}Cl_2N_2O_3$
Gedocarnil	Anxiolytic	109623-97-4		$C_{23}H_{21}ClN_2O_4$
Gefarnate	Antiulcerative	51-77-4		$C_{27}H_{44}O_2$
Gefitinib	Antineoplastic	184475-35-2	Y	$C_{22}H_{24}ClFN_4O_3$
Gemazocine	Analgesic	54063-47-7		$C_{20}H_{29}NO$
Gemcabene Calcium	Antihyperlipidemic	183293-82-5		$C_{16}H_{30}O_5$
Gemcadiol	Antihyperlipidemic	35449-36-6		$C_{14}H_{30}O_2$
Gemcitabine	Antineoplastic	95058-81-4		$C_9H_{11}F_2N_3O_4$
Gemeprost	Prostaglandin	64318-79-2		$C_{23}H_{38}O_5$
Gemfibrozil	Antihyperlipidemic	25812-30-0	Y	$C_{15}H_{22}O_3$
Gemifloxacin	Antibiotic	175463-14-6	Y	$C_{18}H_{20}FN_5O_4$
Gemopatrilat	Antihypertensive	160135-92-2		$C_{19}H_{26}N_2O_4S$
Gepefrine	Antihypotensive	18840-47-6		$C_9H_{13}NO$
Gepirone	Anxiolytic	83928-76-1	Y	$C_{19}H_{29}N_5O_2$
Geroquinol	Antibiotic	10457-66-6		$C_{16}H_{22}O_2$
Gestaclone	Progestogen	19291-69-1		$C_{23}H_{27}ClO_2$
Gestadienol	Unclassified	14340-01-3		$C_{20}H_{26}O_3$
Gestodene	Progestogen	60282-87-3		$C_{21}H_{26}O_2$
Gestonorone Caproate	Progestogen	1253-28-7		$C_{26}H_{38}O_4$
Gestrinone	Progestogen	16320-04-0	Y	$C_{21}H_{24}O_2$
Gevotroline	Capillary Protectant	107266-06-8		$C_{19}H_{20}FN_3$
Gimatecan	Antineoplastic	292618-32-7		$C_{25}H_{25}N_3O_5$
Gimeracil	Antineoplastic	103766-25-2		$C_5H_4ClNO_2$
Giparmen	Unclassified	67268-43-3		$C_{13}H_{10}O_3$
Giracodazole	Antineoplastic	110883-46-0		$C_6H_{11}ClN_4O$
Girisopam	Anxiolytic	82230-53-3		$C_{18}H_{17}ClN_2O_2$
Gitaloxin	Cardiotonic	3261-53-8		$C_{42}H_{64}O_{15}$
Gitoformate	Cardiotonic	10176-39-3		$C_{46}H_{64}O_{19}$
Glafenine	Analgesic	3820-67-5		$C_{19}H_{17}ClN_2O_4$
Glaucarubin	Antiamebic	1448-23-3		$C_{25}H_{36}O_{10}$
Glaziovine	Antiulcerative	6808-72-6		$C_{18}H_{19}NO_3$
Glemanserin	Anxiolytic	107703-78-6		$C_{20}H_{25}NO$
Glenvastatin	Antihyperlipidemic	122254-45-9		$C_{27}H_{26}FNO_3$
Gliamilide	Antidiabetic	51876-98-3		$C_{23}H_{33}N_5O_5S$
Glibornuride	Antidiabetic	26944-48-9	Y	$C_{18}H_{26}N_2O_4S$
Glibutimine	Antiulcerative	25859-76-1		$C_{21}H_{30}N_4O_3S$

Table S1. *Cont.*

Common Name	Indication	CAS Number	Oral	Molecular Formula
Glicaramide	Antidiabetic	36980-34-4		$C_{30}H_{42}N_6O_5S$
Glicetanile Sodium	Antidiabetic	24455-58-1		$C_{23}H_{25}ClN_4O_4S$
Gliclazide	Antidiabetic	21187-98-4		$C_{15}H_{21}N_3O_3S$
Gliflumide	Antidiabetic	35273-88-2		$C_{25}H_{29}FN_4O_4S$
Glimepiride	Antidiabetic	93479-97-1	Y	$C_{24}H_{34}N_4O_5S$
Glipalamide	Antidiabetic	37598-94-0		$C_{12}H_{15}N_3O_3S$
Glipizide	Antidiabetic	29094-61-9	Y	$C_{21}H_{27}N_5O_4S$
Gliquidone	Antidiabetic	33342-05-1		$C_{27}H_{33}N_3O_6S$
Glisamuride	Antidiabetic	74680-07-2		$C_{23}H_{31}N_5O_4S$
Glisentide	Antidiabetic	32797-92-5		$C_{22}H_{27}N_3O_5S$
Glisolamide	Antidiabetic	24477-37-0		$C_{20}H_{26}N_4O_5S$
Glisoxepide	Antidiabetic	25046-79-1		$C_{20}H_{27}N_5O_5S$
Gloxazone	Antibiotic	2507-91-7		$C_8H_{16}N_6OS_2$
Gloximonam	Antibacterial	90850-05-8		$C_{18}H_{25}N_5O_8S$
Glucalox	Unclassified	18673-08-0		$C_3H_7AlO_4$
Glucametacin	Antiinflammatory	52443-21-7		$C_{25}H_{27}ClN_2O_8$
Gluconolactone	Antidote	90-80-2		$C_6H_{10}O_6$
Glucosamine	Antirheumatic	3416-24-8	Y	$C_6H_{13}NO_5$
Glucosulfamide	Antibiotic	500-74-3		$C_{13}H_{22}N_2O_{11}S_2$
Glucosulfone	Antibiotic	551-89-3		$C_{24}H_{36}N_2O_{18}S_3$
Glucurolactone	Unclassified	32449-92-6		$C_6H_8O_6$
Glucuronamide	Antibiotic	3789-97-7		$C_6H_{11}NO_6$
Glufosfamide	Antineoplastic	132682-98-5		$C_{10}H_{21}Cl_2N_2O_7P$
Glunicate	Antihyperlipidemic	80763-86-6		$C_{36}H_{28}N_6O_{10}$
Glutamic Acid	Anticonvulsant	56-86-0		$C_5H_9NO_4$
Glutaurine	Anticonvulsant	56488-60-9		$C_7H_{14}N_2O_6S$
Glutethimide	Sedative	77-21-4		$C_{13}H_{15}NO_2$
Glyburide	Antidiabetic	10238-21-8	Y	$C_{23}H_{28}ClN_3O_5S$
Glybuthiazol	Antidiabetic	535-65-9		$C_{12}H_{16}N_4O_2S_2$
Glybuzole	Antidiabetic	1492-02-0		$C_{12}H_{15}N_3O_2S_2$
Glyclopyramide	Antiarrhythmic	631-27-6		$C_{11}H_{14}ClN_3O_3S$
Glycobiarsol	Antiamebic	775514-86-8		$C_8H_9AsBiNO_6$
Glycocyamine	Cardiotonic	352-97-6		$C_3H_7N_3O_2$
Glycopyrrolate	Antispasmodic	596-51-0	Y	$C_{19}H_{28}BrNO_3$
Glycyclamide	Antitussive	664-95-9		$C_{14}H_{20}N_2O_3S$
Glycyrrhizin	Antiinflammatory	1405-86-3		$C_{42}H_{62}O_{16}$
Glyhexamide	Antidiabetic	451-71-8		$C_{16}H_{22}N_2O_3S$
Glymidine Sodium	Antidiabetic	339-44-6		$C_{13}H_{15}N_3O_4S$
Glyoctamide	Antidiabetic	1038-59-1		$C_{16}H_{24}N_2O_3S$
Glyparamide	Antidiabetic	5581-42-0		$C_{15}H_{16}ClN_3O_3S$
Glypinamide	Unclassified	1228-19-9		$C_{13}H_{18}ClN_3O_3S$
Glysobuzole	Antidiabetic	3567-08-6		$C_{13}H_{17}N_3O_3S_2$
Goralatide	Antihypertensive	120081-14-3		$C_{20}H_{33}N_5O_9$
Goxalapladib	Antihyperlipidemic	412950-27-7		$C_{40}H_{39}F_5N_4O_3$
Granisetron	Antiemetic	109889-09-0	Y	$C_{18}H_{21}D_3N_4O$
Grepafloxacin	Antibiotic	119914-60-2	Y	$C_{19}H_{22}FN_3O_3$
Griseofulvin	Antifungal	126-07-8	Y	$C_{17}H_{17}ClO_6$
Guabenxan	Antihypertensive	19889-45-3	Y	$C_{10}H_{13}N_3O_2$
Guacetisal	Analgesic	55482-89-8		$C_{16}H_{14}O_5$
Guafecainol	Antiarrhythmic	36199-78-7		$C_{16}H_{27}NO_4$
Guaiacol	Expectorant	90-05-1		$C_7H_8O_2$
Guaiacol Carbonate	Expectorant	553-17-3		$C_{15}H_{14}O_5$
Guaiactamine	Expectorant	15687-23-7		$C_{13}H_{21}NO_2$
Guaiapate	Antitussive	852-42-6		$C_{18}H_{29}NO_4$
Guaietolin	Expectorant	63834-83-3		$C_{11}H_{16}O_4$
Guaifenesin	Expectorant	93-14-1	Y	$C_{10}H_{14}O_4$
Guaimesal	Expectorant	81674-79-5		$C_{16}H_{14}O_5$
Guaisteine	Cardiotonic	103181-72-2		$C_{15}H_{19}$NO4S2
Guamecycline	Antibiotic	16545-11-2		$C_{29}H_{38}N_8O_8$
Guanabenz	Antihypertensive	5051-62-7		C_8H_8Cl2N4
Guanacline Sulfate	Antihypertensive	1463-28-1		$C_9H_{18}N_4$
Guanadrel	Antihypertensive	40580-59-4	Y	$C_{10}H_{19}$N3O2
Guanazodine	Antihypertensive	32059-15-7		$C_9H_{20}N_4$

Table S1. *Cont.*

Common Name	Indication	CAS Number	Oral	Molecular Formula
Guanclofine	Unclassified	55926-23-3		$C_9H_{12}Cl_2N_4$
Guancydine	Antihypertensive	1113-10-6		$C_7H_{14}N_4$
Guanethidine	Antihypertensive	55-65-2	Y	$C_{10}H_{22}N_4$
Guanfacine	Antihypertensive	29110-47-2	Y	$C_9H_9Cl_2N_3O$
Guanidine	Cholinergic	113-00-8		CH_5N_3
Guanisoquin Sulfate	Antihypertensive	154-73-4		$C_{10}H_{12}BrN_3$
Guanoclor Sulfate	Antihypertensive	5001-32-1		$C_9H_{12}Cl_2N_4O$
Guanoctine	Antihypertensive	3658-25-1		$C_9H_{21}N_3$
Guanoxabenz	Antihypertensive	24047-25-4		$C_8H_8Cl_2N_4O$
Guanoxan	Antihypertensive	2165-19-7		$C_{10}H_{13}N_3O_2$
Guanoxyfen Sulfate	Antihypertensive	13050-83-4		$C_{10}H_{15}N_3O$
Gusperimus Trihydrochloride	Immunosuppressant	98629-43-7		$C_{17}H_{37}N_7O_3$
Halazepam	Sedative	23092-17-3	Y	$C_{17}H_{12}ClF_3N_2O$
Halcinonide	Antiinflammatory	3093-35-4		$C_{24}H_{32}ClFO_5$
Halobetasol Propionate	Antiinflammatory	66852-54-8		$C_{25}H_{31}ClF_2O_5$
Halocortolone	Glucocorticoid	24320-27-2		$C_{22}H_{27}ClF_2O_3$
Halofantrine	Antimalarial	69756-53-2	Y	$C_{26}H_{30}Cl_2F_3NO$
Halofenate	Uricosuric	26718-25-2		$C_{19}H_{17}ClF_3NO_4$
Halofuginone	Antiprotozoal	55837-20-2		$C_{16}H_{17}BrClN_3O_3$
Halometasone	Antiinflammatory	50629-82-8		$C_{22}H_{27}ClF_2O_5$
Halonamine	Antibiotic	50583-06-7		$C_{15}H_{15}ClFNO$
Halopemide	Antipsychotic	59831-65-1		$C_{21}H_{22}ClFN_4O_2$
Halopenium Chloride	Unclassified	7008-13-1		$C_{22}H_{30}BrCl_2NO$
Haloperidol	Antispasmodic	52-86-8	Y	$C_{21}H_{23}ClFNO_2$
Haloperidol Decanoate	Antipsychotic	74050-97-8		$C_{31}H_{41}ClFNO_3$
Halopredone Acetate	Antiinflammatory	57781-14-3		$C_{25}H_{29}BrF_2O_7$
Haloprogesterone	Progestogen	3538-57-6		$C_{21}H_{28}BrFO_2$
Haloprogin	Antibacterial	777-11-7		$C_9H_4Cl_3IO$
Halothane	Anesthetic	151-67-7		$C_2HBrClF_3$
Haloxazolam	Sedative	59128-97-1		$C_{17}H_{14}BrFN_2O_2$
Haloxon	Anthelminthic	321-55-1		$C_{14}H_{14}Cl_3O_6P$
Hepronicate	Vasodilator	7237-81-2		C_{28}H31N3O6
Heptabarbital	Sedative	509-86-4		$C_{13}H_{18}N_2O_3$
Heptaminol	Antihypotensive	372-66-7		$C_8H_{19}NO$
Heptaverine	Antispasmodic	54063-48-8		$C_{18}H_{25}N$
Heptolamide	Antidepressant	1034-82-8		$C_{15}H_{22}N_2O_3S$
Hepzidine	Antidepressant	1096-72-6		$C_{21}H_{25}NO$
Hetacillin	Antibiotic	3511-16-8		$C_{19}H_{23}N_3O_4S$
Heteronium Bromide	Mydriatic	7247-57-6		$C_{18}H_{22}BrNO_3S$
Hexacyclonate Sodium	Nootropic	7491-42-1		$C_9H_{16}O_3$
Hexacyprone	Mydriatic	892-01-3		$C_{16}H_{20}O_3$
Hexadiline	Vasodilator	3626-67-3		$C_{19}H_{33}N$
Hexafluorenium	Muscle Relaxant	4844-10-4		$C_{36}H_{42}Br_2N_2$
Hexamethonium Bromide	Antihypertensive	55-97-0		$C_{12}H_{30}Br_2N_2$
Hexamidine	Antiulcerative	125-33-7		$C_{12}H_{14}N_2O_2$
Hexapradol	Unclassified	15599-37-8		$C_{19}H_{25}NO$
Hexaprofen	Antiinflammatory	24645-20-3		$C_{15}H_{20}O_2$
Hexapropymate	Sedative	358-52-1		$C_{10}H_{15}NO_2$
Hexasonium Iodide	Antispasmodic	25330-82-9		$C_{18}H_{27}IO_2S$
Hexazole	Unclassified	4671-03-8		$C_{10}H_{17}N_3$
Hexcarbacholine Bromide	Muscle Relaxant	306-41-2		$C_{20}H_{26}N_4O_2$
Hexedine	Antibacterial	5980-31-4		$C_{22}H_{45}N_3$
Hexestrol	Estrogen	84-16-2		$C_{18}H_{22}O_2$
Hexobarbital	Sedative	56-29-1		$C_{12}H_{16}N_2O_3$
Hexobendine	Vasodilator	54-03-5		C_{30}H44N2O10
Hexoprenaline	Bronchodilator	3215-70-1		$C_{22}H_{32}N_2O_6$
Hexylcaine	Anesthetic	532-77-4		$C_{16}H_{23}NO_2$
Hexylresorcinol	Anthelminithic	136-77-6		$C_{12}H_{18}O_2$
Histamine	Antineoplastic	51-45-6		$C_5H_9N_3$
Histapyrrodine	Antihistaminic	493-80-1		$C_{19}H_{24}N_2$
Homarylamine	Muscle Relaxant	451-77-4		$C_{10}H_{13}NO_2$
Homatropine Hydrobromide	Mydriatic	87-00-3		$C_{16}H_{21}NO_3$
Homatropine Methylbromide	Mydriatic	31610-86-3	Y	$C_{17}H_{24}BrNO_3$

Table S1. *Cont.*

Common Name	Indication	CAS Number	Oral	Molecular Formula
Homidium Bromide	Anthelminithic	1239-45-8		$C_{21}H_{20}BrN_3$
Homochlorcyclizine	Antihistaminic	848-53-3		$C_{19}H_{23}ClN_2$
Homofenazine	Sedative	3833-99-6		$C_{23}H_{28}F_3N_3OS$
Homopipramol	Antidepressant	35142-68-8		$C_{24}H_{31}N_3O$
Homosalate	Dermatologic	118-56-9		$C_{16}H_{22}O_3$
Homprenorphine	Analgesic	16549-56-7		$C_{28}H_{37}NO_4$
Hopantenic Acid	Nootropic	18679-90-8		$C_{10}H_{19}NO_5$
Hoquizil	Bronchodilator	21560-59-8		$C_{19}H_{26}N_4O_5$
Hycanthone	Anthelminithic	3105-97-3		$C_{20}H_{24}N_2O_2S$
Hydracarbazine	Diuretic	3614-47-9		$C_5H_7N_5O$
Hydralazine	Antihypertensive	86-54-4	Y	$C_8H_8N_4$
Hydrastine	Cardiotonic	118-08-1		$C_{21}H_{21}NO_6$
Hydrastinine	Cardiotonic	6592-85-4		C11H13NO3
Hydrobentizide	Diuretic	13957-38-5		$C_{15}H_{16}ClN_3O_4S_3$
Hydrochlorothiazide	Diuretic	58-93-5	Y	$C_7H_8ClN_3O_4S_2$
Hydrocodone	Antitussive	125-29-1	Y	$C_{18}H_{21}NO_3$
Hydrocortamate	Glucocorticoid	76-47-1		$C_{27}H_{41}NO_6$
Hydrocortisone	Glucocorticoid	50-23-7	Y	$C_{21}H_{30}O_5$
Hydrocortisone Aceponate	Glucocorticoid	74050-20-7		$C_{26}H_{36}O_7$
Hydrocortisone Acetate	Glucocorticoid	50-03-3		$C_{23}H_{32}O_6$
Hydrocortisone Buteprate	Glucocorticoid	72590-77-3		$C_{28}H_{40}O_7$
Hydrocortisone Butyrate	Glucocorticoid	13609-67-1		$C_{25}H_{36}O_6$
Hydrocortisone Cypionate	Glucocorticoid	508-99-6		$C_{29}H_{42}O_6$
Hydrocortisone Hemisuccinate	Glucocorticoid	2203-97-6		$C_{25}H_{34}O_8$
Hydrocortisone Sodium Phosphate	Glucocorticoid	3863-59-0		$C_{21}H_{31}O_8P$
Hydrocortisone Tebutate	Glucocorticoid	508-96-3		$C_{27}H_{40}O_6$
Hydrocortisone Valerate	Glucocorticoid	57524-89-7		$C_{26}H_{38}O_6$
Hydrocotarnine	Hemostatic	550-10-7		$C_{12}H_{15}NO_3$
Hydroflumethiazide	Antihypertensive	135-09-1	Y	$C_8H_8F_3N_3O_4S_2$
Hydromadinone	Progestogen	16469-74-2		$C_{21}H_{29}ClO_3$
Hydromorphinol	Analgesic	2183-56-4		$C_{17}H_{21}NO_4$
Hydromorphone	Analgesic	466-99-9	Y	$C_{17}H_{19}NO_3$
Hydroxindasate	Unclassified	7008-14-2		$C_{21}H_{24}N_2O_3$
Hydroxindasol	Unclassified	7008-15-3		$C_{19}H_{22}N_2O_2$
Hydroxyamphetamine	Adrenergic	103-86-6		$C_9H_{13}NO$
Hydroxychloroquine	Antimalarial	118-42-3	Y	$C_{18}H_{26}ClN_3O$
Hydroxydione Sodium Succinate	Anesthetic	80-96-6		$C_{25}H_{36}O_6$
Hydroxyhexamide	Antidiabetic	3168-01-2		$C_{15}H_{22}N_2O_4S$
Hydroxypethidine	Analgesic	468-56-4		$C_{15}H_{21}NO_3$
Hydroxyphenamate	Anxiolytic	50-19-1		$C_{11}H_{15}NO_3$
Hydroxyprocaine	Analgesic	487-53-6		$C_{13}H_{20}N_2O_3$
Hydroxyprogesterone	Progestogen	68-96-2		$C_{21}H_{30}O_3$
Hydroxyprogesterone Caproate	Progestogen	630-56-8		$C_{27}H_{40}O_4$
Hydroxypyridine Tartrate	Analgesic	7008-17-5		$C_9H_9NO_6$
Hydroxystenozole	Unclassified	19120-01-5		$C_{21}H_{30}N_2O$
Hydroxystilbamidine	Antiprotozoal	495-99-8		$C_{16}H_{16}N_4O$
Hydroxytetracaine	Anesthetic	490-98-2		$C_{15}H_{24}N_2O_3$
Hydroxytoluic Acid	Antiinflammatory	83-40-9		$C_8H_8O_3$
Hydroxyurea	Antineoplastic	127-07-1	Y	$CH_4N_2O_2$
Hydroxyzine	Anxiolytic	68-88-2	Y	$C_{21}H_{27}ClN_2O_2$
Hydroxyzine Pamoate	Anxiolytic	130-85-8		$C_{23}H_{16}O_6$
Hymecromone	Choleretic	90-33-5		$C_{10}H_8O_3$
Hyoscyamine	Antispasmodic	101-31-5		$C_{17}H_{23}NO_3$
Hypericin	Antidepressant	548-04-9		$C_{30H16O8}$
Ibacitabine	Antiviral	611-53-0		$C_9H_{12}IN_3O_4$
Ibafloxacin	Antibacterial	91618-36-9		$C_{15}H_{14}FNO_3$
Ibandronate	Bone Resorption Inhibitor	114084-78-5		$C_9H_{23}NO_7P_2$
Ibazocine	Analgesic	57653-28-8		$C_{20}H_{29}NO$
Ibopamine	Cardiotonic	66195-31-1	Y	$C_{17}H_{25}NO_4$
Ibrolipim	Antiobesity	133208-93-2		$C_{19}H_{20}BrN_2O_4P$
Ibrotamide	Sedative	466-14-8		$C_7H_{14}BrNO$
Ibudilast	Vasodilator	50847-11-5	Y	$C_{14}H_{18}N_2O$
Ibufenac	Analgesic	1553-60-2		$C_{12}H_{16}O_2$

Table S1. *Cont.*

Common Name	Indication	CAS Number	Oral	Molecular Formula
Ibuprofen	Antiinflammatory	15687-27-1	Y	$C_{13}H_{18}O_2$
Ibuprofen Piconol	Antiinflammatory	64622-45-3		$C_{19}H_{22}NO_2$
Ibuproxam	Antiinflammatory	53648-05-8		$C_{13}H_{19}NO_2$
Ibutamoren Mesylate	Pituitary	159634-47-6		$C_{27}H_{36}N_4O_5S$
Ibuterol	Bronchodilator	53034-85-8		C20H31NO5
Ibutilide	Antiarrhythmic	122647-31-8		$C_{20}H_{36}N_2O_3S$
Ibuverine	Antispasmodic	31221-85-9		$C_{18}H_{26}O_3$
Iclaprim	Antibacterial	192314-93-5		$C_{19}H_{22}N_4O_3$
Iclazepam	Anxiolytic	57916-70-8		$C_{21}H_{21}ClN_2O_2$
Icodulinum	Unclassified	138511-81-6		$C_{10}H_{10}N_2OS$
Icofungipen	Antifungal	198022-65-0		$C_7H_{11}NO_2$
Icometasone Enbutate	Steroid	103466-73-5		$C_{28}H_{37}ClO_7$
Icomucret	Unclassified	54845-95-3		$C_{20}H_{32}O_3$
Icopezil Maleate	Nootropic	145508-78-7		$C_{23}H_{25}N_3O_2$
Icosapent	Antihyperlipidemic	10417-94-4	Y	$C_{20}H_{30}O_2$
Icospiramide	Antiinflammatory	79449-99-3		$C_{28}H_{31}F_2N_5O_2$
Icotidine	Antihistaminic	71351-79-6		$C_{21}H_{25}N_5O_2$
Icrocaptide	Antiinflammatory	169543-49-1		$C_{21}H_{40}N_8O_5$
Idarubicin	Antineoplastic	58957-92-9	Y	$C_{26}H_{27}NO_9$
Idaverine	Antispasmodic	100927-13-7		$C_{24}H_{39}N_3O_3$
Idazoxan	Antiparkinsonian	79944-58-4		$C_{11}H_{12}N_2O_2$
Idebenone	Nootropic	58186-27-9	Y	$C_{19}H_{30}O_5$
Idenast	Bronchodilator	108674-88-0		$C_{28}H_{31}FN_4O_2$
Idoxifene	Antineoplastic	116057-75-1		$C_{28}H_{30}INO$
Idoxuridine	Antiviral	54-42-2		$C_9H_{11}IN_2O_5$
Idralfidine	Sedative	95668-38-5		$C_{11}H_{14}N_4O$
Idramantone	Unclassified	20098-14-0		$C_{10}H_{14}O_2$
Idrapril	Antihypertensive	127420-24-0		$C_{11}H_{18}N_2O_5$
Idremcinal	Gastroprokinetic	110480-13-2		$C_{39}H_{69}NO_{12}$
Idrocilamide	Muscle Relaxant	6961-46-2		$C_{11}H_{13}NO_2$
Idronoxil	Antineoplastic	81267-65-4	Y	$C_{15}H_{12}O_3$
Idropranolol	Antihypertensive	27581-02-8		$C_{16}H_{23}NO_2$
Ifenprodil	Vasodilator	23210-56-2	Y	$C_{21}H_{27}NO_2$
Iferanserin	Antihypertensive	58754-46-4		$C_{23}H_{28}N_2O$
Ifetroban	Antithrombotic	143443-90-7		$C_{25}H_{32}N_2O_5$
Ifosfamide	Antineoplastic	3778-73-2		$C_7H_{15Cl2N2O2P}$
Ifoxetine	Antidepressant	66208-11-5		$C_{13}H_{19}NO_2$
Iganidipine	Vasodilator	119687-33-1		$C_{28}H_{38}N_4O_6$
Igmesine	Antidepressant	140850-73-3		$C_{23}H_{29}N$
Iguratimod	Immunomodulator	123663-49-0	Y	$C_{17}H_{14}N_2O_6S$
Ilaprazole	Antiulcerative	172152-36-2	Y	$C_{19}H_{18}N_4O_2S$
Ilepcimide	Anticonvulsant	82857-82-7		$C_{15}H_{17}NO_3$
Iliparcil	Antithrombotic	137214-72-3		$C_{16}H_{18}O_6S$
Ilmofosine	Antineoplastic	83519-04-4		$C_{26}H_{56}NO_5PS$
Ilomastat	Opthalmic	142880-36-2		$C_{20}H_{28}N_4O_4$
Ilonidap	Antiinflammatory	135202-79-8		$C_{14}H_8ClFN_2O_3S$
Iloperidone	Antipsychotic	133454-47-4	Y	$C_{24}H_{27}FN_2O_4$
Iloprost	Antithrombotic	78919-13-8	Y	$C_{22}H_{32}O_4$
Imafen	Antidepressant	59198-18-4		$C_{11}H_{13}N_3$
Imanixil	Antihypertensive	75689-93-9		$C_{17}H_{17}F_3N_6O_2$
Imatinib	Antineoplastic	152459-95-5	Y	$C_{29}H_{31}N_7O$
Imazodan	Cardiotonic	84243-58-3		$C_{13}H_{12}N_4O$
Imcarbofos	Anthelminithic	66608-32-0		$C_{17}H_{30}N_4O_7P_2S_2$
Imexon	Immunomodulator	59643-91-3		$C_4H_5N_3O$
Imiclopazine	Antipsychotic	7224-08-0		$C_{25}H_{32}ClN_5OS$
Imidafenacin	Antidepressant	170105-16-5	Y	$C_{20}H_{21}N_3O$
Imidapril	Antihypertensive	89371-37-9	Y	$C_{20}H_{27}N_3O_6$
Imidaprilat	Antihypertensive	89371-44-8		$C_{18}H_{23}N_3O_6$
Imidazole Salicylate	Antiinflammatory	288-32-4		$C_3H_4N_2$
Imidocarb	Antiprotozoal	27885-92-3		$C_{19}H_{20}N_6O$
Imidoline	Antipsychotic	7303-78-8		$C_{13}H_{18}ClN_3O$
Imiglitazar	Antidiabetic	250601-04-8		$C_{28}H_{26}N_2O_5$
Imiloxan	Antidepressant	81167-16-0		$C_{14}H_{16}N_2O_2$

Table S1. *Cont.*

Common Name	Indication	CAS Number	Oral	Molecular Formula
Iminophenimide	Unclassified	7008-18-6		$C_{12}H_{14}N_2O_2$
Imipenem	Antibacterial	64221-86-9		$C_{12}H_{17}N_3O_4S$
Imipramine	Antidepressant	50-49-7	Y	$C_{19}H_{24}N_2$
Imipraminoxide	Antidepressant	6829-98-7		$C_{19}H_{24}N_2O$
Imiquimod	Antiviral	99011-02-6		$C_{14}H_{16}N_4$
Imirestat	Antidiabetic	89391-50-4		$C_{15}H_8F_2N_2O_2$
Imitrodast	Antiinflammatory	114686-12-3		C_{13}H12N2O2S
Imolamine	Antianginal	318-23-0		$C_{14}H_{20}$N4O
Imoxiterol	Bronchodilator	88578-07-8		$C_{20}H_{25}N_3O_3$
Impacarzine	Anthelminithic	41340-39-0		$C_{28}H_{55}N_5O_2$
Implitapide	Antihyperlipidemic	177469-96-4		$C_{35}H_{37}N_3O_2$
Improsulfan	Antineoplastic	13425-98-4		$C_8H_{19}NO_6S_2$
Imuracetam	Nootropic	67542-41-0		$C_{11}H_{18}N_4O_3$
Inaperisone	Muscle Relaxant	99323-21-4		$C_{16}H_{23}NO$
Incadronic Acid	Antineoplastic	124351-85-5		$C_8H_{19}NO_6P_2$
Incyclinide	Dermatologic	15866-90-7	Y	$C_{19}H_{17}NO_7$
Indacaterol	Bronchodilator	312753-06-3		$C_{24H28N2O3}$
Indacrinone	Antihypertensive	56049-88-8		$C_{18}H_{14}Cl_2O_4$
Indalpine	Antidepressant	63758-79-2		$C_{15}H_{20}N_2$
Indanazoline	Decongestant	40507-78-6		$C_{12}H_{15}N_3$
Indanidine	Antihypotensive	85392-79-6		$C_{11}H_{13}N_5$
Indanorex	Anorexic	16112-96-2		$C_{12}H_{17}NO$
Indapamide	Antihypertensive	26807-65-8	Y	$C_{16}H_{16}ClN_3O_3S$
Indatraline	Antipsychotic	86939-10-8		$C_{16}H_{15}Cl_2N$
Indecainide	Antiarrhythmic	74517-78-5		$C_{20}H_{24}N_2O$
Indeloxazine	Antidepressant	60929-23-9	Y	$C_{14}H_{17}NO_2$
Indenolol	Antiarrhythmic	60607-68-3	Y	$C_{15}H_{21}NO_2$
Indibulin	Antineoplastic	204205-90-3	Y	$C_{22}H_{16}ClN_3O_2$
Indinavir	Antiviral	150378-17-9	Y	$C_{36}H_{47}N_5O_4$
Indiplon	Sedative	325715-02-4		$C_{20}H_{16}N_4O_2S$
Indisetron	Antiemetic	141549-75-9	Y	$C_{17}H_{23}N_5O$
Indisulam	Antineoplastic	165668-41-7		$C_{14}H_{12}ClN_3O_4S_2$
Indobufen	Antithrombotic	63610-08-2		$C_{18}H_{17}NO_3$
Indocate	Estrogen	31386-25-1		$C_{22}H_{26}N_2O_2$
Indolapril	Antihypertensive	80876-01-3		$C_{24}H_{34}N_2O_5$
Indolidan	Cardiotonic	100643-96-7		$C_{14}H_{15}N_3O_2$
Indomethacin	Antiinflammatory	53-86-1	Y	$C_{19}H_{16}ClNO_4$
Indopanolol	Antianginal	69907-17-1		$C_{20}H_{23}ClN_2O_3$
Indopine	Urologic	3569-26-4		$C_{23}H_{28}N_2$
Indoprofen	Analgesic	31842-01-0		$C_{17}H_{15}NO_3$
Indoramin	Antihypertensive	26844-12-2		$C_{22}H_{25}N_3O$
Indorenate	Antihypertensive	73758-06-2		$C_{13}H_{16}N_2O_3$
Indoxole	Antiinflammatory	5034-76-4		$C_{22}H_{19}NO_2$
Indriline	Nootropic	7395-90-6		$C_{19}H_{21}N$
Inecalcitol	Bone Resorption Inhibitor	163217-09-2	Y	$C_{26}H_{40}O_3$
Ingliforib	Antidiabetic	186392-65-4		$C_{23}H_{24}ClN_3O_5$
Inicarone	Anticoagulant	39178-37-5		$C_{17}H_{15}NO_2$
Inocoterone Acetate	Dermatologic	83646-86-0		$C_{18}H_{26}O_3$
Inogatran	Antithrombotic	155415-08-0		$C_{21}H_{38}N_6O_4$
Inosine Pranobex	Antiviral	58-63-9	Y	$C_{10}H_{12}N_4O_5$
Inositol Niacinate	Vasodilator	6556-11-2		$C_{42}H_{30}N_6O_{12}$
Inproquone	Antiamebic	436-40-8		$C_{16}H_{22}N_2O_4$
Intermedine	Antineoplastic	10285-06-0		$C_{15}H_{25}NO_5$
Intoplicine	Antiarrhythmic	125974-72-3		$C_{21}H_{24}N_4O$
Intrazole	Antiinflammatory	15992-13-9		$C_{17}H_{12}ClN_5O$
Intriptyline	Antidepressant	27466-27-9		$C_{21}H_{19}N$
Iobutoic Acid	Choleretic	13445-12-0		$C_{15}H_{16}I_3NO_5$
Iodetryl	Unclassified	7008-02-8		$C_{20}H_{38}I_2O_2$
Iodobenzoic Acid	Unclassified	1321-07-9		$C_7H_5IO_2$
Iodocystine	Unclassified	1320-92-9		$C_6H_{11}IN_2O_4S_2$
Iodoform	Antibacterial	75-47-8		CHI_3
Iodophthalein Sodium	Laxative	386-17-4		$C_{20}H_{10}I_4O_4$
Iodoquinol	Antiamebic	83-73-8		$C_9H_5I_2NO$

Table S1. *Cont.*

Common Name	Indication	CAS Number	Oral	Molecular Formula
Iodothiouracil	Antineoplastic	5984-97-4		$C_4H_3IN_2OS$
Iosimide	Antiinflammatory	79211-10-2		$C_{21}H_{30}I_3N_3O_9$
Ipamorelin	Cardiotonic	170851-70-4		$C_{38}H_{49}N_9O_5$
Ipazilide Fumarate	Antiarrhythmic	115436-73-2		$C_{24}H_{30}N_4O$
Ipenoxazone	Antidepressant	104454-71-9		$C_{22}H_{34}N_2O_2$
Ipidacrine	Nootropic	62732-44-9		$C_{12}H_{16}N_2$
Ipragratine	Unclassified	22150-28-3		$C_{20}H_{29}NO_3$
Ipramidil	Vasodilator	83656-38-6		$C_{10}H_{16}N_4O_4$
Ipratropium	Bronchodilator	60205-81-4		$C_{20}H_{30}NO_3$
Ipravacaine	Analgesic	166181-63-1		$C_{18}H_{26}N_2O$
Iprazochrome	Hemostatic	7248-21-7		$C_{12}H_{16}N_4O_3$
Ipriflavone	Steroid	35212-22-7		$C_{18}H_{16}O_3$
Iprindole	Antidepressant	5560-72-5		$C_{19}H_{28}N_2$
Iproclozide	Antidepressant	3544-35-2		$C_{11}H_{15}ClN_2O_2$
Iprocrolol	Unclassified	37855-80-4		$C_{18}H_{21}NO_6$
Iproheptine	Antihistaminic	13946-02-6		$C_{11}H_{25}N$
Iproniazid	Antidepressant	54-92-2		$C_9H_{13}N_3O$
Ipronidazole	Antiprotozoal	14885-29-1		$C_7H_{11}N_3O_2$
Iprotiazem	Antihypertensive	105118-13-6		$C_{37}H_{49}N_3O_5S$
Iproxamine	Vasodilator	52403-19-7		$C_{18}H_{29}NO_4$
Iprozilamine	Unclassified	55477-19-5		$C_{13}H_{22}ClN_5S$
Ipsalazide	Antiinflammatory	623571-82-4		$C_{16}H_{13}N_3O_6$
Ipsapirone	Anxiolytic	95847-70-4		$C_{19}H_{23}N_5O_3S$
Iquindamine	Analgesic	55299-11-1		$C_{15}H_{23}N_3$
Iralukast	Bronchodilator	151581-24-7		$C_{38}H_{37}F_3O_8S$
Irampanel	Nootropic	206260-33-5		C18H19N3O2
Irbesartan	Antihypertensive	138402-11-6	Y	$C_{25}H_{28}N_6O$
Irindalone	Antihypertensive	96478-43-2		$C_{24}H_{29}FN_4O$
Irinotecan	Antineoplastic	97682-44-5		$C_{33}H_{38}N_4O_6$
Irloxacin	Antibiotic	91524-15-1		$C_{16}H_{13}FN_2O_3$
Irofulven	Antineoplastic	158440-71-2		$C_{15}H_{18}O_3$
Irolapride	Antiemetic	64779-98-2		$C_{19}H_{28}N_2O_3$
Iroxanadine	Antihistaminic	276690-58-5		$C_{14}H_{20}$N4O
Irsogladine	Antiulcerative	57381-26-7	Y	$C_9H_7Cl_2N_5$
Irtemazole	Uricosuric	129369-64-8		$C_{18}H_{16}N_4$
Isaglidole	Antidiabetic	110605-64-6		$C_{11}H_{13}FN_4$
Isalmadol	Analgesic	269079-62-1		$C_{22}H_{27}NO_4$
Isalsteine	Mucolytic	116818-99-6		$C_{14}H_{15}NO_6S$
Isamfazone	Antiinflammatory	55902-02-8		$C_{22}H_{23}N_3O_2$
Isamoltan	Antidepressant	55050-95-8		$C_{16}H_{22}N_2O_2$
Isamoxole	Bronchodilator	57067-46-6		$C_{12}H_{20}N_2O_2$
Isatoribine	Immunomodulator	122970-40-5		$C_{10}H_{12}N_4O_6S$
Isaxonine	Nootropic	4214-72-6		$C_7H_{11}N_3$
Isbogrel	Antithrombotic	89667-40-3		$C_{18}H_{19}NO_2$
Isbufylline	Bronchodilator	90162-60-0		$C_{11}H_{16}N_4O_2$
Isepamicin	Antibacterial	58152-03-7		$C_{22}H_{43}N_5O_{12}$
Isoaminile	Antitussive	77-51-0		$C_{16}H_{24}N_2$
Isobromindione	Anticoagulant	1470-35-5		$C_{15}H_9BrO_2$
Isobucaine	Analgesic	14055-89-1		$C_{15}H_{23}NO_2$
Isobutamben	Anesthetic	94-14-4		$C_{11}H_{15}NO_2$
Isocarboxazid	Antidepressant	59-63-2		$C_{12}H_{13}N_3O_2$
Isoconazole	Antibacterial	27523-40-6		$C_{18}H_{14}Cl_4N_2O$
Isocromil	Antihistaminic	57009-15-1		$C_{19}H_{16}O_5$
Isocyclamine	Unclassified	105-23-7		$C_8H_{17}N$
Isoetharine	Bronchodilator	530-08-5		$C_{13}H_{21}NO_3$
Isofezolac	Antiinflammatory	50270-33-2		$C_{23}H_{18}N_2O_2$
Isoflupredone Acetate	Antiinflammatory	338-98-7		$C_{23}H_{29}FO_6$
Isoflurane	Anesthetic	26675-46-7		C3H2ClF5O
Isoflurophate	Cholinergic	55-91-4		C_6H_{14}FO3P
Isomazole	Cardiotonic	86315-52-8		$C_{14}H_{13}N_3O_2S$
Isometamidium Chloride	Anthelminithic	34301-55-8		$C_{28}H_{26}ClN_7$
Isomethadone	Analgesic	466-40-0		$C_{21}H_{27}NO$

Table S1. *Cont.*

Common Name	Indication	CAS Number	Oral	Molecular Formula
Isometheptene	Antimigraine	503-01-5		$C_9H_{19}N$
Isomolpan	Antipsychotic	107320-86-5		$C_{15}H_{21}NO_2$
Isomylamine	Muscle Relaxant	28815-27-2		$C_{18}H_{35}NO_2$
Isoniazid	Antibiotic	54-85-3	Y	$C_6H_7N_3O$
Isonixin	Analgesic	57021-61-1		$C_{14}H_{14}N_2O_2$
Isoprazone	Analgesic	56463-68-4		$C_{15}H_{18}N_2O$
Isoprednidene	Glucocorticoid	17332-61-5		C22H28O5
Isoprofen	Antiinflammatory	57144-56-6		$C_{15}H_{20}O_2$
Isopropamide	Antispasmodic	7492-32-2		$C_{23}H_{33}N_2O$
Isopropicillin	Antibiotic	4780-24-9		$C_{18}H_{22}N_2$O5S
Isoproterenol	Bronchodilator	7683-59-2		$C_{11}H_{17}NO_3$
Isosorbide	Antianginal	652-67-5		$C_6H_{10}O_4$
Isosorbide Dinitrate	Antianginal	87-33-2	Y	$C_6H_8N_2O_8$
Isosorbide Mononitrate	Vasodilator	16051-77-7		$C_6H_9NO_6$
Isospaglumic Acid	Nootropic	3106-85-2		$C_{11}H_{16}N_2O_8$
Isosulpride	Antipsychotic	42792-26-7		$C_{15}H_{23}N_3O_4S$
Isothipendyl	Antihistaminic	482-15-5		$C_{16}H_{19}N_3S$
Isotiquimide	Antiulcerative	56717-18-1		$C_{11}H_{14}N_2S$
Isotretinoin	Dermatologic	4759-48-2	Y	$C_{20}H_{28}O_2$
Isotretinoin Anisatil	Dermatologic	127471-94-7		$C_{29}H_{36}O_4$
Isovaleramide	Anxiolytic	541-46-8		$C_5H_{11}NO$
Isoxaprolol	Opthalmic	75949-60-9		$C_{19}H_{26}N_2O_3$
Isoxepac	Antiinflammatory	55453-87-7		$C_{16}H_{12}O_4$
Isoxicam	Antiinflammatory	34552-84-6		$C_{14}H_{13}N_3O_5S$
Isoxsuprine	Vasodilator	395-28-8		$C_{18}H_{23}NO_3$
Ispinesib Mesylate	Antineoplastic	336113-53-2		$C_{30}H_{33}$ClN4O2
Ispronicline	Analgesic	252870-53-4	Y	$C_{14}H_{22}N_2O$
Isradipine	Antihypertensive	75695-93-1	Y	$C_{19}H_{21}N_3O_5$
Israpafant	Antithrombotic	117279-73-9		$C_{28}H_{29}ClN_4S$
Istaroxime	Antiviral	203737-93-3		$C_{21}H_{32}N_2O_3$
Istradefylline	Antiparkinsonian	155270-99-8	Y	$C_{20}H_{24}N_4O_4$
Itameline	Antipsychotic	145071-44-9	Y	$C_{14}H_{15}ClN_2O_3$
Itanoxone	Analgesic	58182-63-1		$C_{17}H_{13}ClO_3$
Itasetron	Antidepressant	123258-84-4		$C_{16}H_{20}N_4O_2$
Itazigrel	Anticoagulant	70529-35-0		$C_{18}H_{14}F_3NO_2S$
Itopride	Gastroprokinetic	122898-67-3		$C_{20}H_{26}N_2O_4$
Itraconazole	Antifungal	84625-61-6	Y	$C_{35}H_{38}Cl_2N_8O_4$
Itriglumide	Gastroprokinetic	201605-51-8		$C_{33}H_{38}N_2O_4$
Itrocainide	Antiarrhythmic	90828-99-2		$C_{23}H_{27}N_3O$
Itrocinonide	Steroid	106033-96-9		$C_{29}H_{38}F_2O_9$
Ivabradine	Antihypotensive	155974-00-8	Y	$C_{27}H_{36}N_2O_5$
Ivarimod	Antiarrhythmic	53003-81-9		$C_{30}H_{44}N_2O_5$
Ivoqualine	Anxiolytic	72714-75-1		$C_{20}H_{26}N_2O$
Ixabepilone	Antineoplastic	219989-84-1		$C_{27}H_{42}N_2O_5S$
Izonsteride	Antineoplastic	176975-26-1		$C_{24}H_{26}N_2OS_2$
Josamycin	Antibiotic	16846-24-5	Y	$C_{42}H_{69}NO_{15}$
Kainic Acid	Anthelminithic	487-79-6		C10H15NO4
Kalafungin	Antifungal	11048-15-0		$C_{16}H_{12}O_6$
Kanamycin Sulfate	Antibiotic	59-01-8		$C_{18}H_{36}N_4O_{11}$
Kebuzone	Antirheumatic	853-34-9		$C_{19}H_{18}N_2O_3$
Keracyanin	Unclassified	28338-59-2		$C_{27}H_{31}ClO_{15}$
Ketamine	Anesthetic	6740-88-1		$C_{13}H_{16}ClNO$
Ketanserin	Antihypertensive	74050-98-9		$C_{22}H_{22}FN_3O_3$
Ketazocine	Analgesic	36292-69-0		$C_{18}H_{23}NO_2$
Ketazolam	Anxiolytic	27223-35-4	Y	$C_{20}H_{17}ClN_2O_3$
Kethoxal	Antiviral	27762-78-3		$C_6H_{12}O_4$
Ketipramine Fumarate	Antidepressant	796-29-2		$C_{19}H_{22}N_2O$
Ketobemidone	Analgesic	469-79-4		$C_{15}H_{21}NO_2$
Ketocaine	Analgesic	1092-46-2		$C_{18}H_{29}NO_2$
Ketocainol	Antiarrhythmic	7488-92-8		$C_{18}H_{31}NO_2$
Ketoconazole	Antifungal	65277-42-1	Y	$C_{26}H_{28}Cl_2N_4O_4$
Ketoprofen	Antiinflammatory	22071-15-4	Y	$C_{16}H_{14}O_3$

Table S1. *Cont.*

Common Name	Indication	CAS Number	Oral	Molecular Formula
Ketorfanol	Analgesic	79798-39-3		$C_{20}H_{25}NO_2$
Ketorolac	Analgesic	74103-06-3	Y	$C_{15}H_{13}NO_3$
Ketotifen	Antihistaminic	34580-13-7	Y	$C_{19}H_{19}NOS$
Ketotrexate	Antineoplastic	52196-22-2		$C_{21}H_{27}N_7O6$
Khellin	Vasodilator	82-02-0		$C_{14}H_{12}O5$
Khelloside	Antihyperlipidemic	17226-75-4		$C_{19}H_{20}O_{10}$
Kitasamycin	Antibacterial	1392-21-8		$C_{35}H_{59}NO_{13}$
Labetalol	Antihypertensive	36894-69-6	Y	$C_{19}H_{24N2O3}$
Lachesine Chloride	Unclassified	1164-38-1		$C_{20}H_{26}ClNO_3$
Lacidipine	Antihypertensive	103890-78-4	Y	$C_{26}H_{33}NO_6$
Lacosamide	Antiprotozoal	175481-36-4	Y	$C_{13}H_{18}N_2O_3$
Lactitol	Laxative	585-86-4		$C_{12}H_{24}O_{11}$
Lactulose	Laxative	4618-18-2	Y	$C_{12}H_{22}O_{11}$
Ladirubicin	Antineoplastic	171047-47-5		$C_{29}H_{31}NO_{11}S$
Ladostigil Tartrate	Nootropic	209394-27-4		$C_{16}H_{20}N_2O_2$
Laflunimus	Immunomodulator	147076-36-6		$C_{15}H_{13}F_3N_2O_2$
Lafutidine	Antiulcerative	118288-08-7	Y	$C_{22}H29N3O4S$
Lagatide	Unclassified	157476-77-2		$C_{33}H_{58}N_{10}O_9$
Laidlomycin Propionate Potassium	Antibiotic	78734-47-1		$C_{40}H_{66}O_{13}$
Lamifiban	Antithrombotic	144412-49-7		$C_{24}H_{28}N_4O_6$
Lamivudine	Antiviral	134678-17-4	Y	$C_8H_{11}N_3O_3S$
Lamotrigine	Anticonvulsant	84057-84-1	Y	$C_9H_7Cl_2N_5$
Lamtidine	Antiulcerative	73278-54-3		$C_{18}H_{28}N_{6O}$
Lanatoside C	Cardiotonic	17575-22-3		$C_{49}H_{76}O_{20}$
Landiolol	Antihypertensive	133242-30-5		$C_{25}H_{39}N_3O_8$
Lanepitant	Antiemetic	170566-84-4		$C_{33}H_{45}N_{5O3}$
Lanicemine	Nootropic	153322-05-5		$C_{13}H_{14N2}$
Laniquidar	Antineoplastic	197509-46-9		$C_{37}H_{36}N_4O_3$
Lanoconazole	Antifungal	101530-10-3		$C_{14}H_{10}ClN_3S_2$
Lanperisone	Muscle Relaxant	116287-14-0		$C_{15}H_{18}F_3NO$
Lanproston	Prostaglandin	105674-77-9		$C_{24}H_{31}ClO_7$
Lansoprazole	Antiulcerative	103577-45-3	Y	$C_{16}H_{14}F_3N_3O_2S$
Lantanoprost	Prostaglandin	1402923-77-6		$C_{30}H_{47}BO_5$
Lapatinib Ditosylate	Antineoplastic	231277-92-2	Y	$C_{29}H_{26}ClFN_4O_4S$
Lapisteride	Antineoplastic	142139-60-4		$C_{29}H_{40}N_2O_3$
Laprafylline	Bronchodilator	90749-32-9		$C_{29}H_{36}N_6O_2$
Laquinimod	Nootropic	248281-84-7	Y	$C_{19}H_{17}ClN_2O_3$
Lasalocid	Antibiotic	25999-31-9		$C_{34}H_{54}O_8$
Lasinavir	Antiviral	175385-62-3		$C_{35}H_{53}N_3O_9$
Lasofoxifene	Antineoplastic	180916-16-9		$C_{28}H_{31}NO_2$
Latanoprost	Antiglaucoma	130209-82-4		$C_{26}H_{40}O_5$
Latidectin	Antiprotozoal	926308-28-3		$C_{46}H_{61}NO_{11}$
Laurcetium Bromide	Unclassified	1794-75-8		$C_{18}H_{38}BrNO_2$
Laureth 10s	Contraceptive	13081-34-0		$C_{16}H_{34}O_2S$
Laurixamine	Sedative	7617-74-5		$C_{15}H_{33}NO$
Lauroguadine	Antiprotozoal	135-43-3		$C_{20}H_{36}N_6O$
Lavoltidine Succinate	Antiulcerative	76956-02-0		$C_{19}H_{29}N_5O_2$
Lazabemide	Antiparkinsonian	103878-84-8		$C_8H_{10}ClN_3O$
Lecimibide	Antihyperlipidemic	130804-35-2		$C_{34}H_{40}F_2N_4OS$
Lecozotan	Nootropic	434283-16-6		$C_{28}H_{29N5O3}$
Ledazerol	Antihypertensive	434283-16-6		$C_{11}H_{12}N_2O_2$
Ledoxantrone Trihydrochloride	Antineoplastic	113457-05-9		$C_{21}H_{27}N_5OS$
Lefetamine	Analgesic	7262-75-1		$C_{16}H_{19}N$
Leflunomide	Antirheumatic	75706-12-6	Y	$C_{12}H_9F_3N_2O_2$
Leiopyrrole	Unclassified	5633-16-9		$C_{23}H_{28}N_2O$
Lemidosul	Unclassified	88041-40-1		$C_{12}H_{19}NO_3S$
Lemildipine	Antiarrhythmic	94739-29-4		$C_{20}H_{22}Cl_2N_2O_6$
Leminoprazole	Antiulcerative	104340-86-5		$C_{19}H_{23}N_3OS$
Lemuteporfin	Antineoplastic	215808-49-4		$C_{44}H_{48}N_4O_{10}$
Lenalidomide	Immunomodulator	191732-72-6	Y	$C_{13}H_{13}N_3O_3$
Lenampicillin	Antibacterial	86273-18-9		$C_{21}H_{23}N_3O_7S$
Lenapenem	Antibacterial	149951-16-6		$C_{18}H_{29}N_3O_5S$
Leniquinsin	Antihypertensive	10351-50-5		$C_{20}H_{20}N_2O_4$

Table S1. *Cont.*

Common Name	Indication	CAS Number	Oral	Molecular Formula
Lenperone	Antipsychotic	24678-13-5		$C_{22}H_{23}F_2NO_2$
Leptacline	Antihypertensive	5005-72-1		$C_{12}H_{23}N$
Lercanidipine	Antihypertensive	100427-26-7	Y	$C_{36}H_{41}N_3O_6$
Lergotrile	Pituitary	36945-03-6		$C_{17}H_{18}ClN_3$
Lerisetron	Antiemetic	143257-98-1		$C_{18}H_{20}N_4$
Lesopitron	Anxiolytic	132449-46-8		$C_{15}H_{21}ClN_6$
Lestaurtinib	Antineoplastic	111358-88-4		$C_{26}H_{21}N_3O_4$
Leteprinim	Nootropic	138117-50-7		$C_{15}H_{13}N_5O_4$
Lethane Ethyl Thiocyanate)	Anesthetic	112-56-1		$C_9H_{17}NO_2S$
Letimide	Analgesic	26513-90-6		$C_{14}H_{18}N_2O_3$
Letosteine	Mucolytic	53943-88-7	Y	$C_{10}H_{17}NO_4S_2$
Letrazuril	Antibacterial	103337-74-2		$C_{17}H_9Cl_2FN_4O_2$
Letrozole	Antineoplastic	112809-51-5	Y	$C_{17}H_{11}N_5$
Leucinocaine	Analgesic	92-23-9		$C_{17}H_{28}N_2O_2$
Leucocianidol	Capillary Protectant	480-17-1		$C_{15}H_{14}O_7$
Leucovorin	Antidote	58-05-9		$C_{20}H_{23}N_7O_7$
Leurubicin	Antineoplastic	70774-25-3		$C_{33}H_{40}N_2O_{12}$
Levalbuterol	Bronchodilator	34391-04-3		$C_{13}H_{21}NO_3$
Levallorphan	Antidote	152-02-3		$C_{19}H_{25}NO$
Levamfetamine Succinate	Anorexic	156-34-3		$C_9H_{13}N$
Levamisole	Anthelminithic	14769-73-4		$C_{11}H_{12}N_2S$
Levcromakalim	Antihypertensive	94535-50-9		$C_{16}H_{18}N_2O_3$
Levcycloserine	Antibacterial	339-72-0		$C_3H_6N_2O_2$
Levdobutamine Lactobionate	Cardiotonic	61661-06-1		$C_{18}H_{23}NO_3$
Levdobutamine Lactobionate	Cardiotonic	96-82-2		$C_{12}H_{22}O_{12}$
Levemopamil	Antiarrhythmic	101238-51-1		$C_{23}H_{30}N_2$
Levetiracetam	Anticonvulsant	102767-28-2	Y	$C_8H_{14}N_2O_2$
Levisoprenaline	Bronchodilator	51-31-0		$C_{11}H_{17}NO_3$
Levlofexidine	Antihypertensive	81447-78-1		$C_{11}H_{12}Cl_2N_2O$
Levmetamfetamine	Decongestant	33817-09-3		$C_{10}H_{15}N$
Levobetaxolol	Antihypertensive	93221-48-8		$C_{18}H_{29}NO_3$
Levobunolol	Antiglaucoma	47141-42-4		$C_{17}H_{25}NO_3$
Levobupivacaine	Anesthetic	27262-47-1		$C_{18}H_{28}N_2O$
Levocabastine	Antihistaminic	79516-68-0		$C_{26}H_{29}FN_2O_2$
Levocetirizine	Antihistaminic	130018-77-8	Y	$C_{21}H_{25}ClN_2O_3$
Levodopa	Antiparkinsonian	59-92-7	Y	$C_9H_{11}NO4$
Levodropropizine	Antitussive	99291-25-5	Y	$C_{13}H_{20}N_2O_2$
Levofacetoperane	Anorexic	24558-01-8		$C_{14}H_{19}NO_2$
Levofenfluramine	Anorexic	37577-24-5		$C_{12}H_{16}F_3N$
Levofloxacin	Antibiotic	100986-85-4	Y	$C_{18}H_{20}FN_3O_4$
Levofuraltadone	Antibacterial	3795-88-8		$C_{13}H_{16}N_4O_6$
Levolansoprazole	Antiulcerative	138530-95-7		$C_{16}H_{14}F_3N_3O_2S$
Levoleucovorin Calcium	Antidote	68538-85-2	Y	$C_{20}H_{23}N_7O_7$
Levomenol	Antiarrhythmic	23089-26-1		$C_{15}H_{26}O$
Levomenthol	Dermatologic	2216-51-5		$C_{10}H_{20}O$
Levomepromazine	Analgesic	60-99-1		$C_{19}H_{24}N_2OS$
Levomethadone	Analgesic	125-58-6		$C_{21}H_{27}NO$
Levomethadyl Acetate	Analgesic	1477-40-3		$C_{23}H_{31}NO_2$
Levomethorphan	Antitussive	125-70-2		$C_{18}H_{25}NO$
Levometiomeprazine	Analgesic	1759-09-7		$C_{19}H_{24}N_2S_2$
Levomoprolol	Antihypertensive	77164-20-6		$C_{13}H_{21}NO_3$
Levomoramide	Analgesic	5666-11-5		$C_{25}H_{32}N_2O_2$
Levonantradol	Analgesic	71048-87-8		$C_{27}H_{35}NO_4$
Levonordefrin	Antihypertensive	829-74-3		$C_9H_{13}NO_3$
Levonorgestrel	Progestogen	797-63-7	Y	$C_{21}H_{28}O_2$
Levophenacylmorphan	Analgesic	10061-32-2		$C_{24}H_{27}NO_2$
Levopropoxyphene	Antitussive	2338-37-6		$C_{22}H_{29}NO_2$
Levopropylcillin	Antibacterial	3736-12-7		$C_{18}H_{22}N_2O5S$
Levopropylhexedrine	Adrenergic	6192-97-8		$C_{10}H_{21}N$
Levoprotiline	Antidepressant	76496-68-9		$C_{20}H_{23}NO$
Levormeloxifene	Estrogen	78994-23-7		$C_{30}H_{35}NO_3$
Levorphanol	Analgesic	77-07-6	Y	$C_{17}H_{23}NO$
Levosalbutamol	Bronchodilator	34391-04-3	Y	$C_{13}H_{21}NO_3$

Table S1. *Cont.*

Common Name	Indication	CAS Number	Oral	Molecular Formula
Levosemotiadil	Antiarrhythmic	116476-16-5		$C_{29}H_{32}N_2O_6S$
Levosimendan	Cardiotonic	141505-33-1		$C_{14}H_{12}N_6O$
Levosulpiride	Antidepressant	23672-07-3	Y	$C_{15}H_{23}N_3O_4S$
Levotofisopam	Anxiolytic	82059-51-6		$C_{22}H_{26}N_2O_4$
Levoxadrol	Anesthetic	4792-18-1		$C_{20}H_{23}NO_2$
Lexacalcitol	Dermatologic	131875-08-6		$C_{29}H_{48}O_4$
Lexipafant	Anticoagulant	139133-26-9		$C_{23}H_{30}N_4O_4S$
Lexithromycin	Antibacterial	53066-26-5		$C_{38}H_{70}N_2O_{13}$
Lexofenac	Antiinflammatory	41387-02-4		$C_{14}H_{14}O_3$
Liarozole	Antineoplastic	115575-11-6		$C_{17}H_{13}ClN_4$
Libecillide	Unclassified	27826-45-5		$C_{23}H_{32}N_4O_7S$
Libenzapril	Antihypertensive	109214-55-3		C18H25N3O5
Licarbazepine	Anticonvulsant	29331-92-8		$C_{15}H_{14}N_2O_2$
Licofelone	Antiinflammatory	156897-06-2		$C_{23}H_{22}ClNO_2$
Licostinel	Antipsychotic	153504-81-5		$C_8H_3Cl_2N_3O_4$
Lidadronic Acid	Bone Resorption Inhibitor	63132-38-7		$C_5H_{16}N_2O_6P_2$
Lidamidine	Antidiarrheal	66871-56-5		$C_{11}H_{16}N_4O$
Lidanserin	Anxiolytic	73725-85-6		$C_{26}H_{31}FN_2O_4$
Lidocaine	Anesthetic	137-58-6		$C_{14}H_{22}N_2O$
Lidoflazine	Vasodilator	3416-26-0		$C_{30}H_{35}F_2N_3O$
Lidorestat	Antidiabetic	245116-90-9		$C_{18}H_{11}F_3N_2O_2S$
Lifarizine	Anticoagulant	119514-66-8		$C_{29}H_{32}N_4$
Lifibrate	Antihyperlipidemic	22204-91-7		$C_{20}H_{21}Cl_2NO_4$
Lifibrol	Antihyperlipidemic	96609-16-4		$C_{21}H_{26}O_4$
Lilopristone	Abortifacient	97747-88-1		C29H37NO3
Limaprost	Prostaglandin	74397-12-9	Y	$C_{22}H_{36}O_5$
Limazocic	Hepatoprotectant	128620-82-6		$C_8H_{13}NO_3S_2$
Linaprazan	Antiulcerative	248919-64-4		$C_{21}H_{26}H_4O_2$
Linarotene	Dermatologic	127304-28-3		$C_{23}H_{30}N_2O_2S$
Lincomycin	Antibiotic	154-21-2		$C_{18}H_{34}N_2O_6S$
Lindane	Dermatologic	58-89-9		C6H6Cl6
Linetastine	Antihistaminic	159776-68-8		$C_{35}H_{40}N_2O_6$
Linezolid	Antibiotic	165800-03-3	Y	$C_{16}H_{20}FN_3O_4$
Linogliride	Antidiabetic	75358-37-1		$C_{16}H_{22}N_4O$
Linopirdine	Nootropic	105431-72-9		$C_{26}H_{21}N_3O$
Linotroban	Antithrombotic	120824-08-0		$C_{14}H_{15}NO_5S_2$
Linsidomine	Antianginal	33876-97-0	Y	$C_6H_{10}N_4O_2$
Lintitript	Gastroprokinetic	136381-85-6		$C_{20}H_{14}ClN_3O_3S$
Lintopride	Antipsychotic	107429-63-0		$C_{14}H_{19}ClN_4O_2$
Liothyronine	Thyroid	6893-02-3	Y	$C_{15}H_{12}I_3NO_4$
Lipoic Acid, Alpha	Antidote	1200-22-2		$C_8H_{14}O_2S_2$
Liranaftate	Antifungal	88678-31-3		$C_{18}H_{20}N_2O_2S$
Lirequinil	Sedative	143943-73-1		$C_{26}H_{25}ClN_2O_3$
Lirexapride	Antipsychotic	145414-12-6		$C_{24}H_{36}ClN_3O_2$
Lirimilast	Antiinflammatory	329306-27-6		$C_{17}H_{12}Cl_2N_2O_6S$
Liroldine	Mydriatic	105102-20-3		$C_{20}H_{20}F_2N_4$
Lisadimate	Dermatologic	136-44-7		$C_{10}H_{13}NO_4$
Lisdexamfetamine Dimesylate	Nootropic	608137-32-2	Y	$C_{15}H_{25}N_3O$
Lisinopril	Antihypertensive	76547-98-3	Y	$C_{21}H_{31}N_3O_5$
Lisofylline	Immunomodulator	100324-81-0		$C_{13}H_{20}N_4O_3$
Lisuride	Antimigraine	18016-80-3	Y	$C_{20}H_{26}N_4O$
Lithium Carbonate	Sedative	463-79-6	Y	CH_2O_3
Litomeglovir	Antiviral	321915-31-5		$C_{25}H_{30}N_4O_5S$
Litoxetine	Antidepressant	86811-09-8		$C_{16}H_{19}NO$
Litracen	Antidepressant	5118-30-9		$C_{20}H_{23}N$
Lixazinone Sulfate	Cardiotonic	94192-59-3		$C_{21}H_{28}N_4O_3$
Lixivaptan	Plasma Volume Expander	168079-32-1	Y	$C_{27}H_{21}ClFN_3O_2$
Lobeline	Respiratory Stimulant	90-69-7		$C_{22}H_{27}NO_2$
Lobendazole	Anthelminithic	6306-71-4		$C_{10}H_{11}N_3O_2$
Lobenzarit	Antirheumatic	63329-53-3	Y	$C_{14}H_{10}ClNO_4$
Lobucavir	Antiviral	127759-89-1		$C_{11}H_{15}N_5O_3$
Lobuprofen	Analgesic	98207-12-6		$C_{25}H_{33}ClN_2O_2$
Locicortolone Dicibate	Glucocorticoid	78467-68-2		$C_{36}H_{50}Cl_2O_5$

Table S1. *Cont.*

Common Name	Indication	CAS Number	Oral	Molecular Formula
Lodaxaprine	Antineoplastic	93181-81-8		$C_{15}H_{16}ClN_3O$
Lodazecar	Antihyperlipidemic	87646-83-1		$C_{22}H_{24}BrClN_4O_4$
Lodelaben	Antirheumatic	111149-90-7		$C_{25}H_{41}ClO_3$
Lodenosine	Antiviral	110143-10-7		$C_{10}H_{12}FN_5O_2$
Lodinixil	Antihypertensive	86627-50-1		$C_{14}H_{17}ClN_4$
Lodiperone	Adrenergic	72444-63-4		$C_{21}H_{20}Cl_2FN_3O_2$
Lodoxamide	Antihistaminic	53882-12-5		$C_{11}H_6ClN3O6$
Lofemizole	Antiinflammatory	65571-68-8		$C_{10}H9ClN2$
Lofendazam	Anxiolytic	29176-29-2		$C_{15}H_{13}ClN_2O$
Lofentanil Oxalate	Analgesic	61380-40-3		$C_{25}H_{32}N_2O_3$
Lofepramine	Antidepressant	23047-25-8	Y	$C_{26}H_{27}ClN_2O$
Lofexidine	Antihypertensive	31036-80-3	Y	$C_{11}H_{12}Cl_2N_2O$
Loflucarban	Antifungal	790-69-2		$C_{13}H_9Cl_2FN_2S$
Lombazole	Antibacterial	60628-98-0		$C_{22}H_{17}ClN_2$
Lomefloxacin	Antibiotic	98079-51-7		$C_{17}H_{19}F_2N_3O_3$
Lomeguatrib	Antineoplastic	192441-08-0		$C_{10}H_8BrN_5OS$
Lomerizine	Antimigraine	101477-55-8	Y	$C_{27}H_{30}F_2N_2O_3$
Lometraline	Antipsychotic	34910-85-5		$C_{13}H_{18}ClNO$
Lometrexol Sodium	Antineoplastic	106400-81-1		$C_{21}H_{25}N_5O_6$
Lomevactone	Antidote	81478-25-3		$C_{18}H_{17}ClO_2$
Lomifylline	Bronchodilator	10226-54-7		$C_{13}H_{18}N_4O_3$
Lomofungin	Antifungal	26786-84-5		$C_{15}H_{10}N_2O_6$
Lomustine	Antineoplastic	13010-47-4	Y	$C_9H_{16}ClN_3O_2$
Lonafarnib	Antineoplastic	193275-84-2		$C_{27}H_{31}Br_2ClN_4O_2$
Lonapalene	Dermatologic	91431-42-4		$C_{16}H_{15}ClO_6$
Lonaprofen	Analgesic	41791-49-5		$C_{14}H_{13}ClO_3$
Lonazolac	Antiinflammatory	53808-88-1		$C_{17}H_{13}ClN_2O_2$
Lonidamine	Antineoplastic	50264-69-2	Y	$C_{15}H_{10}Cl_2N_2O_2$
Loperamide	Antidiarrheal	53179-11-6	Y	$C_{29}H_{33}ClN_2O_2$
Loperamide Oxide	Antidiarrheal	106900-12-3		$C_{29}H_{33}ClN_2O_3$
Lopinavir	Antiviral	192725-17-0	Y	$C_{37}H_{48}N_4O_5$
Lopirazepam	Sedative	42863-81-0		C14H9Cl2N3O2
Loprazolam	Sedative	61197-73-7		$C_{23}H_{21}ClN_6O_3$
Loprodiol	Muscle Relaxant	2209-86-1		$C_5H_{10}Cl_2O_2$
Loracarbef	Antibiotic	76470-66-1	Y	$C_{16}H_{16}ClN_3O_4$
Lorajmine	Antiarrhythmic	47562-08-3		$C_{22}H_{27}ClN_2O_3$
Lorapride	Antipsychotic	68677-06-5		$C_{14}H_{22}ClN_3O_3S$
Loratadine	Antihistaminic	79794-75-5	Y	$C_{22}H_{23}ClN_2O_2$
Lorazepam	Anxiolytic	846-49-1	Y	$C_{15}H_{10}Cl_2N_2O_2$
Lorbamate	Muscle Relaxant	24353-88-6		$C_{12}H_{22}N_2O_4$
Lorcainide	Antiarrhythmic	59729-31-6		$C_{22}H_{27}ClN_2O$
Lorcinadol	Analgesic	104719-71-3		$C_{17}H_{19}ClN_4$
Loreclezole	Anticonvulsant	117857-45-1		$C_{10}H_6Cl_3N_3$
Lormetazepam	Sedative	848-75-9	Y	$C_{16}H_{12}Cl_2N_2O_2$
Lornoxicam	Analgesic	70374-39-9	Y	$C_{13}H_{10}ClN_3O_{4S2}$
Lorpiprazole	Antipsychotic	108785-69-9		$C_{21}H_{26}F_3N_5$
Lortalamine	Antidepressant	70384-91-7		$C_{15}H_{17}ClN_2O_2$
Lorzafone	Sedative	59179-95-2		$C_{18}H_{17}Cl2N3O3$
Losartan	Antihypertensive	114798-26-4	Y	$C_{22}H_{23}ClN_6O$
Losigamone	Anticonvulsant	112856-44-7		$C_{12}H_{11}ClO_4$
Losindole	Antidepressant	69175-77-5		$C_{19}H_{20}ClN$
Losmiprofen	Analgesic	74168-08-4		$C_{17}H_{15}ClO_4$
Losoxantrone	Antineoplastic	88303-60-0		$C_{22}H_{27}N_5O_4$
Losulazine	Antihypertensive	72141-57-2		$C_{27}H_{22}F_4N_4O_3S$
Loteprednol Etabonate	Antiinflammatory	82034-46-6		$C_{24}H_{31}ClO_7$
Lotifazole	Antiinflammatory	71119-10-3		$C_{12}H_9Cl_3N_2O_2S$
Lotrafiban	Antithrombotic	171049-14-2		$C_{23}H_{32}N_4O_4$
Lotrifen	Abortifacient	66535-86-2		$C_{16}H_{10}ClN_3$
Lotucaine	Anesthetic	42373-58-0		$C_{18}H_{29NO2}$
Lovastatin	Antihyperlipidemic	75330-75-5	Y	$C_{24}H_{36}O_5$
Loviride	Antiviral	147362-57-0		$C_{17}H_{16}Cl_2N_2O_2$
Loxanast	Unclassified	69915-62-4		$C_{14}H_{26}O_2$
Loxapine	Anxiolytic	1977-10-2	Y	$C_{18}H_{18}ClN_3O$

Table S1. *Cont.*

Common Name	Indication	CAS Number	Oral	Molecular Formula
Loxiglumide	Gastroprokinetic	107097-80-3		$C_{21}H_{30}Cl_2N_2O_5$
Loxoprofen	Antiinflammatory	68767-14-6	Y	$C_{15}H_{18}O_3$
Lozilurea	Antiulcerative	71475-35-9		$C_{10}H_{13}ClN_2O$
Lubazodone	Antidepressant	161178-07-0		$C_{14}H_{18}FNO_2$
Lubeluzole	Nootropic	144665-07-6		$C_{22}H_{25}F_2N_3O_2S$
Lubiprostone	Laxative	333963-40-9	Y	$C_{20}H_{32}F_2O_5$
Lucanthone	Anthelminithic	479-50-5		$C_{20}H_{24}N_2OS$
Lucartamide	Antimigraine	76743-10-7		$C_{12}H_{16}N_2S_2$
Lucimycin	Antibiotic	13058-67-8		$C_{36}H_{53}NO_{13}$
Lufenuron	Ectoparasiticide	103055-07-8		$C_{17}H_8Cl_2F_8N_2O_3$
Lufironil	Dermatologic	128075-79-6		$C_{13}H_{19}N_3O_4$
Lufuradom	Unclassified	94006-14-1		$C_{22}H_{20}FN_3O_2$
Luliconazole	Antifungal	187164-19-8		$C_{14}H_9Cl_2N_3S_2$
Lumefantrine	Antimalarial	82186-77-4		$C_{30}H_{32}Cl_3NO$
Lumiracoxib	Antirheumatic	220991-20-8	Y	$C_{15}H_{13}ClFNO_2$
Lupitidine	Antihistaminic	83903-06-4		$C_{21}H_{27}N_5O_2S$
Luprostiol	Prostaglandin	67110-79-6		$C_{21}H_{29}ClO_6S$
Lurasidone	Antipsychotic	367514-87-2	Y	$C_{28}H_{36}N_4O_2S$
Lurosetron Mesylate	Antiemetic	128486-54-4		$C_{17}H_{17}FN_4O$
Lurtotecan Dihydrochloride	Antineoplastic	149882-10-0		$C_{28}H_{30}N_4O_6$
Lusaperidone	Antipsychotic	214548-46-6		$C_{22}H_{21}N_3O_2$
Luxabendazole	Anthelminithic	90509-02-7		$C_{15}H_{12}FN_3O_5S$
Lycetamine	Antibacterial	60209-20-3		$C_{22}H_{47}N_3O$
Lydimycin	Antifungal	10118-85-1		$C_{10}H_{14}N_2O_3S$
Lymecycline	Antibiotic	992-21-2		C29H38N4O10
Lynestrenol	Progestogen	52-76-6		$C_{20}H_{28}O$
Lysergide	Antidepressant	50-37-3		$C_{20}H_{25}N_3O$
Mabuprofen	Analgesic	82821-47-4		$C_{15}H_{23}NO_2$
Mabuterol	Bronchodilator	56341-08-3		$C_{13}H_{18}ClF_3N_2O$
Maduramicin	Antibacterial	79356-08-4		$C_{47}H_{80}O_{17}$
Mafenide	Antibiotic	138-39-6		$C_7H_{10}N_2O_2S$
Mafoprazine	Antipsychotic	80428-29-1		$C_{22}H_{28}FN_3O_3$
Mafosfamide	Antineoplastic	88859-04-5		$C_9H_{19}Cl_2N_2O_5PS_2$
Malathion	Ectoparasiticide	121-75-5		$C_{10}H_{19}O_6PS_2$
Maleylsulfathiazole	Antibiotic	515-57-1		$C_{13}H_{11}N_3O_5$S2
Malotilate	Hepatic Protectant	59937-28-9		$C_{12}H_{16}O_4S_2$
Mandelic Acid	Dermatologic	90-64-2		$C_8H_8O_3$
Manidipine 6300	Vasodilator	89226-50-6		$C_{35}H_{38}N_4O_6$
Manifaxine	Antidepressant	135306-39-7		$C_{12}H_{15}F_2NO_2$
Manitimus	Immunomodulator	202057-76-9		$C_{15}H_{11}F_3N_2O_2$
Mannitol	Diuretic	69-65-8		$C_6H_{14}O_6$
Mannitol Hexanitrate	Vasodilator	15825-70-4		$C_6H_8N_6O_{18}$
Mannomustine	Antineoplastic	576-68-1		$C_{10}H_{22}Cl_2N_2O_4$
Mannosulfan	Antineoplastic	7518-35-6		$C_{10}H_{22}O_{14}S_4$
Manozodil	Unclassified	77528-67-7		$C_{10}H_{16}N_2S$
Mantabegron	Antidiabetic	36144-08-8		$C_{19}H_{27NO2}$
Mapinastine	Antihistaminic	140945-32-0		$C_{23}H_{34}N_6O$
Maprotiline	Antidepressant	10262-69-8	Y	$C_{20}H_{23}N$
Maraviroc	Antiviral	376348-65-1	Y	$C_{29}H_{41}F_2N_5O$
Marbofloxacin	Antibiotic	115550-35-1		$C_{17}H_{19}FN_4O_4$
Maribavir	Antiviral	176161-24-3	Y	C_{15}H19N3O4Cl2
Marimastat	Antineoplastic	154039-60-8		$C_{15}H_{29}N_3O_5$
Mariptiline	Antidepressant	60070-14-6		$C_{18}H_{18}N_2O$
Maropitant Citrate	Antiemetic	147116-67-4		$C_{32}H_{40}N_2O$
Maroxepin	Antidepressant	65509-24-2		$C_{19}H_{19}NO$
Masoprocol	Antineoplastic	27686-84-6		$C_{18}H_{22}O_4$
Mavacoxib	Antiinflammatory	170569-88-7		$C_{16}H_{11}F_4N_3O_2S$
Maxacalcitol	Dermatologic	103909-75-7		$C_{26}H_{42}O_4$
Maytansine	Antineoplastic	35846-53-8		$C_{34}H_{46}ClN_3O_{10}$
Mazapertine Succinate	Antipsychotic	134208-17-6		$C_{26}H_{35}N_3O_2$
Mazaticol	Antiparkinsonian	42024-98-6		$C_{21}H_{27}NO_3S_2$
Mazindol	Anorexic	22232-71-9	Y	$C_{16}H_{13}ClN_2O$
Mazipredone	Antiinflammatory	13085-08-0		$C_{26}H_{38}N_2O_4$

Table S1. *Cont.*

Common Name	Indication	CAS Number	Oral	Molecular Formula
Mazokalim	Antihypertensive	164178-54-5		$C_{23}H_{28}N_6O_6$
Mebanazine	Antipsychotic	65-64-5		$C_8H_{12}N_2$
Mebendazole	Anthelminithic	31431-39-7	Y	$C_{16}H_{13}N_3O_3$
Mebenoside	Capillary Protectant	55902-93-7		$C_{28}H_{32}O_6$
Mebeverine	Antispasmodic	3625-06-7	Y	$C_{25}H_{35}NO_5$
Mebezonium Iodide	Antitussive	7681-78-9		$C_{19}H_{40}I_2N_2$
Mebhydrolin	Antihistaminic	524-81-2		$C_{19}H_{20}N_2$
Mebiquine	Antidiarrheal	23910-07-8		$C_{10}H_{10}BiNO_3$
Mebolazine	Antianginal	3625-07-8		$C_{42}H_{68}N_2O_2$
Mebutamate	Antihypertensive	64-55-1		$C_{10}H_{20}N_2O_4$
Mebutizide	Diuretic	3568-00-1		$C_{13}H_{20}ClN_3O_4S_2$
Mecamylamine	Antihypertensive	60-40-2		$C_{11}H_{21}N$
Mecarbinate	Antihypertensive	15574-49-9		$C_{13}H_{15}NO_3$
Mechlorethamine	Antineoplastic	51-75-2		$C_5H_{11}Cl_2N$
Meciadanol	Antiulcerative	65350-86-9		$C_{16}H_{16}O_6$
Mecinarone	Vasodilator	26225-59-2		$C_{24}H_{27}NO_6$
Meclinertant	Antineoplastic	146362-70-1		$C_{32}H_{31}ClN_4O_5$
Meclizine	Antiemetic	569-65-3	Y	$C_{25}H_{27}ClN_2$
Meclocycline	Antibacterial	2013-58-3		$C_{22}H_{21}ClN_2O_8$
Meclofenamic Acid	Antiinflammatory	644-62-2		$C_{14}H_{11}Cl_2NO_2$
Meclofenoxate	Nootropic	51-68-3		$C_{12}H_{16}ClNO_3$
Meclonazepam	Sedative	58662-84-3		$C_{16}H_{12}ClN_3O_3$
Mecloqualone	Sedative	340-57-8		$C_{15}H_{11}ClN_2O$
Mecloralurea	Anxiolytic	1954-79-6		$C_4H_7Cl_3N_2O_2$
Meclorisone Dibutyrate	Antiinflammatory	10549-91-4		$C_{30}H_{40}Cl_2O_6$
Mecloxamine	Sedative	5668-06-4		$C_{19}H_{24}ClNO$
Mecrylate	Dermatologic	137-05-3		$C_5H_5NO_2$
Mecysteine	Mucolytic	2485-62-3		$C_4H_9NO_2S$
Medazepam	Sedative	2898-12-6	Y	$C_{16}H_{15}ClN_2$
Medazomide	Antitussive	300-22-1		$C_6H_9N_3O_2$
Medetomidine	Analgesic	86347-14-0		$C_{13}H_{16}N_2$
Medibazine	Vasodilator	53-31-6		$C_{25}H_{26}N_2O_2$
Medifoxamine	Antidepressant	32359-34-5		$C_{16}H_{19}NO_2$
Medorinone	Cardiotonic	88296-61-1		$C_9H_8N_2O$
Medorubicin	Antineoplastic	64314-52-9		$C_{26}H_{27}NO_{10}$
Medrogestone	Progestogen	977-79-7		$C_{23}H_{32}O_2$
Medroxalol	Antihypertensive	56290-94-9		$C_{20}H_{24}N_2O_5$
Medroxyprogesterone Acetate	Progestogen	71-58-9	Y	$C_{24}H_{34}O_4$
Medrylamine	Antihistaminic	524-99-2		$C_{18}H_{23}NO_2$
Medrysone	Glucocorticoid	2668-66-8		$C_{22}H_{32}O_3$
Mefeclorazine	Unclassified	1243-33-0		$C_{20}H_{25}ClN_2O_2$
Mefenamic Acid	Antiinflammatory	61-68-7	Y	$C_{15}H_{15}NO_2$
Mefenidil	Vasodilator	58261-91-9		$C_{12}H_{11}N_3$
Mefenidramium Metilsulfate	Unclassified	4858-60-0		$C_{19}H_{27}NO_5S$
Mefenorex	Anorexic	17243-57-1		$C_{12}H_{18}ClN$
Mefeserpine	Antihypertensive	3735-85-1		$C_{32}H_{38}N_2O_8$
Mefexamide	Nootropic	1227-61-8		$C_{15}H_{24}N_2O_3$
Mefloquine	Antimalarial	53230-10-7	Y	$C_{17}H_{16}F_6N_2O$
Mefruside	Diuretic	7195-27-9		$C_{13}H_{19}ClN_2O_5S_2$
Megalomicin Potassium Phosphate	Antibacterial	28022-11-9		$C_{44}H_{80}N_2O_{15}$
Megestrol Acetate	Progestogen	595-33-5	Y	$C_{24}H_{32}O_4$
Meglitinide	Antidiabetic	54870-28-9		$C_{17}H_{16}ClNO_4$
Meglucycline	Antibiotic	31770-79-3		$C_{29}H_{37}N_3O_{13}$
Meglumine	Antiprotozoal	6284-40-8	Y	$C_7H_{17}NO_5$
Meglumine Metrizoate	Antiprotozoal	1949-45-7		$C_{12}H_{11}I_3N_2O_4$
Meglutol	Antihyperlipidemic	503-49-1		$C_6H_{10}O_5$
Meladrazine	Anorexic	13957-36-3		$C_{11}H_{23}N_7$
Melagatran	Antithrombotic	159776-70-2		$C_{22}H_{31}N_5O_4$
Melarsomine	Antiprotozoal	128470-15-5		$C_{13}H_{21}AsN_8S_2$
Melarsonyl Potassium	Antiprotozoal	37526-80-0		$C_{13}H_{13}AsN_6O_4S_2$
Melarsoprol	Antiprotozoal	494-79-1		$C_{12}H_{15}AsN_6OS_2$
Meldonium	Antiarrhythmic	76144-81-5	Y	$C_6H_{14}N_2O_2$
Melengestrol Acetate	Antineoplastic	2919-66-6		$C_{25}H_{32}O_4$

Table S1. *Cont.*

Common Name	Indication	CAS Number	Oral	Molecular Formula
Meletimide	Antihypertensive	14745-50-7		$C_{24}H_{28}N_2O_2$
Melevodopa	Antiparkinsonian	7101-51-1		$C_{10}H_{13}NO_4$
Melinamide	Antihyperlipidemic	14417-88-0		$C_{26}H_{41}NO$
Melitracen	Antidepressant	5118-29-6		$C_{21}H_{25}N$
Melizame	Unclassified	26921-72-2		$C_7H_6N_4O_2$
Meloxicam	Antiinflammatory	71125-38-7	Y	$C_{14}H_{13}N_3O_4S_2$
Melperone	Antipsychotic	3575-80-2		$C_{16}H_{22}FNO$
Melphalan	Antineoplastic	148-82-3	Y	$C_{13}H_{18}Cl_2N_2O_2$
Melquinast	Bronchodilator	87611-28-7		$C_{15}H_{16}N_4O_3$
Meluadrine	Antihistamine	134865-33-1		$C_{12}H_{18}ClNO_2$
Memantine	Antiparkinsonian	19982-08-2	Y	$C_{12}H_{21}N$
Memotine	Antiviral	18429-69-1		$C_{17}H_{17}NO_2$
Menabitan	Analgesic	83784-21-8		$C_{37}H_{56}N_2O_3$
Menbutone	Choleretic	3562-99-0		$C_{15}H_{14}O_4$
Menfegol	Contraceptive	57821-32-6		$C_{18}H_{28}O_2$
Menglytate	Unclassified	579-94-2		$C_{14}H_{26}O_3$
Menitrazepam	Sedative	28781-64-8		$C_{16}H_{17}N_3O_3$
Menoctone	Antimalarial	14561-42-3		$C_{24}H_{32}O_3$
Menogaril	Antineoplastic	71628-96-1		$C_{28}H_{31}NO_{10}$
Menthyl Salicylate	Dermatologic	89-46-3		$C_{17}H_{24}O_3$
Meobentine Sulfate	Antiarrhythmic	46464-11-3		$C_{11}H_{17}N_3O$
Meparfynol	Sedative	77-75-8		$C_6H_{10}O$
Mepazine Acetate	Antipsychotic	60-89-9		$C_{19}H_{22}N_2S$
Mepenzolate	Antispasmodic	25990-43-6	Y	$C_{21}H_{26}NO_3+$
Meperidine	Analgesic	57-42-1	Y	$C_{15}H_{21}NO_2$
Mephenesin	Muscle Relaxant	59-47-2		$C_{10}H_{14}O_3$
Mephenesin Carbamate	Muscle Relaxant	533-06-2		$C_{11}H_{15}NO_4$
Mephenoxalone	Muscle Relaxant	70-07-5		$C_{11}H_{13}NO_4$
Mephentermine	Antihypotensive	100-92-5		$C_{11}H_{17}N$
Mephenytoin	Anticonvulsant	50-12-4	Y	$C_{12}H_{14}N_2O_2$
Mephobarbital	Anticonvulsant	115-38-8	Y	$C_{13}H_{14}N_2O_3$
Mepindolol	Antianginal	23694-81-7	Y	$C_{15}H_{22}N_2O_2$
Mepiperphenidol Bromide	Diuretic	520-20-7		$C_{19}H_{32}BrNO$
Mepiprazole	Sedative	20326-12-9		$C_{16}H_{21}ClN_4$
Mepiroxol	Antiinflammatory	6968-72-5		$C_6H_7NO_2$
Mepitiostane	Oxytocic	21362-69-6		$C_{25}H_{40}O_2S$
Mepivacaine	Anesthetic	96-88-8		$C_{15}H_{22}N_2O$
Mepixanox	Respiratory Stimulant	17854-59-0		$C_{20}H_{21}NO_3$
Mepramidil	Antihypotensive	23891-60-3		$C_{28}H_{33}NO_5$
Meprednisone	Glucocorticoid	1247-42-3		C22H28O5
Meprobamate	Sedative	57-53-4		$C_9H_{18}N_2O_4$
Meprochol	Steroid	590-31-8		$C_7H_{16}NO.Br$
Meproscillarin	Cardiotonic	33396-37-1		$C_{31}H_{44}O_8$
Meprotixol	Antitussive	4295-63-0		$C_{19}H_{23}NO_2S$
Meprylcaine	Anesthetic	495-70-5		$C_{14}H_{21}NO_2$
Meptazinol	Analgesic	54340-58-8	Y	$C_{15}H_{23}NO$
Mequidox	Antibacterial	16915-79-0		$C_{10}H_{10}N_2O_3$
Mequinol	Dermatologic	150-76-5		$C_7H_8O_2$
Mequitamium Iodide	Antihistaminic	101396-42-3		$C_{21}H_{25}N_2SI$
Mequitazine	Antihistaminic	29216-28-2	Y	$C_{20}H_{22}N_2S$
Meradimate	Dermatologic	134-09-8		$C_{17}H_{25}NO_2$
Merafloxacin	Antibiotic	91188-00-0		$C_{19}H_{23}F_2N_3O_3$
Meragidone Sodium	Antipsychotic	792128-03-1		$C_{10}H_{13}HgNO_5$
Merbromin	Antibacterial	55728-51-3		$C_{20}H_{10}Br_2HgO_6$
Mercaptopurine	Antineoplastic	50-44-2	Y	$C_5H_4N_4S$
Mercurobutol	Antifungal	498-73-7		C_{10}H13ClHgO
Mergocriptine	Antimigraine	81968-16-3		$C_{33}H_{43}$N5O5
Meribendan	Cardiotonic	119322-27-9		$C_{15}H_{14}N_6O$
Merimepodib	Antiviral	198821-22-6		C_{23}H24N4O6
Meropenem	Antibacterial	96036-03-2		$C_{17}H_{25}N_3O_5S$
Mesabolone	Steroid	7483-09-2		$C_{26}H_{40}O_3$
Mesalamine	Antiinflammatory	89-57-6	Y	$C_7H_7NO_3$
Meseclazone	Antiinflammatory	29053-27-8		$C_{11}H_{10}ClNO_3$

Table S1. *Cont.*

Common Name	Indication	CAS Number	Oral	Molecular Formula
Mesna	Mucolytic	3375-50-6		$C_2H_6O_3S_2$
Mesocarb	Nootropic	34262-84-5		$C_{18}H_{18}N_4O_2$
Mesoridazine	Antipsychotic	5588-33-0		$C_{21}H_{26}N_2OS_2$
Mespirenone	Pituitary	87952-98-5		$C_{25}H_{30}O_4S$
Mestanolone	Androgen	521-11-9		$C_{20}H_{32}O_2$
Mesterolone	Androgen	1424-00-6	Y	$C_{20}H_{32}O_2$
Mestilbol	Estrogen	7773-60-6		$C_{19}H_{22}O_2$
Mestranol	Estrogen	72-33-3		$C_{21}H_{26}O_2$
Mesudipine	Antianginal	62658-88-2		$C_{19}H_{24}N_2O_4S$
Mesulergine	Antidepressant	64795-35-3		$C_{18}H_{26}N_4O_2S$
Mesulfamide	Antibiotic	122-89-4		$C_7H_{10}N_2O_5S_2$
Mesulfen	Ectoparasiticide	135-58-0		$C_{14}H_{12}S_2$
Mesuprine	Vasodilator	7541-30-2		$C_{19}H_{26}N_2O_5S$
Metabutethamine	Anesthetic	4439-25-2		$C_{13}H_{20}N_2O_2$
Metabutoxycaine	Anesthetic	3624-87-1		$C_{17}H_{28}N_2O_3$
Metacetamol	Analgesic	621-42-1		$C_8H_9NO_2$
Metaclazepam	Anxiolytic	84031-17-4		$C_{18}H_{18}BrClN_2O$
Metaglycodol	Sedative	13980-94-4		$C_{11}H_{15}ClO_2$
Metahexamide	Antidiabetic	565-33-3		$C_{14}H_{21}N_3O_3S$
Metahomomenthol	Unclassified	17162-28-6		$C_{15}H_{28}O_3$
Metalol	Antihypertensive	7701-65-7		$C_{11}H_{18}N_2O_3S$
Metamelfalan	Antineoplastic	1088-80-8		$C_{13}H_{18}Cl_2N_2O_2$
Metamfazone	Antiinflammatory	54063-49-9		$C_{11}H_{11}N_3O$
Metamfepramone	Anorexic	15351-09-4		$C_{11}H_{15}NO$
Metampicillin	Antibiotic	6489-97-0		$C_{17}H_{19}N_3O_4S$
Metanixin	Antiinflammatory	4394-04-1		$C_{14}H_{14}N_2O_2$
Metapramine	Antidepressant	21730-16-5		$C_{16}H_{18}N_2$
Metaproterenol	Bronchodilator	586-06-1		$C_{11}H_{17}NO_3$
Metaterol	Bronchodilator	3571-71-9		$C_{11}H_{17}NO_2$
Metaxalone	Muscle Relaxant	1665-48-1	Y	$C_{12}H_{15}NO_3$
Metazamide	Antibacterial	14058-90-3		$C_{11}H_{12}N_2O_2$
Metazide	Antibacterial	1707-15-9		$C_{13}H_{14}N_6O_2$
Metazocine	Analgesic	3734-52-9		$C_{15}H_{21}NO$
Metbufen	Antiinflammatory	63472-04-8		$C_{17}H_{16}O_3$
Metcaraphen	Mydriatic	561-79-5		$C_{20}H_{31}NO_2$
Meteneprost	Oxytocic	61263-35-2		$C_{23}H_{38}O_4$
Metergoline	Pituitary	17692-51-2	Y	$C_{25}H_{29}N_3O_2$
Metergotamine	Analgesic	22336-84-1		$C_{34}H_{37}N_5O_5$
Metesculetol	Unclassified	52814-39-8		$C_{12}H_{10}O_6$
Metesind Glucuronate	Antineoplastic	138384-68-6		$C_{23}H_{24}N_4O_3S$
Metethoheptazine	Analgesic	509-84-2		$C_{17}H_{25}NO_2$
Metformin	Antidiabetic	657-24-9	Y	$C_4H_{11}N_5$
Methacholine	Cholinergic	55-92-5		$C_8H_{18}NO_2$
Methacycline	Antibiotic	914-00-1		$C_{22}H_{22}N_2O_8$
Methadone	Analgesic	76-99-3	Y	$C_{21}H_{27}NO$
Methadyl Acetate	Analgesic	509-74-0		$C_{23}H_{31}NO_2$
Methafurylene Fumarate	Antihistaminic	531-06-6		$C_{14}H_{19}N_3O$
Methallatal	Antiemetic	115-56-0		$C_{10}H_{14}N_2O_2S$
Methallenestril	Estrogen	517-18-0		$C_{18}H_{22}O_3$
Methallibure	Pituitary	926-93-2		$C_7H_{14}N_4S_2$
Methalthiazide	Diuretic	5611-64-3		$C_{12}H_{16}ClN_3O_4S_3$
Methamphetamine	Anorexic	537-46-2	Y	$C_{10}H_{15}N$
Methandriol	Steroid	521-10-8		$C_{20}H_{32}O_2$
Methandrostenolone	Androgen	72-63-9		$C_{20}H_{28}O_2$
Methaniazide	Antibacterial	13447-95-5		$C_7H_9N_3O_4S$
Methantheline	Antispasmodic	5818-17-7		C_{21}H26BrNO3
Methaphenilene	Antihistaminic	493-78-7		$C_{15}H_{20}N_2S$
Methapyrilene	Antihistaminic	91-80-5		$C_{14}H_{19}N_3S$
Methaqualone	Sedative	72-44-6		$C_{16}H_{14}N_2O$
Metharbital	Anticonvulsant	50-11-3		$C_9H_{14}N_2O_3$
Methastyridone	Antipsychotic	721-19-7		$C_{13}H_{15}NO_2$
Methazolamide	Diuretic	554-57-4		$C_5H_8N_4O_3S_2$
Methdilazine	Dermatologic	1982-37-2		$C_{18}H_{20}N_2S$

Table S1. *Cont.*

Common Name	Indication	CAS Number	Oral	Molecular Formula
Methenamine	Antibacterial	100-97-0		$C_6H_{12}N_4$
Methenolone Acetate	Steroid	434-05-9		$C_{22}H_{32}O_3$
Methenolone Enanthate	Steroid	303-42-4		$C_{27}H_{42}O_3$
Metheptazine	Analgesic	469-78-3		$C_{16}H_{23}NO_2$
Methestrol	Estrogen	130-73-4		$C_{20}H_{26}O_2$
Methetoin	Anticonvulsant	5696-06-0		$C_{12}H_{14}N_2O_2$
Methicillin	Antibiotic	61-32-5		$C_{17}H_{20}N_2O_6S$
Methimazole	Thyroid	60-56-0	Y	$C_4H_6N_2S$
Methiodal	Anesthetic	143-47-5		CH_3IO_3S
Methiomeprazine	Analgesic	7009-43-0		$C_{19}H_{24}N_2S_2$
Methionine	Hepatoprotectant	63-68-3		$C_5H_{11}NO_2S$
Methisazone	Antiviral	1910-68-5		$C_{10}H_{10}N_4OS$
Methitural	Sedative	467-43-6		$C_{12}H_{20}N_2O_2S_2$
Methixene	Muscle Relaxant	4969-02-2	Y	$C_{20}H_{23}NS$
Methocarbamol	Muscle Relaxant	532-03-6	Y	$C_{11}H_{15}NO_5$
Methohexital	Anesthetic	151-83-7		$C_{14}H_{18}N_2O_3$
Methopholine	Analgesic	2154-02-1		$C_{20}H_{24}ClNO_2$
Methoprene	Dermatologic	40596-69-8		$C_{19}H_{34}O_3$
Methoserpidine	Antihypertensive	865-04-3		$C_{33}H_{40}N_2O_9$
Methotrexate	Antineoplastic	59-05-2	Y	$C_{20}H_{22}N_8O_5$
Methotrimeprazine	Analgesic	60-99-1		$C_{19}H_{24}N_2OS$
Methoxamine	Antihypotensive	390-28-3		$C_{11}H_{17}NO_3$
Methoxsalen	Dermatologic	298-81-7	Y	$C_{12}H_8O_4$
Methoxyflurane	Anesthetic	76-38-0		$C_3H_4Cl_2F_2O$
2--5-Nitrofuran	Antifungal	586-84-5		$C_6H_7NO_4$
Methoxyphedrine	Bronchodilator	530-54-1		$C_{11}H_{15}NO_2$
Methoxyphenamine	Bronchodilator	93-30-1		$C_{11}H17NO$
Methoxypromazine Maleate	Antipsychotic	61-01-8		$C_{18}H_{22}N_2OS$
Methscopolamine	Antispasmodic	13265-10-6	Y	$C_{18}H_{24}NO_4$
Methsuximide	Anticonvulsant	77-41-8	Y	$C_{12}H_{13}NO_2$
Methyclothiazide	Diuretic	135-07-9	Y	$C_9H_{11}Cl_2N_3O_4S_2$
Methyl Aminolevulinate	Antineoplastic	33320-16-0		$C_6H_{11}NO_3$
Methyl Nicotinate	Dermatologic	93-60-7		$C_7H_7NO_2$
Methyl Palmoxirate	Antidiabetic	69207-52-9		$C_{18}H_{34}O_3$
Methyl Salicylate	Dermatologic	119-36-8		$C_8H_8O_3$
Methylatropine Nitrate	Mydriatic	52-88-0		$C_{18}H_{26}N_2O_6$
Methylbenactyzium Bromide	Antidepressant	3166-62-9		$C_{21}H_{28}BrNO_3$
Methylchromone	Antispasmodic	85-90-5		$C_{10}H_8O_2$
Methyldesorphine	Analgesic	16008-36-9		$C_{18}H_{21}NO_2$
Methyldihydromorphine	Analgesic	509-56-8		$C_{18}H_{23}NO_3$
Methyldopa	Antihypertensive	555-30-6		$C_{10}H_{13}NO_4$
Methylene Blue	Hemantic	61-73-4		$C_{16}H_{18}ClN_3S$
Methylephedrine	Nootropic	552-79-4		$C_{11}H17NO$
Methylergonovine	Oxytocic	113-42-8	Y	$C_{20}H_{25}N_3O_2$
Methylhexaneamine	Antihypotensive	105-41-9		$C_7H_{17}N$
Methylmethionine Sulfonium Chloride	Unclassified	1115-84-0		$C_6H_{14}ClNO_2S$
Methylphenidate	Nootropic	113-45-1	Y	$C_{14}H_{19}NO_2$
Methylprednisolone	Glucocorticoid	83-43-2	Y	$C_{22}H_{30}O_5$
Methylprednisolone Aceponate	Glucocorticoid	86401-95-8		$C_{27}H_{36}O_7$
Methylprednisolone Acetate	Glucocorticoid	53-36-1	Y	$C_{24}H_{32}O_6$
Methylprednisolone Hemisuccinate	Glucocorticoid	2921-57-5		$C_{26}H_{34}O_8$
Methylprednisolone Sodium Phosphate	Glucocorticoid	22252-38-6		$C_{22}H_{31}O_8P$
Methylprednisolone Suleptanate	Antiinflammatory	121807-10-1		$C_{33}H_{49}NO_{10}S$
Methylpromazine	Dermatologic	84-96-8		$C_{18}H_{22}N_2S$
Methyltestosterone	Androgen	58-18-4	Y	$C_{20}H_{30}O_2$
Methylthiouracil	Thyroid	56-04-2		$C_5H_6N_2OS$
Methynodiol Diacetate	Progestogen	23163-51-1		$C_{25}H_{34}O_4$
Methyprylon	Sedative	125-64-4		$C_{10}H_{17}NO_2$
Methysergide	Antimigraine	361-37-5	Y	$C_{21}H_{27}N_3O_2$
Metiamide	Antiulcerative	34839-70-8		$C_9H_{16}N_4S_2$
Metiapine	Antipsychotic	5800-19-1		$C_{19}H_{21}N_3S$

Table S1. *Cont.*

Common Name	Indication	CAS Number	Oral	Molecular Formula
Metiazinic Acid	Antiinflammatory	13993-65-2		$C_{15}H_{13}NO_2S$
Metibride	Antithrombotic	77989-60-7		$C_{18}H_{18}ClN_3O_2S_2$
Meticrane	Diuretic	1084-65-7		$C_{10}H_{13}NO_4S_2$
Metildigoxin	Cardiotonic	30685-43-9	Y	$C_{42}H_{66}O_{14}$
Metindizate	Antispasmodic	15687-33-9		$C_{25}H_{31}NO_3$
Metioprim	Antibacterial	68902-57-8		$C_{14}H_{18}N_4O_2S$
Metioxate	Antidepressant	42110-58-7		$C_{22}H_{27}N_3O_4S$
Metipirox	Antibacterial	29342-02-7		$C_7H_9NO_2$
Metipranolol	Antihypertensive	22664-55-7		$C_{17}H_{27}NO_4$
Metiprenaline	Bronchodilator	1212-03-9		$C_{12}H_{19}NO_3$
Metitepine	Antipsychotic	20229-30-5		$C_{20}H_{24}N_2S_2$
Metizoline	Adrenergic	17692-22-7		$C_{13}H_{14}N_2S$
Metkephamid Acetate	Analgesic	66960-34-7		$C_{29}H_{40}N_6O_6S$
Metochalcone	Choleretic	18493-30-6		$C_{18}H_{18}O_4$
Metoclopramide	Antiemetic	364-62-5	Y	$C_{14}H_{22}ClN_3O_2$
Metocurine	Muscle Relaxant	5152-30-7		$C_{40}H_{48}N_2O_6$
Metocurine Iodide	Muscle Relaxant	7601-55-0		$C_{40}H_{48}I_2N_2O_6$
Metofenazate	Antipsychotic	388-51-2		$C_{31}H_{36}ClN_3O_5S$
Metogest	Steroid	52279-58-0		$C_{20}H_{30}O_2$
Metolazone	Diuretic	17560-51-9	Y	$C_{16}H_{16}ClN_3O_3S$
Metomidate	Sedative	5377-20-8		$C_{13}H_{14}N_2O_2$
Metopimazine	Antiemetic	14008-44-7		$C_{22}H_{27}N_3O_3S_2$
Metopon	Analgesic	143-52-2		$C_{18}H_{21}NO_3$
Metoprine	Antineoplastic	7761-45-7		$C_{11}H_{10}Cl_2N_4$
Metoprolol	Antihypertensive	51384-51-1	Y	$C_{15}H_{25}NO_3$
Metoquizine	Mydriatic	7125-67-9		$C_{22}H_{27}N_5O$
Metoserpate	Sedative	1178-28-5		$C_{24}H_{32}N_2O_5$
Metostilenol	Steroid	103980-45-6		$C_{15}H_{21}NO_3$
Metoxepin	Antipsychotic	22013-23-6		$C_{20}H_{22}N_2O_2$
Metrafazoline	Decongestant	38349-38-1		$C_{17}H_{22}N_2$
Metralindole	Antidepressant	54188-38-4		$C_{15}H_{17}N_3O$
Metrazifone	Nootropic	68289-14-5		$C_{20}H_{23}N_5O$
Metrenperone	Steroid	81043-56-3		$C_{24}H_{26}FN_3O_2$
Metribolone	Steroid	965-93-5		$C_{19}H_{24}O_2$
Metrifonate	Anthelminithic	52-68-6		$C_4H_8Cl_3O_4P$
Metrifudil	Antiseizure	23707-33-7		$C_{18}H_{21}N_5O_4$
Metronidazole	Antiprotozoal	443-48-1	Y	$C_6H_9N_3O_3$
Meturedepa	Antineoplastic	661-29-6		$C_{11}H_{22}N_3O_3P$
Metyridine	Analgesic	14-91-0		$C_8H_{11}NO$
Metyrosine	Antineoplastic	672-87-7	Y	$C_{10}H_{13}NO_3$
Mevastatin	Antihyperlipidemic	73573-88-3		$C_{23}H_{34}O_5$
Mexafylline	Bronchodilator	80294-25-3		$C_{14}H_{18}N_4O_2$
Mexazolam	Anxiolytic	31868-18-5		$C_{18}H_{16}Cl_2N_2O_2$
Mexenone	Dermatologic	1641-17-4		$C_{15}H_{14}O_3$
Mexiletine	Antiarrhythmic	31828-71-4	Y	$C_{11}H17NO$
Mexiprostil	Antiulcerative	88980-20-5		$C_{23}H_{40}O_6$
Mexoprofen	Analgesic	37529-08-1		$C_{16}H_{22}O_2$
Mexrenoate Potassium	Antihypertensive	41020-68-2		$C_{24}H_{34}O_6$
Mezacopride	Antiemetic	89613-77-4		$C_{16}H_{22}ClN_3O_2$
Mezepine	Analgesic	27432-00-4		$C_{18}H_{22}N_2$
Mezilamine	Antidepressant	50335-55-2		$C_{11}H_{18}ClN_5S$
Mezlocillin	Antibiotic	51481-65-3		$C_{21}H_{25}N_5O_8S_2$
Mianserin	Antidepressant	24219-97-4	Y	$C_{18}H_{20}N_2$
Mibefradil	Antihypertensive	116644-53-2	Y	$C_{29}H_{38}FN_3O_3$
Mibolerone	Androgen	3704-09-4		$C_{20}H_{30}O_2$
Micinicate	Vasodilator	39537-99-0		$C_{23}H_{27}NO_4$
Miconazole	Antifungal	22916-47-8		$C_{18}H_{14}Cl_4N_2O$
Micronomicin	Antibiotic	52093-21-7		$C_{20}H_{41}N_5O_7$
Midaflur	Sedative	23757-42-8		$C_7H_3F_{12}N_3$
Midafotel	Analgesic	117414-74-1		$C_8H_{15}N_2O_5P$
Midaglizole	Antihypertensive	66529-17-7		$C_{16}H_{17}N_3$
Midamaline	Antihypertensive	496-38-8		$C_{18}H_{21}ClN_4$
Midaxifylline	Bronchodilator	151159-23-8		$C_{16}H_{25}N_5O_2$

Table S1. *Cont.*

Common Name	Indication	CAS Number	Oral	Molecular Formula
Midazogrel	Unclassified	80614-27-3		$C_{18}H_{24}N_2O$
Midazolam	Anesthetic	59467-70-8		$C_{18}H_{13}ClFN_3$
Midecamycin	Antibiotic	35457-80-8		$C_{41}H_{67}NO_{15}$
Midesteine	Antitussive	94149-41-4		$C_{12}H_{13}NO_3S_3$
Midodrine	Antihypotensive	42794-76-3	Y	$C_{12}H_{18}N_2O_4$
Midostaurin	Antineoplastic	120685-11-2	Y	$C_{35}H_{30}N_4O_4$
Mifentidine	Antihistaminic	83184-43-4		$C_{13}H_{16}N_4$
Mifepristone	Abortifacient	84371-65-3		$C_{29}H_{35}NO_2$
Mifobate	Antihyperlipidemic	76541-72-5		$C_{11}H_{17}ClO_7P_2$
Migalastat	Antihypertensive	108147-54-2	Y	$C_6H_{13}NO_4$
Miglitol	Antidiabetic	72432-03-2	Y	C8H17NO5
Miglustat	Antihypertensive	72599-27-0	Y	$C_{10}H_{21}NO_4$
Milacainide	Antiarrhythmic	141725-09-9		$C_{19}H_{25}N_3O$
Milacemide	Anticonvulsant	76990-56-2		$C_7H_{16}N_2O$
Milameline	Nootropic	139886-32-1		$C_8H_{14}N_2O$
Milataxel	Antineoplastic	393101-41-2		$C_{44}H_{55}NO_{16}$
Milenperone	Antipsychotic	59831-64-0		$C_{22}H_{23}ClFN_3O_2$
Milfasartan	Antihypertensive	148564-47-0		$C_{30}H_{30}N_6O_3S$
Milipertine	Antipsychotic	24360-55-2		$C_{24}H_{31}N_3O_3$
Milnacipran	Antidepressant	92623-85-3	Y	$C_{15}H_{22}N_2O$
Miloxacin	Antibiotic	37065-29-5		$C_{12}H_9NO_6$
Milrinone	Cardiotonic	78415-72-2		$C_{12}H_9N_3O$
Miltefosine	Antineoplastic	58066-85-6	Y	$C_{21}H_{46}NO_4P$
Milverine	Antispasmodic	75437-14-8		$C_{20}H_{20}N_2$
Mimbane	Analgesic	3277-59-6		$C_{20}H_{26}N_2$
Minalrestat	Antidiabetic	129688-50-2		$C_{19}H_{11}BrF_2N_2O_4$
Minamestane	Antineoplastic	105051-87-4		$C_{19}H_{23}NO_2$
Minaprine	Antidepressant	25905-77-5		$C_{17}H_{22}N_4O$
Minaxolone	Anesthetic	62571-87-3		$C_{25}H_{43}NO_3$
Mindodilol	Unclassified	70260-53-6		$C_{23}H_{28}N_2O_3$
Mindoperone	Antipsychotic	52157-83-2		$C_{25}H_{29}FN_2O_2$
Minepentate	Unclassified	13877-99-1		$C_{18}H_{27}NO_3$
Minocromil	Antihistaminic	85118-44-1		$C_{18}H_{16}N_2O_6$
Minocycline	Antibiotic	10118-90-8	Y	$C_{23}H_{27}N_3O_7$
Minodronic Acid	Bone Resorption Inhibitor	180064-38-4	Y	$C_9H_{12}N_2O_7P_2$
Minopafant	Antithrombotic	128420-61-1		$C_{46}H_{73}ClN_4O_9$
Minoxidil	Antihypertensive	38304-91-5		$C_9H_{15}N_5O$
Mioflazine	Vasodilator	79467-23-5		$C_{29}H_{30}Cl_2F_2N_4O_2$
Mipimazole	Antibiotic	20406-60-4		$C_6H_{12}N_2S$
Mipitroban	Anticoagulant	136122-46-8		$C_{19}H_{19}Cl_2N_3O_2$
Miproxifene	Antineoplastic	129612-87-9		$C_{29}H_{35}NO_2$
Mirfentanil	Analgesic	117523-47-4		$C_{22}H_{24}N_4O_2$
Mirincamycin	Antibacterial	31101-25-4		$C_{19}H_{35}ClN_2O_5S$
Mirisetron Maleate	Anxiolytic	135905-89-4		$C_{24}H_{31}N_3O_2$
Miroprofen	Analgesic	55843-86-2		$C_{16}H_{14}N_2O_2$
Mirosamicin	Antibacterial	3684-69-2		$C_{37}H_{61}NO_{13}$
Mirtazapine	Antidepressant	85650-52-8	Y	$C_{17}H_{19}N_3$
Misonidazole	Antiprotozoal	13551-87-6		$C_7H_{11}N_3O_4$
Misoprostol	Antiulcerative	59122-46-2		$C_{22}H_{38}O_5$
Mitemcinal Fumarate	Gastroprokinetic	154738-42-8		$C_{40}H_{69}NO_{12}$
Mitiglinide	Antidiabetic	145375-43-5	Y	$C_{19}H_{25}NO_3$
Mitindomide	Antineoplastic	10403-51-7		$C_{14}H_{12}N_2O_4$
Mitobronitol	Antineoplastic	488-41-5		$C_6H_{12}Br_2O_4$
Mitoclomine	Antispasmodic	17692-54-5		$C_{16}H_{19}Cl_2NO$
Mitoflaxone	Antineoplastic	87626-55-9		$C_{17}H_{12}O_4$
Mitoguazone	Antineoplastic	459-86-9		$C_5H_{12}N_8$
Mitolactol	Antineoplastic	10318-26-0		$C_6H_{12}Br_2O_4$
Mitomycin	Antineoplastic	50-07-7		$C_{15}H_{18}N_4O_5$
Mitonafide	Antineoplastic	54824-17-8		$C_{16}H_{15}N_3O_4$
Mitopodozide	Antineoplastic	1508-45-8		$C_{24}H_{30}N_2O_8$
Mitoquidone	Antineoplastic	91753-07-0		$C_{20}H_{13}NO_2$
Mitotane	Antineoplastic	53-19-0	Y	$C_{14}H_{10}Cl_4$
Mitotenamine	Antihypertensive	7696-00-6		$C_{13}H_{15}BrClNS$

Table S1. *Cont.*

Common Name	Indication	CAS Number	Oral	Molecular Formula
Mitoxantrone	Antineoplastic	65271-80-9		$C_{22}H_{28}N_4O_6$
Mitozolomide	Antineoplastic	85622-95-3		$C_7H_7ClN_6O_2$
Mitratapide	Antiobesity	179602-65-4		$C_{36}H_{41}ClN_8O_4S$
Mivazerol	Antihypotensive	125472-02-8		$C_{11}H_{11}N_3O_2$
Mivobulin Isethionate	Antineoplastic	122332-18-7		$C_{17}H_{19}N_5O_2$
Mivotilate	Hepatic Protectant	130112-42-4		$C_{12}H_{14}N_2O_3S_3$
Mixidine	Vasodilator	27737-38-8		$C_{15}H_{22}N_2O_2$
Mizolastine	Antihistaminic	108612-45-9	Y	$C_{24}H_{25}FN_6O$
Mizoribine	Immunosuppressant	50924-49-7	Y	$C_9H_{13}N_3O_6$
Mobenzoxamine	Antiemetic	65329-79-5		$C_{30}H_{35}FN_2O_3$
Mocimycin	Antibiotic	50935-71-2		$C_{43}H_{60}N_2O_{12}$
Mociprazine	Antiemetic	56693-13-1		$C_{22}H_{32}N_2O_3$
Moclobemide	Antidepressant	71320-77-9	Y	$C_{13}H_{17}ClN_2O_2$
Moctamide	Antithrombotic	29619-86-1		$C_{33}H_{47}NO$
Modafinil	Nootropic	68693-11-8	Y	$C_{15}H_{15}NO_2S$
Modaline Sulfate	Antidepressant	2856-74-8		$C_{10}H_{15}N_3$
Modecainide	Antiarrhythmic	81329-71-7		$C_{22}H_{28}N_2O_3$
Modipafant	Antithrombotic	122957-06-6		$C_{34}H_{29}ClN_6O_3$
Moexipril	Antihypertensive	103775-10-6	Y	$C_{27}H_{34}N_2O_7$
Moexiprilat	Antihypertensive	103775-14-0		$C_{25}H_{30}N_2O_7$
Mofarotene	Antineoplastic	125533-88-2		$C_{29}H_{39}NO_2$
Mofebutazone	Antiinflammatory	2210-63-1		$C_{13}H_{16}N_2O_2$
Mofegiline	Antiparkinsonian	119386-96-8		$C_{11}H_{13}F_2N$
Mofezolac	Antiinflammatory	78967-07-4	Y	$C_{19}H_{17}NO_5$
Mofloverine	Antispasmodic	54063-50-2		$C_{16}H_{23}NO_6$
Mofoxime	Analgesic	29936-79-6		$C_{14}H_{18}N_2O_4$
Moguisteine	Antitussive	19637-67-1		$C_{16}H_{21}NO_5S$
Molfarnate	Antiulcerative	83689-23-0		$C_{31}H_{50}O_2$
Molinazone	Analgesic	5581-46-4		$C_{11}H_{12}N_4O_2$
Molindone	Antipsychotic	7416-34-4	Y	$C_{16}H_{24}N_2O_2$
Molracetam	Nootropic	94746-78-8		$C_{18}H_{25}N_3O_4$
Molsidomine	Antianginal	25717-80-0	Y	$C_9H_{14}N_4O_4$
Mometasone Furoate	Steroid	83919-23-7		$C_{27}H_{30}Cl_2O_6$
Monalazone Disodium	Antiinflammatory	106145-03-3		$C_7H_6ClNO_4S$
Monatepil Maleate	Antianginal	103377-41-9		$C_{28}H_{30}FN_3OS$
Monensin	Antibiotic	17090-79-8		$C_{36}H_{62}O_{11}$
Monobenzone	Dermatologic	103-16-2		$C_{13}H_{12}O_2$
Monoctanoin	Anticholelithogenic	502-54-5		$C_{11}H_{22}O_4$
Monometacrine	Antidepressant	4757-49-7		$C_{19}H_{24}N_2$
Monoxerutin	Capillary Protectant	23869-24-1		$C_{29}H_{34}O_{17}$
Montelukast	Bronchodilator	158966-92-8	Y	$C_{35}H_{36}ClNO_3S$
Montirelin	Thyroid	90243-66-6		$C_{17}H_{24}N_6O_4S$
Moperone	Antipsychotic	1050-79-9		$C_{22}H_{26}FNO_2$
Mopidamol	Antineoplastic	13665-88-8		$C_{19}H_{31}N_7O_4$
Mopidralazine	Antihypertensive	75841-82-6		$C_{14}H_{19}N_5O$
Moprolol	Antihypertensive	5741-22-0		$C_{13}H_{21}NO_3$
Moquizone	Choleretic	19395-58-5		$C_{20}H_{21}N_3O_3$
Morantel Tartrate	Anthelminithic	20574-50-9		$C_{12}H_{16}N_2S$
Morazone	Analgesic	6536-18-1		$C_{23}H_{27}N_3O_2$
Morclofone	Antitussive	31848-01-8		$C_{21}H_{24}ClNO_5$
Morforex	Anorexic	41152-17-4		$C_{15}H_{24}N_2O$
Moricizine	Antiarrhythmic	31883-05-3	Y	$C_{22}H_{25}N_3O_4S$
Morinamide	Antibacterial	952-54-5		$C_{10}H_{14}N_4O_2$
Morniflumate	Antiinflammatory	65847-85-0		$C_{19}H_{20}F_3N_3O_3$
Morocromen	Antithrombotic	35843-07-3		$C_{21}H_{27}N_3O_5$
Moroxydine	Antiviral	3731-59-7		$C_6H_{13}N_5O$
Morpheridine	Analgesic	469-81-8		$C_{20}H_{30}N_2O_3$
Morphine Glucuronide	Nootropic	20290-10-2		$C_{23}H_{27}NO_9$
Morphine	Analgesic	57-27-2	Y	$C_{17}H_{19}NO_3$
Morsuximide	Anticonvulsant	3780-72-1		$C_{16}H_{20}N_2O_3$
Mosapramine	Antipsychotic	89419-40-9		$C_{28}H_{35}ClN_4O$
Mosapride	Gastroprokinetic	112885-41-3	Y	$C_{21}H_{25}ClFN_3O_3$
Motapizone	Bronchodilator	90697-57-7		$C_{12}H_{12}N_4OS$

Table S1. *Cont.*

Common Name	Indication	CAS Number	Oral	Molecular Formula
Motexafin	Antineoplastic	189752-49-6		$C_{48}H_{67}N_5O_{10}$
Motrazepam	Anxiolytic	29442-58-8		$C_{17}H_{15}N_3O_4$
Motretinide	Dermatologic	56281-36-8		$C_{23}H_{31}NO_2$
Moveltipril	Antihypertensive	85856-54-8		$C_{19}H_{30}N_2O_5S$
Moxadolen	Unclassified	75992-53-9		$C_{11}H_{13}NO_4$
Moxalactam	Antibiotic	64952-97-2		$C_{20}H_{20}N_6O_9S$
Moxaprindine	Antiarrhythmic	53076-26-9		$C_{23}H_{32}N_2O$
Moxastine	Antihistaminic	3572-74-5		$C_{18}H_{23}NO$
Moxaverine	Antispasmodic	10539-19-2		$C_{20}H_{21}NO_2$
Moxazocine	Analgesic	58239-89-7		$C_{18}H_{25}NO_2$
Moxestrol	Estrogen	34816-55-2		$C_{21}H_{26}O_3$
Moxicoumone	Unclassified	17692-56-7		$C_{22}H_{30}N_2O_6$
Moxidectin	Antiprotozoal	113507-06-5		C_{37}H53NO8
Moxifloxacin	Antibiotic	151096-09-2	Y	$C_{21}H_{24}FN_3O_4$
Moxilubant Maleate	Antirheumatic	146978-48-5		$C_{26}H_{37}N_3O_4$
Moxipraquine	Antiprotozoal	23790-08-1		$C_{24}H_{38}N_4O_2$
Moxiraprine	Muscle Relaxant	82239-52-9		$C_{17}H_{22}N_4O_2$
Moxisylyte	Vasodilator	54-32-0	Y	$C_{16}H_{25}NO_3$
Moxnidazole	Antiprotozoal	52279-59-1		$C_{13}H_{18}N_6O_5$
Moxonidine	Antihypertensive	75438-57-2		$C_9H_{12}ClN_5O$
Mozavaptan	Plasma Volume Expander	137975-06-5	Y	$C_{27}H_{29}N_3O_2$
Mozenavir	Antiviral	174391-92-5		$C_{33}H_{36}N_4O_3$
Mubritinib	Antineoplastic	366017-09-6		$C_{25}H_{23}F_3N_4O_2$
Mupirocin	Antibacterial	12650-69-0		$C_{26}H_{44}O_9$
Murabutide	Immunomodulator	74817-61-1		$C_{23}H_{40}N_4O_{11}$
Muraglitazar	Antidiabetic	331741-94-7		$C_{29}H_{28}N_2O_7$
Murocainide	Antiarrhythmic	66203-94-9		$C_{19}H_{27}N_3O_5$
Muzolimine	Diuretic	55294-15-0		$C_{11}H_{11}Cl_2N_3O$
Mycophenolate Mofetil	Immunomodulator	128794-94-5	Y	$C_{23}H_{31}NO_7$
Mycophenolic Acid	Immunosuppressant	24280-93-1		$C_{17}H_{20}O_6$
Myfadol	Analgesic	4575-34-2		$C_{21}H_{25}NO_2$
Myralact	Unclassified	25737-87-5		$C_{16}H_{35}NO$
Myrophine	Analgesic	467-18-5		$C_{38}H_{51}NO_4$
Myrtecaine	Anesthetic	7712-50-7		$C_{17}H_{31}NO$
N Pyridinium Chloride	Unclassified	6272-74-8		$C_{21}H_{35}ClN_2O_3$
N,1-Dimethylhexylamine	Decongestant	540-43-2		$C_8H_{19}N$
N,N Dimethyl P Toluidine	Unclassified	99-97-8		$C_9H_{13}N$
Nabazenil	Anticonvulsant	58019-65-1		$C_{35}H_{55}NO_3$
Nabilone	Antiemetic	51022-71-0	Y	$C_{24}H_{36}O_3$
Nabitan	Analgesic	66556-74-9		$C_{35}H_{52}N_2O_3$
Naboctate	Antiglaucoma	74912-19-9		$C_{33}H_{53}NO_3$
Nabumetone	Antiinflammatory	42924-53-8	Y	$C_{15}H_{16}O_2$
Nacartocin	Oxytocic	77727-10-7		$C_{46}H_{71}N_{11}O_{11}S$
Nadide	Antidote	53-84-9		$C_{21}H_{27}N_7O_{14}P_2$
Nadifloxacin	Antibiotic	124858-35-1		$C_{19}H_{21}FN_2O_4$
Nadolol	Antihypertensive	42200-33-9	Y	$C_{17}H_{27}NO_4$
Nadoxolol	Antiarrhythmic	54063-51-3		$C_{14}H_{16}N_2O_3$
Naepaine	Anesthetic	2188-67-2		$C_{14}H_{22}N_2O_2$
Nafagrel	Antiinflammatory	97901-21-8		$C_{15}H_{16}N_2O_2$
Nafamostat Mesylate	Anticoagulant	81525-10-2		$C_{19}H_{17}N_5O_2$
Nafazatrom	Antithrombotic	59040-30-1		$C_{16}H_{16}N_2O_2$
Nafcaproic Acid	Hemostatic	1085-91-2		$C_{16}H_{18}O_2$
Nafcillin	Antibiotic	147-52-4		$C_{21}H_{22}N_2O_5S$
Nafenodone	Antidepressant	92615-20-8		$C_{20}H_{23}NO$
Nafenopin	Antihyperlipidemic	3771-19-5		$C_{20}H_{22}O_3$
Nafetolol	Antihypertensive	42050-23-7		$C_{19}H_{29}NO_3$
Nafimidone	Anticonvulsant	64212-22-2		$C_{15}H_{12}N_2O$
Nafiverine	Antispasmodic	5061-22-3		$C_{34}H_{38}N_2O_4$
Naflocort	Steroid	59497-39-1		$C_{29}H_{33}FO_4$
Nafomine Malate	Muscle Relaxant	46263-35-8		$C_{12}H_{13}NO$
Nafoxadol	Analgesic	84145-90-4		$C_{15}H_{15}NO_2$
Nafoxidine	Steroid	1845-11-0		$C_{29}H_{31}NO_2$
Nafronyl	Vasodilator	31329-57-4	Y	$C_{24}H_{33}NO_3$

Table S1. *Cont.*

Common Name	Indication	CAS Number	Oral	Molecular Formula
Naftalofos	Anthelminithic	1491-41-4		$C_{16}H_{16}NO_6P$
Naftazone	Capillary Protectant	15687-37-3	Y	$C_{11}H_9N_3O_2$
Naftifine	Antifungal	65472-88-0		$C_{21}H_{21}N$
Naftopidil	Antihypertensive	57149-07-2	Y	$C_{24}H_{28}N_2O_3$
Naftoxate	Unclassified	28820-28-2		$C_{19}H_{14}N_2OS_2$
Naftypramide	Antiemetic	1505-95-9		$C_{19}H_{26}N_2O$
Naglivan	Antidiabetic	122575-28-4		$C_{22}H_{46}N_4O_3S_2V$
Nalbuphine	Analgesic	20594-83-6	Y	$C_{21}H_{27}NO_4$
Nalfurafine	Dermatologic	152657-84-6		C28H32N2O5
Nalidixic Acid	Antibiotic	389-08-2		$C_{12}H_{12}N_2O_3$
Nalmefene	Antidote	55096-26-9		$C_{21}H_{25}NO_3$
Nalmexone	Analgesic	16676-26-9	Y	$C_{21}H_{25}NO_4$
Nalorphine	Antidote	62-67-9		$C_{19}H_{21}NO_3$
Naloxone	Antidote	465-65-6	Y	$C_{19}H_{21}NO_4$
Naltrexone	Antidote	16590-41-3		$C_{20}H_{23}NO_4$
Naminidil	Vasodilator	220641-11-2	Y	$C_{15}H_{19}N_5$
Naminterol	Bronchodilator	93047-40-6		$C_{19}H_{26}N_2O_3$
Namirotene	Immunomodulator	101506-83-6		$C_{17}H_{18}O_2S$
Nanafrocin	Antibiotic	52934-83-5		$C_{16}H_{14}O_6$
Nandrolone Cyclotate	Steroid	22263-51-0		$C_{28}H_{38}O_3$
Nandrolone Decanoate	Steroid	360-70-3		$C_{28}H_{44}O_3$
Nandrolone Phenpropionate	Androgen	62-90-8		$C_{27}H_{34}O_3$
Nanofin	Antirheumatic	504-03-0		$C_7H_{15}N$
Nanterinone	Vasodilator	102791-47-9		$C_{15}H_{15}N_3O$
Nantradol	Analgesic	72028-54-7		$C_{27}H_{35}NO_4$
Napactadine	Antidepressant	76631-45-3		$C_{14}H_{16}N_2$
Napamezole	Antidepressant	91524-14-0		$C_{14}H_{16}N_2$
Naphazoline	Decongestant	835-31-4		$C_{14}H_{14}N_2$
Naphthalene	Anthelminithic	91-20-3		$C_{10}H_8$
Naphthocaine	Analgesic	5656-83-7		$C_{17}H_{22}N_2O_2$
Naphthonone	Unclassified	7114-11-6		$C_{16}H_{16}O2$
Napirimus	Immunosuppressant	70696-66-1		$C_{17}H_{13}NO3$
Napitane Mesylate	Antidepressant	148152-63-0		$C_{22}H_{25}NO_2$
Naprodoxime	Analgesic	57925-64-1		$C_{13}H_{14}N_2O_2$
Naproxen	Antiinflammatory	22204-53-1	Y	$C_{14}H_{14}O_3$
Naproxol	Antiinflammatory	26159-36-4		$C_{14}H_{16}O_2$
Napsagatran	Antithrombotic	154397-77-0		$C_{26}H_{34}N_6O_6S$
Naranol	Antipsychotic	22292-91-7		$C_{18}H_{21}NO_2$
Narasin	Antibacterial	55134-13-9		$C_{43}H_{72}O_{11}$
Naratriptan	Antimigraine	121679-13-8	Y	$C_{17}H_{25}N_3O_2S$
Narceine	Analgesic	131-28-2		$C_{23}H_{27}NO_8$
Nardeterol	Bronchodilator	73865-18-6		$C_{20}H_{24}FN_3O_2$
Naringin	Antiinflammatory	10236-47-2		$C_{27}H_{32}O_{14}$
Naroparcil	Antithrombotic	120819-70-7		$C_{19}H_{17}NO_4S_2$
Natamycin	Antifungal	7681-93-8		$C_{33}H_{47}NO_{13}$
Nateglinide	Antidiabetic	105816-04-4	Y	$C_{19}H_{27}NO_3$
Naveglitazar	Antidiabetic	476436-68-7		$C_{25}H_{26}O_6$
Navuridine	Antiviral	84472-85-5		$C_9H_{11}N_5O_4$
Naxagolide	Antiparkinsonian	88058-88-2		$C_{15}H_{21}NO_2$
Naxaprostene	Prostaglandin	87269-59-8		$C_{25}H_{32}O_4$
Naxifylline	Cardiotonic	166374-49-8		$C_{18}H_{24}N_4O_3$
Nealbarbital	Sedative	561-83-1		$C_{12}H_{18}N_2O_3$
Nebentan	Unclassified	403604-85-3		$C_{24}H_{21}N_5O_5S$
Nebicapone	Antiparkinsonian	274925-86-9		$C_{14}H_{11}NO_5$
Nebidrazine	Anorexic	55248-23-2		$C_9H_8Cl_2N_6$
Nebivolol	Antihypertensive	118457-14-0	Y	$C_{22}H_{25}F_2NO_4$
Neboglamine	Antifungal	163000-63-3		$C_{13}H_{24}N_2O_3$
Nebracetam	Nootropic	97205-34-0		$C_{12}H_{16}N_2O$
Necopidem	Sedative	103844-77-5		$C_{23}H_{29}N_3O$
Nedocromil	Antihistaminic	69049-73-6		$C_{19}H_{17}NO_7$
Nefazodone	Antidepressant	83366-66-9		$C_{25}H_{32}ClN_5O_2$
Nefiracetam	Nootropic	77191-36-7		$C_{14}H_{18}N_2O_2$
Neflumozide	Antipsychotic	86636-93-3		C_{22}H23FN4O2

Table S1. *Cont.*

Common Name	Indication	CAS Number	Oral	Molecular Formula
Nefopam	Analgesic	13669-70-0		$C_{17}H_{19}NO$
Nelarabine	Antineoplastic	121032-29-9		$C_{11}H_{15}N_5O5$
Neldazosin	Antihypertensive	109713-79-3		$C_{18}H_{25}N_5O_4$
Nelezaprine Maleate	Muscle Relaxant	69624-60-8		$C_{18}H_{21}ClN_2$
Nelfinavir	Antiviral	159989-64-7	Y	$C_{32}H_{45}N_3O_4S$
Neltenexine	Respiratory Stimulant	99453-84-6		$C_{18}H_{20}Br_2N_2O_2S$
Nemadectin	Antiprotozoal	102130-84-7		$C_{36}H_{52}O_8$
Nemazoline	Decongestant	130759-56-7		$C_{10}H_{11}Cl_2N_3$
Nemifitide Ditriflutate	Antidepressant	173240-15-8		$C_{33}H_{43}FN_{10}O_6$
Nemonapride	Antiemetic	75272-39-8	Y	$C_{21}H_{26}ClN_3O_2$
Nemorubicin	Antineoplastic	108852-90-0		$C_{32}H_{37}NO_{13}$
Neoarsphenamine	Antibiotic	637-30-9		$C_{13}H_{14}As_2N_2O_4S$
Neocinchophen	Analgesic	485-34-7		$C_{19}H_{17}NO_2$
Neostigmine Bromide	Cholinergic	114-80-7		$C_{12}H_{19}BrN_2O_2$
Nepadutant	Bronchodilator	183747-35-5	Y	$C_{45}H_{58}N_{10}O_{13}$
Nepafenac	Antiinflammatory	78281-72-8		$C_{15}H_{14}N_2O_2$
Nepaprazole	Antiulcerative	156601-79-5		$C_{18}H_{19}N_3O_2S$
Nepicastat	Cardiotonic	173997-05-2	Y	$C_{14}H_{15}F_2N_3S$
Nepinalone	Hemostatic	22443-11-4	Y	$C_{18}H_{25}NO$
Nequinate	Antibacterial	13997-19-8		$C_{22}H_{23}NO_4$
Neramexane Mesylate	Antidepressant	219810-59-0	Y	$C_{11}H_{23}N$
Neraminol	Antihypotensive	86140-10-5		$C_{20}H_{26}N_4O_2$
Nerbacadol	Unclassified	99803-72-2		$C_{10}H_{14}N_2O_2$
Neridronic Acid	Bone Resorption Inhibitor	79778-41-9		$C_6H_{17}NO_7P_2$
Nerisopam	Anxiolytic	102771-12-0		C18H19N3O2
Nerispirdine	Analgesic	119229-65-1		$C_{17}H_{18}FN_3$
Nesapidil	Vasodilator	118778-75-9		$C_{23}H_{28}N_4O_4$
Nesosteine	Mucolytic	84233-61-4		$C_{11}H_{11}NO_3S$
Nestifylline	Bronchodilator	116763-36-1		$C_{11}H_{14}N_4O_2S_2$
Neticonazole	Antifungal	130726-68-0		$C_{17}H_{22}N_2OS$
Netilmicin	Antibiotic	56391-56-1		$C_{21}H_{41}N_5O_7$
Netivudine	Antiviral	84558-93-0		$C_{12}H_{14}N_2O_6$
Netobimin	Anthelminithic	88255-01-0		$C_{14}H_{20}N_4O_7S_2$
Netoglitazone	Antidiabetic	161600-01-7		$C_{21}H_{16}FNO_3S$
Netupitant	Antiemetic	290297-26-6		$C_{30}H_{32}F_6N_4O$
Neutramycin	Antibacterial	1404-08-6		$C_{34}H_{54}O_{14}$
Nevirapine	Antiviral	129618-40-2	Y	$C_{15}H_{14}N_4O$
Nexeridine	Analgesic	53716-48-6		$C_{19}H_{29}NO_2$
Nexopamil	Antiarrhythmic	136033-49-3		$C_{24}H_{40}N_2O_3$
Niacin	Dermatologic	59-67-6	Y	$C_6H_5NO_2$
Niacinamide	Dermatologic	98-92-0		$C_6H_6N_2O$
Nialamide	Antidepressant	51-12-7		$C_{16}H_{18}N_4O_2$
Niaprazine	Sedative	27367-90-4		$C_{20}H_{25}FN_4O$
Nibroxane	Antibacterial	53983-00-9		$C_5H_8BrNO_4$
Nicafenine	Analgesic	64039-88-9		$C_{24}H_{19}ClN_4O_3$
Nicainoprol	Antihypertensive	76252-06-7		$C_{21}H_{27}N_3O_3$
Nicametate	Vasodilator	3099-52-3		C_{12}H18N2O2
Nicanartine	Antihyperlipidemic	150443-71-3		$C_{23}H_{33}NO_2$
Nicaraven	Cardiotonic	79455-30-4		$C_{15}H_{16}N_4O_2$
Nicarbazin	Antibacterial	587-90-6		$C_{13}H_{10}N_4O_5$
Nicarbazin	Antibacterial	108-79-2		$C_6H_8N_2O$
Nicardipine	Antianginal	55985-32-5	Y	$C_{26}H_{29}N_3O_6$
Nicergoline	Vasodilator	27848-84-6	Y	$C_{24}H_{26}BrN_3O_3$
Niceritrol	Antihyperlipidemic	5868-05-3		$C_{29}H_{24}N_4O_8$
Niceverine	Antispasmodic	2545-24-6		$C_{30}H_{23}N_3O_6$
Niclofolan	Anthelminthic	10331-57-4		$C_{12}H_6Cl_2N_2O_6$
Niclosamide	Anthelminithic	50-65-7		$C_{13}H_8Cl_2N_2O_4$
Nicoboxil	Analgesic	13912-80-6		$C_{12}H_{17}NO_3$
Nicoclonate	Nootropic	10571-59-2		$C_{16}H_{16}ClNO_2$
Nicocodine	Antitussive	3688-66-2		$C_{24}H_{24}N_2O_4$
Nicocortonide	Glucocorticoid	65415-41-0		$C_{31}H_{37}NO_7$
Nicodicodine	Antitussive	808-24-2		$C_{24}H_{26}N_2O_4$
Nicofibrate	Antihyperlipidemic	31980-29-7		$C_{16}H_{16}ClNO_3$

Table S1. *Cont.*

Common Name	Indication	CAS Number	Oral	Molecular Formula
Nicofuranose	Vasodilator	15351-13-0		$C_{30}H_{24}N_4O_{10}$
Nicofurate	Unclassified	4397-91-5		$C_{35}H_{28}N_4O_{11}$
Nicogrelate	Antithrombotic	80614-21-7		$C_{17}H_{21}N_3O_2$
Nicomol	Capillary Protectant	27959-26-8		$C_{34}H_{32}N_4O_9$
Nicomorphine	Analgesic	639-48-5		$C_{29}H_{25}N_3O_5$
Nicopholine	Analgesic	492-85-3		$C_{10}H_{12}N_2O_2$
Nicoracetam	Nootropic	128326-80-7		$C_{11}H_{12}N_2O_3$
Nicorandil	Vasodilator	65141-46-0	Y	$C_8H_9N_3O_4$
Nicothiazone	Unclassified	555-90-8		$C_7H_8N_4S$
Nicotine	Nootropic	54-11-5	Y	$C_{10}H_{14}N_2$
Nicotinyl Alcohol	Vasodilator	100-55-0		C_6H_7NO
Nicotredole	Unclassified	29876-14-0		$C_{16}H_{15}N_3O$
Nicoxamat	Unclassified	5657-61-4		C_6H_{6N2O2}
Nictiazem	Antihypertensive	95058-70-1		$C_{26}H_{27}N_3O_4S$
Nictindole	Antithrombotic	36504-64-0		$C_{17}H_{16}N_2O$
Nifedipine	Antianginal	21829-25-4	Y	$C_{17}H_{18}N_2O_6$
Nifekalant	Cardiotonic	130636-43-0		$C_{19}H_{27}N_5O_5$
Nifenalol	Antiarrhythmic	7413-36-7		$C_{11}H_{16}N_2O_3$
Nifenazone	Antiinflammatory	2139-47-1		$C_{17}H_{16}N_4O_2$
Niflumic Acid	Antiinflammatory	4394-00-7		$C_{13}H_9F_3N_2O_2$
Nifluridide	Ectoparasiticide	61444-62-0		$C_{10}H_6F_7N_3O_3$
Nifuradene	Antibiotic	555-84-0		$C_8H_8N_4O_4$
Nifuraldezone	Antibiotic	3270-71-1		$C_7H_6N_4O_5$
Nifuralide	Antibiotic	54657-96-4		$C_{14}H_{13}N_5O_4S$
Nifuratel	Antibiotic	4936-47-4		$C_{10}H_{11}N_3O_5S$
Nifuratrone	Antibiotic	19561-70-7		$C_7H_8N_2O_5$
Nifurdazil	Antibiotic	5036-03-3		$C_{10}H_{12}N_4O_5$
Nifurethazone	Antibiotic	5580-25-6		$C_{10}H_{15}N_5O_4$
Nifurfoline	Antibiotic	3363-58-4		$C_{13}H_{15}N_5O_6$
Nifurimide	Antibiotic	21638-36-8		$C_9H_{10}N_4O_4$
Nifurizone	Antibiotic	26350-39-0		$C_{12}H_{13}N_5O_5$
Nifurmazole	Antibiotic	18857-59-5		$C_{11}H_{10}N_4O_6$
Nifurmerone	Antibiotic	5579-95-3		$C_6H_4ClNO_4$
Nifuroquine	Antibiotic	57474-29-0		$C_{14}H_8N_2O_6$
Nifuroxazide	Antibiotic	965-52-6		$C_{12}H_9N_3O_5$
Nifuroxime	Antibacterial	6236-05-1		$C_5H_4N_2O_4$
Nifurpipone	Antibiotic	24632-47-1		$C_{12}H_{17}N_5O_4$
Nifurpirinol	Antibiotic	13411-16-0		$C_{12}H_{10}N_2O_4$
Nifurprazine	Antibiotic	1614-20-6		$C_{10}H_8N_4O_3$
Nifurquinazol	Antibiotic	5055-20-9		$C_{16}H_{16}N_4O_5$
Nifursemizone	Antiprotozoal	5579-89-5		$C_8H_{10}N_4O_4$
Nifursol	Antiprotozoal	16915-70-1		$C_{12}H_7N_5O_9$
Nifurthiazole	Antibiotic	3570-75-0		$C_8H_6N_4O_4S$
Nifurtimox	Antibiotic	23256-30-6	Y	$C_{10}H_{13}N_3O_5S$
Nifurtoinol	Antibiotic	1088-92-2		$C_9H_8N_4O_6$
Nifurvidine	Antibiotic	1900-13-6		$C_{11}H_9N_3O_4$
Nifurzide	Antibiotic	39978-42-2	Y	$C_{12}H_8N_4O_6S$
Niguldipine	Antihypertensive	113165-32-5		$C_{36}H_{39}N_3O_6$
Nihydrazone	Antibacterial	67-28-7		$C_7H_7N_3O_4$
Nikethamide	Nootropic	59-26-7		$C_{10}H_{14}N_2O$
Nileprost	Prostaglandin	71097-83-1		$C_{22}H_{33}NO_5$
Nilprazole	Antiulcerative	60662-19-3		$C_{26}H_{33}N_5O_2$
Niludipine	Antianginal	22609-73-0		$C_{25}H_{34}N_2O_8$
Nilutamide	Antineoplastic	63612-50-0	Y	$C_{12}H_{10}F_3N_3O_4$
Nilvadipine	Antianginal	75530-68-6	Y	$C_{19}H_{19}N_3O_6$
Nimazone	Antiinflammatory	17230-89-6		$C_{11}H_9ClN_4O$
Nimesulide	Antiinflammatory	51803-78-2	Y	$C_{13}H_{12}N_2O_5S$
Nimetazepam	Muscle Relaxant	2011-67-8		$C_{16}H_{13}N_3O_3$
Nimidane	Antiprotozoal	50435-25-1		$C_9H_8ClNS_2$
Nimodipine	Vasodilator	66085-59-4	Y	$C_{21}H_{26}N_2O_7$
Nimorazole	Antiprotozoal	6506-37-2		$C_9H_{14}N_4O_3$
Nimustine	Antineoplastic	42471-28-3		$C_9H_{13}ClN_6O_2$
Niometacin	Antiinflammatory	16426-83-8		C18H16N2O4

Table S1. *Cont.*

Common Name	Indication	CAS Number	Oral	Molecular Formula
Niperotidine	Antiulcerative	84845-75-0		$C_{20}H_{26}N_4O_5S$
Nipradilol	Antianginal	81486-22-8	Y	$C_{15}H_{22}N_2O_6$
Niprofazone	Antiinflammatory	15387-10-7		$C_{21}H_{25}N_5O_2$
Niravoline	Diuretic	130610-93-4		$C_{22}H_{25}N_3O_3$
Niridazole	Anthelminithic	61-57-4		$C_6H_6N_4O_3S$
Nisbuterol Mesylate	Bronchodilator	57540-78-0		$C_{22}H_{27}NO_6$
Nisobamate	Sedative	25269-04-9		$C_{13}H_{26}N_2O_4$
Nisoldipine	Antihypertensive	63675-72-9	Y	$C_{20}H_{24}N_2O_6$
Nisoxetine	Antidepressant	53179-07-0		$C_{17}H_{21}NO_2$
Nisterime Acetate	Androgen	51354-31-5		$C_{27}H_{35}N_2O_5$
Nitarsone	Antiprotozoal	98-72-6		$C_6H_6AsNO_5$
Nitazoxanide	Anthelminithic	55981-09-4	Y	$C_{12}H_9N_3O_5S$
Nitecapone	Capillary Protectant	116313-94-1		$C_{12}H_{11}NO_6$
Nithiamide	Antiprotozoal	140-40-9		$C_5H_5N_3O_3S$
Nitisinone	Dermatologic	104206-65-7	Y	$C_{14}H_{10}F_3NO_5$
Nitracrine	Antidepressant	4533-39-5		$C_{18}H_{20}N_4O_2$
Nitrafudam	Antidepressant	64743-09-5		$C_{11}H_9N_3O_3$
Nitralamine	Antifungal	71872-90-7		$C_{10}H_{13}ClN_2O_2S$
Nitramisole	Anthelminithic	6646-49-7		$C_{11}H_{11}N_3O_2S$
Nitraquazone	Antiinflammatory	56739-21-0		$C_{16}H_{13}N_3O_4$
Nitrazepam	Anticonvulsant	146-22-5	Y	$C_{15}H_{11}N_3O_3$
Nitrefazole	Alcohol Deterrant	21721-92-6		$C_{10}H_8N_4O_4$
Nitrendipine	Antihypertensive	39562-70-4	Y	$C_{18}H_{20}N_2O_6$
Nitricholine Perchlorate	Cholinergic	7009-91-8		$C_5H_{13}ClN_2O_7$
Nitrocefin	Antibiotic	41906-86-9		$C_{21}H_{16}N_4O_8S_2$
Nitroclofene	Anthelminithic	39224-48-1		$C_{13}H_8Cl_2N_2O_6$
Nitrocycline	Antibacterial	5585-59-1		C_{21}H21N3O9
Nitrodan	Anthelminithic	962-02-7		$C_{10}H_8N_4O_3$S2
Nitrofurantoin	Antibiotic	67-20-9	Y	$C_8H_6N_4O_5$
Nitroglycerin	Antianginal	55-63-0		$C_3H_5N_3O_9$
Nitromide	Antibacterial	121-81-3		$C_7H_5N_3O_5$
Nitromifene	Steroid	10448-84-7		$C_{27}H_{28}N_2O_4$
Nitroscanate	Anthelminithic	19881-18-6		$C_{13}H_8N_2O_3S$
Nitrovin	Antibacterial	804-36-4		$C_{14}H_{12}N_6O_6$
Nitroxinil	Anthelminthic	1689-89-0		$C_7H_3IN_2O_3$
Nitroxoline	Antibiotic	4008-48-4		$C_9H_6N_2O_3$
Nivazol	Glucocorticoid	24358-76-7		$C_{28}H_{31}FN_2O$
Nixylic Acid	Unclassified	4394-05-2		$C_{14}H_{14}N_2O_2$
Nizatidine	Antiulcerative	76963-41-2	Y	$C_{12}H_{21}N_5O_2S_2$
Nizofenone	Nootropic	54533-85-6		$C_{21}H_{21}ClN_4O_3$
N Methylepinephrine	Antihypotensive	554-99-4		$C_{10}H_{15}NO_3$
N Myristyl 3 Hydroxybutylamine	Unclassified	143-26-0		$C_{18}H_{39}NO$
Noberastine	Antihistaminic	110588-56-2		$C_{17}H_{21}N_5O$
Nocloprost	Prostaglandin	79360-43-3		$C_{22}H_{37}ClO_4$
Nocodazole	Antineoplastic	31430-18-9		$C_{14}H_{11}N_3O_3S$
Nofecainide	Antiarrhythmic	50516-43-3		$C_{20}H_{24}N_2O_3$
Nogalamycin	Antineoplastic	1404-15-5		$C_{39}H_{49}NO_{16}$
Nolatrexed	Antineoplastic	147149-76-6		$C_{14}H_{12}N_4OS$
Nolinium Bromide	Antiulcerative	40759-33-9		$C_{15}H_{11}BrCl_2N_2$
Nolomirole	Cardiotonic	90060-42-7		$C_{19}H_{27}NO_4$
Nolpitantium Besilate	Bronchodilator	155418-06-7		$C_{43}H_{50}Cl_2N_2O_5S$
Nomegestrol Acetate	Progestogen	58652-20-3		$C_{23}H_{30}O_4$
Nomelidine	Anesthetic	60324-59-6		$C_{15}H_{15}BrN_2$
Nomifensine	Antidepressant	24526-64-5		$C_{16}H_{18}N_2$
Nonabine	Antiemetic	16985-03-8		$C_{25}H_{33}NO_2$
Nonaperone	Antipsychotic	15997-76-9		$C_{18}H_{24}FNO$
Nonapyrimine	Unclassified	5626-36-8		$C_{15}H_{24}N_4$
Nonathymulin	Thyroid	63958-90-7		$C_{33}H_{54}N_{12}O_{15}$
Nonivamide	Nootropic	2444-46-4		$C_{17}H_{27}NO_3$
Noracymethadol	Analgesic	1477-39-0		$C_{22}H_{29}NO_2$
Norbolethone	Steroid	1235-15-0		$C_{21}H_{32}O_2$
Norbudrine	Antihypotensive	127560-12-7		$C_{12}H_{17}NO_3$
Norclostebol	Steroid	13583-21-6		$C_{18}H_{25}ClO_2$

Table S1. *Cont.*

Common Name	Indication	CAS Number	Oral	Molecular Formula
Norcodeine	Analgesic	467-15-2		$C_{17}H_{19}NO_3$
Nordazepam	Anxiolytic	1088-11-5	Y	$C_{15}H_{11}ClN_2O$
Nordefrin	Antihypotensive	74812-63-8		$C_9H_{13}NO_3$
Nordihydroguaiaretic Acid	Antiinflammatory	500-38-9		$C_{18}H_{22}O_4$
Norelgestromin	Contraceptive	53016-31-2		$C_{21}H_{29}NO_2$
Norepinephrine	Antihypotensive	51-41-2		$C_8H_{11}NO_3$
Norethandrolone	Androgen	52-78-8		$C_{20}H_{30}O_2$
Norethindrone	Progestogen	68-22-4	Y	$C_{20}H_{26}O_2$
Norethindrone Acetate	Progestogen	51-98-9		$C_{22}H_{28}O_3$
Norethynodrel	Progestogen	68-23-5		$C_{20}H_{26}O_2$
Noreximide	Sedative	6319-06-8		C_9H9NO2
Norfenefrine	Antihypotensive	536-21-0		$C_8H_{11}NO_2$
Norfloxacin	Antibiotic	70458-96-7	Y	$C_{16}H_{18}FN_3O_3$
Norfloxacin Succinil	Antibiotic	100587-52-8		$C_{20}H_{22}FN_3$O6
Norflurane	Anesthetic	811-97-2		$C_2H_2F_4$
Norgesterone	Progestogen	13563-60-5		$C_{20}H_{28}O_2$
Norgestimate	Progestogen	35189-28-7		$C_{23}H_{31}NO_3$
Norgestomet	Progestogen	25092-41-5		$C_{23}H_{32}O_4$
Norgestrel	Progestogen	6533-00-2		$C_{21}H_{28}O_2$
Norgestrienone	Progestogen	848-21-5		$C_{20}H_{22}O_2$
Norletimol	Unclassified	886-08-8		$C_{14}H_{13}NO$
Norlevorphanol	Analgesic	1531-12-0		$C_{16}H_{21}NO$
Normethadone	Antitussive	467-85-6		$C_{20}H_{25}NO$
Normethandrone	Androgen	514-61-4		$C_{19}H_{28}O_2$
Normorphine	Analgesic	466-97-7		$C_{16}H_{17}NO_3$
Norpipanone	Analgesic	561-48-8		$C_{23}H_{29}NO$
Nortetrazepam	Anxiolytic	10379-11-0		$C_{15}H_{15}ClN_2O$
Nortopixantrone	Antineoplastic	156090-17-4		C20H24N6O2
Nortriptyline	Antidepressant	72-69-5	Y	$C_{19}H_{21}N$
Norvinisterone	Progestogen	6795-60-4		$C_{20}H_{28}O_2$
Nosantine	Antineoplastic	76600-30-1		$C_{14}H_{22}N_4O_2$
Noscapine	Antitussive	128-62-1	Y	$C_{22}H_{23}NO_7$
Novobiocin	Antibacterial	303-81-1		$C_{31}H_{36}N_2O_{11}$
Noxiptiline	Antidepressant	3362-45-6		$C_{19}H_{22}N_2O$
Nuclomedone	Antihistamine	75963-52-9		$C_{13}H_{11}ClN_2O_2S$
Nuclotixene	Antidepressant	36471-39-3		$C_{21}H_{20}ClNS$
Nufenoxole	Gastroprokinetic	57726-65-5		$C_{25}H_{29}N_3O$
Nupafant	Antithrombotic	139133-27-0		$C_{23}H_{32}N_4O_3S$
Nuvenzepine	Gastroprokinetic	96487-37-5		$C_{19}H_{20}N_4O_2$
Nylestriol	Estrogen	39791-20-3		$C_{25}H_{32}O_3$
Nylidrin	Vasodilator	447-41-6		$C_{19}H_{25}NO_2$
Oberadilol	Antianginal	114856-44-9	Y	$C_{25}H_{30}ClN_5O_3$
Obidoxime Chloride	Antidote	114-90-9		$C_{14}H_{16}Cl_2N_4O_3$
Ocaperidone	Antipsychotic	129029-23-8		$C_{24}H_{25}FN_4O_2$
Ocfentanil	Analgesic	101343-69-5		$C_{22}H_{27}FN_2O_2$
Ociltide	Antidiabetic	78410-57-8		$C_{31}H_{40}N_6O_7S$
Ocinaplon	Anxiolytic	96604-21-6		$C_{17}H_{11}N_5O$
Ocrylate	Dermatologic	6701-17-3		$C_{12}H_{19}NO_2$-
Octabenzone	Dermatologic	1843-05-6		$C_{21}H_{26}O_3$
Octacaine	Anesthetic	13912-77-1		$C_{14}H_{22}N_2O$
Octadecyltrimethylammonium Pentachlorophenate	Unclassified	488-40-4		$C_{27}H_{46}Cl_5NO$
Octamoxin	Antidepressant	4684-87-1		$C_8H_{20}N_2$
Octamylamine	Antispasmodic	502-59-0		$C_{13}H_{29}N$
Octanoic Acid	Antifungal	124-07-2		$C_8H_{16}O_2$
Octapinol	Antibacterial	71138-71-1		$C_{15}H_{31}NO$
Octastine	Antihistaminic	59767-12-3		$C_{23}H_{30}ClNO$
Octaverine	Antispasmodic	549-68-8		$C_{23}H_{27}NO_5$
Octazamide	Analgesic	56391-55-0		$C_{13}H_{15}NO_2$
Octimibate	Anticoagulant	89838-96-0		$C_{29}H_{30}N_2O_3$
Octinoxate	Dermatologic	5466-77-3		$C_{18}H_{26}O_3$
Octisalate	Dermatologic	118-60-5		$C_{15}H_{22}O_3$
Octocrylene	Dermatologic	6197-30-4		$C_{24}H_{27}NO_2$

Table S1. *Cont.*

Common Name	Indication	CAS Number	Oral	Molecular Formula
Octodrine	Decongestant	543-82-8		$C_8H_{19}N$
Octopamine	Antihypotensive	104-14-3		$C_8H_{11}NO_2$
Octriptyline Phosphate	Antidepressant	47166-67-6		$C_{20}H_{21}N$
Octrizole	Dermatologic	3147-75-9		$C_{20}H_{25}N_3O$
Odalprofen	Analgesic	137460-88-9		$C_{20}H_{20}N_2O_2$
Odapipam	Sedative	131796-63-9		C_{19}H20ClNO2
Odiparcil	Antithrombotic	137215-12-4		$C_{15}H_{16}O_6S$
Ofloxacin	Antibiotic	82419-36-1	Y	$C_{18}H_{20}FN_3O_4$
Ofornine	Antihypertensive	87784-12-1		$C_{17}H_{19}N_3O$
Oftasceine	Antineoplastic	1461-15-0		$C_{30}H_{26}N_2O_{13}$
Oglufanide Disodium	Immunomodulator	38101-59-6		$C_{16}H_{19}N_3O_5$
Olaflur	Antibacterial	17671-49-7		$C_{27}H_{58}N_2O_3$
Olamufloxacin	Antibiotic	167887-97-0		$C_{20}H_{23}FN_4O_3$
Olanexidine	Antibacterial	146510-36-3		$C_{17}H_{27}Cl_2N_5$
Olanzapine	Antipsychotic	132539-06-1	Y	$C_{17}H_{20}N_4S$
Olaquindox	Antibacterial	23696-28-8		$C_{12}H_{13}N_3O_4$
Olcegepant	Antimigraine	204697-65-4		$C_{38}H_{47}Br_2N_9O_5$
Oleandomycin Phosphate	Antibiotic	3922-90-5		C35H61NO12
Oletimol	Unclassified	5879-67-4		$C_{15}H_{15}NO$
Olmesartan	Antihypertensive	144689-24-7	Y	$C_{24}H_{26}N_6O_3$
Olmesartan Medoxomil	Antihypertensive	144689-63-4	Y	$C_{29}H_{30}N_6O_6$
Olmidine	Antihypertensive	31105-14-3		$C_9H_{10}N_2O_3$
Olopatadine	Antihistaminic	113806-05-6	Y	$C_{21}H_{23}NO_3$
Olpadronic Acid	Bone Resorption Inhibitor	63132-39-8		$C_5H_{15}NO_7$P2
Olpimedone	Antihistaminic	39567-20-9		$C_7H_{10}N_2OS$
Olprinone	Cardiotonic	106730-54-5		C14H10N4O
Olradipine	Antiparkinsonian	115972-78-6		$C_{22}H_{28}Cl_2N_2O_6$
Olsalazine	Antiinflammatory	15722-48-2		$C_{14}H_{10}N_2O_6$
Oltipraz	Antiviral	64224-21-1		$C_8H_6N_2S_3$
Olvanil	Analgesic	58493-49-5		$C_{26}H_{43}NO_3$
Omaciclovir	Antiviral	124265-89-0		$C_{10}H_{15}N_5O_3$
Omapatrilat	Antihypertensive	167305-00-2		$C_{19}H_{24}N_2O_4S_2$
Omeprazole	Antiulcerative	73590-58-6	Y	$C_{17}H_{19}N_3O_3S$
Omidoline	Analgesic	21590-91-0		$C_{22}H_{27}N_3O_2$
Omigapil	Nootropic	181296-84-4		$C_{19}H_{17}NO$
Omiloxetine	Antidepressant	176894-09-0		$C_{27}H_{25}F_2NO_4$
Omoconazole Nitrate	Antifungal	74512-12-2		$C_{20}H_{17}Cl_3N_2O_2$
Omonasteine	Mucolytic	60175-95-3		C5H9NO2S
Onapristone	Progestogen	96346-61-1		$C_{29}H_{39}$NO3
Ondansetron	Antiemetic	99614-02-5	Y	$C_{18}H_{19}N_3O$
Ontazolast	Bronchodilator	147432-77-7		$C_{21}H_{25}N_3O$
Ontianil	Unclassified	35727-72-1		$C_{13}H_{12}ClNO_2S$
Opanixil	Antihypertensive	152939-42-9		$C_{19}H_{21}F_3N_6O_2$
Opaviraline	Antiviral	178040-94-3		$C_{14}H_{17}FN_2O_3$
Opiniazide	Antibacterial	2779-55-7		$C_{16}H_{15}N_3O_5$
Opipramol	Antidepressant	315-72-0	Y	$C_{23}H_{29}N_3O$
Opratonium Iodide	Cholinergic	146919-78-0		$C_{17}H_{35}N_2O.I$
Orazamide	Hepatic Protectant	360-97-4		$C_4H_6N_4O$
Orazipone	Antiinflammatory	137109-78-5		$C_{13}H_{14}O_4S$
Orbifloxacin	Antibiotic	113617-63-3		$C_{19}H_{20}F_3N_3O_3$
Orbofiban Acetate	Antithrombotic	163250-90-6		$C_{17}H_{23}N_5O_4$
Orbutopril	Antihypertensive	108391-88-4		$C_{20}H_{34}N_2O_5$
Orconazole Nitrate	Antifungal	22833-02-9		$C_{18}H_{15}Cl_3N_2O$
Orestrate	Unclassified	13885-31-9		$C_{27}H_{36}O_3$
Orlistat	Antiobesity	96829-58-2	Y	$C_{29}H_{53}NO_5$
Ormeloxifene	Bone Resorption Inhibitor	31477-60-8		$C_{30}H_{35}NO_3$
Ormetoprim	Antibacterial	6981-18-6		$C_{14}H_{18}N_4O_2$
Ornidazole	Antibacterial	16773-42-5		$C_7H_{10}ClN_3O_3$
Ornoprostil	Prostaglandin	70667-26-4		$C_{23}H_{38}O_6$
Orotic Acid	Uricosuric	65-86-1		$C_5H_4N_2O_4$
Orotirelin	Thyroid	62305-86-6		$C_{16}H_{19}N_7O_5$
Orpanoxin	Antiinflammatory	60653-25-0		$C_{13}H_{11}ClO_4$
Orphenadrine Citrate	Muscle Relaxant	83-98-7		$C_{18}H_{23}NO$

Table S1. *Cont.*

Common Name	Indication	CAS Number	Oral	Molecular Formula
Ortataxel	Antineoplastic	186348-23-2		$C_{44}H_{57}NO_{17}$
Ortetamine	Antineoplastic	5580-32-5		$C_{10}H_{15}N$
Osalmid	Choleretic	526-18-1		$C_{13}H_{11}NO_3$
Osanetant	Antihistaminic	160492-56-8		$C_{35}H_{41}Cl_2N_3O_2$
Osaterone	Steroid	105149-04-0		$C_{20}H_{25}ClO_4$
Oseltamivir	Antiviral	196618-13-0	Y	$C_{16}H_{28}N_2O_4$
Osemozotan	Anxiolytic	137275-81-1		$C_{19}H_{21}NO_5$
Osmadizone	Analgesic	27450-21-1		$C_{23}H_{22}N_2O_4S$
Ospemifene	Antineoplastic	128607-22-7	Y	$C_{24}H_{23}ClO_2$
Ostreogrycin	Antibiotic	21411-53-0		$C_{28}H_{35}N_3O_7$
Osutidine	Antiulcerative	140695-21-2		$C_{19}H_{28}N_4O_5S_2$
Otamixaban	Anticoagulant	193153-04-7		$C_{25}H_{26}N_4O_4$
Otenzepad	Mydriatic	102394-31-0		$C_{24}H_{31}N_5O_2$
Oteracil	Antineoplastic	937-13-3		$C_4H_3N_3O_4$
Otilonium Bromide	Antispasmodic	26095-59-0	Y	$C_{29}H_{43}BrN_2O_4$
Ouabain	Cardiotonic	630-60-4		$C_{29}H_{44}O_{12}$
Oxabolone Cipionate	Steroid	1254-35-9		$C_{26}H_{38}O_4$
Oxabrexine	Antidepressant	65415-42-1		$C_{18}H_{25}Br_2NO_3$
Oxaceprol	Antiinflammatory	33996-33-7		$C_7H_{11}NO_4$
Oxacillin	Antibiotic	66-79-5		$C_{19}H_{19}$N3O5S
Oxadimedine	Antineoplastic	16485-05-5		$C_{18}H_{21}N_3O$
Oxaflozane	Antidepressant	26629-87-8		$C_{14}H_{18}F_3NO$
Oxaflumazine	Antipsychotic	16498-21-8		$C_{26}H_{32}F_3N_3O_2S$
Oxagrelate	Antithrombotic	56611-65-5		$C_{14}H_{16}N_2O_4$
Oxalic Acid	Hemostatic	144-62-7		$C_2H_2O_4$
Oxalinast	Bronchodilator	70009-66-4		$C_{14}H_{13}NO_4$
Oxamarin	Hemostatic	15301-80-1		$C_{22}H_{34}N_2O_4$
Oxametacin	Antiinflammatory	27035-30-9		$C_{19}H_{17}ClN_2O_4$
Oxamisole	Immunomodulator	99258-56-7		C15H20N2O2
Oxamniquine	Anthelminithic	21738-42-1	Y	$C_{14}H_{21}N_3O_3$
Oxanamide	Anxiolytic	126-93-2		$C_8H_{15}NO_2$
Oxandrolone	Androgen	53-39-4	Y	$C_{19}H_{30}O_3$
Oxantel	Anthelminithic	36531-26-7	Y	$C_{13}H_{16}N_2O$
Oxapadol	Analgesic	56969-22-3		$C_{18}H_{16}N_2O_2$
Oxapium Iodide	Antispasmodic	6577-41-9		$C_{22}H_{34}INO_2$
Oxapropanium Iodide	Cholinergic	541-66-2		$C_7H_{16}INO_2$
Oxaprotiline	Antidepressant	56433-44-4		$C_{20}H_{23}NO$
Oxaprozin	Antiinflammatory	21256-18-8		$C_{18}H_{15}NO_3$
Oxarbazole	Bronchodilator	35578-20-2		$C_{21}H_{19}NO_4$
Oxatomide	Antihistaminic	60607-34-3	Y	$C_{27}H_{30}N_4O$
Oxazafone	Sedative	70541-17-2		$C_{19}H_{21}ClN_2O_3$
Oxazepam	Anxiolytic	604-75-1	Y	$C_{15}H_{11}ClN_2O_2$
Oxazidione	Analgesic	27591-42-0		$C_{20}H_{19}NO_3$
Oxazolam	Anxiolytic	24143-17-7		$C_{18}H_{17}ClN_2O_2$
Oxazorone	Unclassified	25392-50-1		$C_{14}H_{15}NO_4$
Oxcarbazepine	Anticonvulsant	28721-07-5	Y	$C_{15}H_{12}N_2O_2$
Oxdralazine	Diuretic	17259-75-5		$C_8H_{15}N_5O_2$
Oxedrine	Antihypotensive	94-07-5		$C_9H_{13}NO_2$
Oxeglitazar	Antidiabetic	280585-34-4		$C_{19}H_{22}O_4$
Oxeladin	Antitussive	468-61-1		$C_{20}H_{33}NO_3$
Oxendolone	Steroid	33765-68-3		$C_{20}H_{30}O_2$
Oxepinac	Antiinflammatory	55689-65-1		$C_{16}H_{12}O_4$
Oxetacillin	Antibiotic	53861-02-2		$C_{19}H_{23}N_3O_5S$
Oxethazaine	Anesthetic	126-27-2		$C_{28}H_{41}N_3O_3$
Oxetorone	Analgesic	26020-55-3		$C_{21}H_{21}NO_2$
Oxfendazole	Anthelminithic	53716-50-0		$C_{15}H_{13}N_3O_3S$
Oxfenicine	Vasodilator	32462-30-9		$C_8H_9NO_3$
Oxibendazole	Anthelminithic	20559-55-1		$C_{12}H_{15}N_3O_3$
Oxibetaine	Antibacterial	7002-65-5		$C_6H_{13}NO_3$
Oxiconazole	Antifungal	64211-45-6		$C_{18}H_{13}Cl_4N_3O$
Oxidopamine	Opthalmic	1199-18-4		$C_8H_{11}NO_3$
Oxidronic Acid	Bone Resorption Inhibitor	15468-10-7		$CH_6O_7P_2$
Oxifentorex	Anorexic	4075-88-1		$C_{17}H_{21}NO$

Table S1. *Cont.*

Common Name	Indication	CAS Number	Oral	Molecular Formula
Oxifungin	Antifungal	64057-48-3		$C_{13}H_{12}N_4O$
Oxiglutatione	Unclassified	27025-41-8		$C_{20}H_{32}N_6O_{12}S_2$
Oxilofrine	Mydriatic	365-26-4		$C_{10}H_{15}NO_2$
Oxilorphan	Antidote	42281-59-4		$C_{20}H_{27}NO_2$
Oximonam	Antibacterial	90898-90-1		$C_{12}H_{15}N_5O_6S$
Oxindanac	Antiinflammatory	68548-99-2		$C_{17}H_{14}O_4$
Oxiniacic Acid	Antihyperlipidemic	2398-81-4		$C_6H_5NO_3$
Oxiperomide	Antipsychotic	5322-53-2		$C_{20}H_{23}N_3O_2$
Oxiracetam	Nootropic	62613-82-5	Y	$C_6H_{10}N_2O_3$
Oxiramide	Antiarrhythmic	13958-40-2		$C_{25}H_{34}N_2O_2$
Oxisopred	Unclassified	18118-80-4		$C_{21}H_{28}O_6$
Oxisuran	Antineoplastic	27302-90-5		$C_8H_9NO_2S$
Oxitefonium Bromide	Antispasmodic	17692-63-6		$C_{19}H_{26}BrNO_3S$
Oxitriptan	Anticonvulsant	4350-09-8		$C_{11}H_{12}N_2O_3$
Oxitriptyline	Antidepressant	29541-85-3		$C_{19}H_{21}NO_2$
Oxitropium Bromide	Bronchodilator	30286-75-0		$C_{19}H_{26}BrNO_4$
Oxmetidine	Antiulcerative	72830-39-8		$C_{19}H_{21}N_5O_3S$
Oxodipine	Antianginal	90729-41-2		$C_{19}H_{21}NO_6$
Oxogestone Phenpropionate	Progestogen	16915-80-3		$C_{29}H_{38}O_3$
Oxolamine	Antiinflammatory	959-14-8		$C_{14}H_{19}N_3O$
Oxolinic Acid	Antibiotic	14698-29-4		$C_{13}H_{11}NO_5$
Oxomemazine	Antihistaminic	3689-50-7		$C_{18}H_{22}N_2O_2S$
Oxonazine	Antimigraine	5580-22-3		$C_9H_{14}N_6O$
Oxophenarsine	Antiprotozoal	306-12-7		$C_6H_6AsNO_2$
Oxoprostol	Antiulcerative	69648-40-4		$C_{22}H_{32}O_4$
Oxpheneridine	Analgesic	546-32-7		$C_{22}H_{27}NO_3$
Oxprenoate Potassium	Antihypertensive	786592-95-8		$C_{25}H_{38}O_4$
Oxprenolol	Antihypertensive	6452-71-7	Y	$C_{15}H_{23}NO_3$
Oxtriphylline	Bronchodilator	4499-40-5		$C_{12}H_{21}N_5O_3$
Oxybenzone	Dermatologic	131-57-7		$C_{14}H_{12}O_3$
Oxybutynin	Urologic	5633-20-5	Y	$C_{22}H_{31}NO_3$
Oxycinchophen	Uricosuric	485-89-2		$C_{16}H_{11}NO_3$
Oxyclozanide	Anthelminthic	2277-92-1		$C_{13}H_6Cl_5NO_3$
Oxycodone	Analgesic	76-42-6	Y	$C_{18}H_{21}NO_4$
Oxydipentonium Chloride	Muscle Relaxant	7174-23-4		$C_{16}H_{38}N_2OCl_2$
Oxyfedrine	Cardiotonic	15687-41-9		$C_{19}H_{23}NO_3$
Oxymesterone	Steroid	145-12-0		$C_{20}H_{30}O_3$
Oxymetazoline	Decongestant	1491-59-4		$C_{16}H_{24}N_2O$
Oxymetholone	Androgen	434-07-1	Y	$C_{21}H_{32}O_3$
Oxymorphone	Analgesic	76-41-5		$C_{17}H_{19}NO_4$
Oxypendyl	Antiemetic	5585-93-3		$C_{20}H_{26}N_4OS$
Oxypertine	Antidepressant	153-87-7		$C_{23}H_{29}N_3O_2$
Oxyphenbutazone	Antiinflammatory	129-20-4		$C_{19}H_{20}N_2O_3$
Oxyphencyclimine	Antispasmodic	125-53-1		$C_{20}H_{28}N_2O_3$
Oxyphenisatin	Laxative	125-13-3		$C_{20}H_{15}NO_3$
Oxyphenonium	Antispasmodic	14214-84-7		$C_{21}H_{34}NO_3$
Oxypurinol	Antiurolithic	2465-59-0		$C_5H_4N_4O_2$
Oxyridazine	Antipsychotic	14759-04-7		$C_{21}H_{26}N_2OS$
Oxysonium Iodide	Antibacterial	3569-58-2		$C_{18}H_{27}O_3S.I$
Oxytetracycline	Antibiotic	79-57-2	Y	$C_{22}H_{24}N_2O_9$
Ozagrel	Antianginal	82571-53-7	Y	$C_{13}H_{12}N_2O_2$
Ozolinone	Diuretic	56784-39-5		$C_{11}H_{16}N_2O_3S$
Paclitaxel	Antineoplastic	33069-62-4	Y	$C_{47}H_{51}NO_{14}$
Paclitaxel Ceribate	Antineoplastic	186040-50-6		$C_{51}H_{57}NO_{18}$
Pacrinolol	Antihypertensive	65655-59-6		$C_{23}H_{28}N_2O_4$
Pactimibe	Antihyperlipidemic	189198-30-9		$C_{25}H_{40}N_2O_3$
Padimate A	Dermatologic	21245-01-2		$C_{14}H_{21}NO_2$
Padimate O	Dermatologic	21245-02-3		$C_{17}H_{27}NO_2$
Pafenolol	Antihypertensive	75949-61-0		$C_{18}H_{31}N_3O_3$
Pagoclone	Anxiolytic	133737-32-3	Y	$C_{23}H_{22}ClN_3O_2$
Palatrigine	Anticonvulsant	98410-36-7		$C_{12}H_{13}Cl_2N_5$
Palinavir	Antiviral	154612-39-2		$C_{41}H_{52}N_6O_5$
Paliperidone	Antipsychotic	144598-75-4	Y	$C_{23}H_{27}FN_4O_3$

Table S1. *Cont.*

Common Name	Indication	CAS Number	Oral	Molecular Formula
Paliperidone Palmitate	Antipsychotic	199739-10-1	Y	$C_{39}H_{57}FN_4O_4$
Paliroden	Nootropic	188396-77-2		$C_{26}H_{24}F_3N$
Palmidrol	Oxytocic	544-31-0		$C_{18}H_{37}NO_2$
Palonidipine	Antianginal	96515-73-0		$C_{29}H_{34}FN_3O_6$
Palonosetron	Antiemetic	135729-61-2		$C_{19}H_{24}N_2O$
Palosuran	Antineoplastic	540769-28-6		$C_{25}H_{30}N_4O_2$
Pamabrom	Diuretic	10381-75-6		$C_7H_7BrN_4O_2$
Pamaqueside	Antihyperlipidemic	150332-35-7		$C_{39}H_{62}O_{14}$
Pamaquine	Antimalarial	491-92-9		$C_{19}H_{29}N_3O$
Pamatolol Sulfate	Antihypertensive	59110-35-9		$C_{16}H_{26}N_2O_4$
Pamicogrel	Antiinflammatory	101001-34-7		$C_{25}H_{24}N_2O_4S$
Pamidronic Acid	Bone Resorption Inhibitor	40391-99-9		$C_3H_{11}NO_7P_2$
Panadiplon	Anxiolytic	124423-84-3		$C_{18}H_{17}N_5O_2$
Panamesine	Antipsychotic	139225-22-2		$C_{23}H_{26}N_2O_6$
Pancopride	Antiemetic	121650-80-4		$C_{18}H24ClN3O2$
Pancuronium	Neuromuscular Blocking Agent	15500-66-0		C35H60Br2N2O4
Panidazole	Antiprotozoal	13752-33-5		$C_{11}H_{12}N_4O_2$
Panipenem	Antibiotic	87726-17-8		$C_{15}H_{21}N_3O_4S$
Panomifene	Steroid	77599-17-8		$C_{25}H_{24}F_3NO_2$
Pantethine	Antihyperlipidemic	16816-67-4		$C_{22}H_{42}N_4O_8S_2$
Pantoprazole	Antiulcerative	102625-70-7	Y	$C_{16}H_{15}F_2N_3O_4S$
Panuramine	Antidepressant	80349-58-2		$C_{24}H_{25}N_3O_2$
Papaverine	Vasodilator	58-74-2	Y	$C_{20}H_{21}NO_4$
Papaveroline	Muscle Relaxant	574-77-6		$C_{16}H_{13}NO_4$
Paraflutizide	Diuretic	1580-83-2		$C_{14}H_{13}ClFN_3O_4S_2$
Paraldehyde	Sedative	123-63-7		$C_6H_{12}O_3$
Paramethadione	Anticonvulsant	115-67-3		$C_7H_{11}NO_3$
Paramethasone Acetate	Glucocorticoid	1597-82-6		$C_{24}H_{31}FO_6$
Paranyline	Antiinflammatory	1729-61-9		$C_{21}H_{16}N_2$
Parapenzolate Bromide	Mydriatic	5634-41-3		$C_{21}H_{26}NO_3Br$
Parapropamol	Unclassified	1693-37-4		$C_9H_{11}NO_2$
Pararosaniline Pamoate	Anthelminithic	479-73-2		$C_{19}H_{17}N_3$
Paraxazone	Antidepressant	26513-79-1		$C_{10}H_{10}N_2O_3$
Parbendazole	Anthelminithic	14255-87-9		$C_{13}H_{17}N_3O_2$
Parcetasal	Unclassified	87549-36-8		$C_{17}H_{15}NO_5$
Parconazole	Antifungal	68685-54-1		$C_{17}H_{16}Cl_2N_2O_3$
Parecoxib	Antiinflammatory	198470-84-7		$C_{19}H_{18}N_2O_4S$
Pareptide Sulfate	Antiparkinsonian	61484-38-6		$C_{14}H_{26}N_4O_3$
Parethoxycaine	Analgesic	94-23-5		$C_{15}H_{23}NO_3$
Pargeverine	Antispasmodic	13479-13-5		$C_{21}H_{23}NO_3$
Pargolol	Antianginal	47082-97-3		$C_{16}H_{23}NO_3$
Pargyline	Antihypertensive	555-57-7		$C_{11}H_{13}N$
Paricalcitol	Pituitary	131918-61-1		$C_{27}H_{44}O_3$
Paridocaine	Analgesic	7162-37-0		$C_{17}H_{26}N_2O_2$
Parodilol	Antihypertensive	113854-64-1		$C_{23}H_{27}N_3O_2$
Paromomycin	Antibiotic	7542-37-2		$C_{23}H_{45}N_5O_{14}$
Paroxetine	Antidepressant	61869-08-7	Y	$C_{19}H_{20}FNO_3$
Paroxypropione	Antispasmodic	70-70-2		C_9H10O2
Parsalmide	Antifungal	30653-83-9		$C_{14}H_{18}N_2O_2$
Parvaquone	Antiamebic	4042-30-2		$C_{16}H_{16}O_3$
Pasiniazid	Antibacterial	65-49-6		$C_7H_7NO_3$
Patamostat	Antiviral	114568-26-2		$C_{20}H_{20}N_4O_4S$
Patupilone	Antineoplastic	152044-54-7		$C_{27}H_{41}NO_6S$
Pavatrin	Muscle Relaxant	4425-78-9		$C_{20}H_{23}NO_2$
Pavatrine	Muscle Relaxant	4425-78-9		$C_{20}H_{23}NO_2$
Paxamate	Antihistaminic	5579-05-5		$C_{14}H_{13}NO_2$
Pazelliptine	Antineoplastic	65222-35-7		$C_{22}H_{27}N_5$
Pazinaclone	Anxiolytic	103255-66-9		$C_{25}H_{23}ClN_4O4$
Pazopanib	Antineoplastic	444731-52-6	Y	$C_{21}H_{23}N_7O_2S$
Pazoxide	Antihypertensive	21132-59-2		$C_{12}H_{10}Cl_2N_2O_2S$
Pazufloxacin	Antibiotic	127045-41-4		$C_{16}H_{15}FN_2O_4$
Pecilocin	Antibiotic	19504-77-9		$C_{17}H_{25}NO_3$

Table S1. *Cont.*

Common Name	Indication	CAS Number	Oral	Molecular Formula
Pecocycline	Antibiotic	15301-82-3		$C_{29}H_{35}N_3O_{10}$
Pefloxacin	Antibacterial	70458-92-3	Y	$C_{17}H_{20}FN_3O_3$
Pegamotecan	Antineoplastic	203066-49-3		$C_{52}H_{48}N_6O_{14}$
Pelanserin	Antihypertensive	2208-51-7		$C_{21}H_{24}N_4O_2$
Pelargonic Acid	Unclassified	112-05-0		$C_9H_{18}O_2$
Peldesine	Antineoplastic	133432-71-0		$C_{12}H_{11}N_5O$
Peliglitazar	Antidiabetic	331744-64-0		$C_{30}H_{30}N_2O_7$
Pelitinib	Antineoplastic	257933-82-7		$C_{24}H_{23}ClFN_5O_2$
Pelitrexol	Antineoplastic	446022-33-9		$C_{20}H_{25}N_5O_6S$
Pelretin	Dermatologic	91587-01-8		$C_{23}H_{28}O_2$
Pelrinone	Cardiotonic	94386-65-9		$C_{12}H_{11}N_5O$
Pelubiprofen	Analgesic	69956-77-0	Y	$C_{16}H_{18}O_3$
Pemaglitazar	Antidiabetic	496050-39-6		$C_{18}H_{17}F_3O_3S$
Pemedolac	Analgesic	114716-16-4		$C_{22}H_{23}NO_3$
Pemerid Nitrate	Antitussive	50432-78-5		$C_{15}H_{32}N_2O$
Pemetrexed	Antineoplastic	137281-23-3		$C_{20}H_{21}N_5O_6$
Pemirolast	Antihistaminic	69372-19-6	Y	$C_{10}H_8N_6O$
Pemoline	Nootropic	2152-34-3	Y	$C_9H_8N_2O_2$
Pempidine	Antihypertensive	79-55-0		$C_{10}H_{21}N$
Penamecillin	Antibacterial	983-85-7		$C_{19}H_{22}N_2O_6S$
Penbutolol Sulfate	Antianginal	38363-40-5		$C_{18}H_{29}NO_2$
Penciclovir	Antiviral	39809-25-1		$C_{10}H_{15}N_5O_3$
Penethamate Hydriodide	Mydriatic	3689-73-4		$C_{22}H_{31}N_3O_4S$
Penfluridol	Antipsychotic	26864-56-2		$C_{28}H_{27}ClF_5NO$
Penflutizide	Diuretic	1766-91-2		$C_{13}H_{18}F_3N_3O_4S_2$
Pengitoxin	Cardiotonic	242-04-8		$C_{51}H_{74}O_{19}$
Penicillamine	Antirheumatic	52-67-5	Y	$C_5H_{11}NO_2S$
Penicillin V	Antibiotic	87-08-1		$C_{16}H_{18}N_2O_5S$
Penicillin X	Antibiotic	525-91-7		$C_{16}H_{18}N_2O_5S$
Penimocycline	Antibiotic	16259-34-0		$C_{39}H_{43}N_5O_{12}S$
Penirolol	Antianginal	58503-83-6		$C_{15}H_{22}N_2O_2$
Penoctonium Bromide	Antibacterial	17088-72-1		$C_{26}H_{50}BrNO_2$
Penprostene	Prostaglandin	61557-12-8		$C_{21}H_{32}O_5$
Pentabamate	Anxiolytic	5667-70-9		$C_8H_{16}N_2O_4$
Pentacynium Chloride	Unclassified	77-12-3		$C_{27}H_{39}N_3O.2Cl$
Pentaerythritol	Sedative	115-77-5		$C_5H_{12}O_4$
Pentaerythritol Tetranitrate	Vasodilator	78-11-5		$C_5H_8N_4O_{12}$
Pentafluranol	Steroid	65634-39-1		$C_{17}H_{15}F_5O_2$
Pentagestrone	Progestogen	7001-56-1		$C_{26}H_{38}O_3$
Pentalamide	Antifungal	5579-06-6		$C_{12}H_{17}NO_2$
Pentamethonium Bromide	Antihypertensive	541-20-8		$C_{11}H_{28}Br_2N_2$
Pentamidine	Antiprotozoal	100-33-4		$C_{19}H_{24}N_4O_2$
Pentamorphone	Analgesic	68616-83-1		$C_{22}H_{28}N_2O_3$
Pentamoxane	Sedative	4730-07-8		$C_{14}H_{21}NO_2$
Pentamustine	Antineoplastic	73105-03-0		$C_8H_{16}ClN_3O_2$
1-Pentanol	Dermatologic	71-41-0		$C_5H_{12}O$
Pentaphonate	Analgesic	24360-58-5		$C_{36}H_{40}Cl_5OP$
Pentapiperide	Antispasmodic	7009-54-3		$C_{18}H_{27}NO_2$
Pentapiperium Methylsulfate	Antispasmodic	7681-80-3		$C_{20}H_{33}NO_6S$
Pentaquine Phosphate	Antimalarial	86-78-2		$C_{18}H_{27}N_3O$
Pentazocine	Analgesic	359-83-1		$C_{19}H_{27}NO$
Penthienate	Mydriatic	22064-27-3		$C_{18}H_{30}NO_3S$
Penthrichloral	Sedative	5684-90-2		$C_7H_{11}Cl_3O_4$
Pentiapine Maleate	Antipsychotic	81382-51-6		$C_{15}H_{17}N_5S$
Pentifylline	Vasodilator	1028-33-7		$C_{13}H_{20}N_4O_2$
Pentigetide	Antihistaminic	62087-72-3		$C_{22}H_{36}N_8O_{11}$
Pentisomicin	Antibiotic	55870-64-9		$C_{19}H_{37}N_5O_7$
Pentisomide	Antiarrhythmic	78833-03-1		$C_{19}H_{33}N_3O$
Pentizidone Sodium	Antibacterial	55694-83-2		$C_8H_{12}N_2O_3$
Pentobarbital	Sedative	76-74-4		$C_{11}H_{18}N_2O_3$
Pentolinium	Antihypertensive	144-44-5		$C_{15}H_{32}N_2$
Pentomone	Androgen	67102-87-8		$C_{24}H_{26}O_5$
Pentopril	Antihypertensive	82924-03-6		$C_{18}H_{23}NO_5$

Table S1. *Cont.*

Common Name	Indication	CAS Number	Oral	Molecular Formula
Pentorex	Anorexic	434-43-5		$C_{11}H_{17}N$
Pentostatin	Antineoplastic	53910-25-1		$C_{11}H_{16}N_4O_4$
Pentoxifylline	Anticoagulant	6493-05-6		$C_{13}H_{18}N_4O_3$
Pentrinitrol	Vasodilator	1607-17-6		$C_5H_9N_3O_{10}$
Pentylenetetrazol	Nootropic	54-95-5		$C_6H_{10}N_4$
Pepstatin	Unclassified	26305-03-3		$C_{34}H_{63}N_5O_9$
Peradoxime	Analgesic	67254-81-3		$C_{22}H_{29}N_3O_4$
Perafensine	Antiparkinsonian	72444-62-3		$C_{19}H_{19}N_3$
Peralopride	Antiemetic	57083-89-3		$C_{20}H_{22}ClN_3O_4$
Peramivir	Antiviral	330600-85-6		$C_{15}H_{28}N_4O_4$
Peraquinsin	Antihypertensive	35265-50-0		$C_{23}H_{28}N_4O_4$
Perastine	Antineoplastic	4960-10-5		$C_{20}H_{25}NO$
Peratizole	Antiurolithic	29952-13-4		$C_{17}H_{26}N_4S_2$
Perazine	Antipsychotic	84-97-9		$C_{20}H_{25}N_3S$
Perbufylline	Bronchodilator	110390-84-6		$C_{23}H_{28}FN_5O_3$
Perflisobutane	Anesthetic	354-92-7		C_4F_{10}
Perfluamine	Cardiotonic	338-83-0		$C_9F_{21}N$
Perflubrodec	Cardiotonic	307-43-7		$C_{10}BrF_{21}$
Perfomedil	Vasodilator	92268-40-1		$C_{19}H_{29}NO_4$
Perfosfamide	Antineoplastic	62435-42-1		$C_7H_{15}Cl_2N_2O_4P$
Pergolide	Antiparkinsonian	66104-22-1	Y	$C_{19}H_{26}N_2S$
Perhexiline	Vasodilator	6621-47-2	Y	$C_{19}H_{35}N$
Periciazine	Antipsychotic	2622-26-6		$C_{21}H_{23}N_3OS$
Perifosine	Antineoplastic	157716-52-4		$C_{25}H_{52}NO_4P$
Perimetazine	Antipsychotic	13093-88-4		$C_{22}H_{28}N_2O_2S$
Perindopril	Antihypertensive	82834-16-0	Y	$C_{19}H_{32}N_2O_5$
Perindoprilat	Antihypertensive	95153-31-4		$C_{17}H_{28}N_2O_5$
Perisoxal	Antiinflammatory	2055-44-9		$C_{16}H_{20}N_2O_2$
Perlapine	Sedative	1977-11-3		$C_{19}H_{21}N_3$
Permethrin	Dermatologic	52645-53-1		$C_{21}H_{20}Cl_2O_3$
Perospirone	Antipsychotic	150915-41-6	Y	$C_{23}H_{30}N_4O_2S$
Perphenazine	Antipsychotic	58-39-9	Y	$C_{21}H_{26}ClN_3OS$
Persilic Acid	Unclassified	4444-23-9		$C_6H_6O_8S_2$
Perzinfotel	Analgesic	144912-63-0		$C_9H_{13}N_2O_5P$
Petrichloral	Sedative	78-12-6		$C_{13}H_{16}Cl_{12}O_8$
Pexantel	Anthelminithic	10001-13-5		$C_{12}H_{22}N_2O$
Phanquone	Antiamebic	84-12-8		$C_{12}H_6N_2O_2$
Phenacaine	Analgesic	101-93-9		$C_{18}H_{22}N_2O_2$
Phenacemide	Anticonvulsant	63-98-9		$C_9H_{10}N_2O_2$
Phenacetin	Analgesic	62-44-2		$C_{10}H_{13}NO_2$
Phenacridane Chloride	Antibiotic	3131-8-6		$C_{26}H_{28}NOCl$
Phenactropinium Chloride	Antihypertensive	3784-89-2		$C_{24}H_{28}ClNO_4$
Phenadoxone	Analgesic	467-84-5		$C_{23}H_{29}NO_2$
Phenaglycodol	Sedative	79-93-6		$C_{11}H_{15}ClO_2$
Phenamazoline	Antibiotic	501-62-2		$C_{10}H_{13}N_3$
Phenampromide	Analgesic	129-83-9		$C_{17}H_{26}N_2O$
Phenanthrene	Antineoplastic	85-01-8		$C_{14}H_{10}$
Phenaphthazine	Analgesic	1716-22-9		$C_{16}H_{10}N_4O_{11}S_2$
Phenarsone Sulfoxylate	Antibiotic	497-97-2		$C_7H_{10}AsNO_6S$
Phenazocine Hydrobromide	Analgesic	127-35-5		$C_{22}H_{27}NO$
Phenazopyridine	Analgesic	94-78-0	Y	$C_{11}H_{11}N_5$
Phenbutazone Sodium Glycerate	Antiinflammatory	798-21-0		$C_{19}H_{20}N_2O_2$
Phencarbamide	Antispasmodic	3735-90-8		$C_{19}H_{24}N_2OS$
Phencyclidine	Anesthetic	77-10-1		$C_{17}H_{25}N$
Phendimetrazine	Anorexic	634-03-7	Y	$C_{12}H_{17}NO$
Phenelzine	Antidepressant	51-71-8	Y	$C_8H_{12}N_2$
Phenethicillin Potassium	Antibiotic	147-55-7	Y	$C_{17}H_{20}N_2O_5S$
Pheneturide	Anticonvulsant	90-49-3		$C_{11}H_{14}N_2O_2$
Phenformin	Antidiabetic	114-86-3		$C_{10}H_{15}N_5$
Phenglutarimide	Antiparkinsonian	1156-05-4		$C_{17}H_{24}N_2O_2$
Phenicarbazide	Antiinflammatory	103-03-7		$C_7H_9N_3O$
Phenindamine	Antihistaminic	82-88-2		$C_{19}H_{19}N$
Phenindione	Anticoagulant	83-12-5		$C_{15}H_{10}O_2$

Table S1. *Cont.*

Common Name	Indication	CAS Number	Oral	Molecular Formula
Pheniprazine	Antihypertensive	55-52-7		$C_9H_{14}N_2$
Pheniramine	Antihistaminic	86-21-5		$C_{16}H_{20}N_2$
Phenisonone Hydrobromide	Respiratory Stimulant	715646-49-4		$C_{12}H_{17}NO_3$
Phenmetrazine	Anorexic	134-49-6		$C_{11}H_{15}NO$
Phenobarbital	Anticonvulsant	50-06-6	Y	$C_{12}H_{12}N_2O_3$
Phenol Camphorated	Dermatologic	108-95-2		C_6H_6O
Phenolphthalein	Laxative	77-09-8		$C_{20}H_{14}O_4$
Phenomorphan	Analgesic	468-07-5		$C_{24}H_{29}NO$
Phenoperidine	Analgesic	562-26-5		$C_{23}H_{29}NO_3$
Phenothiazine	Anthelminithic	92-84-2		$C_{12}H_9NS$
Phenothrin	Dermatologic	26002-80-2		$C_{23}H_{26}O_3$
Phenoxyacetic Acid	Dermatologic	122-59-8		$C_8H_8O_3$
Phenoxybenzamine	Antihypertensive	59-96-1	Y	$C_{18}H_{22}ClNO$
Phenoxypropazine	Antidepressant	3818-37-9		$C_9H_{14}N_2O$
Phenprobamate	Muscle Relaxant	673-31-4		$C_{10}H_{13}NO_2$
Phenprocoumon	Anticoagulant	435-97-2	Y	$C_{18}H_{16}O_3$
Phenpromethamine	Adrenergic	93-88-9		$C_{10}H_{15}N$
Phensuximide	Anticonvulsant	86-34-0		$C_{11}H_{11}NO_2$
Phentermine	Anorexic	122-09-8	Y	$C_{10}H_{15}N$
Phentolamine	Antihypertensive	50-60-2		$C_{17}H_{19}N_3O$
Phenyl Aminosalicylate	Antibacterial	133-11-9		$C_{13}H_{11}NO_3$
Phenyl Salicylate	Analgesic	118-55-8		$C_{13}H_{10}O_3$
Phenylbutazone	Antiinflammatory	50-33-9		$C_{19}H_{20}N_2O_2$
Phenylephrine	Mydriatic	59-42-7		$C_9H_{13}NO_2$
Phenylpropanolamine	Decongestant	14838-15-4		$C_9H_{13}NO$
Phenylthilone	Laxative	115-55-9		$C_{12}H_{13}NO_2S$
Phenylthioacetic Acid	Antispasmodic	949-01-9		$C_{12}H_{15}NOS$
Phenyltoloxamine	Antihistaminic	92-12-6		$C_{17}H_{21}NO$
Phenyracillin	Antibiotic	7463-81-2		$C_{16}H_{18}N_2$
Phenyramidol	Analgesic	553-69-5		$C_{13}H_{14}N_2O$
Phenytoin	Anticonvulsant	57-41-0	Y	$C_{15}H_{12}N_2O_2$
Phetharbital	Sedative	357-67-5		$C_{14}H_{16}N_2O_3$
Phethenylate Sodium	Anticonvulsant	7772-37-4		$C_{13}H_{10}N_2O_2S$
Pholcodine	Antitussive	509-67-1		$C_{23}H_{30}N_2O_4$
Pholedrine	Antihypotensive	370-14-9		$C_{10}H_{15}NO$
Phthalofyne	Anthelminithic	131-67-9		$C_{14}H_{14}O_4$
Phthalylsulfacetamide	Antibiotic	131-69-1		$C_{16}H_{14}N_2O_6S$
Phthalylsulfamethizole	Antibiotic	485-24-5		$C_{17}H_{14}N_4O_5S_2$
Phthalylsulfathiazole	Antibiotic	85-73-4		$C_{17}H_{13}N_3O_5S_2$
Physostigmine	Cholinergic	57-47-6		$C_{15}H_{21}N_3O_2$
Piberaline	Antidepressant	39640-15-8		$C_{17}H_{19}N_3O$
Piboserod	Gastroprokinetic	152811-62-6		$C_{22}H_{31}N_3O_2$
Pibrozelesin	Antineoplastic	154889-68-6		$C_{32}H_{40}BrN_5O8$
Pibutidine	Antiulcerative	103922-33-4		$C_{19}H_{24}N_4O_3$
Picafibrate	Antihyperlipidemic	57548-79-5		$C_{18}H_{19}ClN_2O_4$
Picartamide	Antimigraine	76732-75-7		$C_{11}H_{14}N_2S_2$
Picenadol	Analgesic	79201-85-7		$C_{16}H_{25}NO$
Picilorex	Anorexic	62510-56-9		$C_{14}H_{18}ClN$
Piclamilast	Antiinflammatory	144035-83-6		$C_{18}H_{18}Cl_2N_2O_3$
Piclonidine	Antihypertensive	72467-44-8		$C_{14}H_{17}Cl_2N_3O$
Piclopastine	Antineoplastic	55837-13-3		$C_{20}H_{26}ClN_3O_2$
Picloxydine	Antibacterial	5636-92-0		$C_{20}H_{24}Cl_2N_{10}$
Piclozotan	Nootropic	182415-09-4		$C_{23}H_{24}ClN_3O_2$
Picobenzide	Antipsychotic	51832-87-2		$C_{15}H_{16}N_2O$
Picodralazine	Diuretic	17692-43-2		$C_{14}H_{13}N_5$
Picolamine	Dermatologic	3731-52-0		$C_6H_8N_2$
Piconol	Decongestant	586-98-1		C_6H_7NO
Picoperine	Antitussive	21755-66-8		$C_{19}H_{25}N_3$
Picoprazole	Antiulcerative	78090-11-6		$C_{17}H_{17}N_3O_3S$
Picotamide	Antithrombotic	32828-81-2		$C_{21}H_{20}N_4O_3$
Picotrin Diolamine	Hepatic Protectant	64063-57-6		$C_{25}H_{19}NO_2$
Picric Acid	Dermatologic	88-89-1		$C_6H_3N_3O_7$
Picrotoxin	Nootropic	21416-53-5		$C_{15}H_{18}O_7$

Table S1. *Cont.*

Common Name	Indication	CAS Number	Oral	Molecular Formula
Picrotoxin	Nootropic	17617-45-7		$C_{15}H_{16}O_6$
Picumast	Antihistaminic	39577-19-0		$C_{25}H_{29}ClN_2O_3$
Picumeterol Fumarate	Bronchodilator	130641-36-0		$C_{21}H_{29}Cl_2N_3O_2$
Pidobenzone	Dermatologic	138506-45-3		$C_{11}H_{11}NO_4$
Pidolacetamol	Analgesic	114485-92-6		$C_{13}H_{14}N_2O_4$
Pidolic Acid	Unclassified	98-79-3		$C_5H_7NO_3$
Pidotimod	Immunomodulator	121808-62-6		$C_9H_{12}N_2O_4S$
Pifarnine	Antiulcerative	56208-01-6		$C_{27}H_{40}N_2O_2$
Pifenate	Uricosuric	15686-87-0		$C_{22}H_{27}NO_2$
Pifexole	Antiparkinsonian	27199-40-2		$C_{13}H_8ClN_3O$
Piflutixol	Antipsychotic	54341-02-5		$C_{24}H_{25}F_4NOS$
Pifoxime	Analgesic	31224-92-7		$C_{15}H_{20}N_2O_3$
Piketoprofen	Antiinflammatory	60576-13-8		$C_{22}H_{20}N_2O_2$
Pildralazine	Vasodilator	64000-73-3		$C_8H_{15}N_5O$
Pilocarpine	Antiglaucoma	92-13-7	Y	$C_{11}H_{16}N_2O_2$
Pilsicainide	Antiarrhythmic	88069-67-4	Y	$C_{17}H_{24}N_2O$
Pimagedine	Antidiabetic	79-17-4		CH_6N_4
Pimeclone	Respiratory Stimulant	534-84-9		$C_{12}H_{21}NO$
Pimecrolimus	Immunosuppressant	137071-32-0		$C_{43}H_{68}ClNO_{11}$
Pimefylline	Vasodilator	10001-43-1		$C_{15}H_{18}N_6O_2$
Pimelautide	Immunomodulator	78512-63-7		$C_{29}H_{52}N_6O_9$
Pimetacin	Analgesic	79992-71-5		$C_{25}H_{21}ClN_2O_3S$
Pimethixene	Antipsychotic	314-03-4		$C_{19}H_{19}NS$
Pimetine	Antihyperlipidemic	3565-03-5		$C_{16}H_{26}N_2$
Pimetremide	Anticonvulsant	578-89-2		$C_{16}H_{18}N_2O_2$
Pimilprost	Prostaglandin	139403-31-9		$C_{23}H_{40}O_5$
Piminodine Esylate	Analgesic	13495-09-5		$C_{23}H_{30}N_2O_2$
Pimobendan	Cardiotonic	74150-27-9	Y	$C_{19}H_{18}N_4O_2$
Pimonidazole	Antiprotozoal	70132-50-2		$C_{11}H_{18}N_4O_3$
Pimozide	Antipsychotic	2062-78-4	Y	$C_{28}H_{29}F_2N_3O$
Pinacidil	Antihypertensive	60560-33-0		$C_{13}H_{19}N_5$
Pinadoline	Analgesic	38955-22-5		$C_{19}H_{19}Cl_2N_3O_3$
Pinafide	Antineoplastic	54824-20-3		$C_{18}H_{17}N_3O_4$
Pinaverium Bromide	Antispasmodic	53251-94-8	Y	$C_{26}H_{41}Br_2NO_4$
Pinazepam	Anxiolytic	52463-83-9		$C_{18}H_{13}ClN_2O$
Pincainide	Antiarrhythmic	83471-41-4		$C_{16}H_{24}N_2O$
Pindolol	Antihypertensive	13523-86-9	Y	$C_{14}H_{20}N_2O_2$
Pinokalant	Antiarrhythmic	149759-26-2		$C_{41}H_{48}N_2O_9$
Pinolcaine	Anesthetic	28240-18-8		$C_{23}H_{29}NO_2$
Pinoxepin	Antipsychotic	14008-66-3		$C_{23}H_{27}ClN_2O_2$
Pioglitazone	Antidiabetic	111025-46-8	Y	$C_{19}H_{20}N_2O_3S$
Pipacycline	Antibiotic	1110-80-1		$C_{29}H_{38}N_4O_9$
Pipamazine	Antiemetic	84-04-8		$C_{21}H_{24}ClN_3OS$
Pipamperone	Antipsychotic	1893-33-0		$C_{21}H_{30}FN_3O_2$
Pipazethate	Antitussive	2167-85-3		$C_{21}H_{25}N_3O_3S$
Pipebuzone	Antiinflammatory	27315-91-9		$C_{25}H_{32}N_4O_2$
Pipecuronium Bromide	Muscle Relaxant	52212-02-9		$C_{35}H_{62}Br_2N_4O_4$
Pipemidic Acid	Antibiotic	51940-44-4		$C_{14}H_{17}N_5O_3$
Pipendoxifene	Progestogen	198480-55-6		$C_{29}H_{32}N_2O_3$
Pipenzolate Bromide	Antispasmodic	125-51-9		$C_{22}H_{28}BrNO_3$
Pipequaline	Anxiolytic	77472-98-1		$C_{22}H_{24}N_2$
Piperacetazine	Antipsychotic	3819-00-9		$C_{24}H_{30}N_2O_2S$
Piperacillin	Antibiotic	61477-96-1		$C_{23}H_{27}N_5O_7S$
Piperamide Maleate	Anthelminithic	299-48-9		$C_{17}H_{28}N_4O$
Piperazine	Anthelminithic	110-85-0		$C_4H_{10}N_2$
Piperidine Phosphate	Unclassified	110-89-4		$C_5H_{11}N$
Piperidione	Antitussive	77-03-2		$C_9H_{15}NO_2$
Piperidolate	Antispasmodic	82-98-4		$C_{21}H_{25}NO_2$
Piperilate	Antispasmodic	4546-39-8		$C_{21}H_{25}NO_3$
Piperine	Antibacterial	94-62-2		$C_{17}H_{19}NO_3$
Piperocaine	Anesthetic	136-82-3		$C_{16}H_{23}NO_2$
Piperonyl Butoxide	Anthelminthic	51-03-6		$C_{19}H_{30}O_5$
Piperoxan	Antihypertensive	59-39-2		$C_{14}H_{19}NO_2$

Table S1. *Cont.*

Common Name	Indication	CAS Number	Oral	Molecular Formula
Piperphenidol	Antiemetic	90-23-3		$C_{18}H_{29}NO$
Piperylone	Analgesic	2531-04-6		$C_{17}H_{23}N_3O$
Pipobroman	Antineoplastic	54-91-1		$C_{10}H_{16}Br_2N_2O_2$
Pipoctanone	Bone Resorption Inhibitor	18841-58-2		$C_{22}H_{35}NO$
Pipofezine	Unclassified	24886-52-0		$C_{16}H_{19}N_5O$
Piposulfan	Antineoplastic	2608-24-4		$C_{12}H_{22}N_2O_8S_2$
Pipotiazine Palmitate	Antipsychotic	37517-26-3		$C_{40}H_{63}N_3O_4S_2$
Pipoxizine	Sedative	55837-21-3		$C_{24}H_{31}NO_3$
Pipoxolan	Muscle Relaxant	23744-24-3		$C_{22}H_{25}NO_3$
Pipradimadol	Analgesic	68797-29-5		$C_{24}H_{37}ClN_2O_2$
Pipradrol	Nootropic	467-60-7		$C_{18}H_{21}NO$
Pipramadol	Analgesic	55313-67-2		$C_{23}H_{35}ClN_2O_2$
Pipratecol	Antiulcerative	15534-05-1		$C_{19}H_{24}N_2O_4$
Piprocurarium Iodide	Neuromuscular Blocking Agent	3562-55-8		$C_{23}H_{40}I_2N_2O_3$
Piprofurol	Antiarrhythmic	40680-87-3		$C_{26}H_{33}NO_6$
Piprozolin	Choleretic	17243-64-0		$C_{14}H_{22}N_2O_3S$
Piquindone	Antipsychotic	78541-97-6		$C_{15}H_{22}N_2O$
Piquizil	Bronchodilator	21560-58-7		$C_{19}H_{26}N_4O_4$
Piracetam	Nootropic	7491-74-9	Y	$C_6H_{10}N_2O_2$
Pirandamine	Antidepressant	42408-79-7		$C_{17}H_{23}NO$
Pirarubicin	Antineoplastic	72496-41-4		$C_{32}H_{37}NO_{12}$
Piraxelate	Unclassified	82209-39-0		$C_{15}H_{25}NO_3$
Pirazmonam Sodium	Antibiotic	108319-07-9		$C_{22}H_{24}N_{10}O_{12}S_2$
Pirazolac	Antirheumatic	71002-09-0		$C_{17}H_{12}ClFN_2O_2$
Pirbenicillin Sodium	Antibacterial	55975-92-3		$C_{24}H_{26}N_6O_5S$
Pirbuterol	Bronchodilator	38677-81-5		$C_{12}H_{20}N_2O_3$
Pirdonium Bromide	Antihistaminic	35620-67-8		$C_{22}H_{30}NOBr$
Pirenoxine	Opthalmic	1043-21-6		$C_{16}H_8N_2O_5$
Pirenperone	Antidote	75444-65-4		$C_{23}H_{24}FN_3O_2$
Pirenzepine	Antiulcerative	28797-61-7	Y	$C_{19}H_{21N5O2}$
Pirepolol	Antihypertensive	69479-26-1		$C_{21}H_{32}N_4O_5$
Piretanide	Diuretic	55837-27-9	Y	$C_{17}H_{18}N_2O_5S$
Pirfenidone	Analgesic	53179-13-8	Y	$C_{12}H_{11}NO$
Piribedil	Vasodilator	3605-01-4	Y	$C_{16}H_{18}N_4O_2$
Piridicillin Sodium	Antibacterial	69414-41-1		$C_{32}H_{35}N_5O_{11}S_2Na$
Piridocaine	Analgesic	87-21-8		$C_{14}H_{20}N_2O_2$
Piridoxilate	Nootropic	46794-57-4		$C_{10}H_{13}NO_6$
Piridoxilate	Nootropic	17692-44-3		$C_{10}H_{13}NO_6$
Piridronic Acid	Bone Resorption Inhibitor	75755-07-6		$C_7H_{11}NO_6P_2$
Pirifibrate	Antihyperlipidemic	55285-45-5		$C_{17}H_{18}ClNO_4$
Pirinidazole	Antiprotozoal	55432-15-0		$C_{10}H_{10}N_4O_2S$
Pirinixic Acid	Antihyperlipidemic	50892-23-4		$C_{14}H_{14}ClN_3O_2S$
Pirinixil	Antihyperlipidemic	65089-17-0		$C_{16}H_{19}ClN_4O_2S$
Piriprost	Bronchodilator	79672-88-1		$C_{26}H_{35}NO_4$
Piriqualone	Muscle Relaxant	1897-89-8		$C_{22}H_{17}N_3O$
Pirisudanol	Antidepressant	33605-94-6		$C_{16H24N2O6}$
Piritramide	Analgesic	302-41-0		$C_{27}H_{34}N_4O$
Piritrexim Isethionate	Antineoplastic	72732-56-0		$C_{17}H_{19}N_5O_2$
Pirlimycin	Antibacterial	79548-73-5		$C_{17}H_{31ClN2O5S}$
Pirlindole	Antidepressant	60762-57-4		$C_{15}H_{18}N_2$
Pirmagrel	Antithrombotic	85691-74-3		$C_{13}H_{16}N_2O_2$
Pirmenol	Antiarrhythmic	68252-19-7		$C_{22}H_{30}N_2O$
Pirnabine	Antiglaucoma	19825-63-9	Y	$C_{19}H_{24}O_3$
Piroctone	Dermatologic	50650-76-5		$C_{14}H_{23}NO_2$
Pirodavir	Antiviral	124436-59-5		$C_{21}H_{27}N_3O_3$
Pirodomast	Antihistamine	108310-20-9		$C_{18}H_{17}N_3O_2$
Pirogliride Tartrate	Antidiabetic	62625-18-7		$C_{16}H_{22}N_4$
Piroheptine	Antiparkinsonian	16378-21-5		$C_{22}H_{25}N$
Pirolate	Bronchodilator	55149-05-8		$C_{16}H_{15}N_3O_5$
Pirolazamide	Antiarrhythmic	39186-49-7		$C_{23}H_{29}N_3O$
Piromidic Acid	Antibacterial	19562-30-2		$C_{14}H_{16}N_4O_3$
Piroxantrone	Antineoplastic	91441-23-5		$C_{21}H_{25}N_5O_4$

Table S1. *Cont.*

Common Name	Indication	CAS Number	Oral	Molecular Formula
Piroxicam	Antiinflammatory	36322-90-4	Y	$C_{15}H_{13}N_3O_4S$
Piroxicam Cinnamate	Antiinflammatory	87234-24-0		$C_{24}H_{19}N_3O_5S$
Piroxicillin	Antibiotic	82509-56-6		$C_{27}H_{28}N_8O_9S_2$
Piroximone	Cardiotonic	84490-12-0		$C_{11}H_{11}N_3O_2$
Pirozadil	Antihyperlipidemic	54110-25-7		$C_{27}H_{29}NO_{10}$
Pirprofen	Antiinflammatory	31793-07-4		$C_{13}H_{14}ClNO_2$
Pirquinozol	Antihistaminic	65950-99-4		$C_{11}H_9N_3O_2$
Pirsidomine	Vasodilator	132722-74-8		$C_{17}H_{22}N_4O_3$
Pirtenidine	Antibacterial	103923-27-9		$C_{21}H_{38}N_2$
Pitavastatin	Antihyperlipidemic	147511-69-1	Y	$C_{25}H_{24}FNO_4$
Pitenodil	Cardiotonic	59840-71-0		$C_{17}H_{27}N_3O_3S$
Pitofenone	Antispasmodic	54063-52-4		$C_{22}H_{25}NO_4$
Pituxate	Antiulcerative	39123-11-0		$C_{23}H_{27}NO_2$
Pivagabine	Anticonvulsant	69542-93-4		$C_9H_{17}NO_3$
Pivampicillin	Antibacterial	33817-20-8		$C_{22}H_{29}N_3O_6S$
Pivenfrine	Unclassified	71206-88-7		$C_{14}H_{21}NO_3$
Pivhydrazine	Antidepressant	306-19-4		$C_{12}H_{18}N_2O$
Pivopril	Antihypertensive	81045-50-3		$C_{16}H_{27}NO_4S$
Pivoxazepam	Anxiolytic	55299-10-0		C_{20}H19ClN2O3
Pixantrone	Antineoplastic	144510-96-3		$C_{17}H_{19}N_5O_2$
Pizotyline	Antidepressant	15574-96-6		$C_{19}H_{21}NS$
Plafibride	Antithrombotic	63394-05-8		$C_{16}H_{22}ClN_3O_4$
Platonin	Immunomodulator	6009-02-5		$C_{38}H_{61}N_3S_3$.2Cl
Plaunotol	Antiulcerative	64218-02-6	Y	$C_{20}H_{34}O_2$
Pleconaril	Antiviral	153168-05-9		$C_{18}H_{18}F_3N_3O_3$
Plerixafor	Antineoplastic	110078-46-1		$C_{28}H_{54}N_8$
Pleuromulin	Antibiotic	125-65-5		$C_{22}H_{34}O_5$
Plevitrexed	Antineoplastic	153537-73-6		$C_{26}H_{25}FN_8O_4$
Plomestane	Antineoplastic	77016-85-4		$C_{21}H_{26}O_2$
P Nitrophenyl O Ethyl Ethylphosphonate	Unclassified	546-71-4		$C_{10}H_{14}NO_5P$
Pobilukast Edamine	Bronchodilator	107023-41-6		$C_{26}H_{34}O_5S$
Podilfen	Unclassified	13409-53-5		$C_{18}H_{23}N_3O_2S$
Podofilox	Antiviral	518-28-5		$C_{22}H_{22}O_8$
Polaprezinc	Antiulcerative	107667-60-7	Y	$C_9H_{12}N_4O_3Zn$
Poldine	Mydriatic	596-50-9		$C_{21}H_{26}NO_3$
Polythiazide	Diuretic	346-18-9	Y	$C_{11}H_{13}ClF_3N_3O_4S_3$
Pomisartan	Antihypertensive	144702-17-0		$C_{31}H_{30}N_4O_2$
Ponalrestat	Antidiabetic	72702-95-5		$C_{17}H_{12}BrFN_2O_3$
Ponazuril	Antibiotic	69004-04-2		$C_{18}H_{14}F_3N_3O_6S$
Ponfibrate	Antihyperlipidemic	53341-49-4		$C_{18}H_{16}Cl_2O_4$
Porfiromycin	Antibacterial	801-52-5		$C_{16}H_{20}N_4O_5$
Posaconazole	Antifungal	171228-49-2	Y	$C_{37}H_{42}F_2N_8O_4$
Posatirelin	Pituitary	78664-73-0		$C_{17}H_{28}N_4O_4$
Posizolid	Antibiotic	252260-02-9	Y	$C_{21}H_{21}F_2N_3O_7$
Poskine	Anxiolytic	585-14-8		$C_{20}H_{25}NO_5$
Nitrazepate	Anxiolytic	60676-80-4		$C_{16}H_{11}N_3O_5$
Thiocyanate	Unclassified	302-04-5		CNS
Practolol	Antiarrhythmic	6673-35-4		$C_{14}H_{22}N_2O_3$
Pradefovir Mesylate	Antiviral	625095-60-5	Y	$C_{17}H_{19}ClN_5O_4P$
Pradofloxacin	Antibiotic	195532-12-8		C_{21}H21FN4O3
Prajmalium Bitartrate	Antiarrhythmic	2589-47-1	Y	$C_{27}H_{38}N_2O_8$
Pralatrexate	Antineoplastic	146464-95-1		$C_{23}H_{23}N_7O_5$
Pralidoxime	Antidote	6735-59-7		$C_7H_9N_2O$
Pralmorelin Dihydrochloride	Pituitary	158861-67-7		$C_{45}H_{55}N_9O_6$
Pralnacasan	Antirheumatic	192755-52-5		$C_{26}H_{29}N_5O_7$
Pramipexole	Antiparkinsonian	104632-26-0	Y	$C_{10}H_{17}N_3S$
Pramiracetam	Nootropic	68497-62-1	Y	$C_{14}H_{27}$N3O2
Pramiverine	Antispasmodic	14334-40-8		$C_{21}H_{27}N$
Pramoxine	Anesthetic	140-65-8		$C_{17}H_{27}NO_3$
Prampine	Unclassified	7009-65-6		$C_{20}H_{27}NO_4$
Pranazepide	Antispasmodic	150408-73-4		$C_{26}H_{19}FN_4O_2$
Pranidipine	Antihypertensive	99522-79-9		$C_{25}H_{24}N_2O_6$

Table S1. *Cont.*

Common Name	Indication	CAS Number	Oral	Molecular Formula
Pranlukast	Bronchodilator	103177-37-3	Y	$C_{27}H_{23}N_5O_4$
Pranolium Chloride	Antiarrhythmic	42879-47-0		$C_{18}H_{26}ClNO_2$
Pranoprofen	Antiinflammatory	52549-17-4		$C_{15}H_{13}NO_3$
Pranosal	Unclassified	17716-89-1		$C_{16}H_{23}NO_3$
Prasterone	Steroid	53-43-0	Y	$C_{19}H_{28}O_2$
Prasugrel	Anticoagulant	150322-43-3	Y	$C_{20}H_{20}FNO_3S$
Pratosartan	Antihypertensive	153804-05-8	Y	$C_{25}H_{26}N_6O$
Pravadoline	Analgesic	92623-83-1		$C_{23}H_{26}N_2O_3$
Pravastatin Sodium	Antihyperlipidemic	81093-37-0	Y	$C_{23}H_{36}O_7$
Praxadine	Antibiotic	4023-00-1		$C_4H_6N_4$
Prazepam	Sedative	2955-38-6	Y	C_{19}H17ClN2O
Prazepine	Anticonvulsant	50-49-7		$C_{19}H_{24}N_2$
Praziquantel	Anthelminithic	55268-74-1	Y	$C_{19}H_{24}N_2O_2$
Prazitone	Antipsychotic	2409-26-9		$C_{16}H_{19}N_3O_3$
Prazocillin	Antibiotic	15949-72-1		$C_{19}H_{18}Cl_2N_4O_4S$
Prazosin	Antihypertensive	19216-56-9	Y	$C_{19}H_{21}N_5O_4$
Preclamol	Antipsychotic	85966-89-8		$C_{14}H_{21}NO$
Prednazoline	Antiinflammatory	4846-91-7		$C_{13}H_{18}N_2O$
Prednicarbate	Glucocorticoid	73771-04-7		$C_{27}H_{36}O_8$
Prednimustine	Antineoplastic	29069-24-7		$C_{35}H_{45}Cl_2NO_6$
Prednisolamate	Glucocorticoid	5626-34-6		$C_{27}H_{39}NO_6$
Prednisolone	Glucocorticoid	50-24-8	Y	$C_{21}H_{28}O_5$
Prednisolone Acetate	Glucocorticoid	52-21-1	Y	C23H30O6
Prednisolone Hemisuccinate	Glucocorticoid	2920-86-7		$C_{25}H_{32}O_8$
Prednisolone Pivalate	Glucocorticoid	1107-99-9		$C_{26}H_{36}O_6$
Prednisolone Sodium Phosphate	Glucocorticoid	302-25-0		$C_{21}H_{29}O_8P$
Prednisolone Steaglate	Glucocorticoid	5060-55-9		$C_{41}H_{64}O_8$
Prednisolone Tebutate	Glucocorticoid	7681-14-3		$C_{27}H_{38}O_6$
Prednisolone Valerate Acetate	Glucocorticoid	72064-79-0		$C_{28}H_{38}O_7$
Prednisone	Glucocorticoid	53-03-2	Y	$C_{21}H_{26}O_5$
Prednival	Glucocorticoid	15180-00-4		$C_{26}H_{36}O_6$
Prednylidene	Glucocorticoid	599-33-7		$C_{22}H_{28}O_5$
Prefenamate	Antiinflammatory	57775-28-7		$C_{19}H_{18}F_3NO_2$
Pregabalin	Anticonvulsant	148553-50-8	Y	$C_8H_{17}NO_2$
Pregnandiol	Progestogen	25908-35-4		$C_{21}H_{36}O_2$
Pregnenolone	Progestogen	145-13-1		$C_{21}H_{32}O_2$
Pregnenolone Acetate	Progestogen	1778-02-5		$C_{23}H_{34}O_3$
Pregnenolone Succinate	Progestogen	4598-67-8		$C_{25}H_{36}O_5$
Premafloxacin	Antibacterial	143383-65-7		$C_{21}H_{26}FN_3O_4$
Premazepam	Anxiolytic	57435-86-6		$C_{15}H_{15}N_3O$
Prenalterol	Adrenergic	57526-81-5		$C_{12}H_{19}NO_3$
Prenderol	Unclassified	115-76-4		$C_7H_{16}O_2$
Prenisteine	Antitussive	5287-46-7		$C_8H_{15}NO_2S$
Prenoverine	Mucolytic	66022-25-1		$C_{25}H_{29}NO_2$
Prenoxdiazine	Antitussive	47543-65-7		$C_{23}H_{27}N_3O$
Prenylamine	Vasodilator	390-64-7		$C_{24}H_{27}N$
Pretamazium Iodide	Unclassified	24840-59-3		$C_{29}H_{29}N_2SI$
Pretiadil	Antianginal	30840-27-8		$C_{26}H_{31}N_3O_2S$
Pribecaine	Anesthetic	55837-22-4		$C_{16}H_{23}NO_3$
Pridefine	Antidepressant	5370-41-2		$C_{19}H_{21}N$
Prideperone	Antipsychotic	95374-52-0		$C_{23}H_{24}FN_3O_3$
Pridinol	Antiparkinsonian	511-45-5		$C_{20}H_{25}NO$
Prifelone	Antiinflammatory	69425-13-4		$C_{19}H_{24}O_2S$
Prifinium Bromide	Antispasmodic	4630-95-9		$C_{22}H_{28}BrN$
Prifuroline	Antipsychotic	70833-07-7		$C_{14}H_{16}N_2O$
Prilocaine	Anesthetic	721-50-6		$C_{13}H_{20}N_2O$
Primaperone	Antipsychotic	1219-35-8		$C_{15}H_{20}FNO$
Primaquine	Antimalarial	90-34-6	Y	$C_{15}H_{21}N_3O$
Primidolol	Antihypertensive	67227-55-8		$C_{17}H_{23}N_3O_4$
Prinomastat	Antineoplastic	192329-42-3		$C_{18}H_{21}N_3O_5S_2$
Prinomide Tromethamine	Antirheumatic	77639-66-8		$C_{15H13N3O2}$
Prinoxodan	Cardiotonic	111786-07-3		$C_{13}H_{14}N_4O_2$
Prisotinol	Nootropic	76906-79-1		$C_{11}H_{18}N_{2O}$

Table S1. *Cont.*

Common Name	Indication	CAS Number	Oral	Molecular Formula
Prizidilol	Antihypertensive	59010-44-5		$C_{17}H_{25}N_5O_2$
Proadifen	Antidiabetic	302-33-0		$C_{23}H_{31}NO_2$
Probarbital Sodium	Sedative	76-76-6		$C_9H_{14}N_2O_3$
Probenecid	Uricosuric	57-66-9	Y	$C_{13}H_{19}NO_4S$
Probicromil Calcium	Antihistaminic	58805-38-2		$C_{17}H_{12}O_8$
Probucol	Antihyperlipidemic	23288-49-5		$C_{31}H_{48}O_2S_2$
Procainamide	Antiarrhythmic	51-06-9	Y	$C_{13}H_{21}N_3O$
Procaine	Anesthetic	59-46-1	Y	$C_{13}H_{20}N_2O_2$
Procarbazine	Antineoplastic	671-16-9	Y	$C_{12}H_{19}N_3O$
Procaterol	Bronchodilator	72332-33-3		$C_{16}H_{22}N_2O_3$
Prochlorperazine	Antiemetic	58-38-8	Y	$C_{20}H_{24}ClN_3S$
Procinolol	Antihypertensive	27325-36-6		$C_{15}H_{23}NO_2$
Procinonide	Steroid	58497-00-0		$C_{27}H_{34}F_2O_7$
Proclonol	Anthelminithic	14088-71-2		$C_{16}H_{14}Cl_2O$
Procodazole	Immunomodulator	23249-97-0		$C_{10}H_{10}N_2O_2$
Procyclidine	Antiparkinsonian	77-37-2	Y	$C_{19}H_{29}NO$
Procymate	Anxiolytic	13931-64-1		C_{10}H19NO2
Prodeconium Bromide	Antifungal	3690-61-7		$C_{28}H_{58}Br_2N_2O_6$
Prodilidine	Analgesic	3734-17-6		$C_{15}H_{21}NO_2$
Prodipine	Antiparkinsonian	31314-38-2		$C_{20}H_{25}N$
Prodolic Acid	Antiinflammatory	36505-82-5		$C_{16}H_{19}NO_3$
Profadol	Analgesic	428-37-5		$C_{14}H_{21}NO$
Profexalone	Sedative	34740-13-1		$C_{13}H_{16}N_2O_3$
Proflazepam	Anxiolytic	52829-30-8		$C_{18}H_{16}ClFN_2O_3$
Progabide	Anticonvulsant	62666-20-0		$C_{17}H_{16}ClFN_2O_2$
Progesterone	Progestogen	57-83-0	Y	$C_{21}H_{30}O_2$
Proglumetacin	Antiinflammatory	57132-53-3	Y	$C_{46}H_{58}ClN_5O_8$
Proglumide	Antiulcerative	6620-60-6	Y	$C_{18}H_{26}N_2O_4$
Proheptazine	Analgesic	77-14-5		$C_{17}H_{25}NO_2$
Proligestone	Progestogen	23873-85-0		$C_{24}H_{34}O_4$
Prolintane	Antidepressant	493-92-5		$C_{15}H_{23}N$
Promazine	Antipsychotic	58-40-2		$C_{17}H_{20}N_2S$
Promegestone	Progestogen	34184-77-5	Y	$C_{22}H_{30}O_2$
Promestriene	Dermatologic	39219-28-8		$C_{22}H_{32}O_2$
Promethazine	Antihistaminic	60-87-7	Y	$C_{17}H_{20}N_2S$
Promethestrol Dipropionate	Estrogen	84-13-9		$C_{26}H_{34}O_4$
Promolate	Antitussive	3615-74-5		$C_{16}H_{23}NO_4$
Promoxolane	Unclassified	470-43-9		$C_{10}H_{20}O_3$
Pronetalol	Antianginal	54-80-8		$C_{15}H_{19}NO$
Propacetamol	Analgesic	66532-85-2		$C_{14}H_{20}N_2O_3$
Propafenone	Antiarrhythmic	54063-53-5	Y	$C_{21}H_{27}NO_3$
Propamidine	Antiprotozoal	104-32-5		$C_{17}H_{20}N_4O_2$
Propanidid	Anesthetic	1421-14-3		$C_{18}H_{27}NO_5$
Propanocaine	Anesthetic	493-76-5		$C_{20}H_{25}NO_2$
Propantheline	Antispasmodic	298-50-0	Y	$C_{23}H_{30}NO_3$
Proparacaine	Anesthetic	499-67-2		$C_{16}H_{26}N_2O_3$
Propatyl Nitrate	Vasodilator	2921-92-8		$C_6H_{11}N_3O_9$
Propentofylline	Nootropic	55242-55-2		$C_{15}H_{22}N_4O_3$
Propenzolate	Mydriatic	4354-45-4		$C_{25}H_{29}NO_3$
Properidine	Analgesic	561-76-2		$C_{16}H_{23}NO_2$
Propetamide	Antidiabetic	730-07-4		$C_{14}H_{22}N_2O_2$
Propetandrol	Steroid	3638-82-2		$C_{23}H_{36}O_3$
Propicillin	Antibiotic	551-27-9		$C_{18}H_{22}N_2$O5S
Propikacin	Antibacterial	66887-96-5		$C_{21}H_{43}N_5O_{12}$
Propinetidine	Antiulcerative	3811-53-8		$C_{19}H_{25}NO_2$
Propiomazine	Sedative	362-29-8		$C_{20}H_{24}N_2OS$
Propionic Acid	Antifungal	79-09-4		$C_3H_6O_2$
Propipocaine	Anesthetic	3670-68-6		$C_{17}H_{25}NO_2$
Propiram Fumarate	Analgesic	15686-91-6		$C_{16}H_{25}N_3O$
Propisergide	Antimigraine	5793-04-4		$C_{20}H_{25}N_3O_2$
Propiverine	Antispasmodic	60569-19-9	Y	$C_{23}H_{29}NO_3$
Propizepine	Antidepressant	10321-12-7		$C_{17}H_{20}N_4O$
Propofol	Anesthetic	2078-54-8		$C_{12}H_{18}O$

Table S1. *Cont.*

Common Name	Indication	CAS Number	Oral	Molecular Formula
Propoxate	Antidepressant	7036-58-0		$C_{15}H_{18}N_2O_2$
Propoxycaine	Anesthetic	86-43-1		$C_{16}H_{26}N_2O_3$
Propoxyphene	Analgesic	469-62-5		$C_{22}H_{29}NO_2$
Propranolol	Antihypertensive	525-66-6	Y	$C_{16}H_{21}NO_2$
Propylhexedrine	Adrenergic	101-40-6		$C_{10}H_{21}N$
Propylthiouracil	Thyroid	51-52-5	Y	$C_7H_{10}N_2OS$
Propyperone	Antipsychotic	3781-28-0		C23H33FN2O2
Propyphenazone	Analgesic	479-92-5		$C_{14}H_{18}N_2O$
Propyromazine Bromide	Antispasmodic	145-54-0		$C_{20}H_{23}BrN_2OS$
Proquazone	Antiinflammatory	22760-18-5		$C_{18}H_{18}N_2O$
Proquinolate	Antibacterial	1698-95-9		$C_{17}H_{21}NO_5$
Prorenoate Potassium	Antihypertensive	49848-01-3		$C_{23}H_{32}O_4$
Proroxan	Antihypertensive	33743-96-3		$C_{21}H_{23}NO_3$
Proscillaridin	Cardiotonic	466-06-8		$C_{30}H_{42}O_8$
Prospidium Chloride	Antineoplastic	23476-83-7		$C_{18}H_{36}Cl_4N_4O_2$
Prostalene	Prostaglandin	54120-61-5		$C_{22}H_{36}O_5$
Prosulpride	Antipsychotic	68556-59-2		$C_{16}H_{25}N_3O_4S$
Protein Hydrolysate	Plasma Volume Expander	852812-98-7		$C_{35}H_{47}N_7O_{10}$
Proterguride	Antipsychotic	77650-95-4		$C_{22}H_{32}N_4O$
Protheobromine	Diuretic	50-39-5		$C_{10}H_{14}N_4O_3$
Prothipendyl	Antipsychotic	303-69-5		$C_{16}H_{19}N_3S$
Prothixene	Antipsychotic	2622-24-4		$C_{18}H_{19}NS$
Protiofate	Antibiotic	58416-00-5		$C_{12}H_{16}O_6S$
Protionamide	Antibacterial	14222-60-7		$C_9H_{12}N_2S$
Protirelin	Pituitary	24305-27-9		$C_{16}H_{22}N_6O_4$
Protizinic Acid	Antiinflammatory	13799-03-6		C_{17}H17NO3S
Protokylol	Bronchodilator	136-70-9		$C_{18}H_{21}NO_5$
Protoporphyrin Ix	Hepatic Protectant	553-12-8		$C_{34}H_{34}N_4O_4$
Protoveratrine A	Antihypertensive	143-57-7		C_{41}H63NO14
Protoveratrine B	Antihypertensive	124-97-0		$C_{41}H_{63}NO_{15}$
Protriptyline	Antidepressant	438-60-8	Y	$C_{19}H_{21}N$
Proxazole	Muscle Relaxant	5696-09-3		$C_{17}H_{25}N_3O$
Proxibarbal	Sedative	2537-29-3		$C_{10}H_{14}N_2O_4$
Proxibutene	Analgesic	14089-84-0		$C_{22}H_{27}NO_2$
Proxicromil	Antihistaminic	60400-92-2		$C_{17}H_{18}O_5$
Proxorphan Tartrate	Analgesic	69815-38-9`		$C_{19}H_{25}NO_2$
Proxyphylline	Bronchodilator	603-00-9		$C_{10}H_{14}N_4O_3$
Prozapine	Antispasmodic	3426-08-2		$C_{21}H_{27}N$
Prucalopride	Gastroprokinetic	179474-81-8	Y	$C_{18}H_{26}ClN_3O_3$
Prulifloxacin	Antibacterial	123447-62-1	Y	$C_{21}H_{20}FN_3O_6S$
Pruvanserin	Antidepressant	443144-26-1		$C_{22}H_{21}FN_4O$
Pseudoephedrine	Decongestant	90-82-4	Y	$C_{10}H_{15}NO$
Psilocybine	Antipsychotic	520-52-5		$C_{12}H_{17}N_2O_4P$
Pteropterin	Unclassified	89-38-3		$C_{29}H_{33}N_9O_{12}$
Pumafentrine	Antimalarial	207993-12-2		$C_{29}H_{39}N_3O_3$
Pumaprazole	Antiulcerative	158364-59-1		$C_{19}H_{22}N_4O_2$
Pumitepa	Antineoplastic	42061-52-9		$C_{12}H_{19}N_8OP$
Pumosetrag	Gastroprokinetic	153062-94-3		$C_{15}H_{17}N_3O_2S$
Puromycin	Antineoplastic	53-79-2		$C_{22}H_{29}N_7O_5$
Pyrabrom	Antihistaminic	606-05-3		$C_{24}H_{30}BrN_7O_3$
Pyrantel	Anthelminithic	15686-83-6	Y	$C_{11}H_{14}N_2S$
Pyrathiazine	Anthelminithic	84-08-2		$C_{18}H_{20}N_2S$
Pyrazinamide	Antibacterial	98-96-4	Y	$C_5H_5N_3O$
Pyrazofurin	Antineoplastic	30868-30-5		$C_9H_{13}N_3O_6$
Pyricarbate	Glucocorticoid	1882-26-4		$C_{11}H_{15}N_3O_4$
Pyridarone	Antiarrhythmic	7035-04-3		$C_{13}H_9NO$
Pyridofylline	Vasodilator	53403-96-6		$C_9H_{12}N_4O_6S$
Pyridofylline	Vasodilator	65-23-6		$C_8H_{11}NO_3$
Pyridostigmine	Cholinergic	155-97-5	Y	$C_9H_{13}N_2O_2$
Pyrilamine	Antihistaminic	91-84-9		$C_{17}H_{23}N_3O$
Pyrimethamine	Antimalarial	58-14-0	Y	$C_{12}H_{13}ClN_4$
Pyrinoline	Antiarrhythmic	1740-22-3		$C_{27}H_{20}N_4O$
Pyrithione	Antibacterial	1121-30-8		C_5H_5NOS

Table S1. *Cont.*

Common Name	Indication	CAS Number	Oral	Molecular Formula
Pyrithioxin	Nootropic	1098-97-1		$C_{16}H_{20}N_2O_4S_2$
Pyrithyldione	Sedative	77-04-3		$C_9H_{13}NO_2$
Pyritidium	Antiprotozoal	3616-05-5		$C_{25}H_{24}N_7$
Pyrogallol	Neuromuscular Blocking Agent	87-66-1		$C_6H_6O_3$
Pyrovalerone	Nootropic	3563-49-3		$C_{16}H_{23}NO$
Pyroxamine Maleate	Antihistaminic	7009-68-9		$C_{18}H_{20}ClNO$
Pyrrobutamine Phosphate	Antihistaminic	91-82-7		$C_{20}H_{22}ClN$
Pyrrocaine	Anesthetic	2210-77-7		$C_{14}H_{20}N_2O$
Pyrroliphene	Analgesic	15686-97-2		$C_{23}H_{29}NO_2$
Pyrrolnitrin	Antifungal	1018-71-9		C_{10}H6Cl2N2O2
Pytamine	Antidepressant	15301-88-9		$C_{20}H_{28}N_2O$
Quadazocine Mesylate	Analgesic	71276-43-2		$C_{25}H_{37}NO_2$
Quadrosilan	Unclassified	33204-76-1		$C_{18}H_{28}O_4Si_4$
Quatacaine	Anesthetic	17692-45-4		$C_{14}H_{22}N_2O$
Quazepam	Sedative	36735-22-5	Y	$C_{17}H_{11}ClF_4N_2S$
Quazinone	Cardiotonic	70018-51-8		$C_{11}H_{10}ClN_3O$
Quazodine	Cardiotonic	4015-32-1		$C_{12}H_{14}N_2O_2$
Quazolast	Bronchodilator	86048-40-0		$C_{12}H_7ClN_2O_3$
Quercetin	Capillary Protectant	117-39-5	Y	$C_{15}H_{10}O_7$
Quetiapine	Antipsychotic	111974-69-7	Y	$C_{21}H_{25}N_3O_2S$
Quifenadine	Antihistaminic	10447-39-9		$C_{20}H_{23}NO$
Quiflapon Sodium	Antiinflammatory	136668-42-3		$C_{34}H_{35}ClN_2O_3S$
Quillifoline	Antibacterial	15301-89-0		$C_{21}H_{24}ClNO_2$
Quilostigmine	Cholinergic	139314-01-5		$C_{23}H_{27}N_3O_2$
Quinacainol	Antiarrhythmic	86073-85-0		$C_{21}H_{30}N_2O$
Quinacillin	Antibiotic	1596-63-0		$C_{18}H_{16}N_4O_6S$
Quinacrine	Antimalarial	83-89-6	Y	$C_{23}H_{30}ClN_3O$
Quinagolide	Antipsychotic	87056-78-8	Y	$C_{20}H_{33}N_3O_3S$
Quinaldine Blue	Antineoplastic	2768-90-3		$C_{25}H_{25N2.Cl}$
Quinapril	Antihypertensive	85441-61-8	Y	$C_{25}H_{30}N_2O_5$
Quinaprilat	Antihypertensive	82768-85-2	Y	$C_{23}H_{26}N_2O_5$
Quinazosin	Antihypertensive	15793-38-1		$C_{17}H_{23}N_5O_2$
Quinbolone	Steroid	2487-63-0		$C_{24}H_{32}O_2$
Quincarbate	Urologic	54340-59-9		$C_{17}H_{18}ClNO_6$
Quindecamine Acetate	Antibacterial	19056-26-9		$C_{30}H_{38}N_4$
Quindonium Bromide	Antiarrhythmic	130-81-4		$C_{16}H_{20}NO.Br$
Quindoxin	Antibacterial	2423-66-7		$C_8H_6N_2O_2$
Quinelorane	Antihypertensive	97466-90-5		$C_{14}H_{22}N_4$
Quinestradol	Estrogen	1169-79-5		$C_{23}H_{32}O_3$
Quinestrol	Estrogen	152-43-2		$C_{25}H_{32}O_2$
Quinethazone	Diuretic	73-49-4		$C_{10}H_{12}ClN_3$O3S
Quinetolate	Muscle Relaxant	27166-18-3		$C_{14}H_{19}N_3O$
Quinetolate	Muscle Relaxant	131-64-6		$C_{12}H_{19}NO_3$
Quinezamide	Antidiabetic	77197-48-9		$C_{13}H_{12}N_4O$
Quinfamide	Antiamebic	62265-68-3		$C_{16}H_{13}Cl_2NO_4$
Quingestanol Acetate	Progestogen	3000-39-3		$C_{27}H_{36}O_3$
Quingestrone	Progestogen	67-95-8		C_{26}H38O2
Quinidine	Antimalarial	56-54-2	Y	$C_{20}H_{24}N_2O_2$
Quinine	Antimalarial	130-95-0	Y	$C_{20}H_{24}N_2O_2$
Quinocide	Antimalarial	525-61-1		$C_{15}H_{21}N_3O$
Quinpirole	Antihypertensive	80373-22-4		$C_{13}H_{21}N_3$
Quinterenol Sulfate	Bronchodilator	13757-97-6		$C_{14}H_{18}N_2O_2$
Quintiofos	Ectoparasiticide	1776-83-6		$C_{17}H_{16}NO_2PS$
Quinuclium Bromide	Antihypertensive	35425-83-3		$C_{14}H_{18}BrNO$
Quinupramine	Antidepressant	31721-17-2		$C_{21}H_{24}N_2$
Quipazine Maleate	Antidepressant	4774-24-7		$C_{13}H_{15}N_3$
Quisultazine	Antidepressant	64099-44-1		$C_{21}H_{25}N_3O_2$S2
Rabeprazole	Antiulcerative	117976-89-3	Y	$C_{18}H_{21}N_3O_3S$
Racecadotril	Antidiarrheal	81110-73-8	Y	$C_{21}H_{23}NO_4S$
Racefemine	Antispasmodic	22232-57-1		$C_{18}H_{23}NO$
Racementhol	Decongestant	89-78-1		$C_{10}H_{20}O$
Racemethorphan	Antitussive	510-53-2		$C_{18}H_{25}NO$

Table S1. *Cont.*

Common Name	Indication	CAS Number	Oral	Molecular Formula
Racemetirosine	Unclassified	658-48-0		$C_{10}H_{13}NO_3$
Racemoramide	Analgesic	545-59-5		$C_{25}H_{32}N_2O_2$
Racemorphan	Analgesic	297-90-5		$C_{17}H_{23}NO$
Racephedrine	Bronchodilator	90-81-3		$C_{10}H_{15}NO$
Racephenicol	Antibacterial	847-25-6		$C_{12}H_{15}Cl_2NO_5S$
Racepinephrine	Bronchodilator	329-65-7		$C_9H_{13}NO_3$
Ractopamine	Steroid	97825-25-7		$C_{18}H_{23}NO_3$
Radafaxine	Antidepressant	192374-14-4		$C_{13}H_{18}ClNO_2$
Rafabegron	Antidiabetic	244081-42-3		$C_{21}H_{23}ClN2O4$
Rafoxanide	Anthelminithic	22662-39-1		$C_{19}H_{11}Cl_2I_2NO_3$
Ragaglitazar	Antidiabetic	222834-30-2		$C_{25}H_{25}NO_5$
Ralitoline	Anticonvulsant	93738-40-0		$C_{13}H_{13}ClN2O2S$
Raloxifene	Bone Resorption Inhibitor	84449-90-1	Y	$C_{28}H_{27}NO_4S$
Raltitrexed	Antineoplastic	112887-68-0		$C_{21}H_{22}N_4O_6S$
Raluridine	Antiviral	119644-22-3		$C_9H_{10}ClFN2O4$
Ramatroban	Antithrombotic	116649-85-5	Y	$C_{21}H_{21}FN_2O_4S$
Ramciclane	Antihypotensive	96743-96-3		$C_{21}H_{33}NO$
Ramelteon	Anxiolytic	196597-26-9	Y	$C_{16}H_{21}NO_2$
Ramifenazone	Analgesic	3615-24-5		$C_{14}H_{19}N_3O$
Ramipril	Antihypertensive	87333-19-5	Y	$C_{23}H_{32}N_2O_5$
Ramiprilat	Antihypertensive	87269-97-4	Y	$C_{21}H_{28}N_2O_5$
Ramixotidine	Antiulcerative	84071-15-8		$C_{16}H_{21}N_3O_3S$
Ramnodigin	Cardiotonic	33156-28-4		$C_{29}H_{44}O_6$
Ramosetron	Antidepressant	132036-88-5	Y	$C_{17}H_{17}N_3O$
Ranelic Acid	Bone Resorption Inhibitor	135459-90-4		$C_{12}H_{10}N_2O_8S$
Ranimustine	Antineoplastic	58994-96-0		$C_{10}H_{18}ClN_3O_7$
Ranirestat	Antidiabetic	147254-64-6	Y	$C_{17}H_{11}BrFN_3O_4$
Ranitidine	Antiulcerative	66357-35-5	Y	$C_{13}H_{22}N_4O_3S$
Ranolazine	Antianginal	95635-55-5	Y	$C_{24}H_{33}N_3O_4$
Rapacuronium	Neuromuscular Blocking Agent	465499-11-0		$C_{37}H_{61}N_2O_4$
Rasagiline Mesylate	Antiparkinsonian	136236-51-6	Y	$C_{12}H_{13}N$
Rathyronine	Thyroid	3130-96-9		$C_{15}H_{12}I_3NO_4$
Ravuconazole	Antifungal	182760-06-1		$C_{22}H_{17}F_2N_5OS$
Raxofelast	Hepatic Protectant	128232-14-4		$C_{15}H_{18}O_5$
Razaxaban	Anticoagulant	218298-21-6		$C_{24}H_{20}F_4N_8O_2$
Razinodil	Cardiotonic	30271-85-3		$C_{27}H_{34}N_4O_{10}$
Razobazam	Anxiolytic	78466-98-5		$C_{14}H_{14}N_4O_2$
Razoxane	Antianginal	21416-67-1		$C_{11}H_{16}N_4O_4$
Rebamipide	Antiulcerative	90098-04-7	Y	$C_{19}H_{15}ClN_2O_4$
Rebimastat	Antineoplastic	259188-38-0		$C_{23}H_{41}N_5O_5S$
Reboxetine	Antidepressant	71620-89-8	Y	$C_{19}H_{23}NO_3$
Recainam	Antiarrhythmic	74738-24-2		$C_{15}H_{25}N_3O$
Reclazepam	Sedative	76053-16-2		$C_{18}H_{13}Cl_2N_3O_2$
Regadenoson	Vasodilator	313348-27-5		$C_{15}H_{18}N_8O_5$
Reglitazar	Antidiabetic	170861-63-9		$C_{22}H_{20}N_2O_5$
Relcovaptan	Antihypertensive	150375-75-0		$C_{28}H_{27}Cl_2N_3O_7S$
Relomycin	Antibacterial	1404-48-4		$C_{46}H_{79}NO_{17}$
Remacemide	Anticonvulsant	128298-28-2		$C_{17}H_{20}N_2O$
Remifentanil	Analgesic	132875-61-7		$C_{20}H_{28}N_2O_5$
Remikiren	Antihypertensive	126222-34-2		$C_{33}H_{50}N_4O_6S$
Remiprostol	Antiulcerative	110845-89-1		$C_{25}H_{36}O_5$
Remoxipride	Antipsychotic	80125-14-0	Y	$C_{16}H_{23}BrN_2O_3$
Renanolone	Antiinflammatory	565-99-1		$C_{21}H_{32}O_3$
Rentiapril	Antihypertensive	80830-42-8		$C_{13}H_{15}NO_4S_2$
Renzapride	Gastroprokinetic	112727-80-7		$C_{16}H_{22}ClN_3O_2$
Repagermanium	Immunomodulator	12758-40-6		$C_6H_{10}Ge_2O_7$
Repaglinide	Antidiabetic	135062-02-1	Y	$C_{27}H_{36}N_2O_4$
Reparixin	Analgesic	266359-83-5	Y	$C_{14}H21NO3S$
Repinotan	Nootropic	144980-29-0		$C_{21}H_{24}N_2O_4S$
Repirinast	Antihistaminic	73080-51-0		$C_{20}H_{21}NO_5$
Repromicin	Antibacterial	56689-42-0		$C_{31}H_{51}NO_8$
Reproterol	Bronchodilator	54063-54-6		$C_{18}H_{23}N_5O_5$

Table S1. *Cont.*

Common Name	Indication	CAS Number	Oral	Molecular Formula
Rescimetol	Antihypertensive	73573-42-9		$C_{33}H_{38}N_2O_8$
Rescinnamine	Antihypertensive	24815-24-5		$C_{35}H_{42}N_2O_9$
Reserpine	Antihypertensive	50-55-5		$C_{33}H_{40}N_2O_9$
Resiquimod	Antiviral	144875-48-9		$C_{17}H_{22}N_4O_2$
Resocortol Butyrate	Antiinflammatory	76738-96-0		$C_{26}H_{38}O_5$
Resorantel	Anthelminithic	20788-07-2		$C_{13}H_{10}BrNO_3$
Resorcin Brown	Anthelminithic	30282-44-1		$C_{20}H_{18}N_4O_5S$
Retapamulin	Antibiotic	224452-66-8		$C_{30}H_{47}NO_4S$
Retelliptine	Antineoplastic	72238-02-9		$C_{25}H_{32}N_4O$
Retigabine	Anticonvulsant	150812-12-7	Y	C16H18FN3O2
Revaprazan	Antiulcerative	199463-33-7	Y	$C_{22}H_{23}FN_4$
Revatropate	Bronchodilator	149926-91-0		$C_{19}H_{27}NO_4S$
Revenast	Bronchodilator	85673-87-6		$C_{27}H_{29}N_5O$
Revizinone	Cardiotonic	133718-29-3		$C_{26}H_{29}N_5O_3$
Revospirone	Anxiolytic	95847-87-3		$C_{18}H_{21}N_5O_3S$
Ribavirin	Antiviral	36791-04-5	Y	$C_8H_{12}N_4O_5$
Riboprine	Antineoplastic	7724-76-7		$C_{15}H_{21}N_5O_4$
Ribostamycin	Antibiotic	25546-65-0		$C_{17}H_{34}N_4O_{10}$
Ricasetron	Antiemetic	117086-68-7		$C_{19}H_{27}N_3O$
Ricinolsulfuric Acid	Unclassified	36634-48-7		$C_{18}H_{34}O_6S$
Ridazolol	Antihypertensive	83395-21-5		$C_{15}H_{18}Cl_2N_4O_3$
Ridogrel	Antithrombotic	110140-89-1		$C_{18}H_{17}F_3N_2O_3$
Rifabutin	Antibacterial	72559-06-9	Y	$C_{46}H_{62}N_4O_{11}$
Rifalazil	Antibacterial	129791-92-0	Y	$C_{51}H_{64}N_4O_{13}$
Rifametane	Antibacterial	94168-98-6		$C_{44}H_{60}N_4O_{12}$
Rifamexil	Antibacterial	113102-19-5		$C_{42}H_{55}N_3O_{11}S$
Rifamide	Antibacterial	2750-76-7		$C_{43}H_{58}N_2O_{13}$
Rifampin	Antibacterial	13292-46-1		$C_{43}H_{58}N_4O_{12}$
Rifamycin	Antibiotic	6998-60-3	Y	$C_{37}H_{47}NO_{12}$
Rifapentine	Antibacterial	61379-65-5	Y	$C_{47}H_{64}N_4O_{12}$
Rifaximin	Antibacterial	80621-81-4	Y	$C_{43}H_{51}N_3O_{11}$
Rilapine	Antipsychotic	79781-95-6		$C_{22}H_{20}ClN_3$
Rilapladib	Antihyperlipidemic	412950-08-4	Y	$C_{40}H_{38}F_5N_3O_3S$
Rilmakalim	Antihypertensive	132014-21-2		$C_{21}H_{23}NO_5S$
Rilmazafone	Sedative	99593-25-6	Y	$C_{21}H_{20}Cl_2N_6O_3$
Rilmenidine	Antihypertensive	54187-04-1	Y	$C_{10}H_{16}N_2O$
Rilopirox	Antifungal	104153-37-9		$C_{19}H_{16}ClNO_4$
Rilozarone	Antiarrhythmic	79282-39-6		$C_{32}H_{36}BrClN_2O_2$
Rilpivirine	Antiviral	500287-72-9	Y	$C_{22}H_{18}N_6$
Riluzole	Nootropic	1744-22-5	Y	$C_8H_5F_3N_2OS$
Rimacalib	Unclassified	215174-50-8		C_{22}H23FN4O2
Rimantadine	Antiviral	13392-28-4	Y	$C_{12}H_{21}N$
Rimazolium Metilsulfate	Analgesic	28610-84-6		$C_{13}H_{19}N_2O_3.CH_3O_4S$
Rimcazole	Antipsychotic	75859-04-0		$C_{21}H_{27}N_3$
Rimeporide	Cardiotonic	187870-78-6		$C_{11}H_{15}N_3O_5S_2$
Rimexolone	Antiinflammatory	49697-38-3		$C_{24}H_{34}O_3$
Rimiterol	Bronchodilator	32953-89-2		$C_{12}H_{17}NO_3$
Rimonabant	Antiobesity	168273-06-1		$C_{22}H_{21}Cl_3N_4O$
Rimoprogin	Antibiotic	37750-83-7		$C_8H_7IN_2OS$
Riodipine	Antiarrhythmic	71653-63-9		$C_{18}H_{19}F_2NO_5$
Rioprostil	Antiulcerative	77287-05-9		$C_{21}H_{38}O_4$
Ripazepam	Anxiolytic	26308-28-1		$C_{15}H_{16}N_4O$
Ripisartan	Antihypertensive	148504-51-2		$C_{23}H_{22}N_8O$
Risarestat	Antidiabetic	79714-31-1		$C_{16}H_{21}NO_4S$
Risedronic Acid	Bone Resorption Inhibitor	105462-24-6		$C_7H_{11}NO_7P_2$
Risocaine	Anesthetic	94-12-2		$C_{10}H_{13}NO_2$
Risotilide	Antiarrhythmic	120688-08-6		$C_{15}H_{27}N_3O_4S_2$
Rispenzepine	Bronchodilator	96449-05-7		$C_{19}H_{20}N_4O_2$
Risperidone	Antipsychotic	106266-06-2	Y	$C_{23}H_{27}FN_4O_2$
Ristianol Phosphate	Immunomodulator	78092-65-6		$C_8H_{11}NOS$
Ritanserin	Anxiolytic	87051-43-2		$C_{27}H_{25}F_2N_3OS$
Ritiometan	Unclassified	34914-39-1		$C_7H_{10}O_6S_3$
Ritipenem	Antibiotic	84845-57-8		$C_{10}H_{12}N_2O_6S$

Table S1. *Cont.*

Common Name	Indication	CAS Number	Oral	Molecular Formula
Ritobegron	Antidiabetic	255734-04-4		$C_{21}H_{27}NO_5$
Ritodrine	Tocolytic	26652-09-5		$C_{17}H_{21}NO_3$
Ritolukast	Bronchodilator	111974-60-8		$C_{17}H_{13}F_3N_2O_3S$
Ritonavir	Antiviral	155213-67-5	Y	$C_{37}H_{48}N_6O_5S_2$
Ritropirronium Bromide	Neuromuscular Blocking Agent	58493-54-2		$C_{19}H_{28}BrNO_3$
Ritrosulfan	Antineoplastic	4148-16-7		$C_{10}H_{24}N_2O_8S_2$
Rivanicline	Nootropic	15585-43-0		$C_{10}H_{14}N_2$
Rivaroxaban	Anticoagulant	366789-02-8	Y	$C_{19}H_{18}ClN_3O_5S$
Rivastigmine	Nootropic	123441-03-2	Y	$C_{14}H_{22}N_2O_2$
Rivenprost	Anticoagulant	256382-08-8		$C_{24}H_{34}O_6S$
Rivoglitazone	Antidiabetic	185428-18-6		$C_{20}H_{19}N_3O_4S$
Rizatriptan Benzoate	Antimigraine	144034-80-0	Y	$C_{15}H_{19}N_5$
Rizatriptan Benzoate	Antimigraine	65-85-0	Y	$C_7H_6O_2$
Robenacoxib	Antiinflammatory	220991-32-2		$C_{16}H_{13}F_4NO_2$
Robenidine	Antibiotic	25875-51-8		$C_{15}H_{13}Cl_2N_5$
Rocastine	Antihistaminic	91833-49-7		$C_{13}H_{19}N_3OS$
Rocepafant	Antithrombotic	132579-32-9		$C_{26}H_{23}ClN_6OS_2$
Rociclovir	Antiviral	108436-80-2		$C_{15}H_{25}N_5O_3$
Rociverine	Antispasmodic	53716-44-2		$C_{20}H_{37}NO_3$
Rocuronium	Neuromuscular Blocking Agent	143558-00-3		$C_{32}H_{53}N_2O_4$
Rodocaine	Anesthetic	38821-80-6		$C_{18}H_{25}ClN_2O$
Rodorubicin	Antineoplastic	96497-67-5		$C_{48}H_{64}N_2O_{17}$
Rofecoxib	Antiinflammatory	162011-90-7		$C_{17}H_{14}O_4S$
Rofelodine	Muscle Relaxant	76696-97-4		$C_{13}H_{14}N_2O$
Rofleponide	Steroid	144459-70-1		$C_{25}H_{34}F_2O_6$
Roflumilast	Bronchodilator	162401-32-3	Y	$C_{17}H_{14}Cl_2F_2N_2O_3$
Roflurane	Anesthetic	679-90-3		$C_3H_4BrF_3O$
Rogletimide	Antineoplastic	92788-10-8		$C_{12}H_{14}N_2O_2$
Rokitamycin	Antibiotic	74014-51-0		$C_{42}H_{69}NO_{15}$
Rolafagrel	Antithrombotic	89781-55-5		$C_{14}H_{12}N_2O_2$
Roletamide	Sedative	10078-46-3		$C_{16}H_{19}NO_4$
Rolgamidine	Antidiarrheal	66608-04-6		$C_9H_{16}N_4O$
Rolicyclidine	Cholinergic	2201-39-0		$C_{16}H_{23}N$
Rolicyprine	Antidepressant	2829-19-8		$C_{14}H_{16}N_2O_2$
Rolipram	Antidepressant	61413-54-5		$C_{16}H_{21}NO_3$
Rolitetracycline	Antibiotic	751-97-3		$C_{27}H_{33}N_3O_8$
Rolodine	Muscle Relaxant	1866-43-9		$C_{14}H_{14}N_4$
Rolziracetam	Nootropic	18356-28-0		$C_7H_9NO_2$
Romazarit	Antiinflammatory	109543-76-2	Y	$C_{15}H_{16}ClNO_4$
Romergoline	Antiparkinsonian	107052-56-2		$C_{20}H_{22}N_4O2$
Romifenone	Antiarrhythmic	38373-83-0		$C_{13}H_{17}NO_3$
Romifidine	Sedative	65896-16-4		$C_9H_9BrFN_3$
Romurtide	Immunomodulator	78113-36-7		$C_{43}H_{78}N_6O_{13}$
Ronactolol	Antiarrhythmic	90895-85-5		$C_{20}H_{26}N_2O_4$
Ronidazole	Antiprotozoal	7681-76-7		$C_6H_8N_4O_4$
Ronifibrate	Antihyperlipidemic	42597-57-9	Y	$C_{19}H_{20}ClNO_5$
Ronipamil	Vasodilator	85247-77-4		$C_{32}H_{48}N_2$
Ropinirole	Antiparkinsonian	91374-21-9	Y	$C_{16}H_{24}N_2O$
Ropitoin	Antiarrhythmic	56079-81-3		$C_{30}H_{33}N_3O_3$
Ropivacaine	Anesthetic	84057-95-4		$C_{17}H_{26}N_2O$
Ropizine	Anticonvulsant	3601-19-2		$C_{24}H_{26}N_4$
Roquinimex	Immunomodulator	84088-42-6		$C_{18}H_{16}N_2O_3$
Rosaprostol	Antiulcerative	56695-65-9		$C_{18}H_{34}O_3$
Rosaramicin	Antibacterial	35834-26-5		$C_{31}H_{51}NO_9$
Rosiglitazone	Antidiabetic	122320-73-4	Y	$C_{18}H_{19}N_3O_3S$
Rosoxacin	Antibacterial	40034-42-2		$C_{17}H_{14}N_2O_3$
Rostafuroxin	Antihypertensive	156722-18-8	Y	$C_{23}H_{34}O_4$
Rostaporfin	Opthalmic	284041-10-7		$C_{37}H_{42}Cl_2N_4O_2Sn$
Rosterolone	Androgen	79243-67-7		$C_{23}H_{38}O_2$
Rosuvastatin	Antihyperlipidemic	287714-41-4	Y	$C_{22}H_{28}FN_3O_6S$
Rotamicillin	Antibiotic	55530-41-1		$C_{28}H_{31}N_5O_5S$

Table S1. *Cont.*

Common Name	Indication	CAS Number	Oral	Molecular Formula
Rotenone	Antibacterial	83-79-4		$C_{23}H_{22}O_6$
Rotigaptide	Antiarrhythmic	355151-12-1		$C_{28}H_{39}N_7O_9$
Rotigotine	Antiparkinsonian	99755-59-6		$C_{19}H_{25}NOS$
Rotoxamine	Antihistaminic	5560-77-0		$C_{16}H_{19}ClN_2O$
Rotraxate	Antiulcerative	92071-51-7		$C_{17}H_{23}NO_3$
Roxadimate	Dermatologic	58882-17-0		$C_{15}H_{23}NO_4$
Roxarsone	Antibacterial	121-19-7		$C_6H_6AsNO_6$
Roxatidine Acetate	Antiulcerative	78628-28-1		$C_{19}H_{28}N_2O_4$
Roxibolone	Steroid	60023-92-9		$C_{21}H_{28}O_5$
Roxifiban Acetate	Antithrombotic	170902-47-3		$C_{21}H_{29}N_5O_6$
Roxindole	Antidepressant	112192-04-8		$C_{23}H_{26}N_2O$
Roxithromycin	Antibiotic	80214-83-1	Y	$C_{41}H_{76}N_2O_{15}$
Roxolonium Metilsulfate	Antihypertensive	53862-80-9		$C_{38}H_{63}NO_8S$
Roxoperone	Antipsychotic	2804-00-4		$C_{19}H_{23}FN_2O_3$
Rubitecan	Antineoplastic	91421-42-0		$C_{20}H_{15}N_3O_6$
Ruboxistaurin	Antineoplastic	169939-94-0		$C_{28}H_{28}N_4O_3$
Rufinamide	Anticonvulsant	106308-44-5	Y	$C_{10}H_8F_2N_4O$
Rufloxacin	Antibiotic	101363-10-4	Y	$C_{17}H_{18}FN_3O_3S$
Rupatadine	Antihistaminic	158876-82-5	Y	$C_{26}H26ClN3$
Rupintrivir	Antiviral	223537-30-2		$C_{31}H39FN4O7$
Rutamycin	Antifungal	1404-59-7		$C_{44}H_{72}O_{11}$
Rutin	Capillary Protectant	153-18-4		$C_{27}H_{30}O_{16}$
Ruvazone	Analgesic	20228-27-7		$C_{12}H_{14}N_2O_4$
Ruzadolane	Unclassified	115762-17-9		$C_{18}H_{19}F_2N_5S$
Sabarubicin	Antineoplastic	211100-13-9		$C_{32}H_{37}NO_{13}$
Sabcomeline	Nootropic	159912-53-5		$C_{10}H_{15}N_3O$
Sabeluzole	Anticonvulsant	104383-17-7		$C_{22}H_{26}FN_3O_2S$
Sabiporide	Cardiotonic	261505-80-0		$C_{18}H_{19}F_3N_6O_2$
Safinamide	Diuretic	133865-89-1	Y	$C_{17}H_{19}FN_2O_2$
Safingol	Antineoplastic	15639-50-6		$C_{18}H_{39}NO_2$
Safironil	Dermatologic	134377-69-8		$C_{15}H_{23}N_3O_4$
Safrazine	Antipsychotic	33419-68-0		$C_{11}H_{16}N_2O_2$
Sagandipine	Antihypertensive	126294-30-2		$C_{27}H_{31}FN_2O_5$
Salacetamide	Analgesic	487-48-9		$C_9H_9NO_3$
Salafibrate	Antihyperlipidemic	64496-66-8		$C_{32}H_{32}Cl_2O_{10}$
Salantel	Anthelminithic	36093-47-7		$C_{20}H_{11}Cl_2I_2NO3$
Salazodine	Antihypertensive	22933-72-8		$C_{18}H_{15}N_5O_6S$
Salazosulfadimidine	Antibiotic	2315-08-4		$C_{19}H_{17}N_5O_5S$
Salazosulfamide	Antibiotic	139-56-0		$C_{13}H_{11}N_3O_5S$
Salazosulfathiazole	Antibiotic	515-58-2		$C_{16}H_{12}N_4O_5S_2$
Salcaprozic Acid	Unclassified	183990-46-7		$C_{15}H_{21}NO_4$
Salclobuzic Acid	Unclassified	387825-03-8		$C_{11}H_{12}ClNO_4$
Salcolex	Analgesic	2016-36-6		$C_7H_5O_3.C_5H_{14}NO$
Salethamide Maleate	Analgesic	46803-81-0		$C_{13}H_{20}N_2O_2$
Salfluverine	Antispasmodic	587-49-5		$C_{14}H10F3NO2$
Salicin	Analgesic	138-52-3		$C_{13}H18O7$
Salicyl Alcohol	Anesthetic	90-01-7		$C_7H_8O_2$
Salicylamide	Analgesic	65-45-2		$C_7H_7NO_2$
Salicylanilide	Antifungal	87-17-2		$C_{13}H_{11}NO_2$
Salicylic Acid	Dermatologic	69-72-7	Y	$C_7H_6O_3$
Salicylic Anhydride Diacetate	Antiinflammatory	1466-82-6		$C_{18}H_{14}O_7$
Salicyluric Acid	Antiinflammatory	487-54-7		C9H9NO4
Salinazid	Antibiotic	495-84-1		$C_{13}H_{11}N_3O_2$
Salinomycin	Antibiotic	53003-10-4		$C_{42}H_{70}O_{11}$
Salmefamol	Bronchodilator	18910-65-1		$C_{19}H_{25}NO_4$
Salmeterol	Bronchodilator	89365-50-4		$C_{25}H_{37}NO_4$
Salmeterol Xinafoate	Bronchodilator	86-48-6		$C_{11}H_8O_3$
Salmisteine	Mucolytic	89767-59-9		$C_{14}H_{15}NO_6S$
Salnacedin	Antiinflammatory	87573-01-1		$C_{12}H_{13}NO_5S$
Salprotoside	Cardiotonic	33779-37-2		$C_{25}H_{30}O_{10}$
Salsalate	Analgesic	552-94-3	Y	$C_{14}H_{10}O_5$
Salverine	Analgesic	6376-26-7		$C_{19}H_{24}N_2O_2$
Sameridine	Anesthetic	143257-97-0		$C_{21}H_{34}N_2O$

Table S1. *Cont.*

Common Name	Indication	CAS Number	Oral	Molecular Formula
Samixogrel	Antithrombotic	133276-80-9		$C_{25}H_{25}ClN_2O_4S$
Sampatrilat	Antihypertensive	129981-36-8		$C_{26}H_{40}N_4O_9S$
Sampirtine	Analgesic	115911-28-9		$C_{12}H_{12}FN_3$
Sancycline	Antibacterial	808-26-4		$C_{21}H_{22}N_2O_7$
Sanfetrinem Cilexetil	Antibacterial	141646-08-4		$C_{23}H_{33}NO_8$
Sanfetrinem Sodium	Antibacterial	156769-21-0		$C_{14}H_{19}NO_5$
Santonin	Anthelminithic	481-06-1		$C_{15}H_{18}O_3$
Saperconazole	Antifungal	110588-57-3		$C_{35}H_{38}F_2N_8O_4$
Saprisartan Potassium	Antihypertensive	146623-69-0		$C_{25}H_{22}BrF_3N_4O_4S$
Saquinavir	Antiviral	127779-20-8	Y	$C_{38}H_{50}N_6O_5$
Sarafloxacin	Antibiotic	98105-99-8	Y	$C_{20}H_{17}F_2N_3O_3$
Sarakalim	Antihypertensive	148430-28-8		$C_{20}H_{19}F_3N_2O_4$
Saralasin	Antihypertensive	34273-10-4		$C_{42}H_{65}N_{13}O_{10}$
Sarcolysin	Antineoplastic	531-76-0		$C_{13}H_{18}Cl_2N_2O_2$
Sardomozide	Antipsychotic	149400-88-4		$C_{11}H_{14}N_6$
Saredutant	Bronchodilator	142001-63-6		$C_{31}H_{35}Cl_2N_3O_2$
Saripidem	Sedative	103844-86-6		$C_{19}H_{20}ClN_3O$
Sarizotan	Antiparkinsonian	351862-32-3		$C_{22}H_{21}FN_2O$
Sarmazenil	Anxiolytic	78771-13-8		$C_{15}H_{14}ClN_3O_3$
Sarmoxicillin	Antibacterial	67337-44-4		$C_{21}H_{27}N_3O_6S$
Sarpicillin	Antibacterial	40966-79-8		$C_{21}H_{27}N_3O_5S$
Sarpogrelate	Antithrombotic	125926-17-2		$C_{24}H_{31}NO_6$
Satavaptan	Hepatoprotectant	185913-78-4		$C_{33}H_{45}N_3O_8S$
Saterinone	Cardiotonic	102669-89-6		$C_{27}H_{30}N_4O_4$
Satigrel	Antithrombotic	111753-73-2		$C_{20}H_{19}NO_4$
Satranidazole	Antiamebic	56302-13-7		$C_8H_{11}N5O5S$
Saviprazole	Antiulcerative	121617-11-6		$C_{15}H_{10}F_7N_3O_2S_2$
Savoxepin	Antidepressant	79262-46-7		$C_{25}H_{26}N_2O$
Saxagliptin	Antidiabetic	361442-04-8		$C_{18}H_{25}N_3O2$
Scopinast	Antihistamine	145574-90-9		$C_{31}H_{31}F_2NO_5$
Scopolamine	Antispasmodic	51-34-3		$C_{17}H_{21}NO_4$
Secalciferol	Steroid	55721-11-4		$C_{27}H_{44}O_3$
Seclazone	Antiinflammatory	29050-11-1		$C_{10}H_8ClNO_3$
Secnidazole	Antiamebic	3366-95-8	Y	$C_7H_{11}N_3O_3$
Secobarbital	Sedative	76-73-3	Y	$C_{12}H_{18}N_2O_3$
Secoverine	Antispasmodic	57558-44-8		$C_{22}H_{35}NO_2$
Sedecamycin	Antibacterial	23477-98-7		$C_{27}H_{35}NO_8$
Seganserin	Antidepressant	87729-89-3		$C_{29}H_{27}F_2N_3O$
Segesterone	Progestogen	7690-08-6		$C_{21}H_{28}O_3$
Seglitide	Antidiabetic	81377-02-8		$C_{44}H_{56}N_8O_7$
Selamectin	Anthelminithic	220119-17-5		$C_{43}H_{63}NO_{11}$
Selegiline	Antiparkinsonian	14611-51-9	Y	$C_{13}H_{17}N$
Seletracetam	Anticonvulsant	357336-74-4		$C_{10}H_{14}F_2N_2O$
Selfotel	Antipsychotic	110347-85-8		$C_7H_{14}NO_5P$
Seliciclib	Antineoplastic	186692-46-6	Y	$C_{19}H_{26}N_6O$
Selodenoson	Antiarrhythmic	110299-05-3		$C_{17}H_{24}N_6O_4$
Selprazine	Antipsychotic	103997-59-7		$C_{24}H_{31}N_3O_3$
Semapimod	Antiinflammatory	352513-83-8	Y	$C_{34}H_{52}N_{18}O_2$
Sematilide	Antiarrhythmic	101526-83-4		$C_{14}H_{23}N_3O_3S$
Semaxanib	Antineoplastic	194413-58-6		$C_{15}H_{14}N_2O$
Semduramicin	Antibiotic	113378-31-7		$C_{45}H_{76}O_{16}$
Semorphone	Analgesic	88939-40-6		$C_{19}H_{23}NO_5$
Semotiadil	Antianginal	116476-13-2		$C_{29}H_{32}N_2O_6S$
Semustine	Antineoplastic	13909-09-6		$C_{10H18ClN3O2}$
Senazodan	Cardiotonic	98326-32-0		$C_{15}H_{14}N_4O$
Seocalcitol	Steroid	134404-52-7		$C_{30}H_{46}O_3$
Seperidol	Antipsychotic	10457-91-7		$C_{22}H_{22}ClF_4NO_2$
Sepimostat	Anticoagulant	103926-64-3		$C_{21}H_{19}N_5O_2$
Seprilose	Antirheumatic	133692-55-4		$C_{16}H_{30}O_6$
Seproxetine	Antidepressant	126924-38-7		$C_{16}H_{16}F_3NO$
Sequifenadine	Antihistaminic	57734-69-7		$C_{22}H_{27}NO$
Seratrodast	Antiinflammatory	112665-43-7	Y	$C_{22}H_{26}O_4$
Serazapine	Anxiolytic	115313-22-9		$C_{22}H_{23}N_3O_2$

Table S1. *Cont.*

Common Name	Indication	CAS Number	Oral	Molecular Formula
Serfibrate	Antihyperlipidemic	54657-98-6		$C_{16}H_{20}ClNO_5S$
Sergolexole Maleate	Antimigraine	108674-86-8		$C_{26}H_{36}N_2O_3$
Sermetacin	Antiinflammatory	57645-05-3		$C_{22}H_{21}ClN_2O_6$
Sertaconazole	Antifungal	99592-32-2		$C_{20}H_{15}Cl_3N_2OS$
Sertindole	Antipsychotic	106516-24-9	Y	$C_{24}H_{26}ClFN_4O$
Sertraline	Antidepressant	79617-96-2	Y	$C_{17}H_{17}Cl_2N$
Setastine	Antihistaminic	64294-95-7	Y	$C_{22}H_{28}ClNO$
Setazindol	Antidepressant	28570-99-2		$C_{15}H_{16}ClNO$
Setipafant	Antithrombotic	132418-35-0		$C_{26}H_{23}ClN_6O_2S$
Setiptiline	Antidepressant	57262-94-9		$C_{19}H_{19}N$
Setoperone	Antipsychotic	86487-64-1		$C_{21}H_{24}FN_3O_2S$
Sevitropium Mesilate	Bronchodilator	88199-75-1		$C_{24}H_{29}NO_5S_2$
Sevoflurane	Anesthetic	28523-86-6		$C_4H_3F_7O$
Sevopramide	Antidepressant	57227-17-5		$C_{29}H_{43}N_3O_3$
Sezolamide	Antiglaucoma	123308-22-5		$C_{11}H_{18}N_2O_4S_3$
Sibenadet	Respiratory Stimulant	154189-40-9		$C_{22}H_{28}N_2O_5S_2$
Sibopirdine	Nootropic	122955-18-4		$C_{23}H_{18}N_4$
Sibrafiban	Antithrombotic	172927-65-0		$C_{20}H_{28}N_4O_6$
Sibutramine	Antiobesity	106650-56-0		$C_{17}H_{26}ClN$
Siccanin	Antifungal	22733-60-4		$C_{22}H_{30}O_3$
Sifaprazine	Sedative	131635-06-8		$C_{18}H_{22}N_2$
Siguazodan	Cardiotonic	115344-47-3		$C_{14}H_{16}N_6O$
Silandrone	Androgen	5055-42-5		$C_{22}H_{36}O_2Si$
Sildenafil	Erectile Dysfunction	139755-83-2	Y	$C_{22}H_{30}N_6O_4S$
Silibinin	Hepatic Protectant	22888-70-6		$C_{25}H_{22}O_{10}$
Silicristin	Hepatic Protectant	33889-69-9		$C_{25}H_{22}O_{10}$
Silidianin	Hepatic Protectant	29782-68-1		$C_{25}H_{22}O_{10}$
Silodosin	Urologic	160970-54-7	Y	$C_{25}H_{32}F_3$N3O4
Silperisone	Muscle Relaxant	140944-31-6		$C_{15}H_{24}FNSi$
Siltenzepine	Anticonvulsant	98374-54-0		$C_{19}H_{20}ClN_3O_4$
Silver Sulfadiazine	Antibacterial	22199-08-2		$C_{10}H_9AgN_4O_2S$
Simendan	Cardiotonic	131741-08-7		$C_{14}H_{12}N_6O$
Simetride	Analgesic	154-82-5		$C_{28}H_{38}N_2O_6$
Simfibrate	Antihyperlipidemic	14929-11-4		$C_{23}H_{26}Cl_2O_6$
Simotaxel	Antineoplastic	791635-59-1		$C_{46}H_{57}NO_{15}S$
Simtrazene	Antineoplastic	5579-27-1		$C_{14}H_{16}N_4$
Simvastatin	Antihyperlipidemic	79902-63-9	Y	$C_{25}H_{38}O_5$
Sinefungin	Antifungal	58944-73-3		$C_{15}H_{23}N_7O_5$
Sinitrodil	Antihypertensive	143248-63-9		$C_{10}H_{10}N_2O_5$
Sintropium Bromide	Antispasmodic	79467-19-9		C19H36BrNO2
Sipatrigine	Anticonvulsant	130800-90-7		$C_{15}H_{16}Cl_3N_5$
Sipoglitazar	Antidiabetic	342026-92-0		$C_{25}H_{25}N_3O_4S$
Siramesine	Antipsychotic	147817-50-3		$C_{30}H_{31}FN_2O$
Siratiazem	Antihypertensive	138778-28-6		$C_{24}H_{30}N_2O_4S$
Sirolimus	Immunosuppressant	53123-88-9	Y	C51H79NO13
Sisomicin	Antibacterial	32385-11-8		$C_{19}H_{37}N_5O_7$
Sitafloxacin	Antibacterial	127254-12-0	Y	$C_{19}H_{18}ClF_2N_3O_3$
Sitalidone	Diuretic	119636-74-7		$C_{23}H_{29}ClN_2O_5S$
Sitamaquine	Antimalarial	57695-04-2		$C_{21}H_{33}N_3O$
Sitaxentan	Antihypertensive	184036-34-8		$C_{18}H_{15}ClN_2O_6S_2$
Sitofibrate	Antihyperlipidemic	55902-94-8		$C_{39}H_{59}ClO_3$
Sitogluside	Unclassified	474-58-8		$C_{35}H_{60}O_6$
Sitosterol	Steroid	83-46-5		$C_{29}H_{50}O$
Sivelestat	Respiratory Stimulant	127373-66-4		$C_{20}H_{22}N_2O_7S$
Soblidotin	Antineoplastic	149606-27-9		$C_{39}H_{67}N_5O_6$
Sobuzoxane	Antineoplastic	98631-95-9		$C_{22}H_{34}N_4O_{10}$
Cacodylate	Anthelminthic	15132-04-4		$C_2H_6AsO_2$
Dehydroacetate	Choleretic	45990-85-0		$C_8H_7O_4$
Dibunate	Antitussive	14992-58-6		$C_{18}H_{24}O_3S$
Dichloroacetic Acid	Nootropic	79-43-6		$C_2H_2Cl_2O_2$
Sodium Ethasulfate	Respiratory Stimulant	72214-01-8		$C_8H_{18}O_4S$
Sodium Gentisate	Analgesic	490-79-9		C7H6O4
Gluconate	Plasma Volume Expander	608-59-3		$C_6H_{11}O_7$

Table S1. *Cont.*

Common Name	Indication	CAS Number	Oral	Molecular Formula
Glucosulfone	Antibiotic	551-89-3		$C_{24}H_{36}N_2O_{18}S_3$
Gualenate	Antiinflammatory	16915-32-5		$C_{15}H_{18}O_3S$
Hydroxybenzylphosphinate	Unclassified	52705-43-8		$C_7H_9O_3P$
Monofluorophosphate	Dermatologic	13537-32-1		FH_2O_3P
Oxybate	Anesthetic	591-81-1		$C_4H_8O_3$
Phenylacetate	Antidote	7631-42-7		$C_8H_7O_2$
Picofosfate	Unclassified	36175-06-1		$C_{18}H_{17}NO_8P_2$
Picosulfate	Laxative	10040-34-3	Y	$C_{18}H_{15}N_2O_8S_2$
Stibocaptate	Antiprotozoal	1986-66-9		$C_{12}H_{12}O_{12}S_6Sb_2$
Stibogluconate	Antiprotozoal	100817-46-7		$C_{12}H_{20}O_{17}Sb_2$
Succinate	Respiratory Stimulant	56-14-4	Y	$C_4H_4O_4$
Sofalcone	Antiulcerative	64506-49-6		$C_{27}H_{30}O_6$
Solabegron	Antidiabetic	252920-94-8		$C_{23}H_{23}ClN_2O_3$
Solasulfone	Antibiotic	118-84-3		$C_{30}H_{32}N_2O_{14}S_5$
Solifenacin	Antispasmodic	242478-37-1	Y	$C_{23}H_{26}N_2O_2$
Solimastat	Antineoplastic	226072-63-5		$C_{20}H_{32}$N4O5
Solpecainol	Antiarrhythmic	155321-96-3		$C_{18}H_{23}NO_3$
Solypertine Tartrate	Antihypertensive	4448-96-8		$C_{22}H_{25}N_3O_3$
Somantadine	Antiviral	79594-24-4		$C_{14}H_{25}N$
Soneclosan	Antibacterial	3380-30-1		$C_{12}H_8Cl_2O_2$
Sonepiprazole	Antipsychotic	170858-33-0		$C_{21}H_{27}N_3O_3S$
Sopitazine	Antihistaminic	23492-69-5		$C_{20}H_{23}N_3OS$
Sopromidine	Antihistaminic	79313-75-0		$C_{14}H_{23}N_{7S}$
Soquinolol	Antihypertensive	61563-18-6		$C_{17}H_{26}N_2O_3$
Sorafenib	Antineoplastic	284461-73-0	Y	$C_{21}H_{16}ClF_3N_4O_3$
Soraprazan	Antiulcerative	261944-46-1		$C_{21}H_{25}N_3O_3$
Sorbinicate	Antihyperlipidemic	6184-06-1		$C_{42}H_{32}N_{6O12}$
Sorbinil	Antidiabetic	68367-52-2		$C_{11}H_9FN_2O_3$
Soretolide	Anticonvulsant	130403-08-6		$C_{13}H_{14}N_2O_2$
Sorivudine	Antiviral	77181-69-2		$C_{11}H_{13}BrN_2O_6$
Sornidipine	Vasodilator	95105-77-4		$C_{22}H_{24}N_2O_9$
Sotalol	Antianginal	3930-20-9	Y	$C_{12}H_{20}N_2O_3S$
Soterenol	Bronchodilator	13642-52-9		$C_{12}H_{20}N_2O_4S$
Sotirimod	Dermatologic	227318-75-4		$C_{14}H_{17}N_5$
Spaglumic Acid	Antihistaminic	4910-46-7		$C_{11}H_{16}N_2O_8$
Sparfloxacin	Antibiotic	110871-86-8	Y	$C_{19}H_{22}F_2N_4O_3$
Sparfosate Sodium	Antineoplastic	51321-79-0		$C_6H_{10}NO_8P$
Sparsomycin	Antineoplastic	1404-64-4		$C_{13}H_{19}N_3O_5S_2$
Sparteine	Cardiotonic	90-39-1		$C_{15}H_{26}N_2$
Spectinomycin	Antibiotic	1695-77-8		$C_{14}H_{24}N_2O_7$
Spiclamine	Antidepressant	90243-97-3		$C_{20}H_{25}ClN_2O$
Spiclomazine	Antipsychotic	24527-27-3		$C_{22}H_{24}ClN_3OS_2$
Spiperone	Antipsychotic	749-02-0		$C_{23}H_{26}FN_3O_2$
Spiradoline Mesylate	Analgesic	87151-85-7		$C_{22}H_{30}Cl_2N_2O_2$
Spiramide	Nootropic	510-74-7		$C_{22}H_{26}FN_3O_2$
Spirapril	Antihypertensive	83647-97-6	Y	$C_{22}H_{30}N_2O_5S_2$
Spiraprilat	Antihypertensive	83602-05-5		$C_{20}H_{26}N_2O_5S_2$
Spirendolol	Antihypertensive	81840-58-6		$C_{21}H_{31}NO_3$
Spirgetine	Unclassified	144-45-6		$C_{10}H_{20}N_4$
Spirilene	Antipsychotic	357-66-4		$C_{24}H_{28}FN_3O$
Spiriprostil	Prostaglandin	122946-42-3		$C_{20}H_{34}N_2O_4$
Spirobarbital Sodium	Sedative	72035-36-0		$C_{12}H_{20}N_2O_2S$
Spirofylline	Bronchodilator	98204-48-9		$C_{24}H_{28}N_6O_5$
Spirogermanium	Antineoplastic	41992-23-8		$C_{17}H_{36}GeN_2$
Spiroglumide	Antiulcerative	137795-35-8		$C_{21}H_{26}Cl_2N_2O_4$
Spiromustine	Antineoplastic	56605-16-4		$C_{14}H_{23}Cl_2N_3O_2$
Spironolactone	Diuretic	1952-01-7	Y	$C_{24}H_{32}O_4S$
Spirorenone	Steroid	74220-07-8		$C_{24}H_{28}O_3$
Spiroxasone	Diuretic	6673-97-8		$C_{24}H_{34}O_3S$
Spiroxatrine	Vasodilator	1054-88-2		$C_{22}H_{25}N_3O_3$
Spiroxepin	Antidepressant	47254-05-7		$C_{19}H_{21}NO_3$
Spizofurone	Antiulcerative	72492-12-7		$C_{12}H_{10}O_3$
Squalamine Lactate	Antineoplastic	148717-90-2		$C_{34}H_{65}N_3O_5S$

Table S1. *Cont.*

Common Name	Indication	CAS Number	Oral	Molecular Formula
Stacofylline	Bronchodilator	98833-92-2		C20H33N7O3
Stallimycin	Antibacterial	636-47-5		$C_{22}H_{27}N_9O_4$
Stanolone	Androgen	521-18-6		$C_{19}H_{30}O_2$
Stanozolol	Steroid	10418-03-8		$C_{21}H_{32}N_2O$
Stavudine	Antiviral	3056-17-5	Y	$C_{10}H_{12}N_2O_4$
Stearylsulfamide	Diuretic	498-78-2		$C_{24}H_{42}N_2O_3S$
Steffimycin	Antibacterial	11033-34-4		$C_{28}H_{30}O_{13}$
Stenbolone Acetate	Steroid	1242-56-4		$C_{22}H_{32}O_3$
Stepronin	Mucolytic	72324-18-6		$C_{10}H_{11}NO_4S_2$
Stercuronium Iodide	Muscle Relaxant	30033-10-4		$C_{26}H_{43}N_2.I$
Stevaladil	Unclassified	6535-03-1		$C_{27}H_{45}NO_4$
Stilbamidine Isethionate	Antiprotozoal	122-06-5		$C_{16}H_{16}N_4$
Stilbazium Iodide	Anthelminithic	3784-99-4		$C_{31}H_{36}IN_3$
Stilonium Iodide	Antispasmodic	77257-42-2		$C_{22}H_{30}INO$
Stirimazole	Antifungal	30529-16-9		$C_{14}H_{11}N_3O_4$
Stiripentol	Anticonvulsant	49763-96-4	Y	$C_{14}H_{18}O_3$
Stirocainide	Antiarrhythmic	78372-27-7		$C_{22}H_{34}$N2O
Streptomycin Sulfate	Antibiotic	57-92-1		$C_{21}H_{39}N_7O_{12}$
Streptonicozid	Antibacterial	4480-58-4		$C_{27}H_{44}N_{10}O_{12}$
Streptonigrin	Antineoplastic	3930-19-6		$C_{25}H_{22}N_4O_8$
Streptozocin	Antineoplastic	18883-66-4		$C_8H_{15}N_3O_7$
Strinoline	Antiarrhythmic	39862-58-3		$C_{10}H_6N_4$
Strychnine	Nootropic	57-24-9		$C_{21}H_{22}N_2O_2$
Subathizone	Antibacterial	121-55-1		$C_{10}H_{13}N_3O_2S_2$
Subendazole	Anthelminithic	54340-66-8		$C_{10}H_5Cl_3N_4S_3$
Succimer	Antidote	304-55-2		$C_4H_6O_4S_2$
Succinchlorimide	Anticonvulsant	128-09-6		$C_4H_4ClNO_2$
Succinylcholine	Neuromuscular Blocking Agent	306-40-1		$C_{14}H_{30}N_2O_{42+}$
Succinylsulfathiazole	Antibiotic	116-43-8		$C_{13}H_{13}N_3O_5S_2$
Succisulfone	Antibiotic	5934-14-5		$C_{16}H_{16}N_2O_5S$
Suclofenide	Anticonvulsant	30279-49-3		$C_{16}H_{13}ClN_2O_4S$
Sucralose	Antiulcerative	56038-13-2		$C_{12}H_{19}Cl_3O_8$
Sucralox	Unclassified	57-50-1		$C_{12}H_{22}O_{11}$
Sucrosofate Potassium	Antiulcerative	57680-56-5		$C_{12}H_{22}O_{35}S_8$
Sudexanox	Antihistaminic	58761-87-8		$C_{21}H_{23}NO_5S$
Sudoxicam	Antiinflammatory	34042-85-8		$C_{13}H_{11}N_3O_4S_2$
Sufentanil	Analgesic	56030-54-7		$C_{22}H_{30}N_2O_2S$
Sufosfamide	Antineoplastic	37753-10-9		$C_8H_{18}ClN_2O_5PS$
Sufotidine	Antiulcerative	80343-63-1		$C_{20}H_{31}N_5O_3S$
Sufugolix	Pituitary	308831-61-0		$C_{36}H_{31}F_2N_5O_4S$
Sulamserod	Antiarrhythmic	219757-90-1		$C_{19}H_{28}ClN_3O_5S$
Sulazepam	Anxiolytic	2898-13-7		$C_{16}H_{13}ClN_2S$
Sulazuril	Antibiotic	108258-89-5		$C_{17}H_{15}Cl_2N_3O_5S$
Sulbactam Pivoxil	Antibacterial	69388-79-0		$C_{14}H_{21}NO_7S$
Sulbactam	Antibiotic	68373-14-8		$C_8H_{11}NO_5S$
Sulbenicillin	Antibiotic	41744-40-5		$C_{16}H_{18}N_2O_7S_2$
Sulbenox	Steroid	58095-31-1		$C_9H_{10}N_2O_2S$
Sulbentine	Antifungal	350-12-9		$C_{17}H_{18}N_2S_2$
Sulclamide	Antitussive	2455-92-7		$C_7H_7ClN_2O_3S$
Sulconazole	Antifungal	61318-90-9		$C_{18}H_{15}Cl_3N_2S$
Sulfabenz	Antibacterial	127-77-5		$C_{12}H_{12}$N2O2S
Sulfabenzamide	Antibacterial	127-71-9		$C_{13}H_{12}N_2O_3S$
Sulfabromomethazine Sodium	Antibiotic	116-45-0		$C_{12}H_{13}BrN_4O_2S$
Sulfacarbamide	Antibiotic	547-44-4		$C_7H_9N_3O_3S$
Sulfacecole	Unclassified	21662-79-3		$C_{14}H_{17}N_3O_5S$
Sulfacetamide	Antibiotic	144-80-9		$C_8H_{10}N_2O_3S$
Sulfachlorpyridazine	Antibiotic	80-32-0		$C_{10}H_9ClN_4O_2S$
Sulfachrysoidine	Antibiotic	485-41-6		$C_{13}H_{13}N_5O_4S$
Sulfaclomide	Antibacterial	4015-18-3		$C_{12}H_{13}ClN_4O_2S$
Sulfaclorazole	Unclassified	54063-55-7		$C_{16}H_{15}ClN_4O_2S$
Sulfaclozine	Antibiotic	102-65-8		$C_{10}H_9ClN_4O_2S$
Sulfacytine	Antibacterial	17784-12-2		$C_{12}H_{14}N_4O_3S$

Table S1. *Cont.*

Common Name	Indication	CAS Number	Oral	Molecular Formula
Sulfadiazine	Antibiotic	68-35-9		$C_{10}H_{10}N_4O_2S$
Sulfadicramide	Antibiotic	115-68-4		$C_{11}H_{14}N_2O_3S$
Sulfadimethoxine	Antibiotic	122-11-2		$C_{12}H_{14}N_4O_4S$
Sulfadoxine	Antibiotic	2447-57-6		$C_{12}H_{14}N_4O_4S$
Sulfaethidole	Antibiotic	94-19-9		$C_{10}H_{12}N_4O_2S_2$
Sulfaguanidine	Antibiotic	57-67-0		$C_7H_{10}N_4O_2S$
Sulfaguanole	Antibiotic	27031-08-9		$C_{12}H_{15}N_5O_3S$
Sulfalene	Antibacterial	152-47-6		$C_{11}H_{12}N_4O_3S$
Sulfaloxic Acid	Antibiotic	14376-16-0		$C_{16}H_{15}N_3O_7S$
Sulfamazone	Antiinflammatory	65761-24-2		$C_{23}H_{24}N_6O_7S_2$
Sulfamerazine	Antibiotic	127-79-7		$C_{11}H_{12}N_4O_2S$
Sulfameter	Antibiotic	651-06-9		$C_{11}H_{12}N_4O_3S$
Sulfamethazine	Antibiotic	57-68-1		$C_{12}H_{14}N_4O_2S$
Sulfamethizole	Antibiotic	144-82-1		$C_9H_{10}N_4O_2S_2$
Sulfamethoxazole	Antibiotic	723-46-6		$C_{10}H_{11}N_3O_3S$
Sulfamethoxypyridazine	Antibiotic	80-35-3		$C_{11}H_{12}N_4O_3S$
Sulfamethoxypyridazine Acetyl	Antibiotic	3568-43-2		$C_{13}H_{14}N_4O_4S$
Sulfametomidine	Antibiotic	3772-76-7		$C_{12}H_{14}N_4O_3S$
Sulfametrole	Antibiotic	32909-92-5		$C_9H_{10}N_4O_3S_2$
Sulfamonomethoxine	Antibiotic	1220-83-3		$C_{11}H_{12}N_4O_3S$
Sulfamoxole	Antibacterial	729-99-7		$C_{11}H_{13}N_3O_3S$
Sulfanilamide	Antibiotic	63-74-1		$C_6H_8N_2O2S$
Sulfanilate Zinc	Antibiotic	121-57-3		$C_6H_7NO_3S$
Sulfanitran	Antibacterial	122-16-7		$C_{14}H_{13}N_3O_5S$
Sulfaperin	Antibiotic	599-88-2		$C_{11}H_{12}N_4O_2S$
Sulfaphenazole	Antibiotic	526-08-9		$C_{15}H_{14}N_4O_2S$
Sulfaproxyline	Antibiotic	116-42-7		$C_{16}H18N2O4S$
Sulfapyrazine	Antibiotic	116-44-9		$C_{10}H_{10}N_4O_2S$
Sulfapyridine	Antibiotic	144-83-2		$C_{11}H_{11}N_3O_2S$
Sulfaquinoxaline	Antiprotozoal	59-40-5		$C_{14}H_{12}N_4O_2S$
Sulfarsphenamine	Antibacterial	534-93-0		$C_{14}H_{16}As_2N_2O_8S_2$
Sulfasalazine	Antiinflammatory	599-79-1	Y	$C_{18}H_{14}N_4O_5S$
Sulfasomizole	Antibacterial	632-00-8		$C_{10}H_{11}N_3O_2S_2$
Sulfasuccinamide	Antibiotic	3563-14-2		$C_{10}H_{12}N_2O_5S$
Sulfasymazine	Antibiotic	1984-94-7		$C_{13}H_{17}N_5O_2S$
Sulfathiazole	Antibiotic	72-14-0		$C_9H_9N_3O_2S_2$
Sulfathiourea	Antibiotic	515-49-1		$C_7H_9N_3O_2S_2$
Sulfatroxazole	Antibacterial	23256-23-7		$C_{11}H_{13}N_3O_3S$
Sulfatrozole	Antiprotozoal	13369-07-8		$C_{10}H_{12}N_4O_3S_2$
Sulfazamet	Antibacterial	852-19-7		$C_{16}H_{16}N_4O_2S$
Sulfinalol	Antihypertensive	66264-77-5		$C_{20}H_{27}NO_4S$
Sulfinpyrazone	Antiurolithic	57-96-5	Y	$C_{23}H_{20}N_2O_3S$
Sulfiram	Ectoparasiticide	95-05-6		$C_{10}H_{20}N_2S_3$
Sulfisomidine	Antibiotic	515-64-0		$C_{12}H_{14}N_4O_2S$
Sulfisoxazole	Antibiotic	127-69-5	Y	$C_{11}H_{13}N_3O_3S$
Sulfisoxazole Acetyl	Antibacterial	80-74-0		$C_{13}H_{15}N_3O_4S$
Sulfonethylmethane	Sedative	76-20-0		$C_8H_{18}O_4S_2$
Sulfonmethane	Sedative	115-24-2		$C_7H_{16}O_4S_2$
Sulfonterol	Bronchodilator	42461-79-0		$C_{14}H_{23}NO_4S$
Sulforidazine	Antipsychotic	14759-06-9		$C_{21}H_{26}N_2O_2S_2$
Sulfosalicylic Acid	Antiinflammatory	97-05-2		$C_7H_6O_6S$
Sulfoxone	Antibiotic	144-76-3		$C_{14}H_{16}N_2O_6S_3$
Sulicrinat	Diuretic	90207-12-8		$C_{15}H_{10}Cl_3NO_6S$
Sulindac	Antineoplastic	38194-50-2	Y	$C_{20}H_{17}FO_3S$
Sulisatin	Laxative	54935-03-4		$C_{21}H_{17}NO_9S_2$
Sulisobenzone	Dermatologic	4065-45-6		$C_{14}H_{12}O_6S$
Sulmarin	Hemostatic	29334-07-4		$C_{10}H_8O_{10}S_2$
Sulmazole	Cardiotonic	73384-60-8		$C_{14}H_{13}N_3O_2S$
Sulmepride	Gastroprokinetic	57479-88-6		$C_{14}H_{21}N_3O_4S$
Sulnidazole	Antiprotozoal	51022-76-5		$C_9H_{14}N_4O_3S$
Sulocarbilate	Opthalmic	121-64-2		$C_9H_{12}N_2O_5S$
Suloctidil	Vasodilator	54767-75-8		$C_{20}H_{35}NOS$
Sulofenur	Antineoplastic	110311-27-8		$C_{16}H_{15}ClN_2O_3S$

Table S1. *Cont.*

Common Name	Indication	CAS Number	Oral	Molecular Formula
Sulopenem	Antibacterial	120788-07-0		$C_{12}H_{15}NO_5S_3$
Sulosemide	Diuretic	82666-62-4		$C_{17}H_{16}N_2O_7S_2$
Sulotroban	Anticoagulant	72131-33-0		$C_{16}H_{17}NO_5S$
Suloxifen Oxalate	Bronchodilator	25827-12-7		$C_{18}H_{24}N_2OS$
Sulpiride	Antipsychotic	15676-16-1	Y	$C_{15}H_{23}N_3O_4S$
Sulprosal	Unclassified	58703-77-8		$C_{10}H_{12}O_6S$
Sulprostone	Prostaglandin	60325-46-4		$C_{23}H_{31}NO_7S$
Sultamicillin	Antibacterial	76497-13-7		$C_{25}H_{30}N_4O_9S_2$
Sulthiame	Anticonvulsant	61-56-3		$C_{10}H_{14}N_2O_4S_2$
Sultopride	Antipsychotic	53583-79-2	Y	$C_{17}H_{26}N_2O_4S$
Sultosilic Acid	Antihyperlipidemic	57775-26-5		$C_{13}H_{12}O_7S_2$
Sultroponium	Antispasmodic	15130-91-3		$C_{20}H_{29}NO_6S$
Sulukast	Bronchodilator	98116-53-1		$C_{25}H_{36}N_4O_3S$
Sulverapride	Antiemetic	73747-20-3		$C_{16}H_{25}N_3O_5S$
Sumacetamol	Analgesic	69217-67-0		$C_{15}H_{20}N_2O_4S$
Sumanirole	Antiparkinsonian	179386-43-7		$C_{11}H_{13}N_3O$
Sumarotene	Dermatologic	105687-93-2		$C_{24}H_{30}O_2S$
Sumatriptan	Antimigraine	103628-46-2		$C_{14}H_{21}N_3O_2S$
Sumetizide	Antidiabetic	32059-27-1		C_{12}H13ClN4O6S2
Sunagrel	Anticoagulant	85418-85-5		$C_{25}H_{32}N_2O_2S$
Suncillin Sodium	Antibacterial	22164-94-9		$C_{16}H_{19}N_3O_7S_2$
Sunepitron	Antidepressant	131831-03-3		$C_{17}H_{24}ClN_5O_2$
Sunitinib	Antineoplastic	557795-19-4	Y	$C_{22}H_{27}FN_4O_2$
Supidimide	Sedative	49785-74-2		$C_{12}H_{12}N_2O_4S$
Suplatast Tosilate	Antihistaminic	94055-76-2	Y	$C_{23}H_{33}NO_7S_2$
Suproclone	Sedative	76535-71-2		$C_{22}H_{22}ClN_5O_4S_2$
Suprofen	Antiinflammatory	40828-46-4		$C_{14}H_{12}O_3S$
Suricainide Maleate	Antiarrhythmic	85053-46-9		$C_{18}H_{31}N_3O_3S$
Suriclone	Anxiolytic	53813-83-5		$C_{20}H_{20}ClN_5O_3S_2$
Surinabant	Antibiotic	288104-79-0		$C_{23}H_{23}BrCl_2N_4O$
Suritozole	Antidepressant	110623-33-1		$C_{10}H_{10}FN_3S$
Suronacrine Maleate	Neuromuscular Blocking Agent	104675-35-6		$C_{20}H_{20}N_2O$
Susalimod	Immunomodulator	149556-49-0		$C_{21}H_{16}N_2O_5S$
Suxamethonium Bromide	Muscle Relaxant	55-94-7		$C_{14}H_{30}Br_2N_2O_4$
Suxemerid Sulfate	Antitussive	47662-15-7		$C_{24}H_{44}N_2O_4$
Suxethonium Chloride	Neuromuscular Blocking Agent	54063-57-9		$C_{16}H_{34}Cl_2N_2O_4$
Suxibuzone	Antiinflammatory	27470-51-5		$C_{24}H_{26}N_2O_6$
Symclosene	Antibacterial	87-90-1		$C_3Cl_3N_3O_3$
Symetine	Antiamebic	15599-45-8		$C_{30}H_{48}N_2O_2$
Syrosingopine	Antihypertensive	84-36-6		$C_{35}H_{42}N_2O_{11}$
Tabilautide	Immunomodulator	78088-46-7		$C_{27}H_{49}N_5O_8$
Tabimorelin	Cardiotonic	193079-69-5		$C_{32}H_{40}N_4O_3$
Tacalcitol	Dermatologic	57333-96-7		$C_{27}H_{44}O_3$
Tacapenem	Antibiotic	193811-33-5		$C_{14}H_{18}N_2O_5S$
Tacedinaline	Antineoplastic	112522-64-2		$C_{15}H_{15}N_3O_2$
Taclamine	Anxiolytic	34061-33-1		$C_{21}H_{23}N$
Tacrine	Nootropic	321-64-2	Y	$C_{13}H_{14}N_2$
Tacrolimus	Immunosuppressant	104987-11-3	Y	$C_{44}H_{69}NO_{12}$
Tadalafil	Erectile Dysfunction	171596-29-5	Y	$C_{22}H_{19}N_3O_4$
Tafenoquine	Antimalarial	106635-80-7		$C_{24}H_{28}F_3N_3O_3$
Tafluprost	Prostaglandin	209860-87-7		$C_{25}H_{34}F_2O_5$
Taglutimide	Sedative	14166-26-8		$C_{14}H_{16}N_2O_4$
Tagorizine	Antihistaminic	118420-47-6		$C_{30}H_{36}N_4O$
Talabostat	Hematinic	149682-77-9		$C_9H_{19}BN_2O_3$
Talaglumetad	Antineoplastic	441765-98-6		$C_{11}H_{16}N_2O_5$
Talampanel	Antiseizure	161832-65-1		$C_{19}H_{19}N_3O_3$
Talampicillin	Antibacterial	47747-56-8		$C_{24}H_{23}N_3O_6S$
Talaporfin Sodium	Antineoplastic	110230-98-3		$C_{38}H_{41}N_5O_9$
Talastine	Antihistaminic	16188-61-7		$C_{19}H_{21}N_3O$
Talbutal	Sedative	115-44-6		$C_{11}H_{16}N_2O_3$
Taleranol	Pituitary	42422-68-4		$C_{18}H_{26}O_5$

Table S1. *Cont.*

Common Name	Indication	CAS Number	Oral	Molecular Formula
Talibegron	Antiobesity	146376-58-1		$C_{18}H_{21}NO_4$
Talinolol	Antihypertensive	57460-41-0	Y	$C_{20}H_{33}N_3O_3$
Talipexole	Antiparkinsonian	101626-70-4	Y	$C_{10}H_{15}N_3S$
Tallimustine	Antineoplastic	115308-98-0		$C_{32}H_{38}Cl_2N_{10}O_4$
Talmetacin	Analgesic	67489-39-8		$C_{27}H_{20}ClNO_6$
Talmetoprim	Antibiotic	66093-35-4		$C_{22}H_{20}N_4O_5$
Talnetant	Antispasmodic	174636-32-9		$C_{25}H_{22}N_2O_2$
Talniflumate	Antiinflammatory	66898-62-2	Y	$C_{21}H_{13}F_3N_2O_4$
Talopram	Antidepressant	7182-51-6		$C_{20}H_{25}NO$
Talosalate	Analgesic	66898-60-0		$C_{17}H_{12}O_6$
Talotrexin Ammonium	Antineoplastic	113857-87-7		$C_{27}H_{27}N_9O_6$
Taloximine	Bronchodilator	17243-68-4		$C_{12}H_{16}N_4O_2$
Talsaclidine Fumarate	Nootropic	147025-53-4		$C_{10}H_{15}NO$
Talsupram	Antidepressant	21489-20-3	Y	$C_{20}H_{25}NS$
Taltirelin	Pituitary	103300-74-9	Y	$C_{17}H_{23}N_7O_5$
Taltobulin	Antineoplastic	228266-40-8		$C_{27}H_{43}N_3O_4$
Taltrimide	Anticonvulsant	81428-04-8		$C_{13}H_{16}N_2O_4S$
Talviraline	Antiviral	163451-80-7		$C_{15}H_{20}N_2O_3S_2$
Tameridone	Sedative	102144-78-5		$C_{22}H_{26}N_6O_2$
Tameticillin	Antibacterial	56211-43-9		$C_{23}H_{33}N_3O_6S$
Tametraline	Antidepressant	52795-02-5		$C_{17}H_{19}N$
Tamibarotene	Immunomodulator	94497-51-5	Y	$C_{22}H_{25}NO_3$
Tamitinol	Nootropic	59429-50-4		$C_{11}H_{18}N_2OS$
Tamolarizine	Vasodilator	93035-32-6		$C_{27}H_{32}N_2O_3$
Tamoxifen	Antineoplastic	10540-29-1	Y	$C_{26}H_{29}NO$
Tampramine Fumarate	Antidepressant	83166-17-0		C_{23}H24N4
Tamsulosin	Antineoplastic	106133-20-4	Y	$C_{20}H_{28}N_2O_5S$
Tanaproget	Contraceptive	304853-42-7		$C_{16}H_{15}N_3OS$
Tandamine	Antidepressant	42408-80-0		$C_{18}H_{26}N_2S$
Tandospirone Citrate	Anxiolytic	87760-53-0	Y	$C_{21}H_{29}N_5O_2$
Tandutinib	Antineoplastic	387867-13-2		$C_{31}H_{42}N_6O_4$
Taniplon	Sedative	106073-01-2		$C_{14}H_{15}N_5$O2
Tanogitran	Unclassified	637328-69-9		$C_{25}H_{31}N_7O_3$
Tanomastat	Antirheumatic	179545-77-8		$C_{23}H_{19}ClO_3S$
Tapentadol	Analgesic	175591-23-8	Y	$C_{14}H_{23}NO$
Taprizosin	Antihypertensive	210538-44-6		$C_{25}H_{26}N_6O_4S$
Taprostene	Antithrombotic	108945-35-3		$C_{24}H_{30}O_5$
Tarazepide	Antispasmodic	141374-81-4		$C_{28}H_{24}N_4O_2$
Tariquidar	Antineoplastic	206873-63-4		$C_{38}H_{38}N_4O_6$
Tartaric Acid	Antibacterial	87-69-4		$C_4H_6O_6$
Tasidotin	Antineoplastic	192658-64-3		$C_{32}H_{58}N_6O_5$
Tasosartan	Antihypertensive	145733-36-4		$C_{23}H_{21}N_7O$
Tasquinimod	Nootropic	254964-60-8	Y	$C_{20}H_{17}F_3N_2O_4$
Tasuldine	Mucolytic	88579-39-9		$C_{10}H_9N_3S$
Taurocholic Acid	Choleretic	81-24-3		$C_{26}H_{45}NO_7S$
Taurolidine	Antibacterial	19388-87-5		$C_7H_{16}N_4O_4S_2$
Tauromustine	Antineoplastic	85977-49-7		$C_7H_{15}ClN_4O_4S$
Tauroselcholic Acid	Gastroprokinetic	75018-71-2		$C_{26}H_{45}$NO7SSe
Taurosteine	Mucolytic	124066-33-7		$C_7H_9NO_4S_2$
Taurultam	Antineoplastic	38668-01-8		$C_3H_8N_2O_2S$
Tazadolene Succinate	Analgesic	87936-75-2		$C_{16}H_{21}N$
Tazanolast	Antihistaminic	82989-25-1		$C_{13}H_{15}N_5O_3$
Tazarotene	Dermatologic	118292-40-3		$C_{21}H_{21}NO_2S$
Tazasubrate	Antihyperlipidemic	79071-15-1		$C_{18}H_{17}NO_3S_2$
Tazeprofen	Antiinflammatory	76816-33-6		$C_{16}H_{13}NO_2S$
Tazifylline	Antihistaminic	79712-55-3		$C_{23}H_{32}N_6O_3S$
Taziprinone	Cardiotonic	79253-92-2		$C_{22}H_{31}N_3O_3$
Tazobactam	Antibiotic	89786-04-9		$C_{10}H_{12}N_4O_5S$
Tazofelone	Immunomodulator	107902-67-0		$C_{18}H_{27}NO_2S$
Tazolol	Cardiotonic	39832-48-9		$C_9H_{16}N_2O_2S$
Tazomeline Citrate	Nootropic	131987-54-7		$C_{14}H_{23}N_3S_2$
Tebanicline Tosylate	Analgesic	198283-73-7		$C_9H_{11}ClN_2O$
Tebatizole	Antiurolithic	54147-28-3		$C_{12}H_{21}N_3S$

Table S1. *Cont.*

Common Name	Indication	CAS Number	Oral	Molecular Formula
Tebipenem Pivoxil	Antibiotic	161715-24-8	Y	$C_{22}H_{31}N_3O_6S_2$
Tebufelone	Analgesic	112018-00-5		$C_{20}H_{28}O_2$
Tebuquine	Antimalarial	74129-03-6		$C_{26}H_{25}Cl_2N_3O$
Tecadenoson	Antiarrhythmic	204512-90-3		$C_{14}H_{19}N_5O_5$
Tecalcet	Pituitary	148717-54-8		$C_{18}H_{22}ClNO$
Tecastemizole	Antihistaminic	75970-99-9		$C_{19}H_{21}FN_4$
Teclothiazide	Diuretic	4267-05-4		$C_8H_7Cl_4N_3O_4S_2$
Teclozan	Antiamebic	5560-78-1		$C_{20}H_{28}Cl_4N_2O_4$
Tedisamil	Antiarrhythmic	90961-53-8		$C_{19}H_{32}N_2$
Tefazoline	Adrenergic	1082-56-0		$C_{14}H_{18}N_2$
Tefenperate	Unclassified	77342-26-8		$C_{29}H_{37}Cl_2NO_4$
Tefludazine	Antipsychotic	80273-79-6		$C_{22}H_{24}F_4N_2O$
Teflurane	Anesthetic	124-72-1		C_2HBrF_4
Teflutixol	Antipsychotic	55837-23-5		$C_{23}H_{26}F_4N_2OS$
Tegafur	Antineoplastic	17902-23-7	Y	$C_8H_9FN_2O_3$
Tegaserod	Gastroprokinetic	145158-71-0	Y	$C_{16}H_{23}N_5O$
Teglicar	Unclassified	250694-07-6		$C_{22}H_{45}N_3O_3$
Telbivudine	Antiviral	3424-98-4	Y	$C_{10}H_{14}N_2O_5$
Telenzepine	Antiulcerative	80880-90-6		$C_{19}H_{22}N_4O_2S$
Telinavir	Antiviral	143224-34-4		$C_{33}H_{44}N_6O_5$
Telithromycin	Antibiotic	191114-48-4	Y	$C_{43}H_{65}N_5O_{10}$
Telmesteine	Mucolytic	122946-43-4		$C_7H_{11}NO_4S$
Telmisartan	Antihypertensive	144701-48-4	Y	$C_{33}H_{30}N_4O_2$
Teloxantrone	Antineoplastic	91441-48-4		$C_{21}H_{25}N_5O_4$
Teludipine	Antihypertensive	108687-08-7		$C_{28}H_{38}N_2O_6$
Temafloxacin	Antibacterial	108319-06-8		$C_{21}H_{18}F_3N_3O_3$
Temarotene	Immunomodulator	75078-91-0		$C_{23}H_{28}$
Temazepam	Sedative	846-50-4	Y	$C_{16}H_{13}ClN_2O_2$
Temefos	Ectoparasiticide	3383-96-8	Y	$C_{16}H_{20}O_6P_2S_3$
Temelastine	Antihistaminic	86181-42-2		$C_{21}H_{24}BrN_5O$
Temiverine	Antispasmodic	173324-94-2		$C_{24}H_{35}NO_3$
Temocapril	Antihypertensive	111902-57-9	Y	$C_{23}H_{28}N_2O_5S_2$
Temocaprilat	Antihypertensive	110221-53-9		$C_{21}H_{24}N_2O_5S_2$
Temocillin	Antibacterial	66148-78-5		$C_{16}H_{18}N_2O_7S_2$
Temodox	Steroid	34499-96-2		$C_{12}H_{12}N_2O_5$
Temoporfin	Antineoplastic	122341-38-2		$C_{44}H_{32}N_4O_4$
Temozolomide	Antineoplastic	85622-93-1	Y	$C_6H_6N_6O_2$
Tenamfetamine	Nootropic	4764-17-4		$C_{10}H_{13}NO_2$
Tenatoprazole	Antihypertensive	113712-98-4		$C_{16}H_{18}N_4O_3S$
Tenidap	Antiinflammatory	120210-48-2		$C_{14}H_9ClN_2O_3S$
Tenilapine	Antipsychotic	82650-83-7		$C_{17}H_{16}N_4S_2$
Teniloxazine	Antidepressant	62473-79-4		$C_{16}H_{19}NO_2S$
Tenilsetam	Nootropic	86696-86-8		$C_8H_{10}N_2OS$
Teniposide	Antineoplastic	29767-20-2	Y	$C_{32}H_{32}O_{13}S$
Tenivastatin Calcium	Antihyperlipidemic	121009-77-6		$C_{25}H_{40}O_6$
Tenocyclidine	Antiparkinsonian	21500-98-1		$C_{15}H_{23}NS$
Tenofovir	Antiviral	147127-20-6	Y	$C_9H_{14}N_5O_4P$
Tenofovir Disoproxil Fumarate	Antiviral	201341-05-1	Y	$C_{19}H_{30}N_5O_{10}P$
Tenonitrozole	Antiprotozoal	3810-35-3		$C_8H_5N_3O_3S_2$
Tenosal	Unclassified	95232-68-1		$C_{12}H_8O_4S$
Tenosiprol	Antiinflammatory	129336-81-8		$C_{10}H_{11}NO_4S$
Tenoxicam	Antiinflammatory	59804-37-4	Y	$C_{13}H_{11}N_3O_4S_2$
Tenylidone	Diuretic	893-01-6		$C_{16}H_{14}OS_2$
Teopranitol	Vasodilator	81792-35-0		$C_{16}H_{22}N_6O_7$
Teoprolol	Antihypertensive	65184-10-3		$C_{23}H_{30}N_6O_4$
Tepirindole	Nootropic	72808-81-2		$C_{16}H_{19}ClN_2$
Tepoxalin	Dermatologic	103475-41-8		$C_{20}H_{20}ClN_3O3$
Teprenone	Antiulcerative	6809-52-5	Y	$C_{23}H_{38}O$
Terazosin	Antihypertensive	63590-64-7	Y	$C_{19}H_{25}N_5O_4$
Terbequinil	Antipsychotic	113079-82-6		$C_{15}H_{18}N_2O_3$
Terbinafine	Antifungal	91161-71-6	Y	$C_{21}H_{25}N$
Terbogrel	Antithrombotic	149979-74-8		$C_{23}H_{27}N_5O_2$
Terbucromil	Antihistaminic	37456-21-6		$C_{18}H_{22}O_4$

Table S1. *Cont.*

Common Name	Indication	CAS Number	Oral	Molecular Formula
Terbufibrol	Antihyperlipidemic	56488-59-6		$C_{20}H_{24}O_5$
Terbuficin	Unclassified	15534-92-6		$C_{30}H_{44}O_4$
Terbuprol	Choleretic	13021-53-9		$C_8H_{18}O_3$
Terbutaline	Bronchodilator	23031-25-6	Y	$C_{12}H_{19}NO_3$
Terciprazine	Antipsychotic	56693-15-3		$C_{22}H_{29}F_3N_2O_2$
Terconazole	Antifungal	67915-31-5		$C_{26}H_{31}Cl_2N_5O_3$
Terdecamycin	Antibacterial	113167-61-6		$C_{31}H_{43}N_3O_8$
Terestigmine	Muscle Relaxant	147650-57-5		C_{21}H33N3O3
Terfenadine	Antihistaminic	50679-08-8		$C_{32}H_{41}$NO2
Terflavoxate	Muscle Relaxant	86433-40-1		$C_{26}H_{29}NO_4$
Terfluranol	Steroid	64396-09-4		$C_{17}H_{17}F_3O_2$
Terguride	Antiparkinsonian	37686-84-3	Y	$C_{20}H_{28}N_4O$
Teriflunomide	Antirheumatic	163451-81-8	Y	$C_{12}H_9F_3N_2O_2$
Terikalant	Antiarrhythmic	132338-79-5		$C_{24}H_{31}NO_3$
Terizidone	Antibacterial	25683-71-0		$C_{14}H_{14}N_4O_4$
Terlakiren	Antihypertensive	119625-78-4		$C_{31}H_{48}N_4O_7S$
Ternidazole	Antiprotozoal	1077-93-6		$C_7H_{11}N_3O_3$
Terodiline	Vasodilator	15793-40-5		$C_{20}H_{27}N$
Terofenamate	Antiinflammatory	29098-15-5		$C_{17}H_{17}Cl_2NO_3$
Teroxalene	Unclassified	14728-33-7		$C_{28}H_{41}ClN_2O$
Teroxirone	Antineoplastic	59653-73-5		$C_{12}H_{15}N_3O_6$
Terpin	Expectorant	80-53-5		$C_{10}H_{20}O_2$
Tertatolol	Antihypertensive	83688-84-0	Y	$C_{16}H_{25}NO_2S$
Terutroban	Antithrombotic	165538-40-9	Y	$C_{20}H_{22}ClNO_4S$
Tesaglitazar	Antidiabetic	251565-85-2		$C_{20}H_{24}O_7S$
Tesetaxel	Antineoplastic	333754-36-2		$C_{46}H_{60}FN_3O_{13}$
Tesicam	Antiinflammatory	21925-88-2		$C_{16}H_{11}ClN_2O_3$
Tesimide	Antiinflammatory	35423-09-7		$C_{16}H_{15}NO_2$
Tesmilifene	Antihistaminic	98774-23-3		$C_{19}H_{25}NO$
Tesofensine	Antiparkinsonian	195875-84-4		$C_{17}H_{23}Cl_2NO$
Testolactone	Antineoplastic	968-93-4		$C_{19}H_{24}O_3$
Testosterone	Androgen	58-22-0		$C_{19}H_{28}O_2$
Tetomilast	Bronchodilator	145739-56-6		$C_{19}H_{18}N_2O_4S$
Tetrabarbital	Sedative	76-23-3		$C_{12}H_{20}N_2O_3$
Tetrabenazine	Antipsychotic	58-46-8	Y	$C_{19}H_{27}NO_3$
Tetracaine	Anesthetic	94-24-6		$C_{15}H_{24}N_2O_2$
Tetrachloroethylene	Anthelminithic	127-18-4		C_2Cl_4
Tetracycline	Antibiotic	60-54-8		$C_{22}H_{24}N_2O_8$
Tetradonium Bromide	Antiarrhythmic	1119-97-7		$C_{17}H_{38}BrN$
Tetragastrin	Antispasmodic	1947-37-1		$C_{29}H_{36}N_6O_6S$
Tetrahydrozoline	Decongestant	84-22-0		$C_{13}H_{16}N_2$
Tetramisole	Anthelminithic	5036-02-2		$C_{11}H_{12}N_2S$
Tetrantoin	Anticonvulsant	52094-70-9		$C_{12}H_{12}N_2O_2$
Tetraxetan	Antidote	60239-18-1		$C_{16}H_{28}N_4O_8$
Tetrazepam	Muscle Relaxant	10379-14-3		$C_{16}H_{17}ClN_2O$
Tetrazolast Meglumine	Antihistaminic	95104-27-1		$C_{10}H_6N_8$
Tetriprofen	Analgesic	28168-10-7		$C_{15}H_{18}O_2$
Tetronasin 5930	Antibiotic	75139-06-9		$C_{35}H_{54}O_8$
Tetroquinone	Dermatologic	319-89-1		$C_6H_4O_6$
Tetroxoprim	Antibacterial	53808-87-0		$C_{16}H_{22}N_4O_4$
Tetrydamine	Analgesic	17289-49-5		$C_9H_{15}N_3$
Texacromil	Antihistaminic	77005-28-8		$C_{14}H_{14}O_6S$
Tezacitabine	Antineoplastic	130306-02-4		C10H12FN3O4
Tezosentan	Anesthetic	180384-57-0		$C_{27}H_{27}N_9O_6S$
Thalidomide	Immunomodulator	50-35-1	Y	$C_{13}H_{10}N_2O_4$
Thebacon	Analgesic	466-90-0		$C_{20}H_{23}NO_4$
Thenalidine	Antihistaminic	86-12-4		$C_{17}H_{22}N_2S$
Thenium Closylate	Anthelminithic	4304-40-9		$C_{21}H_{24}ClNO_4S_2$
Thenyldiamine	Antihistaminic	91-79-2		$C_{14}H_{19}N_3S$
Theobromine	Diuretic	83-67-0		$C_7H_8N_4O_2$
Theodrenaline	Bronchodilator	13460-98-5		$C_{17}H_{21}N_5O_5$
Theofibrate	Antihyperlipidemic	54504-70-0		$C_{19}H_{21}ClN_4O_5$
Theophylline	Bronchodilator	58-55-9	Y	$C_7H_8N_4O_2$

Table S1. *Cont.*

Common Name	Indication	CAS Number	Oral	Molecular Formula
Thiabendazole	Anthelminithic	148-79-8		$C_{10}H_7N_{3S}$
Thiacetarsamide	Anthelminthic	531-72-6		$C_{11}H_{12}AsNO_5S_2$
Thialbarbital	Anesthetic	467-36-7		$C_{13}H_{16}$N2O2S
Thiambutosine	Antibiotic	500-89-0		$C_{19}H_{25}N_3OS$
Thiamiprine	Antineoplastic	5581-52-2		$C_9H_8N_8O_2S$
Thiamphenicol	Antibiotic	15318-45-3		$C_{12}H_{15}Cl_2NO_5S$
Thiamylal	Anesthetic	77-27-0		$C_{12}H_{18}N_2O_2S$
Thiazesim	Antidepressant	5845-26-1		$C_{19}H_{22}N_2OS$
Thiazinamium Chloride	Antihistaminic	4320-13-2		$C_{18}H_{23}ClN_2S$
Thiazinamium Metilsulfate	Antihistaminic	58-34-4		$C_{19}H_{26}N_2O_4S_2$
Thiazolsulfone	Antibiotic	473-30-3		$C_9H_9N_3O_2S_2$
Thiethylperazine	Antiemetic	1420-55-9		C22H29N3S2
Thiocarbanidin	Antibiotic	92-97-7		$C_{22}H_{23}N_3OS$
Thiocarbarsone	Antiamebic	120-02-5		$C_{11}H_{13}AsN_2O_5S_2$
Thiocolchicoside	Muscle Relaxant	602-41-5	Y	$C_{27}H_{33}NO_{10}S$
Thioctic Acid	Hepatic Protectant	1077-28-7		$C_8H_{14}O_2S_2$
Thioctic Acid Amide	Unclassified	940-69-2		$C_8H_{15}NOS_2$
Thiodiglycol	Antineoplastic	111-48-8		$C_4H_{10}O_2S$
Thiofuradene	Antibacterial	2240-21-3		$C_8H_8N_4O_3S$
Thioguanine	Antineoplastic	154-42-7	Y	$C_5H_5N_5S$
Thiohexamide	Antidiabetic	3692-44-2		$C_{14}H_{20}N_2O_3S_2$
Thioinosine	Immunosuppressant	574-25-4		$C_{10}H_{12}N_4O_4S$
Thiopental	Anesthetic	76-75-5		$C_{11}H_{18}N_2O_2S$
Thiopropazate	Antipsychotic	84-06-0		$C_{23}H_{28}ClN_3O_2S$
Thioproperazine	Antipsychotic	316-81-4		$C_{22}H_{30}N_4O_2S_2$
Thioridazine	Antipsychotic	50-52-2	Y	$C_{21}H_{26}N_2S_2$
Thiotepa	Antineoplastic	52-24-4		$C_6H_{12}N_3PS$
Thiotetrabarbital	Sedative	467-38-9		$C_{12}H_{20}N_2O_2S$
Thiothixene	Antipsychotic	3313-26-6	Y	$C_{23}H_{29}N_3O_2S_2$
Thiouracil	Thyroid	141-90-2		$C_4H_4N_2OS$
Thiourea	Antineoplastic	62-56-6		CH_4N_2S
Thiphenamil	Muscle Relaxant	82-99-5		$C_{20}H_{25}NOS$
Thiphencillin Potassium	Antibacterial	26552-51-2		$C_{16}H_{17}KN_2O_4S_2$
Thonzylamine	Antihistaminic	91-85-0		$C_{16}H_{22}N_4O$
Thozalinone	Antidepressant	655-05-0		$C_{11}H_{12}N_2O_2$
Thurfyl Nicotinate	Antiinflammatory	70-19-9		C11H13NO3
Thymocartin	Immunomodulator	85466-18-8		$C_{21}H_{40}N_8O_7$
Thymoctonan	Antiviral	107489-37-2		$C_{43}H_{67}N_9O_{13}$
Thymol	Antibacterial	89-83-8		$C_{10}H_{14}O$
Thymopentin	Immunosuppressant	69558-55-0		$C_{30}H_{49}N_9O_9$
Thymotrinan	Immunomodulator	85465-82-3		$C_{16}H_{31}N_7O_6$
Thyromedan	Thyroid	15301-96-9		$C_{21}H_{24}I_3NO_4$
Thyropropic Acid	Antihyperlipidemic	51-26-3		$C_{15}H_{11}I_3O_4$
Thyroxine	Thyroid	51-48-9		$C_{15}H_{11}I_{4NO4}$
Tiacrilast	Antihistaminic	78299-53-3		$C_{12}H_{10}N_2O_3S$
Tiadenol	Antihyperlipidemic	6964-20-1		$C_{14}H_{30}O_2S_2$
Tiafibrate	Antihyperlipidemic	55837-28-0		$C_{34}H_{48}Cl_2O_6S_2$
Tiagabine	Anticonvulsant	115103-54-3	Y	$C_{20}H_{25}NO_2S_2$
Tiamenidine	Antihypertensive	31428-61-2		$C_8H_{10}ClN_3S$
Tiametonium Iodide	Cholinergic	10433-71-3		$C_{12}H_{30}I_2N_2S$
Tiamulin Fumarate	Antibacterial	55297-95-5		$C_{28}H_{47}NO_4S$
Tianafac	Antiinflammatory	51527-19-6		$C_{11}H_9ClO_2S$
Tianeptine	Antidepressant	72797-41-2	Y	$C_{21}H_{25}ClN_2O_4S$
Tiapamil	Antiarrhythmic	57010-31-8		$C_{26}H_{37}NO_8S_2$
Tiapirinol	Antibacterial	14785-50-3		$C_{12}H_{16}N_2O_4S$
Tiapride	Antispasmodic	51012-32-9	Y	$C_{15}H_{24}N_2O_4S$
Tiaprofenic Acid	Antiinflammatory	33005-95-7	Y	$C_{14}H_{12}O_3S$
Tiaprost	Prostaglandin	71116-82-0		$C_{20}H_{28}O_6S$
Tiaramide	Antiinflammatory	32527-55-2		$C_{15}H_{18}ClN_3O_3S$
Tiazofurin	Antineoplastic	60084-10-8		$C_9H_{12}N_2O_5S$
Tiazuril	Antibacterial	35319-70-1		$C_{17}H_{14}ClN_3O_{2S}$
Tibalosin	Antihypertensive	63996-84-9		$C_{21}H_{27}NOS$
Tibeglisene	Unclassified	134993-74-1		$C_{18}H_{15}ClO_4S$

Table S1. *Cont.*

Common Name	Indication	CAS Number	Oral	Molecular Formula
Tibenelast Sodium	Bronchodilator	97852-72-7		$C_{13}H_{14}O_4S$
Tibenzate	Unclassified	13402-51-2		$C_{14}H_{12}OS$
Tibezonium Iodide	Antibacterial	54663-47-7	Y	$C_{28}H_{32}IN_3S_2$
Tibolone	Steroid	5630-53-5	Y	$C_{21}H_{28}O_2$
Tibric Acid	Antihyperlipidemic	7087-94-8		$C_{14}H_{18}ClNO_4S$
Ticabesone Propionate	Glucocorticoid	73205-13-7		$C_{25}H_{32}F2O5S$
Ticalopride	Antiemetic	202590-69-0		$C_{14}H_{20}ClN_3O_3$
Ticarbodine	Anthelminithic	31932-09-9		$C_{15}H_{19}F_3N_2S$
Ticarcillin Cresyl Sodium	Antibacterial	59070-07-4		$C_{22}H_{22}N_2O_6S_2$
Ticarcillin	Antibacterial	34787-01-4		$C_{15}H_{16}N_2O_6S_2$
Ticlatone	Antibacterial	70-10-0		C_7H_4ClNOS
Ticlopidine	Antithrombotic	55142-85-3	Y	$C_{14}H_{14}ClNS$
Ticolubant	Dermatologic	154413-61-3		$C_{23}H_{19}Cl_2NO_3S$
Ticrynafen	Diuretic	40180-04-9		$C_{13}H_8Cl_2O_4S$
Tidembersat	Antimigraine	175013-73-7		$C_{20}H_{19}F_2NO_4$
Tidiacic	Unclassified	30097-06-4		$C_5H_7NO_4S$
Tiemonium Iodide	Antispasmodic	144-12-7		$C_{18}H_{24}INO_2S$
Tienocarbine	Antipsychotic	75458-65-0		$C_{15}H_{16}N_2S$
Tienopramine	Antidepressant	37967-98-9		$C_{17}H_{20}N_2S$
Tienoxolol	Diuretic	90055-97-3		$C_{21}H_{28}N_2O_5S$
Tifacogin	Anticoagulant	148883-56-1		$C_{12}H_{13}NS$
Tifemoxone	Antidepressant	39754-64-8		$C_{11H13NO2S}$
Tifenazoxide	Antihypertensive	279215-43-9		$C_9H_{10}ClN_3O_2S_2$
Tiflamizole	Antiinflammatory	62894-89-7		$C_{17}H_{10}F_6N_2O_{2S}$
Tiflorex	Anorexic	53993-67-2		$C_{12}H_{16}F_3NS$
Tifluadom	Analgesic	83386-35-0		$C_{22}H_{20}FN_3OS$
Tiflucarbine	Antidepressant	89875-86-5		$C_{16}H_{17}FN_2S$
Tiformin	Antidiabetic	4210-97-3		$C_5H_{12}N_4O$
Tifurac Sodium	Analgesic	97483-17-5		$C_{18}H_{14}O_4S$
Tigecycline	Antibiotic	220620-09-7		$C_{29}H_{39}N_5O_8$
Tigemonam Dicholine	Antibacterial	102916-21-2		$C_{22}H_{41}N_7O_{11}S_2$
Tigestol	Progestogen	896-71-9		$C_{20}H_{28}O$
Tigloidine	Antispasmodic	495-83-0		$C_{13}H_{21}NO_2$
Tilarginine	Antineoplastic	17035-90-4		$C_7H_{16}N_4O_2$
Tilbroquinol	Antibiotic	7175-09-9		$C_{10}H_8BrNO$
Tiletamine	Anesthetic	14176-49-9		$C_{12}H_{17}NOS$
Tilidine	Analgesic	51931-66-9	Y	$C_{17}H_{23}NO_2$
Tiliquinol	Antibiotic	5541-67-3		$C_{10}H_9NO$
Tilisolol	Antiarrhythmic	85136-71-6	Y	$C_{17}H_{24}N_2O_3$
Tilmacoxib	Antirheumatic	180200-68-4		$C_{16}H_{19}FN_2O_3S$
Tilmicosin	Antibacterial	108050-54-0		$C_{46}H_{80}N_2O_{13}$
Tilnoprofen Arbamel	Antiinflammatory	118635-52-2		$C_{20}H_{22}N_2O_4$
Tilomisole	Immunomodulator	58433-11-7		$C_{17}H_{11}ClN_2O_2S$
Tilorone	Antiviral	27591-97-5		$C_{25}H_{34}N_2O_3$
Tilozepine	Analgesic	42239-60-1		$C_{17}H_{18}ClN_3S$
Tilsuprost	Prostaglandin	80225-28-1		$C_{20}H_{33}NO_4S$
Tiludronate	Bone Resorption Inhibitor	89987-06-4		$C_7H_9ClO_6P_2S$
Timcodar	Antineoplastic	179033-51-3		$C_{43}H_{45}ClN_4O_6$
Timefurone	Antihyperlipidemic	76301-19-4		$C_{15}H_{14}O_5S$
Timegadine	Antiinflammatory	71079-19-1		$C_{20}H_{23}N_5S$
Timelotem	Antipsychotic	96306-34-2		$C_{17}H_{18}FN_3S$
Timepidium Bromide	Mydriatic	35035-05-3		$C_{17}H_{22}BrNOS_2$
Timiperone	Antipsychotic	57648-21-2		$C_{22}H_{24}FN_3OS$
Timirdine	Antipsychotic	100417-09-2		$C_9H_{10}ClN_3S$
Timobesone Acetate	Steroid	79578-14-6		$C_{24}H_{31}FO_5S$
Timofibrate	Antihyperlipidemic	64179-54-0		$C_{14}H_{16}ClNO_4S$
Timolol	Antihypertensive	26839-75-8	Y	$C_{13}H_{24}N_4O_3S$
Timonacic	Hepatic Protectant	444-27-9	Y	$C_4H_7NO_2S$
Timoprazole	Antiulcerative	57237-97-5		$C_{13}H_{11}N_3OS$
Tinabinol	Antihypertensive	50708-95-7		$C_{23}H_{34}O_2S$
Tinazoline	Antihypertensive	62882-99-9		$C_{11}H_{11}N_3S$
Tinidazole	Antiprotozoal	19387-91-8	Y	$C_8H_{13}N_3O_4S$
Tinisulpride	Gastroprokinetic	69387-87-7		$C_{20}H_{29}N_3O_4S$

Table S1. *Cont.*

Common Name	Indication	CAS Number	Oral	Molecular Formula
Tinofedrine	Vasodilator	36702-83-7		$C_{20}H_{21}NOS_2$
Tinoridine	Analgesic	24237-54-5		$C_{17}H_{20}N_2O_2S$
Tiocarlide	Antibacterial	910-86-1		$C_{23}H_{32}N_2O_2S$
Tioclomarol	Anticoagulant	22619-35-8		$C_{22}H_{16}Cl_2O_4S$
Tioconazole	Antifungal	65899-73-2		$C_{16}H_{13}Cl_3N_2OS$
Tioctilate	Hepatic Protectant	10489-23-3		$C_{15}H_{22}OS$
Tiodazosin	Antihypertensive	66969-81-1		$C_{18}H_{21}N_7O_4S$
Tiodonium Chloride	Antibacterial	38070-41-6		$C_{10}H_7Cl_2IS$
Tiomergine	Antipsychotic	57935-49-6		$C_{21}H_{21}N_3S$
Tiomesterone	Androgen	2205-73-4		$C_{24}H_{34}O_4S_2$
Tioperidone	Antipsychotic	52618-67-4		$C_{25}H_{32}N_4O_2S$
Tiopinac	Antiinflammatory	61220-69-7		$C_{16}H_{12}O_3S$
Tiopronin	Antidote	1953-02-2		$C_5H_9NO_3S$
Tiopropamine	Antihistaminic	39516-21-7		$C_{24}H_{27}NS$
Tiospirone	Antipsychotic	87691-91-6		$C_{24}H_{32}N_4O_2S$
Tiotidine	Antihistaminic	69014-14-8		$C_{10}H_{16}N_8S_2$
Tiotropium	Bronchodilator	186691-13-4		$C_{19}H_{22}NO_4S_2$
Tioxacin	Antibiotic	34976-39-1		$C_{14}H_{12}N_2O_4S$
Tioxamast	Antihistaminic	74531-88-7		$C_{14}H_{14}N_2O_4S$
Tioxaprofen	Antiinflammatory	40198-53-6		$C_{18}H_{13}Cl_2NO_3S$
Tioxidazole	Anthelminithic	61570-90-9		$C_{12}H_{14}N_2O_3S$
Tioxolone	Dermatologic	4991-65-5		$C_7H_4O_3S$
Tipentosin	Antihypertensive	95588-08-2		$C_{21}H_{25}NO_3S$
Tipepidine	Antitussive	5169-78-8		$C_{15}H_{17}NS_2$
Tipetropium Bromide	Antispasmodic	54376-91-9		$C_{25}H_{32}BrNOS$
Tipifarnib	Antineoplastic	192185-72-1	Y	$C_{27}H_{22}Cl_2N_4O$
Tipindole	Antidepressant	7489-66-9		$C_{16}H_{20}N_2O_2S$
Tiplasinin	Anticoagulant	393105-53-8		$C_{24}H_{16}F_3NO_4$
Tipranavir	Antiviral	174484-41-4	Y	$C_{31}H_{33}F_3N_2O_5S$
Tipredane	Steroid	85197-77-9		$C_{22}H_{31}FO_2S_2$
Tiprenolol	Antihypertensive	26481-51-6		$C_{13}H_{21}NO_2S$
Tiprinast Meglumine	Antihistaminic	83153-39-3		$C_{12}H_{14}N_2O_3S$
Tipropidil	Vasodilator	70895-45-3		$C_{20}H_{35}NO_2S$
Tiprostanide	Prostaglandin	67040-53-3		$C_{33}H_{45}NO_6S$
Tiprotimod	Immunomodulator	105523-37-3		$C_{10}H_{13}NO_4S_2$
Tiqueside	Antihyperlipidemic	99759-19-0		$C_{39}H_{64}O_{13}$
Tiquinamide	Mydriatic	53400-67-2		$C_{11}H_{14}N_2S$
Tiquizium Bromide	Antispasmodic	71731-58-3		$C_{19}H_{24}BrNS_2$
Tiracizine	Antidepressant	83275-56-3		$C_{21}H_{25}N_3O_3$
Tirapazamine	Antineoplastic	27314-97-2		$C_7H_6N_4O_2$
Tiratricol	Thyroid	51-24-1		$C_{14}H_9I_3O_4$
Tirilazad	Capillary Protectant	110101-66-1		$C_{38}H_{52}N_6O_2$
Tirofiban	Antithrombotic	144494-65-5		$C_{22}H_{36}N_2O_5S$
Tiropramide	Antispasmodic	55837-29-1		$C_{28}H_{41}N_3O_3$
Tisocromide	Antipsychotic	35423-51-9		$C_{19}H_{30}N_2O_6S$
Tisopurine	Antineoplastic	5334-23-6		$C_5H_4N_4S$
Tisoquone	Antineoplastic	40692-37-3		$C_{17}H_{17}NS$
Tivanidazole	Antiprotozoal	80680-05-3		$C_{11}H_{13}N_5O_2S$
Tiviciclovir	Antiviral	103024-93-7		$C_9H_{13}N_5O_3$
Tivirapine	Antiviral	137332-54-8		$C_{16}H_{20}ClN_3S$
Tixadil	Unclassified	2949-95-3		$C_{24}H_{25}NS$
Tixanox	Antihistaminic	40691-50-7		$C_{15}H_{10}O_5S$
Tixocortol Pivalate	Antiinflammatory	55560-96-8		$C_{26}H_{38}O_5S$
Tizabrin	Unclassified	83573-53-9		$C_8H_{15}NO_3S$
Tizanidine	Antispasmodic	51322-75-9	Y	$C_9H_8ClN_5S$
Tizolemide	Diuretic	56488-58-5		$C_{11}H_{14}ClN_3O_3S_2$
Tizoprolic Acid	Antihyperlipidemic	30709-69-4		C7H9NO2S
Tnp-470	Antineoplastic	129298-91-5		$C_{19}H_{28}ClNO_6$
Tobicillin	Antibiotic	151287-22-8		$C_{27}H_{30}N_2O_6S$
Toborinone	Cardiotonic	143343-83-3		$C_{21}H_{24}N_2O_5$
Tobramycin	Antibiotic	32986-56-4		$C_{18}H_{37}N_5O_9$
Tobuterol	Bronchodilator	75626-99-2		$C_{28}H_{31}NO_5$
Tocainide	Antiarrhythmic	41708-72-9		$C_{11}H_{16}N_2O$

Table S1. *Cont.*

Common Name	Indication	CAS Number	Oral	Molecular Formula
Tocamphyl	Choleretic	465-27-0		$C_{19}H_{26}O_4$
Tocladesine	Antineoplastic	41941-56-4		$C_{10}H_{11}ClN_5O_6P$
Tocofenoxate	Antidepressant	61343-44-0		$C_{37}H_{55}ClO_4$
Tocofibrate	Antihyperlipidemic	50465-39-9		$C_{39}H_{59}ClO_4$
Todralazine	Antihypertensive	14679-73-3		$C_{11}H_{12}N_4O_2$
Tofenacin	Antidepressant	15301-93-6		$C_{17}H_{21}NO$
Tofetridine	Analgesic	40173-75-9		$C_{15}H_{21}NO$
Tofimilast	Bronchodilator	185954-27-2		$C_{18}H_{21}N_5S$
Tofisoline	Unclassified	29726-99-6		$C_{22}H_{26}N_2O_4$
Tofisopam	Anxiolytic	22345-47-7		$C_{22}H_{26}N_2O_4$
Tolafentrine	Erectile Dysfunction	139308-65-9		$C_{28}H_{31}N_3O_4S$
Tolamolol	Vasodilator	38103-61-6		$C_{19}H_{24}N_2O_4$
Tolazamide	Antidiabetic	1156-19-0		$C_{14}H_{21}N_3O_3S$
Tolazoline	Vasodilator	59-98-3		$C_{10}H_{12}N_2$
Tolboxane	Antianginal	2430-46-8		$C_{14}H_{21}BO_2$
Tolbutamide	Antidiabetic	64-77-7		$C_{12}H_{18}N_2O_3S$
Tolcapone	Antiparkinsonian	134308-13-7	Y	$C_{14}H_{11}NO_5$
Tolciclate	Antifungal	50838-36-3		$C_{20}H_{21}NOS$
Toldimfos	Antineoplastic	57808-64-7		$C_9H_{14}NO_2P$
Tolfamide	Antibiotic	70788-29-3		$C_8H_{12}N_3O_2P$
Tolfenamic Acid	Antiinflammatory	13710-19-5		$C_{14}H_{12}ClNO_2$
Tolgabide	Anticonvulsant	86914-11-6		$C_{18}H_{18}Cl_2N_2O_2$
Tolimidone	Antiulcerative	41964-07-2		$C_{11}H_{10}N_2O_2$
Tolindate	Antifungal	27877-51-6		$C_{18}H_{19}NOS$
Toliodium Chloride	Antibacterial	19028-28-5		$C_{14}H_{14}ClI$
Toliprolol	Antianginal	2933-94-0		$C_{13}H_{21}NO_2$
Tolmesoxide	Antihypertensive	38452-29-8		$C_{10}H_{14}O_3S$
Tolmetin	Antiinflammatory	26171-23-3		$C_{15}H_{15}NO_3$
Tolnaftate	Antifungal	2398-96-1		$C_{19}H_{17}NOS$
Tolnapersine	Antihypertensive	70312-00-4		$C_{21}H_{26}N_2O$
Tolnidamine	Antihistaminic	50454-68-7		$C_{16}H_{13}ClN_2O_2$
Toloconium Metilsulfate	Antifungal	552-92-1		$C_{23}H_{43}NO_4S$
Tolonidine	Antihypertensive	4201-22-3		$C_{10}H_{12}ClN_3$
Toloxatone	Antidepressant	29218-27-7		C11H13NO3
Tolpadol	Analgesic	77502-27-3		$C_{28}H_{26}N_4O_2$
Tolpentamide	Antispasmodic	1027-87-8		$C_{13}H_{18}N_2O_3S$
Tolperisone	Muscle Relaxant	728-88-1	Y	$C_{16}H_{23}NO$
Tolpiprazole	Antipsychotic	20326-13-0		$C_{17}H_{24}N_4$
Tolpronine	Analgesic	97-57-4		$C_{15}H_{21}NO_2$
Tolpropamine	Antihistaminic	5632-44-0		$C_{18}H_{23}N$
Tolpyrramide	Antidiabetic	5588-38-5		$C_{12}H_{16}N_2O_3S$
Tolquinzole	Unclassified	6187-50-4		$C_{16}H_{23}NO$
Tolrestat	Antidiabetic	82964-04-3		$C_{16}H_{14}F_3NO_3S$
Tolterodine	Misc- Urologic	124937-51-5	Y	$C_{22}H_{31}NO$
Toltrazuril	Antibacterial	69004-03-1		$C_{18}H_{14}F_3N_3O_4S$
Tolufazepam	Anxiolytic	86273-92-9		$C_{24}H_{20}Cl_2N_2O_3S$
Tolvaptan	Cardiotonic	150683-30-0	Y	$C_{26}H_{25}ClN_2O_3$
Tolycaine	Anesthetic	3686-58-6		$C_{15}H_{22}N_2O_3$
Tomeglovir	Antiviral	233254-24-5		$C_{23}H_{27}N_3O_4S$
Tomelukast	Bronchodilator	88107-10-2		$C_{16}H_{22}N_4O_3$
Tomoglumide	Gastroprokinetic	97964-54-0		$C_{24}H_{38}N_2O_4$
Tomoxiprole	Antiinflammatory	76145-76-1		$C_{21}H_{20N2O}$
Tonabersat	Antimigraine	175013-84-0		$C_{20}H_{19}ClFNO_4$
Tonazocine Mesylate	Analgesic	71461-18-2		C_{23}H35NO2
Topicaine	Analgesic	76-91-5		$C_{26}H_{37}NO_4$
Topilutamide	Sedative	260980-89-0		$C_{13}H_{11}F_6N_3O_5$
Topiramate	Anticonvulsant	97240-79-4	Y	$C_{12}H_{21}NO_8S$
Topixantrone	Antineoplastic	156090-18-5		$C_{21}H_{26}N_6O_2$
Topotecan	Antineoplastic	123948-87-8	Y	$C_{23}H_{23}N_3O_5$
Toprilidine	Analgesic	54063-58-0		$C_{19}H_{25}N_3O$
Topterone	Steroid	60607-35-4		$C_{22}H_{34}O_2$
Toquizine	Mydriatic	7125-71-5		$C_{23}H_{29}N_5O$
Torbafylline	Bronchodilator	105102-21-4		$C_{16}H_{26}N_4O_4$

Table S1. *Cont.*

Common Name	Indication	CAS Number	Oral	Molecular Formula
Torcetrapib	Antihyperlipidemic	262352-17-0		$C_{26}H_{25}F_9N_2O_4$
Torcitabine	Antiviral	40093-94-5		$C_9H_{13}N_3O_4$
Toremifene	Antineoplastic	89778-26-7	Y	$C_{26}H_{28}ClNO$
Toripristone	Progestogen	91935-26-1		$C_{31}H_{39}NO_2$
Torsemide	Diuretic	56211-40-6		$C_{16}H_{20}N_4O_3S$
Tosagestin	Contraceptive	110072-15-6		$C_{21}H_{24}O_2$
Tosifen	Antianginal	32295-18-4		$C_{17}H_{20}N_2O_3S$
Tosufloxacin	Antibiotic	100490-36-6	Y	$C_{19}H_{15}F_3N_4O_3$
Tosulur	Unclassified	87051-13-6		$C_{11}H_{15}NO_5S$
Trabectedin	Antineoplastic	114899-77-3		$C_{39}H_{43}N_3O_{11}S$
Traboxopine	Antipsychotic	103624-59-5		$C_{19}H_{23}ClN_2O_2$
Tracazolate	Sedative	41094-88-6		$C_{16}H_{24}N_4O_2$
Tradecamide	Unclassified	132787-19-0		$C_{15}H_{31}NO_2$
Tralonide	Glucocorticoid	21365-49-1		$C_{24}H_{28}Cl_2F_2O_4$
Tramadol	Analgesic	27203-92-5	Y	$C_{16}H_{25}NO_2$
Tramazoline	Decongestant	1082-57-1		$C_{13}H_{17}N_3$
Tramiprosate	Nootropic	3687-18-1		$C_3H_9NO_3S$
Trandolapril	Antihypertensive	87679-37-6		$C_{24}H_{34}N_2O_5$
Trandolaprilat	Antihypertensive	87679-71-8		$C_{22}H_{30}N_2O_5$
Tranexamic Acid	Hemostatic	1197-18-8		$C_8H_{15}NO_2$
Tranilast	Antihistaminic	53902-12-8		$C_{18}H_{17}NO_5$
Transcainide	Antiarrhythmic	88296-62-2		$C_{22}H_{35}N_3O_2$
Trantelinium Bromide	Antispasmodic	4047-34-1		$C_{23}H_{26}NO_3.Br$
Tranylcypromine	Antidepressant	155-09-9		$C_9H_{11}N$
Trapencaine	Anesthetic	104485-01-0		$C_{22}H_{34}N_2O_3$
Trapidil	Vasodilator	15421-84-8		$C_{10}H_{15}N_5$
Travoprost	Antiglaucoma	157283-68-6		$C_{26}H_{35}F_3O_6$
Traxanox	Antihistaminic	58712-69-9		$C_{13}H_6ClN_5O_2$
Traxoprodil Mesylate	Nootropic	134234-12-1		$C_{20}H_{25}NO_3$
Trazitiline	Antidepressant	26070-23-5		$C_{21}H_{24}N_2$
Trazium Esilate	Antidepressant	97110-59-3		$C_{19}H_{18}ClN_3O_4S$
Trazodone	Antidepressant	19794-93-5	Y	$C_{19}H_{22}ClN_5O$
Trazolopride	Antiemetic	86365-92-6		$C_{20}H_{23N5O2}$
Trebenzomine	Antidepressant	23915-73-3		$C_{12}H_{17}NO$
Trecadrine	Antidiabetic	108767-50-6		C27H29NO
Trecetilide	Antiarrhythmic	180918-68-7		$C_{21}H_{37}FN_2O_3S$
Trefentanil	Analgesic	120656-74-8		$C_{25}H_{31}FN_6O_2$
Trelnarizine	Antihistaminic	123205-52-7		$C_{28}H_{30}F_2N_2O_2$
Treloxinate	Antihyperlipidemic	30910-27-1		$C_{16}H_{12}Cl_2O_4$
Trenbolone Acetate	Steroid	10161-34-9		$C_{20}H_{24}O_3$
Trengestone	Progestogen	5192-84-7		$C_{21}H_{25}ClO_2$
Trenizine	Antihistaminic	89224-07-7		$C_{31}H_{40}N_2O$
Treosulfan	Antineoplastic	299-75-2	Y	$C_6H_{14}O_8S_2$
Trepibutone	Choleretic	41826-92-0		$C_{16}H_{22}O6$
Trepipam Maleate	Sedative	56030-50-3		$C_{19}H_{23}NO_2$
Trepirium Iodide	Antihypertensive	1018-34-4		$C_{12}H_{26}I_2N_2O_2$
Treprostinil	Antihypertensive	81846-19-7	Y	$C_{23}H_{34}O_5$
Treptilamine	Antispasmodic	58313-74-9		$C_{20}H_{27}NO$
Trequinsin	Antihypertensive	79855-88-2		$C_{24}H_{27}N_3O_3$
Tresperimus	Immunosuppressant	160677-67-8		$C_{17}H_{37}N_7O_3$
Trestolone Acetate	Androgen	6157-87-5		$C_{21}H_{30}O_3$
Tretazicar	Antineoplastic	21919-05-1		$C_9H_8N_4O_5$
Trethinium Tosilate	Unclassified	1748-43-2		$C_{19}H_{25}NO_3S$
Tretinoin	Antineoplastic	302-79-4	Y	$C_{20}H_{28}O_2$
Tretinoin Tocoferil	Dermatologic	40516-48-1		$C_{49}H_{76}O_3$
Tretoquinol	Bronchodilator	30418-38-3		$C_{19}H_{23}NO_5$
Triacetin	Antifungal	102-76-1		$C_9H_{14}O_6$
Triafungin	Antifungal	55242-77-8		$C_{13}H_{10}N_4$
Triamcinolone	Glucocorticoid	124-94-7		$C_{21}H_{27}FO_6$
Triamcinolone Acetonide	Glucocorticoid	76-25-5		$C_{24}H_{31}FO_6$
Triamcinolone Benetonide	Glucocorticoid	31002-79-6		$C_{35}H_{42FNO8}$
Triamcinolone Diacetate	Glucocorticoid	67-78-7		$C_{25}H_{31}FO_8$
Triamcinolone Furetonide	Glucocorticoid	4989-94-0		$C_{33}H_{35}FO_8$

Table S1. *Cont.*

Common Name	Indication	CAS Number	Oral	Molecular Formula
Triamcinolone Hexacetonide	Antiinflammatory	5611-51-8		$C_{30}H_{41}FO_7$
Triampyzine Sulfate	Mydriatic	6503-95-3		$C_9H_{15}N_3$
Triamterene	Diuretic	396-01-0		$C_{12}H_{11}N_7$
Triaziquone	Antineoplastic	68-76-8		$C_{12}H_{13}N_3O_2$
Triazolam	Sedative	28911-01-5	Y	$C_{17}H_{12}Cl2N4$
Tribendilol	Antihypertensive	96258-13-8		$C_{18}H_{22}N_4O_4$
Tribenoside	Dermatologic	10310-32-4		$C_{29}H_{34}O_6$
Tribromoethanol	Anesthetic	75-80-9		$C_2H_3Br_3O$
Tribromsalan	Antibacterial	87-10-5		$C_{13}H_8Br_3NO_2$
Tribuzone	Antiinflammatory	13221-27-7		$C_{22}H_{24}N_2O_3$
Tricetamide	Sedative	363-20-2		$C_{16}H_{24}N_2O_5$
Trichlormethiazide	Diuretic	133-67-5		$C_8H_8Cl_3N_3O_4S_2$
Trichlormethine	Antineoplastic	555-77-1		$C_6H_{12}Cl_3N$
2,2,2-Trichloroethanol	Sedative	115-20-8		$C_2H_3Cl_3O$
Trichloroethylene	Anesthetic	79-01-6		C_2HCl_3
Triciribine	Antineoplastic	35943-35-2		$C_{13}H_{16}N_6O_4$
Triclabendazole	Anthelminithic	68786-66-3		$C_{14}H_9Cl_3N_2OS$
Triclacetamol	Analgesic	6340-87-0		$C_8H_6Cl_3NO_2$
Triclazate	Anorexic	7009-76-9		$C_{20}H_{23}NO_3$
Triclodazol	Antiprotozoal	56-28-0		$C_{17}H_{15}Cl_3N_2O_2$
Triclofenol Piperazine	Anthelminithic	95-95-4		$C_6H_3Cl_3O$
Triclofos	Sedative	306-52-5		$C_2H_4Cl_3O_4P$
Triclofylline	Bronchodilator	17243-70-8		$C_{11}H_{13}Cl_3N_4O_4$
Triclonide	Antiinflammatory	26849-57-0		$C_{24}H_{28}Cl_3FO_4$
Tricyclamol Chloride	Antiparkinsonian	3818-88-0		$C_{20}H_{32}ClNO$
Tridihexethyl	Antispasmodic	60-49-1		$C_{21}H_{36}NO+$
Tridihexethyl Iodide	Mydriatic	125-99-5		$C_{21}H_{36}INO$
Tridolgosir	Antineoplastic	72741-87-8		$C_8H_{15}NO_3$
Trientine	Antidote	112-24-3		$C_6H_{18}N_4$
Triethylamine	Antiinflammatory	121-44-8		$C_6H_{15}N$
Triethylene Glycol	Antineoplastic	112-27-6		$C_6H_{14}O_4$
Triethylene Glycol Diacetate	Antineoplastic	111-21-7		$C_{10}H_{18}O_6$
Triethylenemelamine	Antineoplastic	51-18-3		$C_9H_{12}N_6$
Trifenagrel	Antithrombotic	84203-09-8		$C_{25}H_{25}N_3O$
Trifezolac	Antiinflammatory	32710-91-1		$C_{23}H_{18}N_2O_2$
Triflocin	Diuretic	13422-16-7		$C_{13}H_9F_3N_2O_2$
Triflubazam	Anxiolytic	22365-40-8		$C_{17}H_{13}F_3N_2O_2$
Triflumidate	Antiinflammatory	24243-89-8		$C_{17}H_{14}F_3NO_5S$
Trifluomeprazine	Analgesic	2622-37-9		$C_{19}H_{21}F_3N_2S$
Trifluoperazine	Antipsychotic	117-89-5		$C_{21}H_{24}F_3N_3S$
Trifluperidol	Antipsychotic	749-13-3		$C_{22}H_{23}F_4NO_2$
Triflupromazine	Antipsychotic	146-54-3		$C_{18}H_{19}F_3N_2S$
Trifluridine	Antiviral	70-00-8		$C_{10}H_{11}F_3N_2O5$
Triflusal	Antithrombotic	322-79-2		$C_{10}H_7F_3O_4$
Trigevolol	Antihypertensive	76812-98-1		$C_{21}H_{28}N_2O_7$
Trihexyphenidyl	Antiparkinsonian	144-11-6		$C_{20}H_{31}NO$
Triletide	Anticoagulant	62087-96-1		$C_{27}H_{31}N_5O_5$
Trilostane	Pituitary	13647-35-3		$C_{20}H_{27}NO_3$
Trimazosin	Antihypertensive	35795-16-5		$C_{20}H_{29}N_5O_6$
Trimebutine	Antispasmodic	39133-31-8		$C_{22}H_{29}NO_5$
Trimecaine	Anesthetic	616-68-2		$C_{15}H_{24}N_2O$
Trimedoxime Bromide	Antidote	56-97-3		$C_{15}H_{18}Br_2N_4O_2$
Trimegestone	Progestogen	74513-62-5		$C_{22}H_{30}O_3$
Trimeperidine	Analgesic	64-39-1		$C_{17}H_{25}NO_2$
Trimetazidine	Antianginal	5011-34-7		$C_{14}H_{22}N_2O_3$
Trimethadione	Anticonvulsant	127-48-0		$C_6H_9NO_3$
Trimethamide	Nootropic	5789-72-0		$C_{17}H_{21}N_3O_4$
Trimethaphan Camsylate	Antihypertensive	68-91-7		$C_{22}H_{25}N_2OS.C_{10}H_{15}O_4S$
Trimethidinium Methosulfate	Antihypertensive	14149-43-0		$C_{19}H_{42}N_2O_8S_2$
Trimethobenzamide	Antiemetic	138-56-7		$C_{21}H_{28}N_2O_5$
Trimethoprim	Antibacterial	738-70-5	Y	$C_{14}H_{18}N_4O_3$
Trimethylcetylammonium Pentachlorophenate	Antibacterial	87-76-3		$C_{19}H_{42}N.C_6Cl_5O$

Table S1. *Cont.*

Common Name	Indication	CAS Number	Oral	Molecular Formula
Trimetozine	Anxiolytic	635-41-6		$C_{14}H_{19}NO_5$
Trimetrexate	Antineoplastic	52128-35-5		$C_{19}H_{23}N_5O_3$
Trimetrexate Glucuronate	Antineoplastic	6556-12-3		$C_6H_{10}O_7$
Trimexiline	Unclassified	64015-58-3		$C_{17}H_{29}N$
Trimipramine	Antidepressant	739-71-9		$C_{20}H_{26}N_2$
Trimoprostil	Antiulcerative	69900-72-7		$C_{23}H_{38}O_4$
Trimoxamine	Antihypertensive	15686-23-4		$C_{15}H_{23}NO_3$
Trioxifene Mesylate	Estrogen	68307-81-3		$C_{30}H_{31}NO_3$
Trioxsalen	Dermatologic	3902-71-4		$C_{14}H_{12}O_3$
Tripamide	Diuretic	73803-48-2		$C_{16}H_{20}ClN_3O_3S$
Triparanol	Antihyperlipidemic	78-41-1		C_{27}H32ClNO2
Tripelennamine	Antihistaminic	91-81-6		$C_{16}H_{21}N_3$
Triprolidine	Antihistaminic	486-12-4		$C_{19}H_{22}N_2$
Tritiozine	Antiulcerative	35619-65-9		$C_{14}H_{19}NO_4S$
Tritoqualine	Antihistaminic	14504-73-5		$C_{26}H_{32}N_2O_8$
Trixolane	Unclassified	47420-28-0		$C_{18}H_{27}NO_6$
Trizoxime	Antibiotic	35710-57-7		$C_{16}H_{15}N_5O_2$
Trocimine	Antipsychotic	14368-24-2		$C_{17}H_{25}NO_4$
Trodusquemine	Antiobesity	186139-09-3		$C_{37}H_{72}N_4O_5S$
Trofosfamide	Antineoplastic	22089-22-1		$C_9H_{18}Cl_3N_2O_2P$
Troglitazone	Antidiabetic	97322-87-7		C24H27NO5S
Trolamine	Analgesic	102-71-6		$C_6H_{15}NO_3$
Troleandomycin	Antibiotic	2751-09-9		$C_{41}H_{67}NO_{15}$
Trolnitrate Phosphate	Antianginal	7077-34-1		$C_6H_{12}N_4O_9$
Tromantadine	Antiviral	53783-83-8		$C_{16}H_{28}N_2O_2$
Tromethamine	Oxytocic	77-86-1		$C_4H_{11}NO_3$
Tropabazate	Unclassified	64294-94-6		$C_{15}H_{19}N_3O_4$
Tropanserin	Antidepressant	85181-40-4		$C_{17}H_{23}NO_2$
Tropapride	Antipsychotic	76352-13-1		$C_{23}H_{28}N_2O_3$
Tropatepine	Antipsychotic	27574-24-9		$C_{22}H_{23}NS$
Tropenziline Bromide	Antispasmodic	143-92-0		$C_{24}H_{30}BrNO_4$
Tropicamide	Mydriatic	1508-75-4		$C_{17}H_{20}N_2O_2$
Tropigline	Unclassified	533-08-4		$C_{13}H_{21}NO_2$
Tropirine	Antihistamine	19410-02-7		$C_{22}H_{24}N_2O$
Tropisetron	Antiemetic	89565-68-4	Y	$C_{17}H_{20}N_2O_2$
Tropodifene	Antihypertensive	15790-02-0		$C_{25}H_{29}NO_4$
Troquidazole	Antiprotozoal	108001-60-1		$C_{14}H_{15}N_5O_3$
Trospectomycin	Antibiotic	88669-04-9		$C_{17}H_{30}N_2O_7$
Trospium	Antispasmodic	47608-32-2		$C_{25}H_{30}ClNO_3$
Trovafloxacin	Antibacterial	147059-72-1	Y	$C_{20}H_{15}F_3N_4O_3$
Trovirdine	Antiviral	149488-17-5		$C_{13}H_{13}BrN_4S$
Troxacitabine	Antineoplastic	145918-75-8		$C_8H_{11}N_3O_4$
Troxerutin	Capillary Protectant	7085-55-4		$C_{33}H_{42}O_{19}$
Troxipide	Antiulcerative	30751-05-4		$C_{15}H_{22N2O4}$
Troxypyrrolium Tosilate	Mydriatic	3612-98-4		$C_{25}H_{35}NO_8S$
Truxicurium Iodide	Muscle Relaxant	4304-01-2		$C_{34}H_{52}I_2N_2O_4$
Truxipicurium Iodide	Muscle Relaxant	35515-77-6		$C_{38}H_{56}I_2N_2O_4$
Tryparsamide	Antiprotozoal	618-25-7		$C_8H_{11}AsN_2O_4$
Tuaminoheptane	Decongestant	123-82-0		$C_7H_{17}N$
Tubocurarine	Neuromuscular Blocking Agent	57-95-4		$C_{37}H_{41}N_2O_6$
Tubulozole	Antineoplastic	84697-22-3		$C_{23}H_{23}Cl_2N_3O_4S$
Tucaresol	Immunomodulator	84290-27-7		$C_{15}H_{12}O_5$
Tuclazepam	Anxiolytic	51037-88-8		$C_{17}H_{16}Cl_2N_2O$
Tulathromycin	Antibacterial	217500-96-4		$C_{41}H_{79}N_3O_{12}$
Tulobuterol	Bronchodilator	41570-61-0		$C_{12}H_{18}ClNO$
Tulopafant	Anticoagulant	116289-53-3		$C_{25}H_{19}N_3O_2S$
Turosteride	Antineoplastic	137099-09-3		$C_{27}H_{45}N_3O_3$
Tuvatidine	Antiulcerative	91257-14-6		$C_{10}H_{17}N_9O_2S_3$
Tybamate	Anxiolytic	4268-36-4		$C_{13}H_{26}N_2O_4$
Tylosin	Antibiotic	1401-69-0		$C_{46}H_{77}NO_{17}$
Tymazoline	Decongestant	24243-97-8		$C_{14}H_{20}N_2O$
Ubenimex	Immunomodulator	58970-76-6		$C_{16}H_{24}N_2O_4$

Table S1. *Cont.*

Common Name	Indication	CAS Number	Oral	Molecular Formula
Ubidecarenone	Antihypertensive	303-98-0		$C_{59}H_{90}O_4$
Ubisindine	Unclassified	26070-78-0		$C_{20}H_{24}N_2O$
Udenafil	Erectile Dysfunction	268203-93-6	Y	$C_{25}H_{36}N_6O_4S$
Ufenamate	Antiinflammatory	67330-25-0		$C_{18}H_{18}F_3NO_2$
Ufiprazole	Anxiolytic	73590-85-9		$C_{17}H_{19}N_3O_2S$
Uldazepam	Anxiolytic	28546-58-9		$C_{18}H_{15}Cl_2N_3O$
Ulifloxacin	Antibiotic	112984-60-8		$C_{16}H_{16}FN_3O_3S$
Umespirone	Antidepressant	107736-98-1		$C_{28}H_{40}N_4O_5$
Undecylenic Acid	Antifungal	112-38-9		$C_{11}H_{20}O_2$
Unoprostone	Prostaglandin	120373-36-6		$C_{22}H_{38}O_5$
Upenazime	Antihypertensive	95268-62-5		$C_{14}H_{30}N_4O_2$
Upidosin	Antihypertensive	152735-23-4		$C_{31}H_{33}N_3O_4$
Uracil	Antineoplastic	66-22-8	Y	$C_4H_4N_2O_2$
Uracil Mustard	Antineoplastic	66-75-1		$C_8H_{11}Cl_2N_3O_2$
Urapidil	Antihypertensive	34661-75-1		$C_{20}H_{29}N_5O_3$
Urea	Diuretic	57-13-6		CH_4N_2O
Uredepa	Antineoplastic	302-49-8		$C_7H_{14}N_3O_3P$
Uredofos	Anthelminithic	52406-01-6		$C_{19}H_{25}N_4O_6PS_2$
Urefibrate	Bone Resorption Inhibitor	38647-79-9		$C_{15}H_{12}Cl_2N_2O_4$
Urethane	Antineoplastic	51-79-6		$C_3H_7NO_2$
Ursodiol	Anticholelithogenic	128-13-2	Y	$C_{24}H_{40}O_4$
Ursulcholic Acid	Anticholelithogenic	88426-32-8		$C_{24}H_{40}O_{10}S_2$
Utibapril	Antihypertensive	109683-61-6		$C_{22}H_{31}N_3O_5S$
Utibaprilat	Antihypertensive	109683-79-6		$C_{20}H_{27}N_3O_5S$
Vadocaine	Anesthetic	72005-58-4		$C_{18}H_{28}N_2O_2$
Valacyclovir	Antiviral	124832-26-4	Y	$C_{13}H_{20}N_6O_4$
Valategrast	Antihistamine	220847-86-9		$C_{30}H_{32}Cl_3N_3O_4$
Valconazole	Antifungal	56097-80-4		$C_{16}H_{18}Cl_2N_2O_2$
Valdecoxib	Antiinflammatory	181695-72-7	Y	$C_{16}H_{14}N_2O_3S$
Valdetamide	Sedative	512-48-1		$C_9H_{17}NO$
Valdipromide	Analgesic	52061-73-1		$C_{11}H_{23}NO$
Valethamate Bromide	Antispasmodic	90-22-2		$C_{19}H_{32}BrNO_2$
Valganciclovir	Antiviral	175865-60-8	Y	$C_{14}H_{22}N_6O_5$
Valnemulin	Antibacterial	101312-92-9		$C_{31}H_{54}N_2O_5S$
Valnoctamide	Anxiolytic	4171-13-5		$C_8H_{17}NO$
Valofane	Sedative	3258-51-3		$C_{10}H_{14}N_2O_4$
Valomaciclovir	Antiviral	195157-34-7		$C_{15}H_{24}N_6O_4$
Valopicitabine	Antineoplastic	640281-90-9		$C_{15}H_{24}N_4O_6$
Valperinol	Antihypertensive	64860-67-9		$C_{16}H_{27}NO_4$
Valproate Pivoxil	Anticonvulsant	77372-61-3		$C_{14}H_{26}O_4$
Valproic Acid	Anticonvulsant	99-66-1		$C_8H_{16}O_2$
Valpromide	Anticonvulsant	2430-27-5		$C_8H_{17}NO$
Valrocemide	Anticonvulsant	92262-58-3		$C_{10}H_{20}N_2O_2$
Valrubicin	Antineoplastic	56124-62-0		$C_{34}H_{36}F_3NO_{13}$
Valsartan	Antihypertensive	137862-53-4	Y	$C_{24}H_{29}N_5O_3$
Valtorcitabine Dihydrochloride	Antiviral	380886-95-3		$C_{14}H_{22}N_4O_5$
Valtrate	Sedative	18296-44-1		$C_{22}H_{30}O_8$
Vamicamide	Mydriatic	132373-81-0		$C_{18}H_{23}N_3O$
Vandetanib	Opthalmic	443913-73-3	Y	$C_{22}H_{24}BrFN_4O_2$
Vaneprim	Antibiotic	84854-86-4		$C_{23}H_{28}N_4O_8S$
Vanitiolide	Choleretic	17692-71-6		$C_{12}H_{15}NO_3S$
Vanoxerine	Antidepressant	67469-69-6		$C_{28}H_{32}F_2N_2O$
Vanyldisulfamide	Antibiotic	119-85-7		C_{20}H22N4O6S2
Vapiprost	Anticoagulant	85505-64-2		$C_{30}H_{39}NO_4$
Vardenafil	Erectile Dysfunction	224785-90-4	Y	$C_{23}H_{32}N_6O_4S$
Varenicline Tartrate	Antidepressant	249296-44-4		$C_{13}H_{13}N_3$
Varespladib Sodium	Antihypotensive	172732-68-2		$C_{21}H_{20}N_2O_5$
Vatalanib	Antineoplastic	212141-54-3		$C_{20}H_{15}ClN_4$
Vatanidipine	Antiarrhythmic	116308-55-5		$C_{41}H_{42}N_4O_6$
Vebufloxacin	Antibiotic	79644-90-9		$C_{19}H_{22}FN_3O_3$
Vecuronium Bromide	Neuromuscular Blocking Agent	50700-72-6		$C_{34}H_{57}BrN_2O_4$
Vedaclidine	Antiparkinsonian	141575-50-0		$C_{13}H_{21}N_3S_2$

Table S1. *Cont.*

Common Name	Indication	CAS Number	Oral	Molecular Formula
Vedaprofen	Antiinflammatory	71109-09-6		$C_{19}H_{22}O_2$
Velaresol	Immunomodulator	77858-21-0		$C_{12}H_{14}O_5$
Velnacrine	Nootropic	124027-47-0		$C_{13}H_{14}N_2O$
Venlafaxine	Antidepressant	93413-69-5	Y	$C_{17}H_{27}NO_2$
Venritidine	Antiulcerative	93064-63-2		$C_{18}H_{26}N_4O_3S$
Veradoline	Analgesic	82924-71-8		$C_{20}H_{26}N_2O_2$
Veralipride	Antipsychotic	66644-81-3		$C_{17}H_{25}N_3O_5S$
Verapamil	Antihypertensive	52-53-9	Y	$C_{27}H_{38}N_2O_4$
Verazide	Antibacterial	93-47-0		$C_{15}H_{15}N_3O_3$
Verilopam	Analgesic	68318-20-7		$C_{20}H_{26}N_2O_2$
Verlukast	Bronchodilator	120443-16-5		$C_{26}H_{27}ClN_2O_3S_2$
Verofylline	Bronchodilator	66172-75-6		$C_{12}H_{18}N_4O2$
Verteporfin	Antineoplastic	121310-58-5		$C_{40}H_{40}N_4O_8$
Vesnarinone	Cardiotonic	81840-15-5		$C_{22}H_{25}N_3O_4$
Vestipitant Mesylate	Antidepressant	334476-46-9		$C_{23}H_{24}F_7N_3O$
Vetrabutine	Antispasmodic	3735-45-3		$C_{20}H_{27}NO_2$
Vicriviroc Maleate	Antiviral	306296-47-9		$C_{28}H_{38}F_3N_5O_2$
Vidarabine	Antiviral	5536-17-4		$C_{10}H_{13}N_5O_4$
Vidarabine Phosphate	Antineoplastic	29984-33-6		$C_{10}H_{14}N_5O_7P$
Vigabatrin	Anticonvulsant	68506-86-5	Y	$C_6H_{11}NO_2$
Vilazodone	Antidepressant	163521-12-8	Y	$C_{26}H_{27}N_5O_2$
Vildagliptin	Antidiabetic	274901-16-5	Y	$C_{17}H_{25}N_3O_2$
Viloxazine	Antidepressant	46817-91-8		$C_{13}H_{19}NO_3$
Viminol	Analgesic	21363-18-8		$C_{21}H_{31}ClN_2O$
Vinbarbital	Sedative	125-42-8		$C_{11}H_{16}N_2O_3$
Vinblastine	Antineoplastic	865-21-4		$C_{46}H_{58}N_4O_9$
Vinburnine	Vasodilator	4880-88-0		$C_{19}H_{22}N_2O$
Vincamine	Antineoplastic	1617-90-9		$C_{21}H_{26}N_2O_3$
Vincanol	Anesthetic	19877-89-5		$C_{19}H_{24}N_2O$
Vincantril	Unclassified	65285-58-7		$C_{14}H_{13}ClN_2O$
Vincofos	Anthelminithic	17196-88-2		$C_{11}H_{21}Cl_2O_4P$
Vinconate	Nootropic	70704-03-9		$C_{18}H_{20}N_2O_2$
Vincristine	Antineoplastic	57-22-7		$C_{46}H_{56}N_4O_{10}$
Vindeburnol	Nootropic	68779-67-9		$C_{17}H_{20}N_2O$
Vindesine	Antineoplastic	53643-48-4		$C_{43}H_{55}N_5O_7$
Vinepidine Sulfate	Antineoplastic	68170-69-4		$C_{46}H_{56}N_4O_9$
Vinflunine	Antineoplastic	162652-95-1		$C_{45}H_{54}F_2N_4O_8$
Vinformide	Unclassified	54022-49-0		$C_{46}H_{54}N_4O_{10}$
Vinfosiltine	Antineoplastic	123286-00-0		$C_{51}H_{72}N_5O10P$
Vinglycinate Sulfate	Antineoplastic	865-24-7		$C_{48}H_{63}N_5O_9$
Vinleucinol	Antineoplastic	81571-28-0		$C_{51}H_{69}N_5O_9$
Vinleurosine Sulfate	Antineoplastic	23360-92-1		$C_{46}H_{56}N_4O_9$
Vinmegallate	Dermatologic	83482-77-3		$C_{30}H_{32}N_2O_5$
Vinorelbine	Antineoplastic	71486-22-1	Y	$C_{45}H_{54}N_4O_8$
Vinpocetine	Vasodilator	42971-09-5		$C_{22}H_{26}N_2O_2$
Vinpoline	Unclassified	57694-27-6		C23H30N2O3
Vinrosidine Sulfate	Antineoplastic	15228-71-4		$C_{46}H_{58}N_4O_9$
Vintoperol	Vasodilator	106498-99-1		$C_{18}H_{24}N_2O$
Vintriptol	Antineoplastic	81600-06-8		$C_{56}H_{68}N_6O_9$
Vinyl Ether	Anesthetic	109-93-3		C_4H_6O
Vinylbital	Sedative	2430-49-1		$C_{11}H_{16}N_2O_3$
Vinzolidine Sulfate	Antineoplastic	67699-40-5		$C_{48}H_{58}ClN_5O_9$
Viomycin	Antibiotic	32988-50-4		$C_{25}H_{43}N_{13}O_{10}$
Viprostol	Vasodilator	73647-73-1		$C_{23}H_{36}O_5$
Viqualine	Alcohol Deterrant	72714-74-0		$C_{20}H_{26}N_2O$
Viquidil	Vasodilator	84-55-9		$C_{20}H_{24}N_2O_2$
Viroxime	Antiviral	63198-97-0		$C_{17}H_{18}N_4O_3S$
Visnadine	Vasodilator	477-32-7		$C_{21}H_{24}O_7$
Visnafylline	Bronchodilator	17243-56-0		$C_{25}H_{29}N_5O_7$
Vofopitant	Antiemetic	168266-90-8		$C_{21}H_{23}F_3N_6O$
Voglibose	Antidiabetic	83480-29-9	Y	$C_{10}H_{21}NO_7$
Volazocine	Analgesic	15686-68-7		$C_{18}H_{25}N$
Volpristin	Antibiotic	21102-49-8		$C_{28}H_{37}N_3O_7$

Table S1. *Cont.*

Common Name	Indication	CAS Number	Oral	Molecular Formula
Voriconazole	Antifungal	137234-62-9	Y	$C_{16}H_{14}F_3N_5O$
Vorinostat	Antineoplastic	149647-78-9	Y	$C_{14}H_{20}N_2O_3$
Vorozole	Antineoplastic	129731-10-8		$C_{16}H_{13}ClN_6$
Voxergolide	Antiparkinsonian	89651-00-3		$C_{16}H_{20}N_2OS$
Warfarin	Anticoagulant	81-81-2		$C_{19}H_{16}O_4$
Xaliproden	Nootropic	135354-02-8		$C_{24}H_{22}F_3N$
Xamoterol	Cardiotonic	81801-12-9		$C_{16}H_{25}N_3O_5$
Xanomeline	Cholinergic	131986-45-3		$C_{14}H_{23}N_3OS$
Xanoxic Acid	Bronchodilator	33459-27-7		$C_{17}H_{14}O_5$
Xanthiol	Unclassified	14008-71-0		$C_{23}H_{29}ClN_2OS$
Xantifibrate	Antihyperlipidemic	2530-97-4		$C_{13}H_{21}N_5O_4$
Xantocillin	Antibiotic	580-74-5		$C_{18}H_{12}N_2O_2$
Xemilofiban	Antianginal	149820-74-6	Y	$C_{18}H_{22}N_4O_4$
Xenalipin	Antihyperlipidemic	84392-17-6		$C_{14}H_9F_3O_2$
Xenazoic Acid	Antiviral	1174-11-4		$C_{23}H_{21}NO_4$
Xenbucin	Antihyperlipidemic	959-10-4		$C_{16}H_{16}O_2$
Xenipentone	Unclassified	55845-78-8		$C_{17}H_{16}O$
Xenthiorate	Unclassified	7009-79-2		$C_{22}H_{29}NOS$
Xenygloxal	Unclassified	1094-85-5		$C_{16}H_{14}O_6$
Xenyhexenic Acid	Unclassified	95040-85-0	Y	$C_{18}H_{18}O_2$
Xenytropium Bromide	Antispasmodic	511-55-7		$C_{30}H_{34}BrNO_3$
Xibenolol	Antiarrhythmic	30187-90-7		$C_{15}H_{25}NO_2$
Xibornol	Antibacterial	13741-18-9		$C_{18}H_{26}O$
Xidecaflur	Unclassified	13127-82-7		$C_{22}H_{45}NO_2$
Xilobam	Muscle Relaxant	50528-97-7		$C_{14}H_{19}N_3O$
Ximelagatran	Antithrombotic	192939-46-1		$C_{24}H_{35}N_5O_5$
Ximoprofen	Antiinflammatory	56187-89-4		$C_{15}H_{19}NO_3$
Xinidamine	Analgesic	50264-78-3		$C_{17}H_{16}N_2O_2$
Xinomiline	Unclassified	52832-91-4		$C_5H_{10}N_2O$
Xipamide	Diuretic	14293-44-8		$C_{15}H_{15}ClN_2O_4S$
Xipranolol	Antihypertensive	19179-78-3		$C_{23}H_{33}NO_2$
Xorphanol Mesylate	Analgesic	77287-89-9		$C_{23}H_{31}NO$
Xylamidine Tosylate	Antidepressant	6443-50-1		$C_{19}H_{24}N_2O_2$
Xylazine	Sedative	7361-61-7		$C_{12}H_{16}N_2S$
Xylocoumarol	Anticoagulant	15301-97-0		$C_{17}H_{14}O_3$
Xylometazoline	Decongestant	526-36-3		$C_{16}H_{24}N_2$
Xyloxemine	Antitussive	1600-19-7		$C_{23}H_{33}NO_2$
Yohimbic Acid	Erectile Dysfunction	522-87-2		$C_{20}H_{24}N_2O_3$
Yohimbine	Erectile Dysfunction	146-48-5		$C_{21}H_{26}N_2O_3$
Zabicipril	Antihypertensive	83059-56-7		$C_{23}H_{32}N_2O_5$
Zabiciprilat	Antihypertensive	90103-92-7		$C_{21}H_{28}N_2O_5$
Zabofloxacin	Antibiotic	219680-11-2	Y	$C_{19}H_{20}FN_5O_4$
Zacopride	Antiemetic	90182-92-6		$C_{15}H_{20}ClN_3O_2$
Zafirlukast	Bronchodilator	107753-78-6	Y	$C_{31}H_{33}N_3O_6S$
Zafuleptine	Antidepressant	59209-97-1		$C_{17}H_{26}FNO_2$
Zalcitabine	Antiviral	7481-89-2	Y	$C_9H_{13}N_3O_3$
Zaldaride	Antidiarrheal	109826-26-8		$C_{26}H_{28}N_4O_2$
Zaleplon	Sedative	151319-34-5	Y	$C_{17}H_{15}N_5O$
Zalospirone	Anxiolytic	114298-18-9		$C_{24}H_{29}N_5O_2$
Zaltidine	Antiulcerative	85604-00-8		$C_8H_{10}N_6S$
Zaltoprofen	Antiinflammatory	74711-43-6		$C_{17}H_{14}O_3S$
Zamifenacin	Mydriatic	127308-82-1		$C_{27}H_{29}NO_3$
Zanamivir	Antiviral	139110-80-8		$C_{12}H_{20}N_4O_7$
Zanapezil	Nootropic	142852-50-4		$C_{25}H_{32}N_2O$
Zankiren	Antihypertensive	138742-43-5		$C_{35}H_{55}N_5O_6S_2$
Zanoterone	Steroid	107000-34-0		$C_{23}H_{32}N_2O_3S$
Zapizolam	Anxiolytic	64098-32-4		$C_{15}H_9Cl_2N_5$
Zaprinast	Antihistaminic	37762-06-4		$C_{13}H_{13}N_5O_2$
Zardaverine	Bronchodilator	101975-10-4		$C_{12}H_{10}F_2N_2O_3$
Zatebradine	Antianginal	85175-67-3		$C_{26}H_{36}N_2O_5$
Zatosetron Maleate	Antimigraine	123482-22-4		$C_{19}H_{25}ClN_2O_2$
Zelandopam	Antihypertensive	139233-53-7		$C_{15}H_{15}NO_4$
Zenarestat	Antidiabetic	112733-06-9		$C_{17}H_{11}BrClFN_2O_4$

Table S1. *Cont.*

Common Name	Indication	CAS Number	Oral	Molecular Formula
Zenazocine Mesylate	Analgesic	68681-43-6		$C_{23}H_{35}NO_2$
Zepastine	Antineoplastic	28810-23-3		$C_{22}H_{26}N_2O_3S$
Zeranol	Steroid	26538-44-3		$C_{18}H_{26}O_5$
Zetidoline	Antipsychotic	51940-78-4		$C_{16}H_{22}ClN_3O$
Zidapamide	Antihypertensive	75820-08-5		$C_{16}H_{16}ClN_3O_3S$
Zidometacin	Antiinflammatory	62851-43-8		$C_{19}H_{16}N_4O_4$
Zidovudine	Antiviral	30516-87-1		$C_{10}H_{13}N_5O_4$
Zifrosilone	Antidote	132236-18-1		$C_{11}H_{13}F_3OSi$
Zilantel	Anthelminithic	22012-72-2		$C_{26}H_{38}N_2O_6P_2S_4$
Zileuton	Antiinflammatory	111406-87-2		$C_{11}H_{12}N_2O_2S$
Zilpaterol	Bronchodilator	119520-05-7		$C_{14}H_{19}N_3O_2$
Zimeldine	Antidepressant	56775-88-3		$C_{16}H_{17}BrN_2$
Zimidoben	Unclassified	90697-56-6		$C_{12}H_{12}N_2O_2$
Zindotrine	Bronchodilator	56383-05-2		$C_{11}H_{15}N_5$
Zindoxifene	Antineoplastic	86111-26-4		$C_{21}H_{21}NO_4$
Zinoconazole	Antifungal	84697-21-2		$C_{15}H_{11}Cl_3N_4S$
Zinterol	Bronchodilator	37000-20-7		$C_{19}H_{26}N_2O_4S$
Zinviroxime	Antiviral	72301-78-1		$C_{17}H_{18}N_4O_3S$
Zipeprol	Antitussive	34758-83-3		$C_{23}H_{32}N2O3$
Ziprasidone	Antipsychotic	146939-27-7	Y	$C_{21}H_{21}ClN_4OS$
Zocainone	Unclassified	68876-74-4		$C_{22}H_{27}NO_3$
Zofenopril Calcium	Antihypertensive	81872-10-8	Y	$C_{22}H_{23}NO_4S_2$
Zofenoprilat Arginine	Antihypertensive	75176-37-3		$C_{15}H_{19}NO_3S_2$
Zoficonazole	Antifungal	71097-23-9		$C_{20}H_{19}Cl_3N_2O_2$
Zolamine	Antihistaminic	553-13-9		$C_{15}H_{21}N_3OS$
Zolasartan	Antihypertensive	145781-32-4		$C_{24}H_{20}BrClN_6O_3$
Zolazepam	Sedative	31352-82-6		$C_{15}H_{15}FN_4O$
Zoledronic Acid	Bone Resorption Inhibitor	118072-93-8		$C_5H_{10}N_2O_7P_2$
Zolenzepine	Antiulcerative	78208-13-6		$C_{19}H_{24}N_6O_2$
Zolertine	Vasodilator	4004-94-8		$C_{13}H_{18}N_6$
Zolimidine	Antiulcerative	1222-57-7		$C_{14}H_{12}N_2O_2S$
Zoliprofen	Antiinflammatory	56355-17-0		$C_{12}H_{11}NO_3S$
Zolmitriptan	Antimigraine	139264-17-8	Y	$C_{16}H_{21}N_3O_2$
Zoloperone	Antipsychotic	52867-74-0		$C_{22}H_{24}FN_3O_3$
Zolpidem	Sedative	82626-48-0	Y	$C_{19}H_{21}N_3O$
Zomebazam	Anxiolytic	78466-70-3		$C_{15}H_{16N4O2}$
Zomepirac	Analgesic	33369-31-2		$C_{15}H_{14}ClNO_3$
Zometapine	Antidepressant	51022-73-2		$C_{14}H_{15}ClN_4$
Zonampanel	Antidepressant	210245-80-0		$C_{13}H_9N_5O_6$
Zoniclezole	Anticonvulsant	121929-20-2		$C_{12}H_{10}ClN_3O$
Zoniporide	Cardiotonic	241800-98-6		$C_{17}H_{16}N_6O$
Zonisamide	Anticonvulsant	68291-97-4		$C_8H_8N_2O_3S$
Zopiclone	Sedative	43200-80-2		$C_{17}H_{17}ClN_6O_3$
Zopolrestat	Antidiabetic	110703-94-1		$C_{19}H_{12}F_3N_3O_3S$
Zorubicin	Antineoplastic	54083-22-6		$C_{34}H_{35}N_3O_{10}$
Zosuquidar Trihydrochloride	Antineoplastic	167354-41-8		$C_{32}H_{31}F_2N_3O_2$
Zotepine	Antipsychotic	26615-21-4		$C_{18}H_{18}ClNOS$
Zoticasone	Antiinflammatory	678160-57-1		$C_{25}H_{30}F_2O_6S$
Zoxazolamine	Uricosuric	61-80-3		$C_7H_5ClN_2O$
Zucapsaicin	Analgesic	25775-90-0		$C_{18}H_{27}NO_3$
Zuclomiphene	Pituitary	15690-55-8		$C_{26}H_{28}ClNO$
Zuclopenthixol	Antipsychotic	53772-83-1		$C_{22}H_{25}ClN_2OS$
Zylofuramine	Antidiabetic	3563-92-6		$C_{14}H_{21}NO$
Cetyl Myristate	Dermatologic	2599-01-1		$C_{30}H_{60}O_2$
Cetyl Myristoleate	Dermatologic	64660-84-0		$C_{30}H_{58}O_2$
Emtricitabine	Antineoplastic	143491-57-0	Y	$C_8H_{10}FN_3O_3S$
Metaraminol	Antihypotensive	54-49-9		$C_9H_{13}NO_2$

"Y" represented the drug was administered orally.
